# Substrate Specificity of the Organic Cation Transporters MATE1 and MATE2K and Functional Overlap with OCT1 and OCT2

**DOI:** 10.1021/acs.jmedchem.5c00056

**Published:** 2025-06-13

**Authors:** Kyra-Elisa Maria Redeker, Nicolai Kirsch, Susann Boretius, Mladen Tzvetkov, Jürgen Brockmöller

**Affiliations:** † Institute of Clinical Pharmacology, University Medical Center Göttingen, Georg-August-University, D-37075 Göttingen, Germany; ‡ Functional Imaging Laboratory, German Primate Center, Leibniz Institute for Primate Research, D-37077 Göttingen, Germany; § Department of General Pharmacology, Institute of Pharmacology, Centre of Drug Absorption and Transport (C-DAT), University Medical Centre Greifswald, D-17487 Greifswald, Germany

## Abstract

The multidrug and toxin extrusion proteins MATE1 and MATE2K may determine the pharmacokinetics and drug–drug interactions of many drugs. However, their substrate spectrum and synergy with organic cation transporters OCT1 and OCT2 remain incompletely understood. Therefore, we screened 590 predominantly positively charged, low molecular weight compounds for transport via these four transporters in HEK293 cells using high-performance liquid chromatography-tandem mass spectrometry (HPLC-MS/MS). MATE1 and MATE2K transported 164 and 114 compounds, respectively, with significant overlap. High-affinity substrates included berberine, pentamidine, and amisulpride, while epinephrine and atenolol had the highest *V*_max_. Despite less than 16% sequence homology, there was high overlap among MATE1/-2K and OCT1/-2 substrates. Neither isolated physicochemical properties nor their linear combinations predicted the substrates of these organic cation transporters. However, machine learning classifiers using 15 parameters allowed 69 to 87% correct prediction. The large number of substrates indicates a possibly broad role of multidrug and toxin extrusion (MATE) transporters in pharmacokinetics and drug interactions.

## Graphical abstract

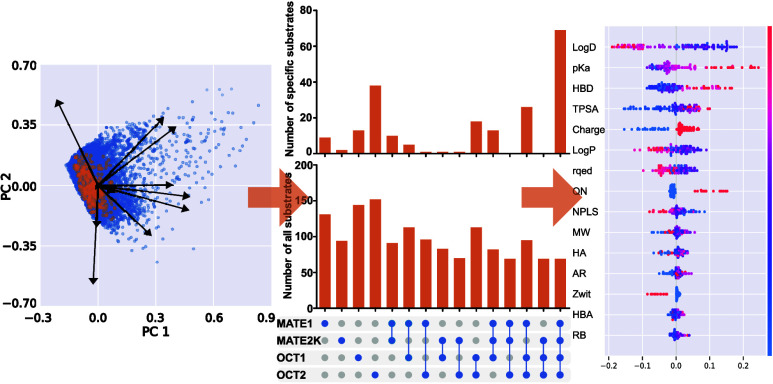

## 1 Introduction

Carrier-mediated membrane transport plays a crucial role in drug absorption, elimination, and effective delivery to target sites within the human body. For most drugs, passive diffusion—including nonionic diffusion of the uncharged fraction—is too slow to achieve therapeutic relevance. In particular, the transport of organic cations across biological membranes relies on carrier-mediated mechanisms. These include either facilitated passive (equilibrative) transport along concentration gradients or antiport mechanisms, where organic cations are exchanged for protons or other cations. The organic cation transporters OCT1 and OCT2 (SLC22A1 and -A2) were first identified as polyspecific carriers capable of transporting a broad range of organic cations^1–3^ and early data already showed that most substrates of those two organic cation transporters share key characteristics: a molecular weight below 500 Da, relatively hydrophilic properties (log *D*_pH 7.4_ < 1.5) and a positive charge due to protonation at physiological pH. Further screening confirmed these findings, also demonstrating that OCT1 and OCT2 can transport some neutral or even negatively charged substrates.^4–6^ However, negatively charged substrates were transported mainly with very low rates and low affinity (*K*_M_), making medical or biological relevance of anion transport by OCTs questionable.

The multidrug and toxin extrusion (MATE) proteins are another family of polyspecific organic cation transporters initially identified as bacterial efflux transporters.^7^ Later, the human homologues MATE1 and MATE2 (SLC47A1 and -A2) were identified as antiporters that exchange organic cations for protons.^8^ While genes coding for OCT1, −2, and −3 are clustered on chromosome 6q25.3, MATE1 and MATE2K are clustered on chromosome 17p11.2. While OCT1, −2, and −3 belong to the Major Facilitator Superfamily (MFS) of transporters, the MATE transporters are part of the Multidrug/Oligosaccharidyl-lipid/Polysaccharide (MOP) Flippase superfamily and operate via a different transport mechanism.^9^ MATE2 is predominantly expressed as a splice variant lacking 36 amino acids between the fourth and fifth transmembrane domains.^10^ Based on its identification in the kidney, this splice variant is named MATE2K. Both OCTs and MATEs share substrates such as tetraethylammonium (TEA) and 1-methyl-4-phenylpyridinium (MPP^+^).^8,11,12^

Functional interactions between OCTs and MATEs may play a crucial role in the renal and hepatic elimination of cationic drugs and particularly in the protection of excretory tissues from toxin accumulation.^8,13,14^ The elimination of endobiotics or xenobiotics follows specific pathways from the blood through the hepatocytes into the bile duct or from the blood through the renal tubular epithelia into the lumen of the proximal tubules, respectively. The expression of OCT1 on the basolateral side of hepatocytes allows the uptake of substances from the blood, while MATE1, expressed on the apical membrane, may transport substrates into the bile ducts for elimination. However, that role of MATE1 in the liver is still not well experimentally confirmed. In the kidneys, OCT2 is expressed on the basolateral membrane of tubular epithelial cells for transport from the blood into the tubular cells, while MATE1 and MATE2K excrete into the proximal tubules (Figure 1).

**Figure 1:**
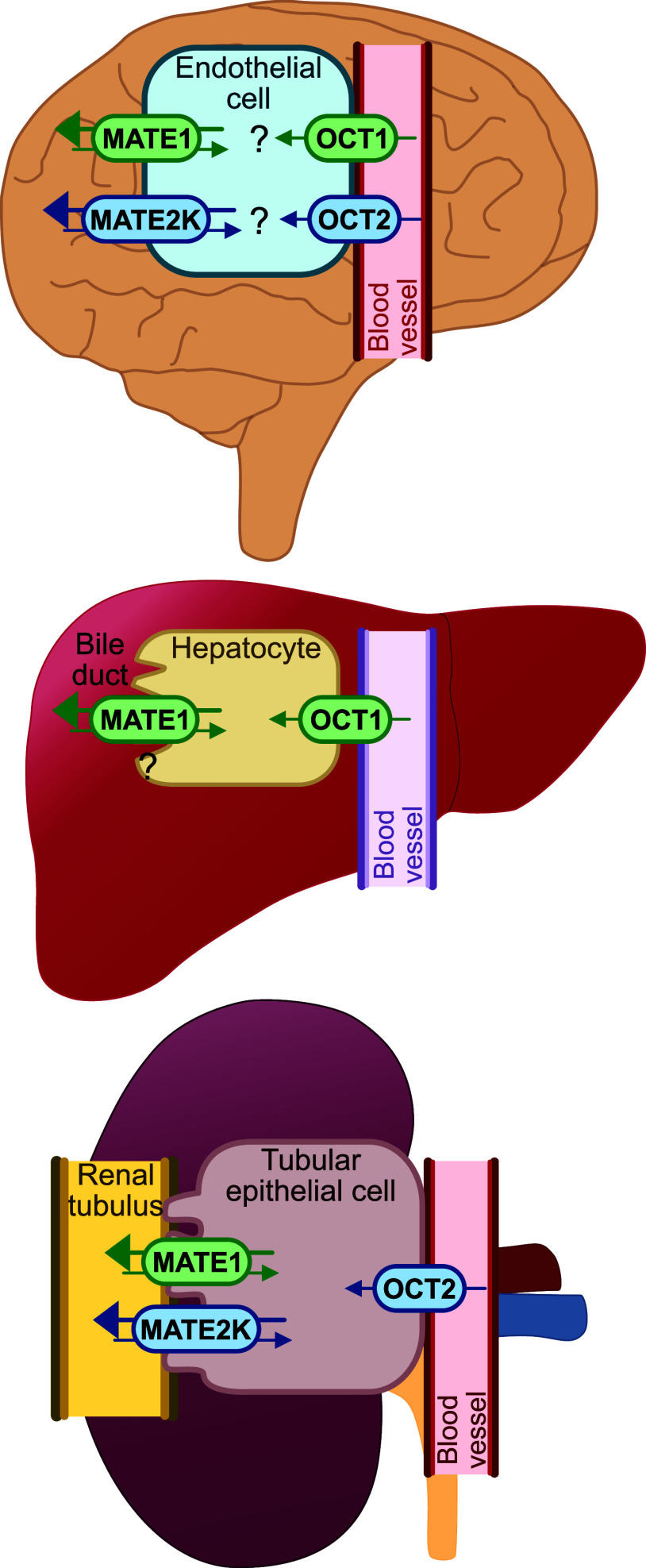
Membrane transport via MATEs and OCTs in the brain, liver and kidneys. The figure illustrates potential interactions between OCTs and MATEs. It also highlights their roles in vectorial transport of substances from the blood into bile in the liver and from the blood into the primary urine in the renal tubular ducts. The precise functions of MATEs in the brain and liver remain incompletely understood.

The interaction between these transporters and their vectorial transport has been experimentally studied in the Madin-Darby Canine Kidney (MDCK) cell model. In vivo, both MATE proteins enhance efflux transport in the direction of higher proton concentration. In the present study, we employed stable overexpression in HEK293 cells and created an acidic intracellular condition by 30 min preincubation with 30 mM ammonium chloride.^15,16^

Also, the expression of not only OCT1 and −2 but also MATE1 and MATE2 at the blood–brain barrier has been shown.^17^ Therefore, a similar transport process might occur from the blood into the brain across the blood–brain barrier, enabling the delivery of organic cations to the brain (Figure 1). However, despite their expression in the brain, the exact function of both MATE transporters in the brain is still not unequivocally confirmed.

Over the past two decades, several organic cation transporters and their substrates have been identified.^4,18,19^ However, for most organic cations, we still do not know the relevant transporters. Knowing these transporters may be essential in drug development,^20^ in drug therapy, and toxicology, as it can help to predict interactions and the effects of genetic variation.^21^ In particular, many structurally diverse low molecular weight substances are transported via the proton organic-cation antiporter.^22^ However, the proteins catalyzing this transport activity have not yet been fully identified.^12^ Both MATE1 and MATE2K function as proton organic-cation antiporters, with 81 known substrates for MATE1 and 48 for MATE2K, though not all have been conclusively confirmed as substrates (Table S1).^10,18,21,23–28^ In this study, we screened a wide range of predominantly positively charged low molecular weight substances as potential substrates of MATE1 and MATE2K. Our aim was to expand the understanding of their substrate and nonsubstrate profiles. Additionally, we examined the overlap between the substrate spectra of MATE1 and MATE2K and those of organic cation transporters OCT1 and OCT2. This may help to understand drug–drug interactions but it may also contribute to our understanding of the biological roles of these transporters.

## 2 Results

In this study, we conducted a comprehensive in vitro screening of MATE1 and MATE2K substrates, focusing on positively charged endogenous compounds and drugs from diverse therapeutic classes. This should allow the identification of drugs affected by MATE-mediated drug interactions, but it should also allow a better definition of structural features of MATE substrates. We then compared transport across MATE, MATE2K, and OCT1 and −2 to identify common substrates and those selectively transported by one or two transporters.

### 2.1 Uptake Ratios of MATE1 and MATE2K

Using a widely accepted uptake ratio threshold of at least 2 under nonsaturating substrate concentrations,^20^ 164 (27.8%) MATE1 substrates were identified, with uptake ratios ranging from 2 to 40. Of these MATE1 substrates, 129 (78.7%) are described here for the first time. In comparison, fewer substances were substrates of MATE2K, with 114 (19.3%) substrates and uptake ratios ranging from 2 to 31.5, of which 91 were not known MATE2K substrates before (Figure S1 and Table S2).

There was substantial overlap in substrate specificity, with 107 of the 114 substrates for MATE2K also being substrates for MATE1. Only eight compounds were transported better by MATE2K than by MATE1, most notably the β-2 agonist reproterol, with an uptake ratio of 17.9 for MATE2K and 5.2 for MATE1. Riboflavin (vitamin B2) is a substrate of MATE2K with a transport ratio of 4.5, but with an uptake ratio of 1.9 it is only a poor substrate of MATE1. Other substances with higher transport efficiency via MATE2K included the pro-apoptotic investigational substance sepantronium and the β-2 agonist olodaterol. In general, however, uptake ratios for most compounds were significantly lower for MATE2K compared to MATE1, indicating that MATE1 has a broader and more efficient uptake capacity for many substrates. Natural substrates with high MATE1 uptake ratios included palmatin, thiamine, and tyramine, suggesting a potential role of MATEs in handling both herbal xenobiotics and endobiotics.

As the Supporting Figure S1 illustrates, MATE1 transported 100 substrates with uptake ratios ranging from 8 to 40. The substrate with the highest uptake ratio was arcaine (also known as 1,4-diguanidinobutane), which is an experimental antagonist at the polyamine binding site of the NMDA receptor. Following arcaine in uptake efficiency at the 2.5 μM substrate concentration were dopamine, the experimental local anesthetic drug *N*-ethyl-lidocaine, and the β-2-agonist metaproterenol (orciprenaline). Other substances with notably high transport rates were the endogenous sympathomimetic deoxyepinephrine (also known as 2-phenylethylamine or phenylethanolamine) and *N*-methylnicotinamide, a biologically active metabolite of nicotinamide (one form of vitamin B3, niacin).

Figure 2 present a summary of pharmacological substance groups, highlighting the large number of good substrates of MATE1. Notably, cholinergic and anticholinergic drugs, betablockers, and sympathomimetics included many good substrates. Similarly, biguanides, H2 antihistamines, and antimigraine triptans were good substrates for both MATE1 and MATE2K.

**Figure 2:**
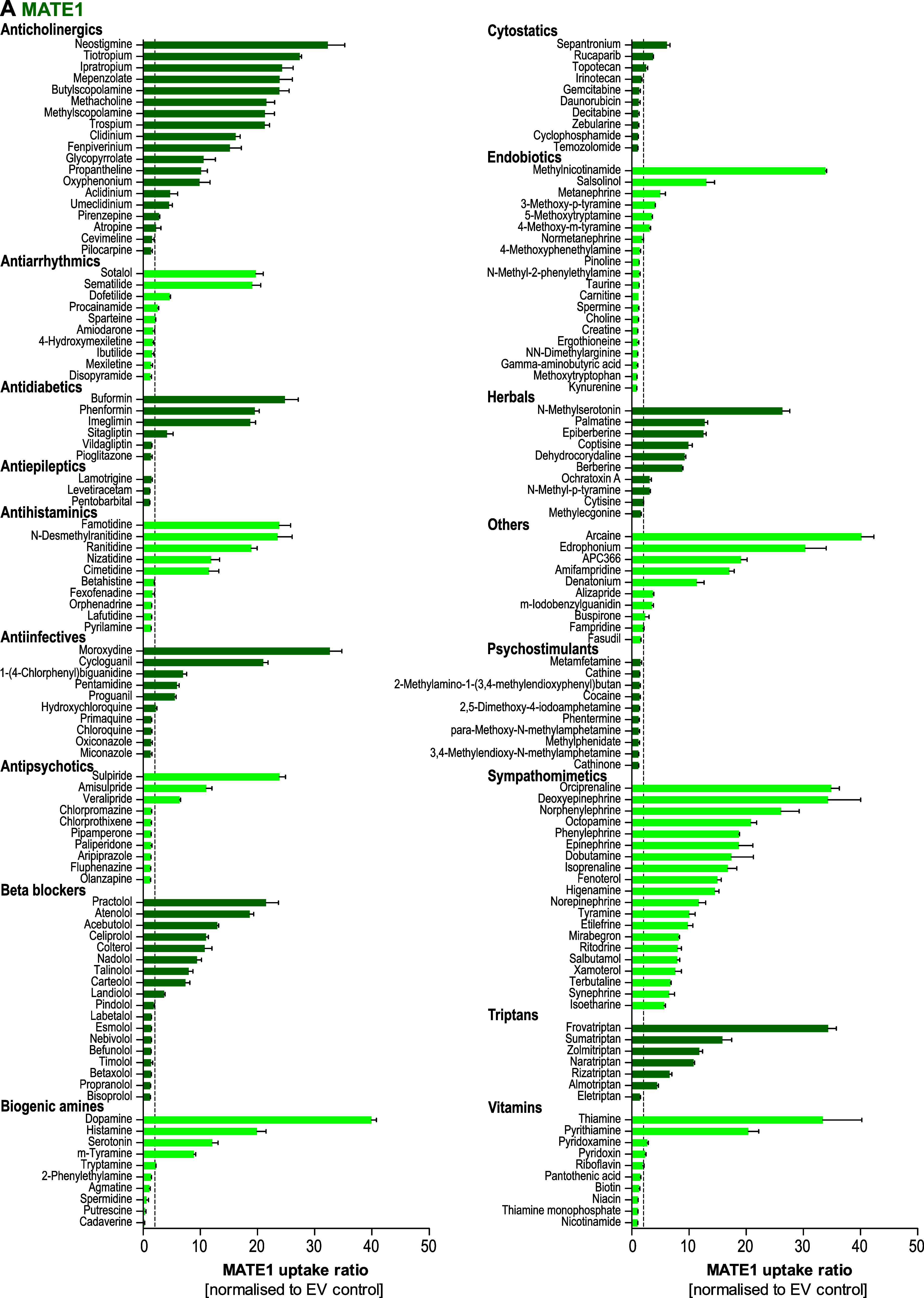

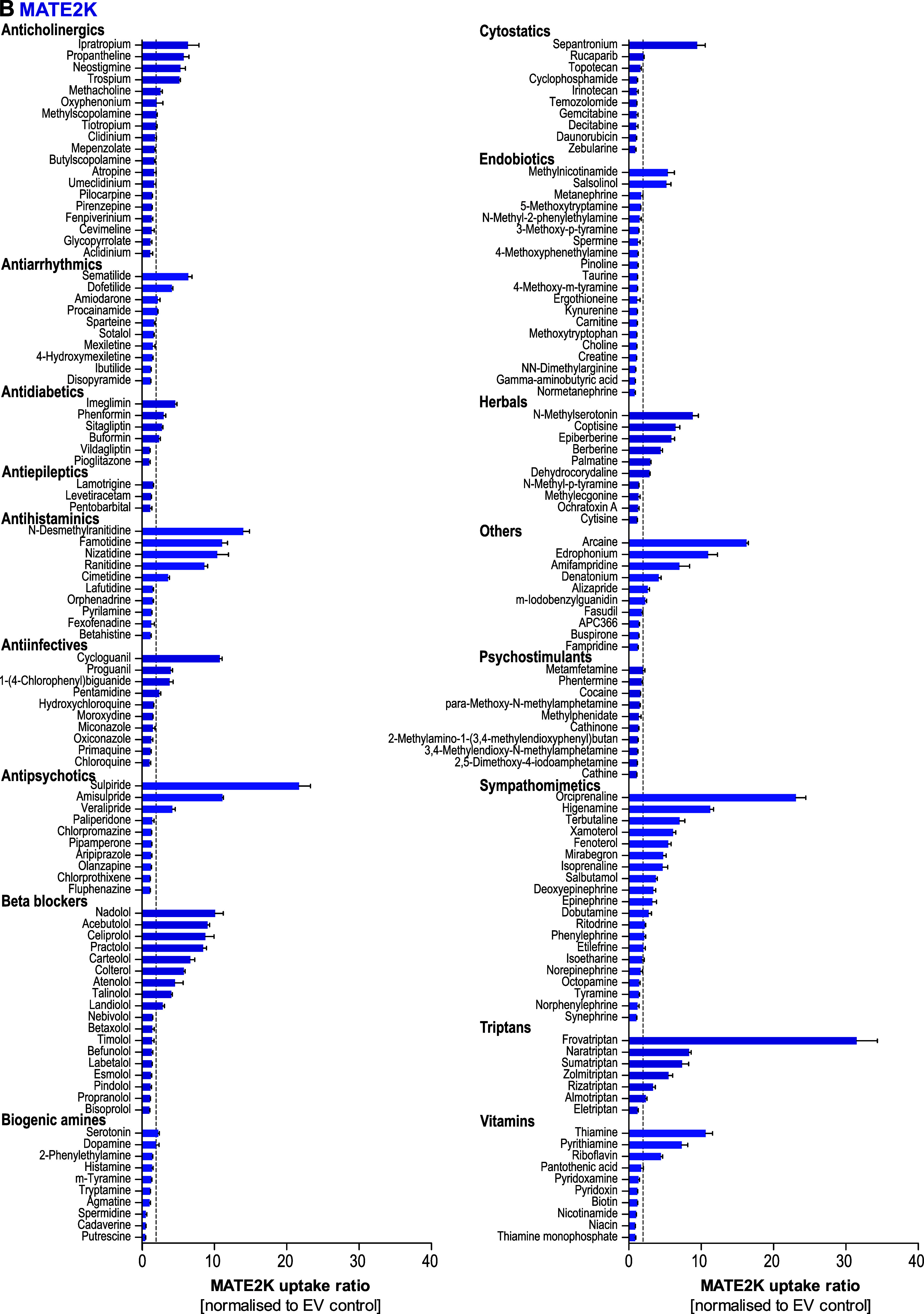
Substrates of MATE1 (A) and MATE2K (B) with representative uptake ratios across 16 therapeutic drug groups. HEK293 cells overexpressing MATE1 (green), MATE2K (blue) or empty vector (EV)-transfected control cells were incubated with 2.5 μM of each substance for 1 min. Intracellular substrate concentration was quantified by HPLC-MS/MS analysis. A total number of 590 substances were tested, and 10–20 substrates from 16 different therapeutic groups are displayed. Uptake is expressed as fold increase in transporter-overexpressing cells over EV-transfected control cells and presented as mean ± SEM of three independent experiments. The dashed horizontal lines at 2.0 indicate the transport ratio above which the transport is generally considered medically relevant.

In contrast, none of the tested psychostimulants or 5HT3 antagonists met the set cutoff of 2 and, therefore, were not considered substrates of either transporter. While MATE1 showed highly increased uptake for several biogenic amines, such as dopamine, this could not be observed for MATE2K. Among local anesthetics and muscle relaxants, only the experimental quaternary amine *N*-ethyl-lidocaine and vecuronium showed markedly increased uptake for both MATE transporters (Figure S1).

### 2.2 Transport Kinetic Characterization of MATE1 and MATE2K-Mediated Uptake

Concentration-dependent uptake was analyzed for 40 substances, including those with very high uptake ratios at the 2.5 μM concentration as well as selected substrates with moderate uptake ratios. Figure 3 illustrates concentration-dependent uptake via MATE1 and MATE2K for 9 medically relevant substances. The transport kinetic parameters are listed in Table 1 and the complete concentration dependent uptake in Figure S2.

**Figure 3:**
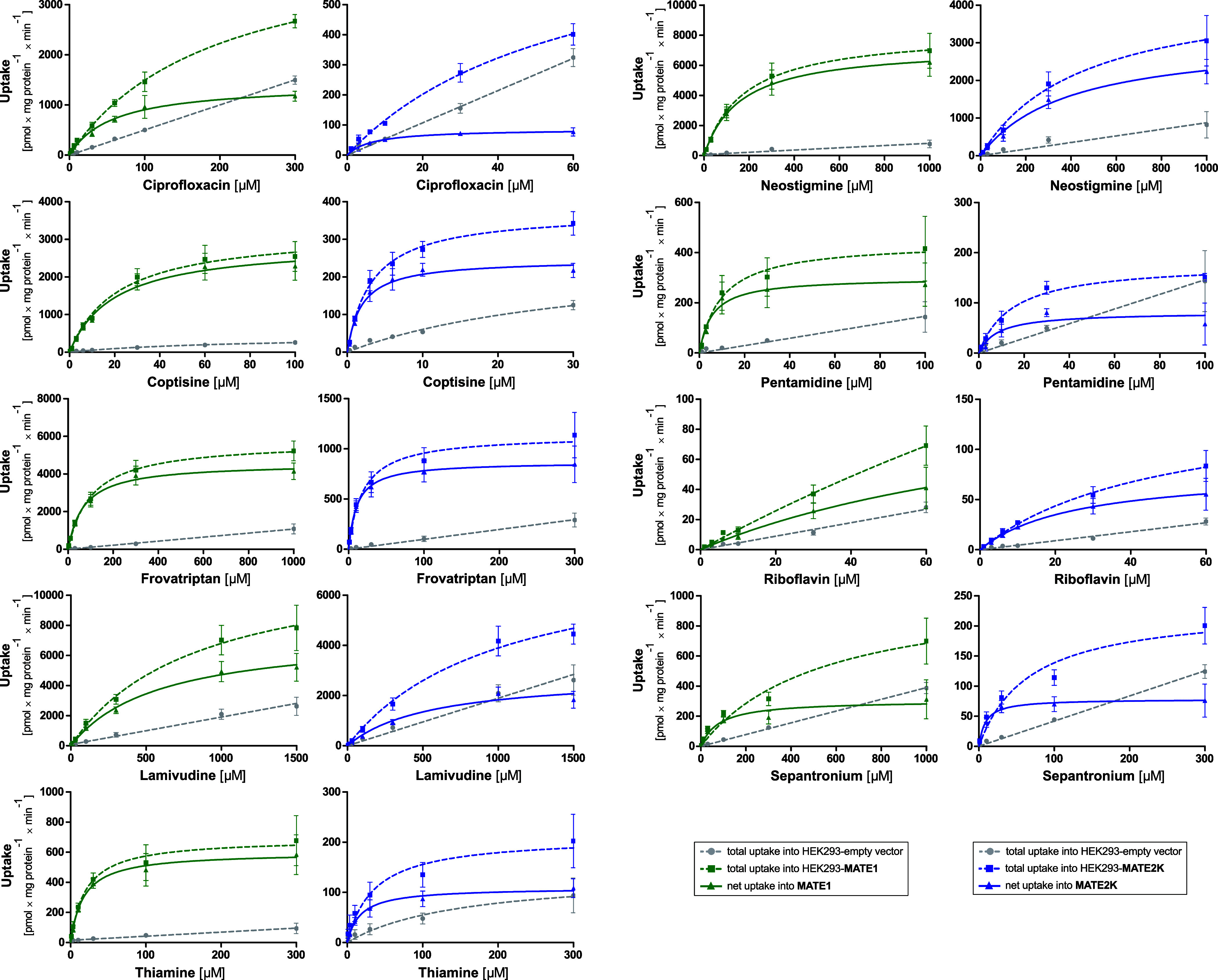
Concentration-dependent transport of selected substances by MATE1 and MATE2K. HEK293 cells overexpressing MATE1, MATE2K, or empty vector (EV)-transfected control cells were incubated with various substance concentrations for 1 min. Overall, 40 substrates were tested with concentration-dependent transport (Figure S2), of which nine are displayed here. Dashed green or blue curves represent total uptake by cells overexpressing MATE1 or MATE2K, while the gray lines indicate total uptake by empty vector-transfected cells. Solid green or blue curves display the net uptake by the respective transporter. Data are displayed as mean ± SEM of three independent experiments. For most substrates, the uptake into the empty vector-transfected cells was linear, thus probably being due to simple or nonionic diffusion. Only for thiamine and coptisine, also in the empty vector cells, some saturation could be detected, probably best explained by the activity of transporters constitutively expressed in the HEK293 cells.

**Table 1:** Transport Kinetic Parameters^a^

	MATE1					MATE2K				
Substance	*V*_max_ [pmol × mg protein^–1^ × min^–1^]	SEM	*K*_M_ [μM]	SEM	Cl_int_ [μL × mg protein^–1^ × min^–1^]	*V*_max_ [pmol × mg protein^–1^ × min^–1^]	SEM	*K*_M_ [μM]	SEM	Cl_int_ [μL × mg protein^–1^ × min^–1^]
4-aminoantipyrine	linear					linear				
Acyclovir	4920	2293	3031	1821	1.62	975	660	1289	1411	0.76
Amiodarone	linear					linear				
Amisulpride	238	32.4	**10.6**	6.33	**22.4**	710	104.3	39.9	19.2	**17.8**
Atenolol	7391	1346	195	103	**38.0**	1792	288	173	83.8	**10.3**
Atenolol (R)-	13,025	444	202	19.9	**64.4**	2965	177.5	181	32.2	**16.4**
Atenolol (S)-	11,907	1033	177	45.9	**67.3**	2677	547	155	98.2	**17.3**
Atropine	397	53.6	**17.8**	7.75	**22.3**	linear				
Berberine	2521	387	**6.99**	4.01	**361**	203	39.03	**0.975**	0.753	**209**
Ciprofloxacin	1426	160.6	57.7	17.9	**24.7**	86.7	7.87	**6.83**	2.15	**12.7**
Coptisine	2901	301.1	**20.0**	6.25	**145**	246	16.04	**1.88**	0.497	**131**
Dobutamine	2586	357.6	88.6	43.4	**29.2**	493	107	86.4	49.7	**5.71**
Dopamine	10,148	2473	530	329.1	**19.2**	1219	613.4	961	858.8	1.27
Emtricitabine	4523	203.3	992	88.77	4.56	2634	327.4	873	226.8	3.02
Epinephrine	22,773	4499	1080	411.6	**21.1**	2968	800.4	1508	626.4	1.97
Frovatriptan	4568	280.5	70.4	16.07	**64.9**	869	77.04	**11.2**	4.29	**77.8**
Imeglimin	6242	553.8	58.0	18.6	**108**	2814	383.5	81.6	37.17	**34.5**
Ipratropium	1646	157	54.4	18.7	**30.3**	432.6	25.7	98.7	18.0	4.38
Lamivudine	7481	1108	586	213.2	**12.8**	2838	435.8	553	213.1	**5.13**
Methacholine	17,843	4877	1987	846.8	**8.98**	1240	354.1	1404	703	0.88
Methylnaltrexone	55.8	6.6	**17.9**	6.85	3.12	41.6	19.9	86.8	75.5	0.48
Milnacipran	1796	335.4	94.9	43.3	**18.9**	2377	1328	351.8	319.8	**6.76**
Neostigmine	7255	815	160	55.3	**45.2**	3139	469.1	385.4	137.9	**8.14**
Norepinephrine	18,720	2041	1866	323.7	**10.0**	673	169.4	1151	545.3	0.59
Pentamidine	300	52.0	**5.67**	3.86	**52.8**	81.2	18.4	**8.25**	6.24	**9.84**
Phenformin	8451	441.2	101	18.11	**83.9**	1361	96.05	73.1	19.08	**18.6**
Phenylephrine	11,611	2238	474	204.8	**24.5**	776	382.3	434	332.3	1.79
Pyridoxamine	5442	2376	1687	1098	3.23	989.5	712.7	1553	1708	0.64
Pyridoxin	10,014	7784	4228	4208	2.37	593	217.3	495	467.5	1.20
Rasagilin	194	497.7	167	545.8	1.17	linear				
Riboflavin	115	105.7	108	140	1.07	79.4	20.44	**25.5**	14.81	3.11
Sepantronium	304	58.8	80.9	56.6	3.76	78.5	10.9	**8.54**	5.98	**9.19**
Sulpiride	851	44.8	**16.0**	3.59	**53.2**	1025	36.3	**22.8**	3.08	**44.9**
Sumatriptan	5693	280	95.8	16.4	**59.4**	1733	80.78	72.3	12.49	**24.0**
Thiamine	599	64.1	**17.2**	7.26	**34.9**	109.2	11.6	**17.3**	7.26	**6.32**
Tyramine	5948	602	136.5	44.2	**43.6**	2868	1034	1060	656.9	2.71
Zalcitabine	10,171	1676	1558	426.6	**6.53**	linear				
Zolmitriptan	2992	334	57.1	17.46	**52.4**	740	100.7	**23.1**	11.79	**32.1**
Zolmitriptan (R)-	2624	260	**27.8**	9.75	**94.5**	992	138.8	**18.3**	10.43	**54.2**
Zolmitriptan (S)	2849	373	43.7	17.5	**65.2**	1662	412.8	94.5	56.5	**17.6**

a *V*_max_: pmol × mg protein^–1^ × min^–1^, *K*_M_: μM, Cl_int_ (intrinsic clearance): μL × mg protein^–1^ × min^–1^; SEM, standard error of the mean. *K*_M_ values <30 and Cl_int_ > 5 are marked in bold characters.

Considering that ciprofloxacin is uncharged at neutral pH, it is noteworthy that it exhibited one of the lowest *K*_M_ values for MATE2K substrates (6.8 μM). However, the corresponding intrinsic clearance was only moderate. In contrast, the herbal isoquinoline alkaloids berberine and coptisine demonstrated extremely high intrinsic clearances for both MATE transporters—361 and 145 μL/mg protein/min for MATE1, and 209 and 131 μL/mg protein/min for MATE2K, respectively (Table 1).

Riboflavin (vitamin B2) and the antiapoptotic investigational anticancer substance sepantronium showed higher uptake ratios for MAETE2K compared to MATE1, corroborated by concentration-dependent uptake analysis (Figure 3). Both had significantly lower *K*_M_ values and a higher intrinsic clearance for MATE2K.

Another aspect of membrane transport, stereoselectivity, was tested with 32 racemic compounds (Figure 4). However, overall, transport via MATEs did not very much differ between the enantiomers. Enantiomers of atenolol and zolmitriptan were evaluated concentration-dependently as substrates of MATE1 and MATE2K to assess in more detail potential stereospecificity in MATE-mediated transport (Figure 4).

**Figure 4:**
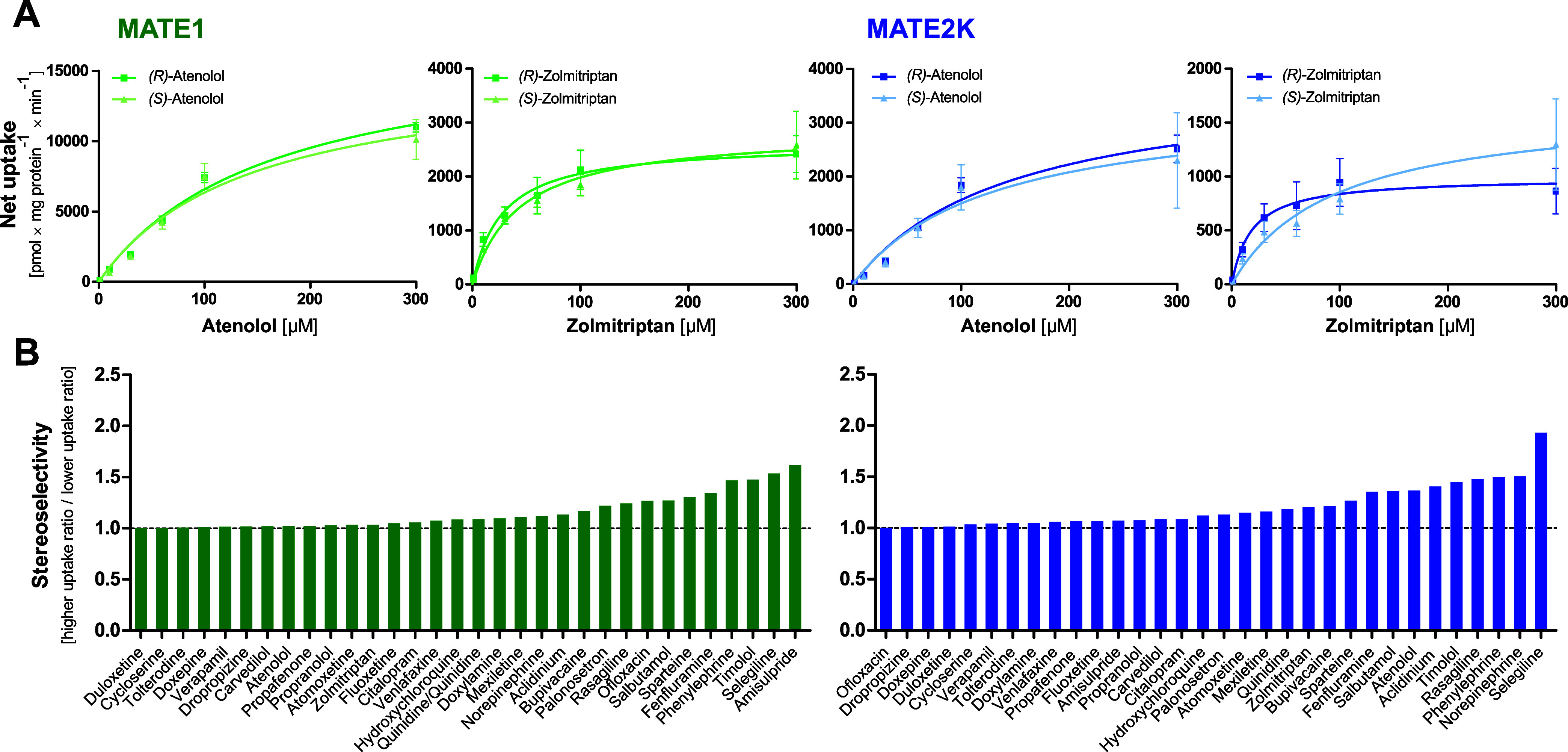
Stereoselective uptake of selected substances by MATE1 and MATE2K. (A) HEK293 cells overexpressing MATE1 (green) and MATE2K (blue) cells were incubated with various concentrations of atenolol and zolmitriptan for 1 min to evaluate uptake. The green and blue curves represent net uptake by MATE1 or MATE2K, respectively, calculated by subtracting the total uptake from the uptake observed in empty vector-transfected cells. (B) Incubation time for substrate uptake was one minute at a concentration of 2.5 μM. The stereoselectivity is expressed as the ratio of uptake by the enantiomer with the higher value to that of the enantiomer with the lower value.

The racemate exhibited a lower *V*_max_ for atenolol resulting in reduced intrinsic clearance compared to either of the single enantiomers. In contrast, zolmitriptan showed distinct stereospecific differences: both MATE1 and MATE2-K displayed a lower *K*_M_ for the R-enantiomer, whereas the S-enantiomer had a significantly higher *K*_M_, particularly for MATE2-K, resulting in a reduced intrinsic clearance.

However, Figure 4B illustrates that several other racemic drugs demonstrated little to no enantio-specific uptake by MATE1 and MATE2-K. The R-enantiomer of amisulpride exhibits a slightly lower ratio in MATE1 transport compared to its racemic form and the other enantiomer. In case of epinephrine, the R-enantiomer shows a ratio approximately twice as high as the racemate in MATE1 transport. In contrast, the MATE2K transporter exhibits the same ratio for both the R-enantiomer and the racemate (Table S3).

### 2.3 Comparison and Correlation of MATE1, MATE2K, OCT1, and OCT2 Transport Activities

A presumed consecutive transport mechanism involving coordinated elimination via OCTs and MATEs (Figure 1) would require overlapping substrate spectra among these transporters. Correlations among all cell uptake activities are illustrated in Figure 5. To ensure meaningful significance levels (i.e., avoiding bias from the many not or only minimally transported substrates) the significance of the correlations, as shown in Figure 5, was calculated exclusively from ratios above 2.0.

**Figure 5:**
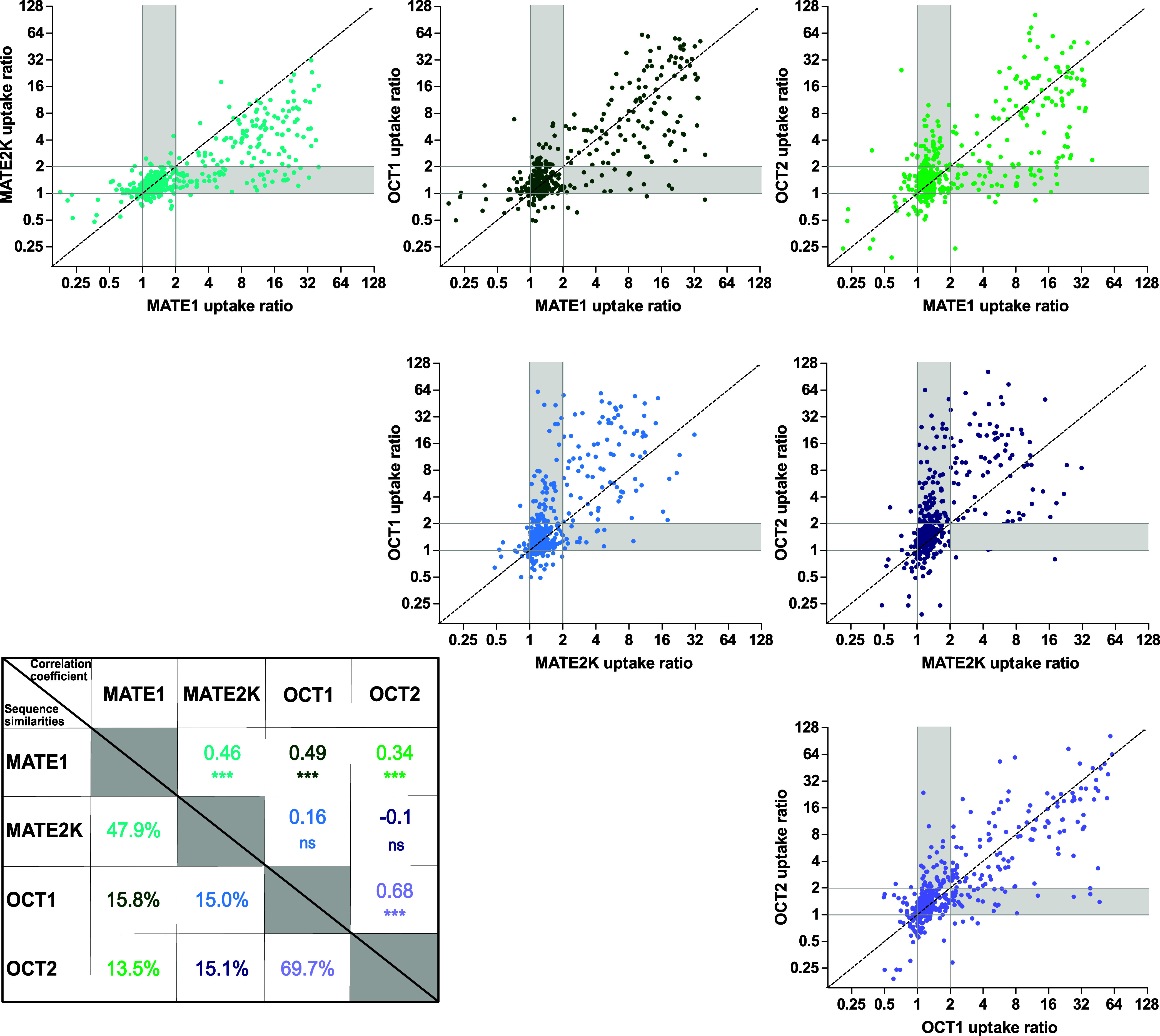
Correlation of MATE1, MATE2K, OCT1, and OCT2 uptake ratios. The graphs display the correlation of the uptake ratios for each transporter pair, with dashed lines indicating the bisector representing equal transport. The accompanying table provides the Spearman correlation coefficients and their significance levels (*** *p*< 0.0001; ns *p* > 0.05) calculated with GraphPad Prism (version 5.01) in comparison to sequence similarities for each transporter pair (analyzed using Geneious Prime 2023.0.1). Gray-shaded areas in the respective correlation graphs highlight substances exhibiting some specificity for one transporter over the other. These substances may serve as valuable probes for transporter-specific in vitro or in vivo, as explored in greater detail in Figure 6 and Table 2. Data points for OCT1 and OCT2 include partly unpublished data of the authors and partly previously published data.^4,6,19^ Notably, the slopes of the correlations between each of the transporters (not shown in the graphs) do not only depend on the specific transport activities of each transporter but also the expression of each transporter. In our experiments, the latter was stable within each stably transfected cell line, but we did not confirm whether it was identical for the different transporters.

Notably, the substrate uptake ratios of MATE1 and OCT1 demonstrated a strong correlation across high, moderate, and low uptake ratios, with a Spearman correlation coefficient of 0.49 compatible with joint hepatic elimination via these two transporters (Figures 1 and 5). In contrast, the similarity in substrates between the predominantly renaly expressed MATE2K and OCT2 was relatively low. For example, several OCT2 substrates with exceptionally high uptake ratios (>20) were only moderate substrates of MATE2K, with uptake ratios between 2 and 8. When comparing MATE2K and OCT2 activities it should however considered that the protein concentration neither in our overexpressing cell lines not in the human kidney is thus far well characterized.

Not only are shared substrates of interest, but also those specific for one of the transporters compared to another or all three others (Figure 6 and Table 2). While most MATE2K substrates are also substrates of MATE1, the reverse is not true, as there were good substrates of MATE1 based on the transport ratio cutoff of 2 which were no substrates of MATE2K. Interestingly, OCT2 had a relatively high number of specific substrates not shared with the other transporters. However, clearly illustrated in Figure 6, all four transporters share many common substrates (*n* = 69).

**Figure 6:**
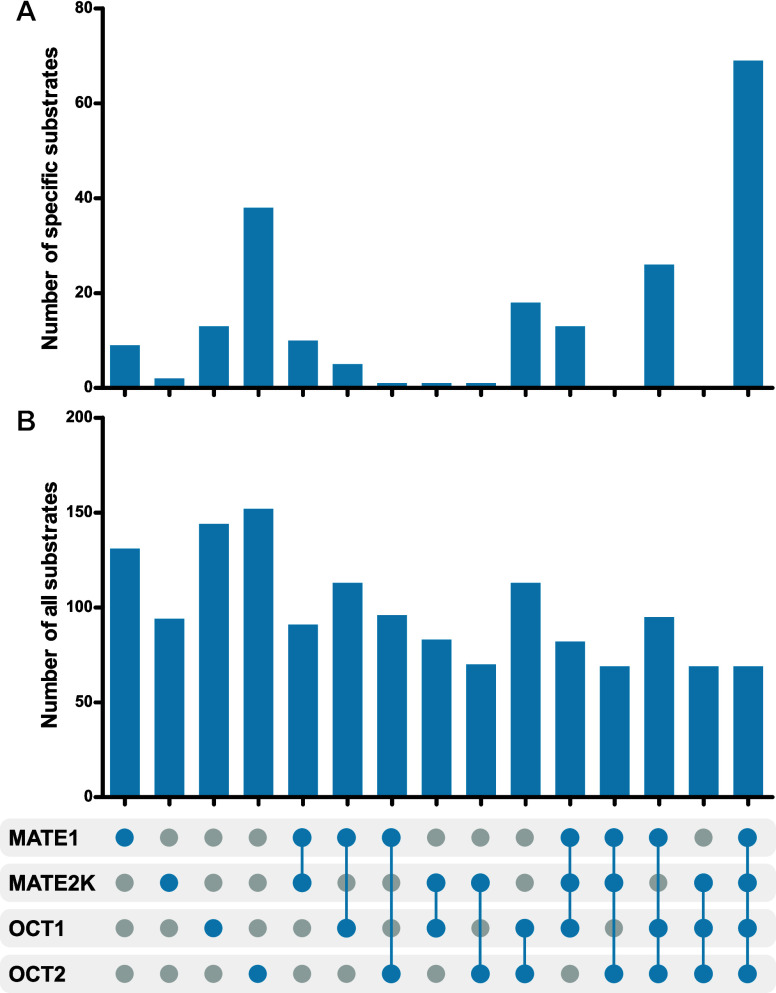
Substrate specificity and substrate overlap of MATE1, MATE2K, OCT1, and OCT2. The UpSet plot illustrates substrate specificity and overlap among the four transporters. (A) The upper plot displays the number of specific substrates exclusive to each transporter or transporter combination. (B) The lower part shows all substrates identified for each respective transporter or transporter combination regardless of the transport of the respective substrates by other transporters.

**Table 2:** Substrate Specificity and Overlap among the Four Transporters

Transporter	Number of substrates	Examples of substrates
MATE1 only	9	Ciprofloxacin, nafamostat, midodrine, pyridoxamine, topotecan
MATE2K only	2	Pethidine (meperidine), amiodarone
OCT1 only	13	Noroxycodone, hexylamine, trimethoprim, normetanephrine
OCT2 only	38	Fampridine, methylphenidate, minoxidil, cathine, ephedrine
All four transporters	69	*N*-Ethyl-lidocaine, methylnaltrexone, neostigmine, orciprenaline, frovatriptan, thiamine, cycloguanil

From a protein structure–activity point of view, it is unsurprising that transporters with high sequence homology, such as MATE1 and MATE2K or OCT1 and OCT2, also exhibit strong correlations in their substrate spectra (Figure 5). However, intriguingly, MATE1 and OCT1–despite sharing only about 16% sequence similarity–demonstrated a high degree of overlap in substrate specificity, comparable to that of MATE1 and MATE2K. This observation suggests that functional evolutionary convergence may play a significant role in shaping substrate profiles, making MATE1 and OCT1 particularly intriguing pairs for further study using methods of recombinant gene technology and structural biology to elucidate the structure–activity relationships.

Table 2 highlights representative substances that are either unique to a single transporter or shared among all of the four transporters studied. It is important to note, however, that “substrate specificity” is mostly not an absolute yes-or-no characteristic but rather a matter of relative preference, as depicted in the correlation graphs in Figure 5.

Substrates of multiple MATE and OCT transporters were from various therapeutic drug classes (Figures 6 and 7). Notably, these included the choline esterase inhibitor neostigmine and the anticholinergic drug ipratropium, H2 antihistaminics, such as famotidine and ranitidine, sympathomimetics, such as orciprenaline, and triptans including Sumatriptan and frovatriptan.

**Figure 7:**
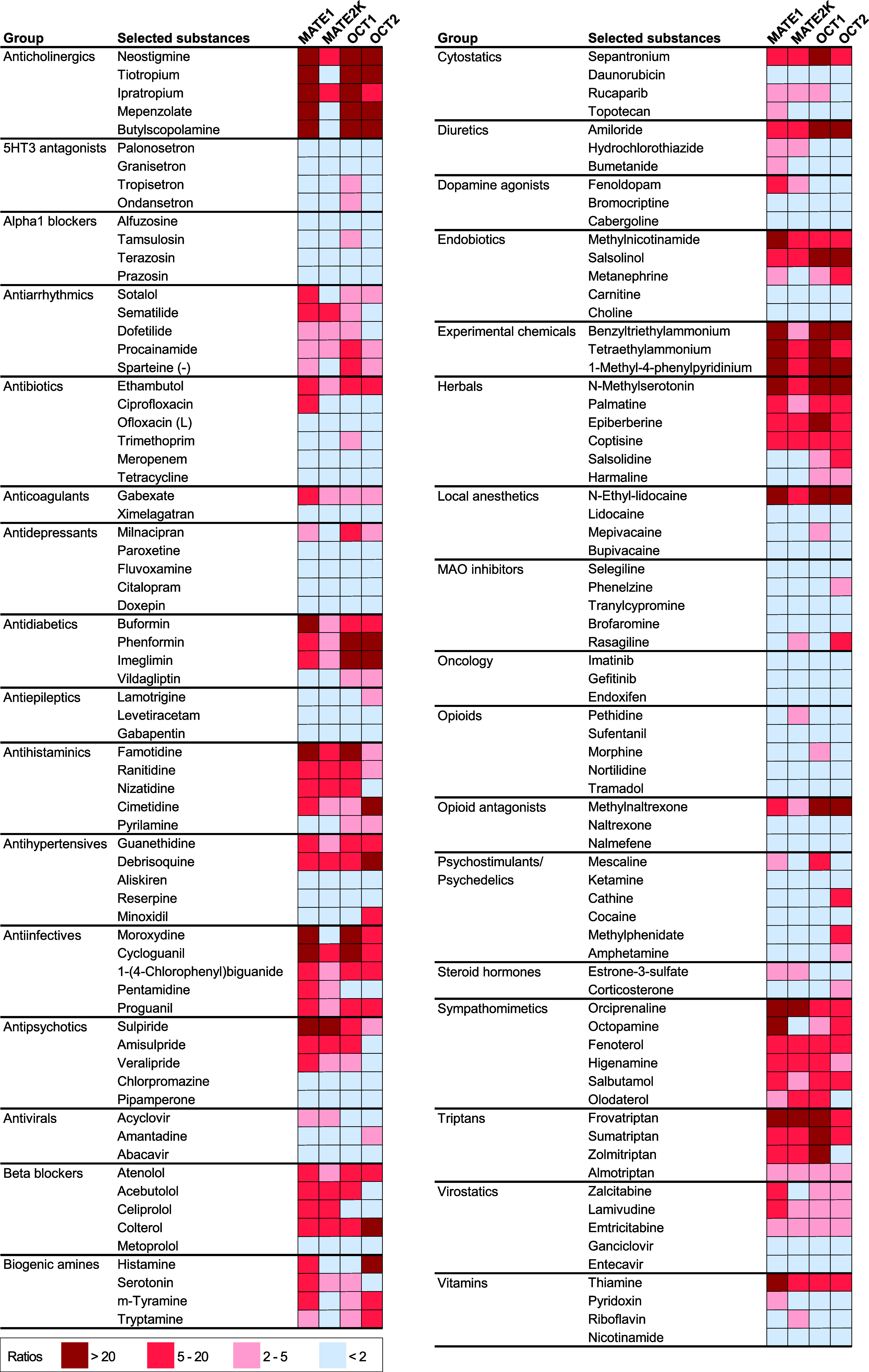
Overlapping versus specific transport via MATE1, MATE2K, OCT1, and OCT2 in different therapeutic groups. The heat map illustrates uptake ratios by all four transporters for selected substances across various therapeutic groups. Color coding indicates the uptake ratio: dark red for ratios >20, light red for ratios between 5 and 20, rose for ratios between 2 and 5, and light blue for ratios below 2. As illustrated, there is a substantial overlap of substrates of all four transporters, but there are also substances only transported by one of the transporters.

Interestingly, certain therapeutic groups contained nonsubstrates of all the four transporters studied here, such as alpha1 blockers, anticancer drugs, or most opioids. However, most drug classes contained a mixture of substrates and nonsubstrates by one or many transporters. That indicates that the specific pharmacophores determining receptor binding (or other targed protein binding) and being a substrate of one a specific organic cation transporter is not identical, even though strong similarities in physicochemical properties do exist.

Then, we analyzed whether being a substrate of the predominantly renal transporters MATE2K and/or OCT2 could predict a high rate of unchanged renal elimination (Figure 1). The fraction of extrarenal elimination (i.e., One minus the fraction of parent substance recovered in urine) was used as the criterion (Figure S3). While shared substrates of MATE2K and OCT2 showed a tendency toward lower extrarenal elimination, this trend was not significantly different from the extrarenal elimination observed for substrates of other transporters. Therefore, being a substrate of MATE2K or OCT2 does not appear to be a dominant driving force of renal elimination via tubular secretion.

### 2.4 Chemoinformatic Characterization and Prediction of Substrate Specificity

Because we focus on identifying parameters differentiating substrate specificities of different organic cation transporters, our substance library mostly included low molecular weight substances positively charged at pH 7.4. As expected and as illustrated by principle component analysis comparing the 590 substances studied here with the 12,276 low molecular weight drugs from drugbank (https://go.drugbank.com/; Figure 8A,B), our sample does not cover the entire chemical space of the substances listed in drugbank. A comparison for each of the 11 parameters between all substances listed in drugbank and the substances studied here is given in supporting Figure S4.

**Figure 8:**
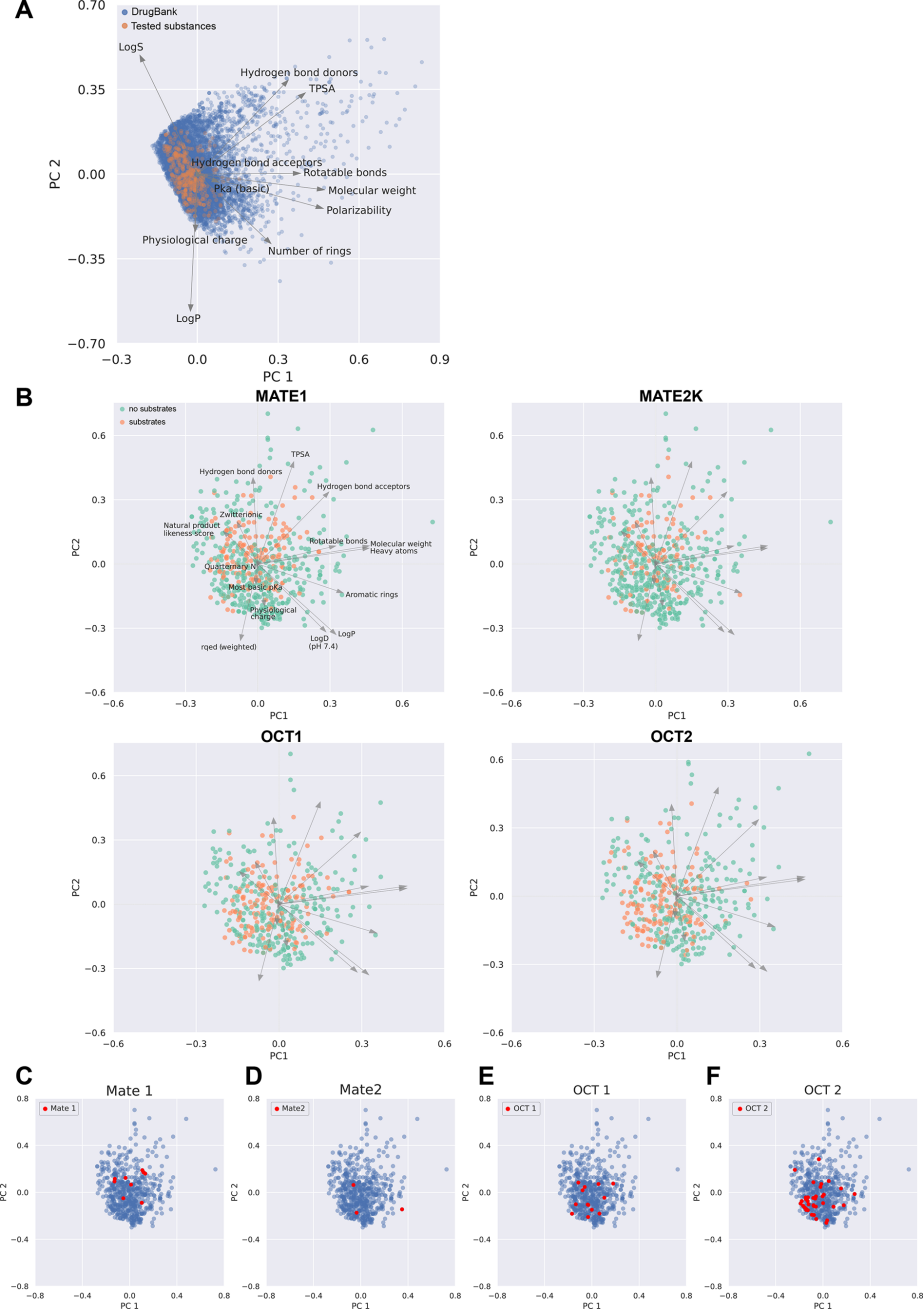
(A) Distribution of the first two principal components of all drugbank substances (blue) and the 590 substances studied here (orange) with the corresponding loading vectors of the 11 features. Below, (B) the loading vectors of a more extensive set of 15 features studied in our substances sample are shown. Green dots represent nonsubstrates, and orange dots represent substrates of the respective transporters. The red dots highlighted in (C–F) indicate those substances exclusively transported via the four SLCs.

The first two principal components of an extended set of 15 parameters used to study characterize the 590 substances studied here as substrates of the organic cation transporters are shown in Figure 8B. As seen, the majority of the MATE substrates are localized in the upper two quadrants, and the majority of OCT1 and −2 substrates were in the right two quadrants. However, although the centroids of the MATE1, OCT1, and OCT2 substrates in the PCA space did not appear identical, for none of the transporters a differentiation between substrates and nonsubstrates was possible. Assuming that only those substances transported by one of the four transporters might have particular properties, we also localized them in the plots of the first two principle components (Figure 8C–F). However, no particular feature was seen.

Linear discriminant analysis (data not shown) did also not differentiate between substrates and nonsubstrates. In summary, neither the single physicochemical parameters nor their linear combinations differentiated between the substrates of the four transporters. This is also illustrated by parameter-wise comparison of the substrates of the 4 transporters and the nonsubstrates (Supporting Figure S5).

Therefore, we used so-called ensemble machine learning methods to elucidate if one can predict substrates versus nonsubstrates of the four cation transporters. Results from the application of the Random Forest Classifier are presented here. The parameters and data used to train and evaluate the classifier are given in Supporting Table S4. As summarized in Table 3, 73, 69, 87, and 83% of the MATE1, MATE2, OCT1, and OCT2 substrates were correctly predicted, and the nonsubstrates were mostly even more precisely predicted.

**Table 3:** Summarized Performance of the Substrate Predictions by Machine Learning Using the Random Forest Classifier

		Experimentally identified	performance of the classifier^a^			
			precision	recall	*f*1-score	support
MATE1	no substrate	426	0.89	0.89	0.89	83
	substrate	**164**	**0.73**	**0.73**	**0.73**	**33**
	accuracy				0.84	116
MATE2K	no substrate	476	0.85	0.96	0.90	92
	substrate	**114**	**0.69**	**0.38**	**0.49**	**24**
	accuracy				0.84	116
OCT1	no substrate	347	0.79	0.95	0.86	55
	substrate	**170**	**0.87**	**0.59**	**0.70**	**34**
	accuracy				0.81	89
OCT2	no substrate	307	0.83	0.91	0.87	55
	substrate	**166**	**0.81**	**0.68**	**0.74**	**31**
	accuracy				0.83	86

a 80% of the experimental identified substrates and nonsubstrates were used for model training and model performance was analyzed with the remaining 20% (randomly selected by the classifier). As illustrated, predictive performance of the classifier increased with increasing number of substrates and nonsubstrates available for training of the classifier.

Figure 9 shows the relative feature importance as so-called shapely additive explanations (SHAP) of the classifier. These figures illustrate the effects of the parameters on the prediction of each single substance. As expected, the most basic p*K*_a_ and lipophilicity were among the best predictors for all four organic cation transporters. Total polar surface area (TPSA) is typically included in predictions of the penetration via the blood–brain barrier. However, here, a large TPSA appeared as a favorable property of MATE substrates. Generally, quaternary amines were good substrates of the transporters studied here, whereas zwitterionic compounds were not (Figure 9).

**Figure 9:**
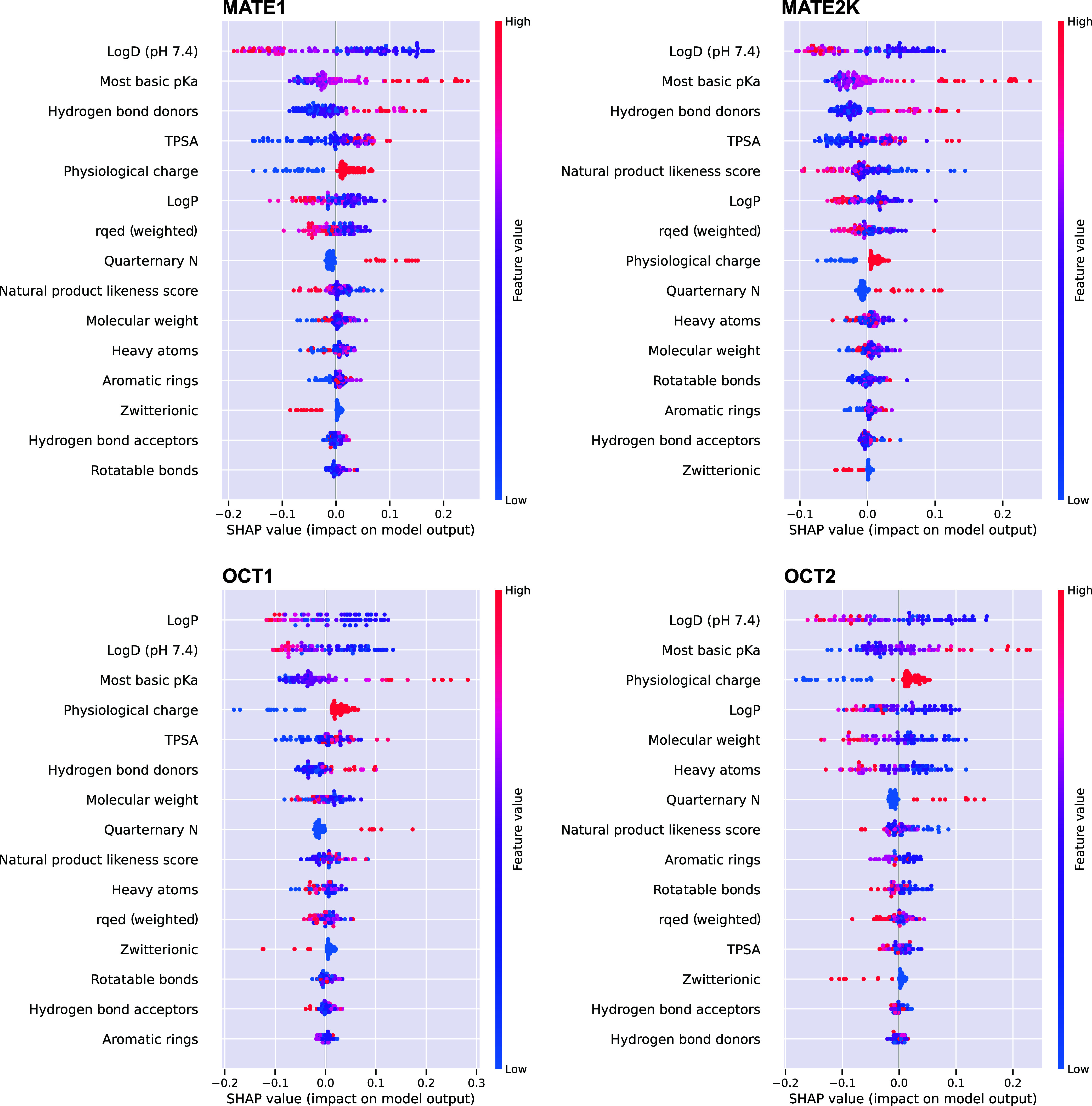
SHAP (Shapley Additive Explanations) summary plot showing the impact of individual parameters on the Random Forest Classifier’s predictions of substrates (> 0) vs nonsubstrates (< 0). Each point represents a feature’s contribution to a single prediction, with color indicating the feature value (red = high, blue = low). Parameters are ranked from top to bottom by decreasing the overall impact on the model’s output for each transporter. The characteristic determinants of the MATE and OCT substrates were generally similar, corresponding with the broad overlap in substrate specificity. As illustrated, hydrophilicity and the highest basic p*K*_a_ most strongly determined the substrates of the four transporters.

## 3 Discussion

In this study, we tested 590 mostly cationic substances as potential substrates of MATE1 and MATE2K. MATE1 transported 164 substrates above the cutoff ratio of 2, and from the same 590 substrates, MATE2K transported 114 substrates above the same cutoff ratio of 2. Among these, 81 and 48 substances were previously identified as substrates of MATE1 and MATE2K, respectively. Although some substances displayed relative specificity by one of the transporters, overall, there was a substantial overlap in the MATE1, MATE2K, OCT1, and OCT2 substrates (Figures 5, 6, and 7). This overlap can complicate our understanding of clinical studies on drug interactions at these transporters. Additionally, the effects of inherited genetic variation on clinical pharmacokinetics may be obscured by the involvement of multiple overlapping transporters. Many MATE1 and MATE2K substrates were from a few substance classes, such as anticholinergics, sympathomimetics, triptans, and biogenic amines. However, single physicochemical parameters or pharmacophores specifically predicting MATE substrates are not evident.

### 3.1 The Medical Relevance of MATE1 and MATE2K as Drug Carriers

Understanding which drugs are substrates for MATE1 and MATE2K would help optimize personalized drug therapy and avoid adverse drug interactions. Drug combinations can have severe effects for patients if one is a perpetrator and one a victim of interactions taking place at MATE1 or MATE2K. Therefore, the FDA recommends clarifying if investigational substances going into clinical drug development are substrates of MATE1 and MATE2K.^29^

Here, we focused on the identification of substrates, thus on possible victims of drug interactions at MATE transporters. However, several MATE1 inhibitors have already been identified; some of them are relatively specific, but others show a broad inhibition of other renal transporters.^30^ Especially for patients with impaired renal or hepatic function, it may become important to know which drugs are substrates of these transporters and understand the elimination pathways.

Victims of drug interactions taking place at the MATEs may primarily be expected among drugs eliminated via the kidneys. From the 590 substances tested here, 74 were eliminated to more than 70% unchanged via the kidneys, and 36 (49%) of these had a MATE1 transport ratio above 2. These include antivirals (acyclovir, zalcitabine, lamivudine), antidiabetics (sitagliptin, imeglimin), H1-antihistaminics (cimetidine, famotidine, ranitidine), anticholinergics (glycopyrrolate, trospium, tiotropium) and others like alizapride, amisulpride, sulpiride, amifampridine, sotalol, and edrophonium. However, whether or not these coeliminations via MATE transporters really result in major drug–drug interactions has not yet been confirmed in clinical studies. There are a few studies on interactions at the MATE transporters showing only minor mutual alterations in pharmacokinetics of the MATE substrates.

As shown here, up to 25% of low molecular weight cationic drugs are transported by MATE1, with substantial overlap involving MATE2K, OCT1, and OCT2. However, the transport mechanisms for the remaining 75% of similar substances remain unclear. Up to 50% of positively charged substances are transported via a genetically still incompletely characterized H^+^/OC antiporter. However, earlier identified substrates of the H+/OC antiporter^31–33^ were mostly no substrates of MATE1 and MATE2K. Transport via the H^+^/OC antiporter versus via MATE1 or MATE2K shows minimal overlap and is almost mutually exclusive. Thus, at present, the genes coding for proton-dependent cell uptake of about 75% of all organic cations are still unknown.

### 3.2 Therapeutic Groups with Single or Several MATE Substrate Members

This section highlights selected therapeutic groups whose members are frequently good substrates of MATE1, including cholinergic or anticholinergic drugs, H2 antihistaminics, β-blockers, herbal substances (notably isoquinolines), sympathomimetics, and triptans (Figures 2 and 7). Finally, a few groups will be discussed that, while not typically consisting of MATE substrates, may include individual substances that are MATE substrates.

Drug groups acting at the same targets (mainly receptors or enzymes) do not necessarily share the same membrane transporters or metabolizing enzymes. However, certain molecular features enabling receptor binding may overlap with those facilitating transporter binding. In this respect, in some instances this analysis may contribute to the elucidation of typical pharmacophores of MATE substrates. Adrenergic drugs serve as an illustration of that. Nearly 100 years ago, the 3 point fit pharmacophore was described for these adrenergic drugs with one hydrophobic site, one hydrophilic hydroxyl group, and on positively charged amino group.^34^ The aromatic group allowing hydrophobic binding via pi-stacking is found in most MATE substrates. Also, the polar hydroxyl group is a typical feature of many MATE and OCT substrates, and the third, the positively charged amino group required for binding to the adrenergic receptor, is in most MATE substrates. Similar comparisons could be made, for instance, for cholinergic receptors. However, these comparisons should also not be overstressed since there is no reason to believe that the exact steric conformation of the receptor′s binding sites is found in the polyspecific OCTs. The lack of a specific one-to-one correspondence between receptor binding and MATE substrates is best illustrated in Figures 1 and 2, which illustrate the predominance of MATE substrates in some therapeutic classes, but in each of the therapeutic drug classes, there were also nonsubstrates.

Most anticholinergics, which are good substrates of MATEs, are used as inhalants to treat bronchogenic asthma and COPD. Thus, for their action in the lung, the local expression of MATEs there would be of interest since exposure to these antiasthmatic drugs may be determined by cell uptake via these transporters.^35^ Additionally, hepatic and/or renal elimination via MATEs may determine the systemic toxicity of these anticholinergics.^36^ MATE1 is expressed in bronchiolar epithelial cells and immune cells in the lung,^37^ and, interestingly, smoking may downregulate MATE1 expression in the lung. Thus, the local effects of anticholinergic drugs in the lung may depend on their expression and possibly also on genetic variation in MATE1. Two function-changing variants were found in an Asian population,^38^ and there is at least one possibly functionally relevant promotor variant that has a reasonable population frequency in Europeans. Thus, more clinical studies on the medical effects of MATE genetic variation may be expected to emerge in future.

Four H2 antihistaminic drugs, but no H1 antihistaminic drugs, were identified as MATE substrates (Figures 1 and 2). H2 antihistaminergic drugs have already earlier been investigated as MATE substrates and inhibitors. For instance, ranitidine appeared to be a moderate inhibitor of the renal elimination of the MATE substrate trospium.^39^ Additionally, a study demonstrated that a dose of 800 mg cimetidine inhibited metformin elimination by approximately 43%, which was attributed to the inhibition of renal MATE1.^40^

Several β-blockers, including atenolol, acebutolol or celiprolol, are good substrates of MATE1 and are eliminated unchanged via the kidneys between 20 and 50%. However, many nowadays more frequently applied β-blockers, such as bisoprolol (Figure 2), metoprolol, or carvedilol, are either poor substrates or no substrates of MATE1 at all. These differences between the β-blockers as MATE substrates are only partially explained by hydrophilicity. For example, bisoprolol is relatively hydrophilic (predicted log *D* at pH 7.4 of 0.34, Table S2), and bisoprolol is renally eliminated to 50%. So, we expected that bisoprolol would be a MATE substrate. However, this is not the case. Thus, despite some predominance of adrenergic and antiadrenergic drugs among MATE substrates, the standard structural features of β blockers are not identical to the standard structural features of MATE substrates.

The involvement of MATEs in membrane transport of herbal substances may be one clue for the endogenous biological roles of the MATE transporters, which might serve to protect us from toxic herbal substances. Several benzylisoquinoline alkaloids, including berberine, coptisine, and palmatine, exhibit increased uptake ratios and are also effective substrates of OCT1 and OCT2. These compounds have been studied for their therapeutic potential, including antimicrobial and anti-inflammatory effects. One notable MATE1 substrate, *N*-methylserotonine (Figure 2) is synthesized in some plants but also in animals and bacteria and may have moderate hallucinogenic effects and MATEs mostly serving as efflux transporters in the living organism, may protect from such substances. Numerous isoquinoline derivatives are found in plants like berberis and used as herbal food and spice particularly in North African countries. Experimentally, they appear to have low toxicity but pharmacokinetic modulation by OCTs and MATEs is still not fully understood. Berberine, once proposed as a natural OCT1 probe, failed to reveal a clear OCT1 genotype-phenotype relationships despite initial expectations from studies in model cell lines.^41,42^ However, the role of MATE transporters has not yet been studied in that context but as shown by our data, might have to be considered in future clinical studies.

Several substances, such as coptisine and thiamine, showed concentration-dependent uptake in the control cell line indicating transport by constitutively expressed transporters in this cell model. Although there is not much expression data in HEK293 cells available, one would assume they need constitutive expressed organic cation transporters, such as amino acid transports, to import nutrients and amino acids or export degradation products.^43^

Sympathomimetic substances comprise the largest group in our screening. It is noteworthy that several sympathomimetics and endogenous compounds, such as biogenic amines, which play an important role in the adrenal gland, seem to be strong substrates for MATE1 but not MATE2K.^10,21,44^ Given that MATE1 is abundantly expressed in the adrenal gland, endobiotic or xenobiotic substrates of MATE1 may interfere with adrenal functions but we are not aware of specific data on that. Regulation of blood and brain concentrations of these sympathomimetic substances is of paramount importance. However, blood concentrations of many of these substances are very low compared with the intrinsic clearances (Table 1) which may argue against the relevance of MATE transporters for sympathomimetics. Nevertheless, protection against overload with some of these substances might be a biological role, as might be indicated by the analogous example of the highly potent drug fenoterol and the organic cation transporter OCT1. As shown in a clinical study, the related transporter OCT1 had a relevant role for blood concentrations despite of its also only moderate intrinsic clearances.^36^

Several classes of positively charged drugs contain either no substrates or only a few singular substrates of MATE1 or MATE2K. This is illustrated in Figure 2 on the example of psychostimulants and hallucinogens, but it is also illustrated in the example of local anesthetics from which only the experimental substance N-ethyl lidocaine has been identified as substrate of MATEs (Figure 7). Surprisingly enough, N-ethyl lidocaine was also identified as a substrate of TRPV1 before,^45^ and as shown here, it was a very good substrate of MATE1. The simple reason for the high uptake ratio of N-ethyl lidocaine is that, as a quaternary amine, it should never be able to diffuse through membranes by non-ionic diffusion. Thus, the baseline membrane passage is very low.

### 3.3 Correlating Uptake Ratios between MATEs and OCTs

Despite the limited sequence homology of less than 16% (Figure S6), there was a surprisingly high correlation in transport activities, particularly between MATE1 and OCT1 (Figure 5). This similarity in the substrate spectrum might indicate evolutionary convergence in the interest of joint handling of toxic (or biologically essential) endobiotics or xenobiotics. MATE1 and MATE2K had a high correlation (Spearman’s rank correlation coefficient rho of 0.46) with generally lower activities of MATE2K compared with MATE1. Also, correlation between the highly homologous OCT1 and OCT2 was very high (rho of 0.68). In all these correlations, we also looked at those substances not transported by one of the two transporters because specificity is an interesting question. Substances with no or minor correlation are highlighted in gray areas in Figure 5, and some substances only transported by one of the four transporters are listed in Table 2. Such substances might be used experimentally or in clinical studies as probe drugs for the respective transporter although that will have to be further confirmed in clinical studies. Therefore, we dichotomized the substrate properties, and the corresponding specificity or overlap is illustrated in the upset plot Figure 6, which also highlights the rather large group of substances transported by all four transporters. That point is also evident from the heatmap of therapeutic groups, which shows numerous substances transported by all four transporters (Figure 7). For instance, two methylated compounds, N-ethyl lidocaine and methylnaltrexone, were rather promiscuously transported by all the four cation transporters, but these “universally transported” cations also included drugs like neostigmine, ipratropium, amiloride, and frovatriptan (Table 2).

### 3.4 Relevance of Sequential Elimination via OCT Uptake and MATE Extrusion

While OCT1 and MATE1 are predominantly expressed in the human liver,^3,8^ OCT2 and MATE2K are predominantly expressed in the kidney.^10,46^ Based on the two-step transport (sometimes referred to as phase 0 and phase 3 of cellular drug disposition), a transported substance would have to be a substrate of both transporters. As illustrated in the upset plot (Figure 6), there were indeed several substances transported even exclusively by the pairs OCT1 and MATE1 or OCT2 and MATE2K, but substances transported via these two transporter pairs did not dominate the overall picture (Figure 6). Further, characterizing the overlap in substrate spectrum of OCTs and MATEs also enhances the understanding of drug–drug interactions. The elimination of drugs that are transported by both OCTs and MATEs can be influenced by inhibitors of both transporters, leading to changes in pharmacokinetics and possible toxicity.

While the substrate correlation of MATE1 with both OCT1 and OCT2 was high, there was only a low correlation between OCT1 or OCT2 with MATE2K mediated transport (Figure 5). This leads to the assumption that MATE1 and OCT1 could indeed work in a two-step transport process for elimination of drugs. However, since the activity of CYP enzymes was not taken into account during the testing, it is unclear how many substances that are transported into the liver by OCT1 are transported without metabolic alterations out of the liver by MATE1. In addition, the functional role of MATE1 expression in hepatocytes still needs further clarification. The substrate overlap between OCT2 and MATE2K substrate was very low (rs of −0.1) and only one substrates specific for only OCT2 and MATE2K could be identified (rasagiline). OCT2 shows a high abundance of specific substrates (38) that were not transported by any of the other three transporters.

### 3.5 Stereoselectivity

The tested racemic drugs and single enantiomers did not display high stereoselectivity of either MATE1 or MATE2K. Although slight differences in *K*_M_ value could be observed for zolmitriptan (Figure 4), most other racemic substances showed little to no enantio-specific uptake. In contrast, OCT2 and OCT3 have been shown to be stereoselective for several substrates, but the effects were very different, even between the related transporters OCT1, −2, and −3.^47^ It may be suggested that the MATE substrate-binding center is larger and less specific.

### 3.6 Chemoinformatic Predictability of Substrates versus Nonsubstrates

As shown by the mutual correlations of the transport activities of the four transporters (Figure 5) and Figures 6 and 7, some substrates were transported exclusively by a single transporter—most notably, OCT2, which had nearly 40 specific substrates. However, beyond this, there was substantial overlap in substrate specificity across the transporters.

None of the standard physicochemical parameters allowed to distinguish MATE1, MATE2-K, OCT1, and OCT2 substrates from one another. As illustrated in Suppo̅rting Figure S5, not even a trend indicative of transporter specific differences was seen. However, the figure shows some differences between substrates and nonsubstrates. For instance, on average nonsubstrates had a lower basic p*K*_a_ and were more lipophilic. In addition, zwitterions are mostly not good substrates of the four organic cation transporters.

We included parameters commonly used to describe the biological interactions of organic molecules. In addition, our machine learning model incorporated two composite descriptors: the natural product likeliness score and the RQED.^48,49^ While the inclusion of composite descriptors might appear redundant, they may enhance predictive performance due to differences in how each parameter encodes molecular characteristics.

As a next step, we analyzed the data with principle component analysis to focus on those physicochemical parameters of the tested substances which explained most of the variance. Although this illustrated possible differences (Figure 8), it did not allow differentiating between substrates and nonsubstrates.

Therefore, finally, we used machine learning approaches to find out, if prediction of substrates based on molecular properties is possible. The prediction performance was lowest for MATE2K. However, as illustrated in Table 3, that simply reflects the lowest number of substrates identified for this transporter compared to the other three ones. Correspondingly, for all transporters except for OCT1 an even prediction of nonsubstrates was achieved corresponding to the higher number of nonsubstrates available for model training. Illustrative are the so-called SHAP plots showing the contribution of each parameter to the prediction of a compound being a substrate or nonsubstrate. (Figure 9). As seen, the most relevant parameters were log *D*, p*K*_a_ (basic), TPSA, and physiological charge. Those parameters are among the most expected parameters. The surprise however is that they were not predictive when analyzed monofactorially. Trying to understand this one may argue that substance-specific combinations of the parameters determine if a substance is a substrate or a nonsubstrate of each transporter. From a structural view, the substrate-specific combinations of physicochemical properties may correspond to different binding sites at the substrate translocation channel. Such different binding sites have been deduced from homology models,^50,51^ but are also seen in cryogenic electron microscopy.^52,53^ With the large number of substrates identified here there may be even more modes of substrate binding and translocation.

Although the predictive power of the machine learning algorithm applied here is surprisingly good, there are limitations. These machine learning approaches (the random forest classifier and the similar histogram gradient boosting classifier) do not result in a molecular understanding or in a structural scheme of a typical substrate on nonsubstrate. Structural fragment-based or substructure-based fingerprints might appear helpful but with that the number of parameters would nearly exceed the number of substrates identified here. In addition, it is unlikely that the polyspecific transporters studied here have one characteristic fingerprint. Thus, machine learning predicts substrates of OCT1 quite well and the parameters predicting were plausible but they do not result in a characteristic molecular scheme of OCT1 substrates and does not help to understand which part of the substrate molecule interacts with which amino acid in the transporters. A second limitation is that the performance of these classifiers strongly depends on the data input for classifier training (Table 3). Thus, these classifiers will not be helpful if only low numbers of experimentally studied substances exist. A third limitation is that the classifiers, in general, will only identify substrates within the chemical space of the training set. The latter point was tested with more than 10,000 substrates from drugbank. More than 270 new substrates of the four transporters were identified with a probability of at least 98% (Table S7). These predicted substances were generally similar concerning chemical features and many were from the same substances classes as found here (Figures 1 and 2). However, we have not verified these predictions by transport measurements. As documented in Table S7, for the latter predictions the histogram gradient boosting and random forest classifiers were applied, mainly yielding corresponding results. In the future, we may also utilise machine learning algorithms, such as the random forest classifier or the histogram gradient boosting classifier, to screen for substrates of the four transporters in other substance databases, such as human metabolomics databases, based on the training data sets provided in the supplement to the present publication.

Understanding and predicting which substances serve as substrates for specific enzymes or transporters remains a long-standing, forward-looking goal of the research presented here. Notable progress has been made in elucidating substrate binding and transport mechanisms of OCT1 and OCT2, as demonstrated by recent structural studies even capturing intermediate steps of the transport cycle.^52,53^ However, these structural insights into the substrate-transporter interactions have so far relied on substrates previously identified through experimental transport kinetics, as done here for the 590 tested compounds. Therefore, despite advancements in structural biology, experimental validation remains essential for confirming substrate identity. And, as illustrated, our view on the substrate spectra may be enhanced by machine learning algorithms.

As illustrated in Figure S7, and as described earlier for most substrates of OCT1, OCT2, and OCT3,^4^ almost all substrates of MATE1 and MATE2K have at least one aromatic ring. Beyond that rule of at least one aromatic ring the MATE substrates greatly differ in complexity. Another rule is that all substrates have at least one polar group (often hydroxyl groups). However, beyond that no common structural features are immediately leaping to the eye. Interesting exceptions to the rule of at least one aromatic ring are the ethylenediamine derivative ethambutol (MATE1 ratio of 14) and 1,4-diguanidinobutane (arcaine) with an exceptionally high MATE1 uptake ratio of 40.

Supporting Figure S5 compares other basic molecular features of MATE substrates with those of OCT substrates and with those of nonsubstrates of all the four transporters. Substances with molecular weights below 100 or above 600 appear to be rarely MATE substrates. However, a broader range of substrates with a molecular weight below 100 and above 600 Da has yet to be tested as possible MATE substrates. Highly lipophilic substances with log *D* above 2.0 are less likely to be transported by MATEs but some very hydrophilic substances are transported. This could be observed for substances that were no substrates of any of the tested transporters that shared the common factor of high lipophilicity. A small topological polar surface is a good criterion for penetration through the blood brain barrier but interestingly several good substrates of MATE1 or MATE2K had a slightly higher TPSA (Figure S5).

### 3.7 Expanding Horizons: Exploring Uncharted Substrate Classes and Chemical Space for Organic Cation Transporters

Here, we mainly studied positively charged organic substances with molecular weight between 86 and 930. So, we cannot answer if MATE1 or MATE2K have smaller substrates. There is indirect evidence that another organic cation transporter can transport ammonia.^54^ From the currently available protein structures of OCT1 and 2,^52,53^ it is unlikely that this family of solute carriers can transport molecules much larger than 1000 Da. Concerning charge, we only studied 20 organic anions, and only three (estrone-3-sulfate, bumetanide and ochratoxin A) were substrates of MATE1, and riboflavin, in addition, was a substrate of MATE2K. Earlier studies showed that prostaglandins E1 and F2alpha are substrates of OCT1 but only with a most likely physiologically irrelevant low affinity in the millimolar range.^6^ And as shown here, those prostaglandins are no substrates of the MATEs. Nevertheless, further expansion in testing organic anions might be promising, and elucidating the mechanisms of how they are transported might add to our understanding of the transporter function. One might also systematically test peptides and peptidomimetics as substrates of these organic cation transporters. Here, we tested thyrotropin-releasing hormone and captopril, which were no substrates of the four transporters. However, comprehensive testing of peptides as MATE substrates may be promising and even give a clue to the endogenous roles of these transporters. Finally, looking even broader, organic cation transporters may be studied as transporters of inorganic cations, and the “classical” transporters of inorganic cations may be tested as transporters of organic cations.

## 4 Conclusions

MATE1 exhibits a broad substrate spectrum encompassing endogenous compounds, drugs across various therapeutic classes, and several herbal substances. There was an astonishing substrate overlap between MATE1 and OCT1 substrates despite less than 16% sequence homology. Clinically, this overlap might complicate understanding of drug–drug interactions or the effects of inherited genetic variation in OCT1. This substantial number and diversity of substrates–varying in size, structure and charge–underscore the importance of studying MATE1 and MATE2K in pharmacokinetics and its potential implications in drug interactions and therapeutic efficacy. Altogether, our study provides extensive substrates and nonsubstrates of MATE1, MATE2K, OCT1, and OCT2 and as shown here, this database can be used to train machine learning models to predict substrates of these transporters.

## 5 Experimental Section

### 5.1 Test Compounds

The compounds used in the in vitro experiments were obtained from the companies listed in Table S5. According to the suppliers, all compounds had purities of at least 95%, as verified by high-performance liquid chromatography (HPLC) analysis. We have reanalyzed representative compounds regarding purity by additional HPLC (Figure S8).

### 5.2 Cell Lines

Uptake experiments were conducted in HEK293 cells stably overexpressing MATE1, MATE2K, OCT1, and OCT2 as well as in the control cell line overexpressing the empty pcDNA5/FRT vector (Thermo Fisher Scientific, Darmstadt, Germany). The cell lines were generated using the recombinant site-specific Flp-In^TM^ system as previously described.^25,55,56^ All cell lines used in the transport experiments were cultured at 37 °C and 5% CO_2_ with Dulbecco’s Modified Eagle’s Medium (DMEM) supplemented with 10% (v/v) FBS and the antibiotics penicillin (100 U/mL) and streptomycin (100 μg/mL).

### 5.3 In Vitro Transport Experiments

All transport measurements in this study evaluated the influx transport into HEK293 cells growing adherently on the cell culture plates. Under standard conditions, where the extracellular proton concentration exceeds the intracellular proton concentration, both MATE transporters usually function as efflux transporters. To modify that efflux transport direction into an influx transport for practical reasons, we changed the proton gradient at the outer cell membrane using pretreatment with ammonium chloride. That treatment initially results in intracellular alkalinization but later, due to intracellular compensatory mechanisms, an intracellular acidification is achieved.^57^

For in vitro uptake experiments, 12-well plates were precoated with poly-d-lysine and seeded with 600,000 cells per well, 48 h prior to the experiment. Prior to substrate incubation, the cells were washed with 37 °C HBSS+ (Hank’s balanced salt solution supplemented with 10 mM HEPES; Thermo Fisher Scientific, Darmstadt, Germany; adjusted to pH 7.4) and then incubated with 30 mM ammonium chloride (NH_4_Cl) in HBSS+ for 30 min at 37 °C. After removing the ammonium chloride containing HBSS+, the cells were exposed to a defined substrate concentration (diluted in HBSS+ without ammonium chloride) for exactly 1 min. Uptake was stopped with ice-cold (4 °C) HBSS+ followed by two washing steps. Finally, the cells were lysed with 80% (v/v) acetonitrile/water containing an appropriate internal standard for mass spectrometry analysis for 15 min (Table S6). To account for interday variations in seeded cell numbers and cell growth in the last 2 days, two extra wells of each cell line were lysed with RIPA buffer and used for total protein quantification with a bicinchinonic acid assay normalized to a standard curve of bovine serum albumin.^58^

### 5.4 Concentration Analysis via HPLC-MS/MS

Intracellular drug concentrations were quantified using high-performance liquid chromatography coupled with tandem mass spectrometry (HPLC-MS/MS) analysis. The Shimadzu Nexera HPLC system consisted of a CBM-20A controller, a LC-30AD pump, a CTO-20AC column oven and an SIL-30AC autosampler (Shimadzu, Kyoto, Japan). Compound separation was achieved using a Brownlee SPP RP-Amide column (4.6 mm × 100 mm inner dimensions, particle size of 2.7 μm) with a Phenomenex C-18 guard column. The mobile phase for reversed-phase chromatography consisted of an 6:1 (v/v) acetonitrile/methanol organic additive in a 0.1% (v/v) formic acid aqueous base. The individual proportion of the organic additive differed between 3 and 50% and is listed for each substance in Table S5. Detection and quantification were performed with an API 4000 tandem mass spectrometer (AB SCIEX, Darmstadt, Germany) and the corresponding Analyst software (AB SCIEX, version 1.6.2). Detailed parameters for mass spectrometric detection are also provided in Table S6.

### 5.5 Calculations and Statistics

The uptake data at a single concentration of 2.5 μM are presented as means with the standard error of the mean in the Supporting Information and in the main text. This data shows the fold increase of uptake in transporter-overexpressing cell compared to empty-vector transfected control cells. Statistical significance was analyzed using a one-sample Student’s *t* test, comparing the fold-change to 1. Significance levels are indicated as follows: * *p* < 0.05, ** *p* < 0.01, *** *p* < 0.001. All statistical analyses were performed using the GraphPad Prism software (version 5.01 for Windows, La Jolla, CA).

Concentration-dependent uptake experiments were conducted using five to eight different concentrations on both MATE1- and MATE2K-overexpressing cells and empty-vector control cells. The net uptake of the respective transporter was obtained by subtracting the uptake in empty-vector cells from MATE1- or MATE2K-overexpressing cells and plotted against the substrate concentration. The net uptake was analyzed with nonlinear regression based on the Michaelis–Menten equation *v* = *V*_max_ × [*S*]/(*K*_M_ + [*S*]). Here, *V*_max_ represents the maximum transport velocity, [S] the substrate concentration, and *K*_M_ the substrate concentration needed to achieve half of *V*_max_. The resulting ratio of *V*_max_ over *K*_M_ is the intrinsic clearance (Cl_int_) that is used as a parameter to compare the transport efficacy of substances.

The uptake data of OCT1 and OCT2 used to analyze possible correlations and interactions with MATE1 and MATE2K (Figures 5 to 8) include new uptake measurements and published data from Hendricks et al.,^19^ Gebauer et al.^4^ and Redeker at al..^6^

The sequence homology analysis was performed with Geneious Prime 2023.0.1 using the MUSCLE 5.1 algorithm (https://www.geneious.com).

Basic chemical descriptors—including most basic p*K*_a_, log *D* at pH 7.4, and net charge—were obtained using MarvinSketch and the Instant JChem package from ChemAxon (version 21.2.0, Budapest, Hungary). Additional descriptors were retrieved from the ChEMBL database in March 2025.^59^ DrugBank data were generously provided by the DrugBank team in March 2025.^60^ The 590 compounds analyzed in this study were compared against 12,276 substances listed in DrugBank (all substances listed excluding peptide or protein therapeutics and other macromolecules exceeding 2000 Da).

To reduce the dimensionality of the data set while preserving the majority of its variance, Principal Component Analysis (PCA) was employed. For PCA, all chemical descriptors were standardized using the *StandardScaler* class from the *sklearn*.*preprocessing* module, ensuring a mean of zero and a unit variance for all features. Following normalization, PCA was performed using the *PCA* class from the *sklearn*.*decomposition* module in *scikit-learn* (version 1.4.2).

For visualization purposes, the first two principal components were used to illustrate the distribution of the 590 investigated components in relation to the representative subset of DrugBank, as well as the distribution of the substances identified as substrates of the respective transporters. These two components capture the greatest variance in the data set and provide a meaningful two-dimensional projection. The percentage of variance explained by the components is shown in Figure S9.

To evaluate to what extent the physicochemical features are suitable for predicting whether a compound is a substrate of a specific transporter, the Random Forest Classifier (with 100 estimators) from the *sklearn*.*ensemble* module was applied. The data set was split into training and evaluation sets, using 80% of the data for training the model and 20% for assessing its predictive performance.

To assess the quality of the classification, the following standard evaluation metrics were used: Precision, which measures how many of the compounds predicted to be substrates are actually true substrates—reflecting the accuracy of positive predictions; recall, which, in contrast, measures how many of the actual substrates were correctly identified by the model—reflecting its ability to detect positive cases; and the *F*1-score, which provides a single value balancing both precision and recall by calculating their harmonic mean. Finally, support refers to the number of actual occurrences of each class (e.g., substrate or nonsubstrate) in the evaluation data set.

To enhance the interpretability of the Random Forest Classifier, SHAP (SHapley Additive exPlanations; version 0.47.0) was employed to quantify the contribution of individual features to the model’s predictions. A *shap*.*summary_plot* was generated to visualize the overall importance and distribution of feature effects across all samples. All computations were performed using Python version 3.9.19.

## Supplementary Material





## Data Availability

All data supporting the conclusions of this article are presented in the main manuscript and in the Supporting Information.

## Notes

MATE1 and MATE2K substrates with highest uptake ratios (Figure S1); Concentration-dependent uptake via MATE1 and MATE2K (Figure S2); Comparison of extrarenal elimination between substrates of the four transporters and shared substrates of two transporters (Figure S3); Comparison of basic chemical parameters between 590 tested substances and substances listed in DrugBank (Figure S4); Comparison of basic chemical parameters between all substrates of MATE1, MATE2K, OCT1, and OCT2 (Figure S5); Sequence alignment of the four SLCs of interest (Figure S6); Chemical structures of MATE1 and MATE2K substrates in comparison to non-substrates (Figure S7); Testing of selected substrates for purity using HPLC and UV detection (Figure S8); Screening plot for principle component analysis (Figure S9); Known substrates of MATE1 and MATE2K from literature (Table S1); Screening of 590 substances as possible substrates of MATE1 and MATE2K (Table S2); Uptake ratio of racemates and single enantiomers by MATE1 and MATE2K (Table S3); Biochemical parameters and substrate data of 590 tested substances (Table S4); All 590 substances tested in the present study (Table S5); HPLC-MS/MS analysis parameters of the substances included in the study (Table S6); Substrates of the four transporters predicted from 10,010 low molecular weight substances in drugbank (Table S7) (PDF)
Compounds and their SMILE (CSV)

## Abbreviations Used

DMEM: Dulbecco’s Modified Eagles Medium
D-PBS, HEK293: human embryonic kidney 293 cell line
HBSS: Hank’s balanced salt solution
HPLC-MS/MS: liquid chromatography coupled to tandem mass spectrometry
MATE: multidrug and toxin extrusion protein
MDCK: Madin-Darby canine kidney cell line
MPP^+^: 1-methyl-4-phenylpyridinium
OCT: organic cation transporter; PCA (principal component analysis)
SEM: standard error of the mean; SHAP (shapely additive explanations))
TEA: tetraethylammonium
