# Supplementary material for: Substrate Specificity of the Organic Cation Transporters MATE1 and MATE2K and Functional Overlap with OCT1 and OCT2
Source: J Med Chem. 2025 Jun 13;68(12):12473–92. doi: 10.1021/acs.jmedchem.5c00056 (PMC12207590; doi:10.1021/acs.jmedchem.5c00056)
Supplement: Supplementary file 1 [file jm5c00056_si_001.pdf]

Supporting information

Substrate specificity of the organic cation transporters MATE1 and MATE2K and functional overlap with OCT1 and OCT2

Kyra-Elisa Maria Redeker[1,*], Nicolai Kirsch[1], Susann Boretius[2], Mladen Tzvetkov[3], and Jürgen Brockmöller[1]

[1] Institute of Clinical Pharmacology, University Medical Center Göttingen, Georg-August-University, D-37075 Göttingen, Germany

[2] Functional Imaging Laboratory, German Primate Center, Leibniz Institute for Primate Research, D-37077 Göttingen, Germany

[3] Department of General Pharmacology, Institute of Pharmacology, Centre of Drug Absorption and Transport (C-DAT), University Medical Centre Greifswald, D-17487 Greifswald, Germany

*kyra-elisamaria.redeker@med.uni-goettingen.de

Table of Content **Page**

Figure S1: MATE1 and MATE2K substrates with highest uptake ratios S3

Figure S2: Concentration-dependent uptake via MATE1 and MATE2K S5

Figure S3: Comparison of extrarenal elimination between substrates of the four transporters and shared substrates of two transporters S9

Figure S4: Comparison of basic chemical parameters between 590 tested substances and substances listed in DrugBank. S10

Figure S5: Comparison of basic chemical parameters between all substrates of MATE1, MATE2K, OCT1, and OCT2 ..... S11

Figure S6: Sequence alignment of the four SLCs of interest S12

Figure S7: Chemical structures of MATE1 and MATE2K substrates in comparison to non-substrates. S13

Figure S8: Testing of selected substrates for purity using HPLC and UV detection S14

Figure S9: Scree plot for principle component analysis S16

Table S1: Known substrates of MATE1 and MATE2K from literature S17

Table S2: Screening of 590 substances as possible substrates of MATE1 and MATE2K.. S23

Table S3: Uptake ratio of racemates and single enantiomers by MATE1 and MATE2K S67

Table S4: Biochemical parameters and substrate data of 590 tested substances.............. S69

Table S5: All 590 substances tested in the present study... S113

Table S6: HPLC-MS/MS analysis parameters of the substances included in the study ... S124

Table S7: Substrates of the four transporters predicted from 10,010 low molecular weight substances in drugbank.. S148

Figure S1: MATE1 and MATE2K substrates with highest uptake ratios

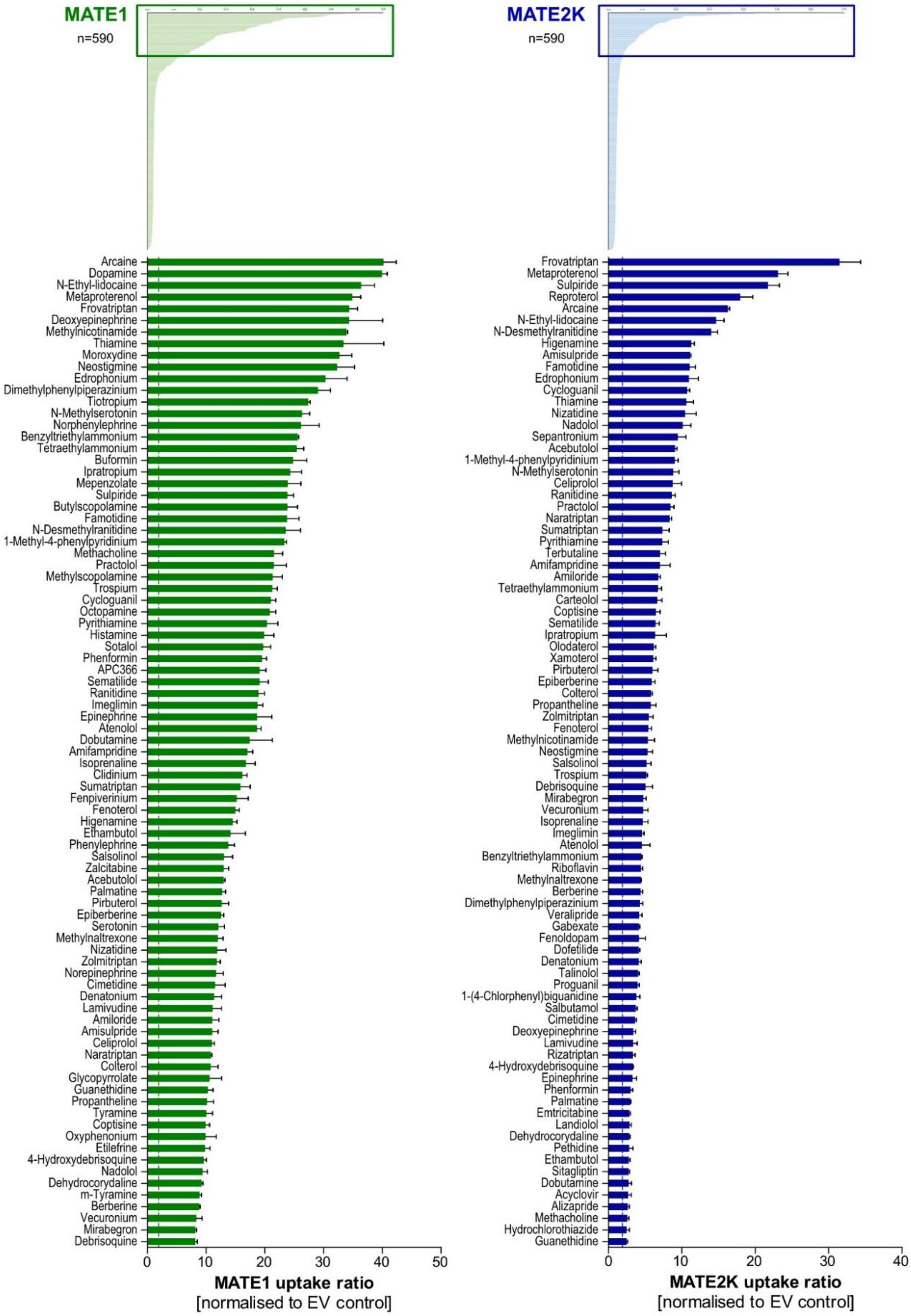

<u>Figure S1:</u> MATE1 and MATE2K substrates with highest uptake ratios.

Uptake ratios of the best 85 substrates of the tested 590 substances are displayed. HEK293 cells overexpressing MATE1 (green), MATE2K (blue) or the empty vector (EV)-transfected control cells were

incubated with 2.5 µM substance for 1 min, and the intracellular substrate concentration was quantified by HPLC-MS/MS analysis. Uptake is expressed as fold-increase in transporter-overexpressing cells over EV-transfected control and presented as mean ± SEM of three independent experiments. The dashed horizontal lines at 2.0 indicate the transport ratio above which the transport is generally considered medically relevant.

Figure S2: Concentration-dependent uptake via MATE1 and MATE2K

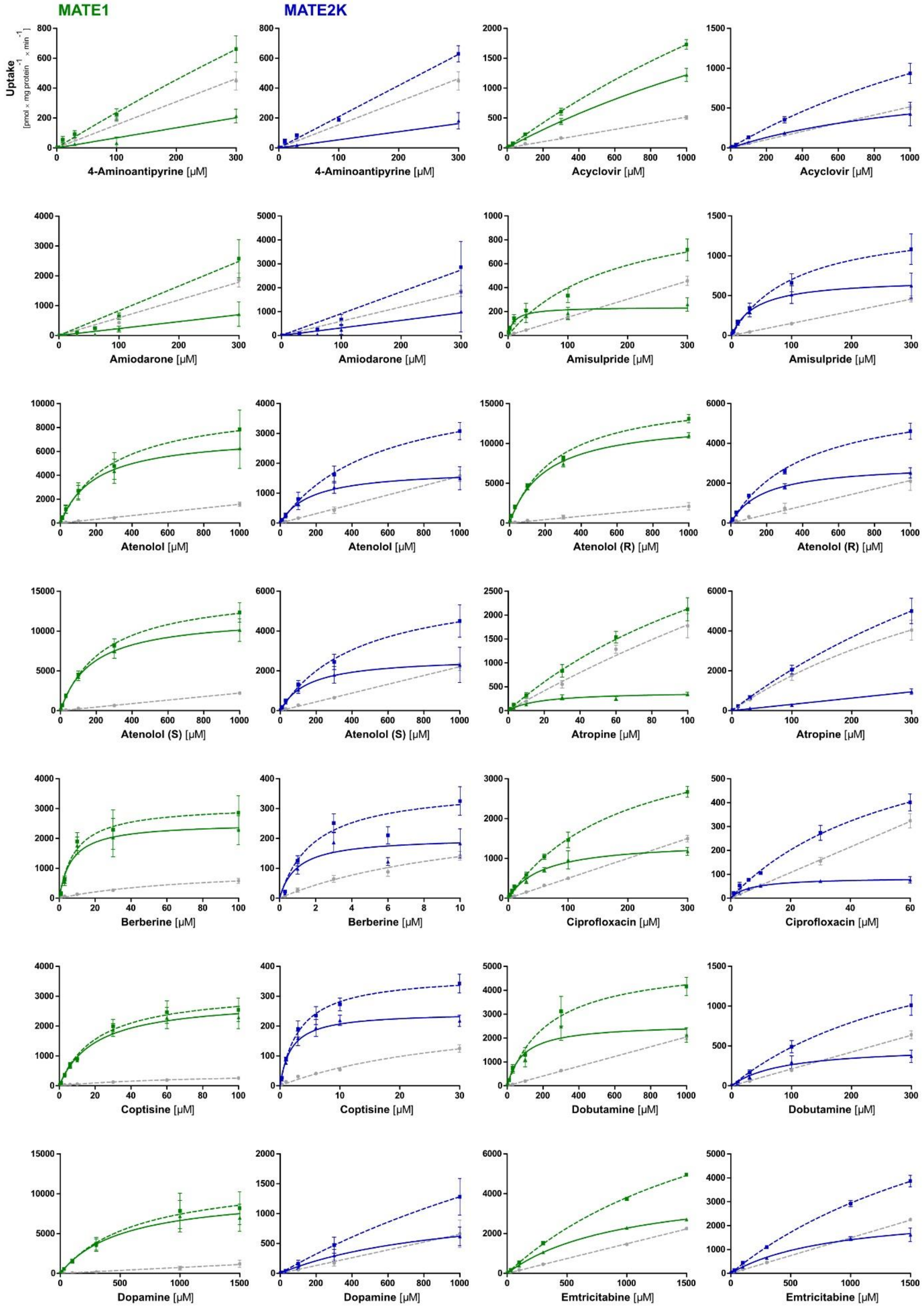

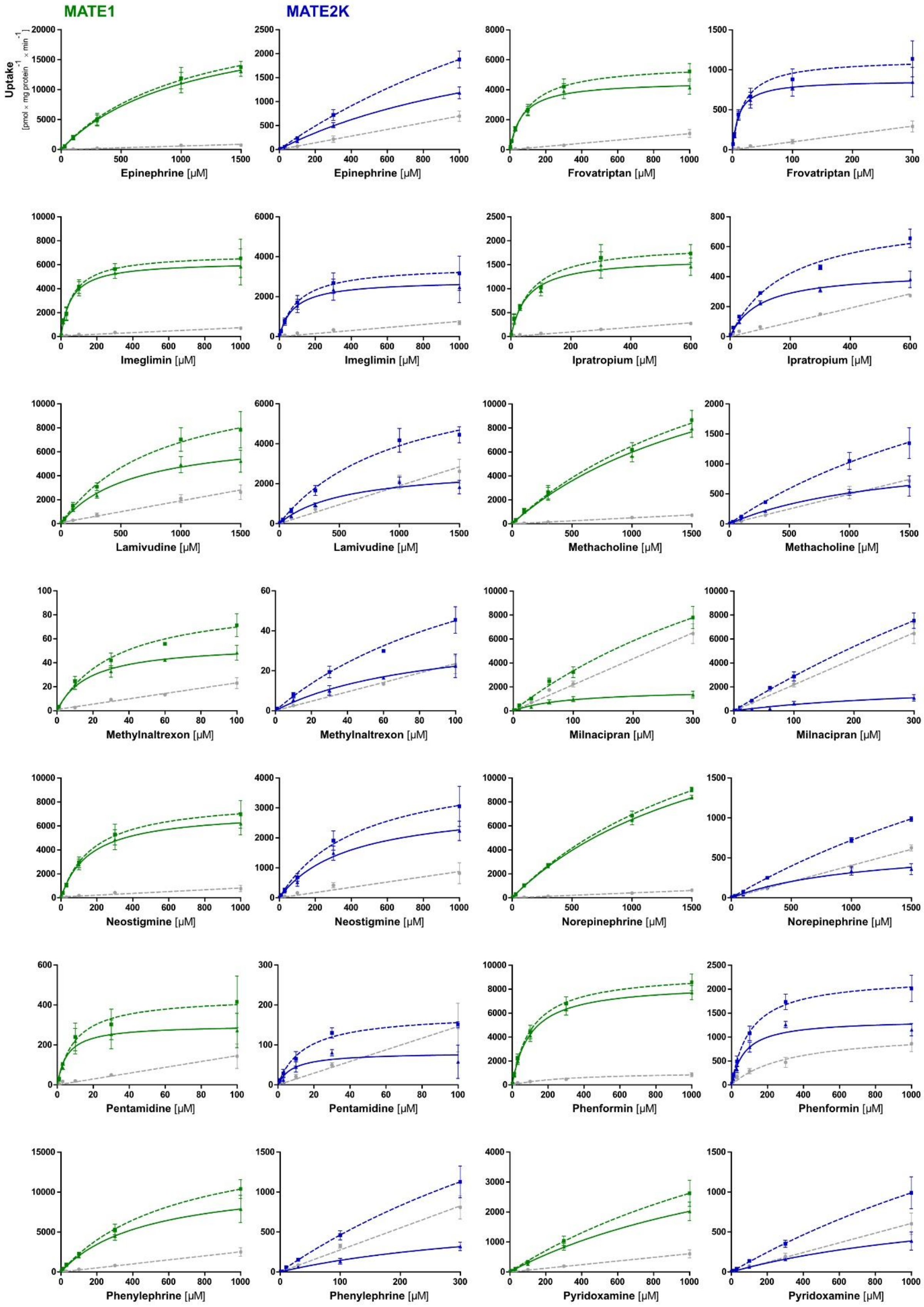
MATE1
MATE2K
Uptake [pmol × mg protein-1 × min-1]
Epinephrine [µM]
Epinephrine [µM]
Frovatriptan [µM]
Frovatriptan [µM]
Imeglimin [µM]
Imeglimin [µM]
Ipratropium [µM]
Ipratropium [µM]
Lamivudine [µM]
Lamivudine [µM]
Methacholine [µM]
Methacholine [µM]
Methylnaltrexon [µM]
Methylnaltrexon [µM]
Milnacipran [µM]
Milnacipran [µM]
Neostigmine [µM]
Neostigmine [µM]
Norepinephrine [µM]
Norepinephrine [µM]
Pentamidine [µM]
Pentamidine [µM]
Phenformin [µM]
Phenformin [µM]
Phenylephrine [µM]
Phenylephrine [µM]
Pyridoxamine [µM]
Pyridoxamine [µM]

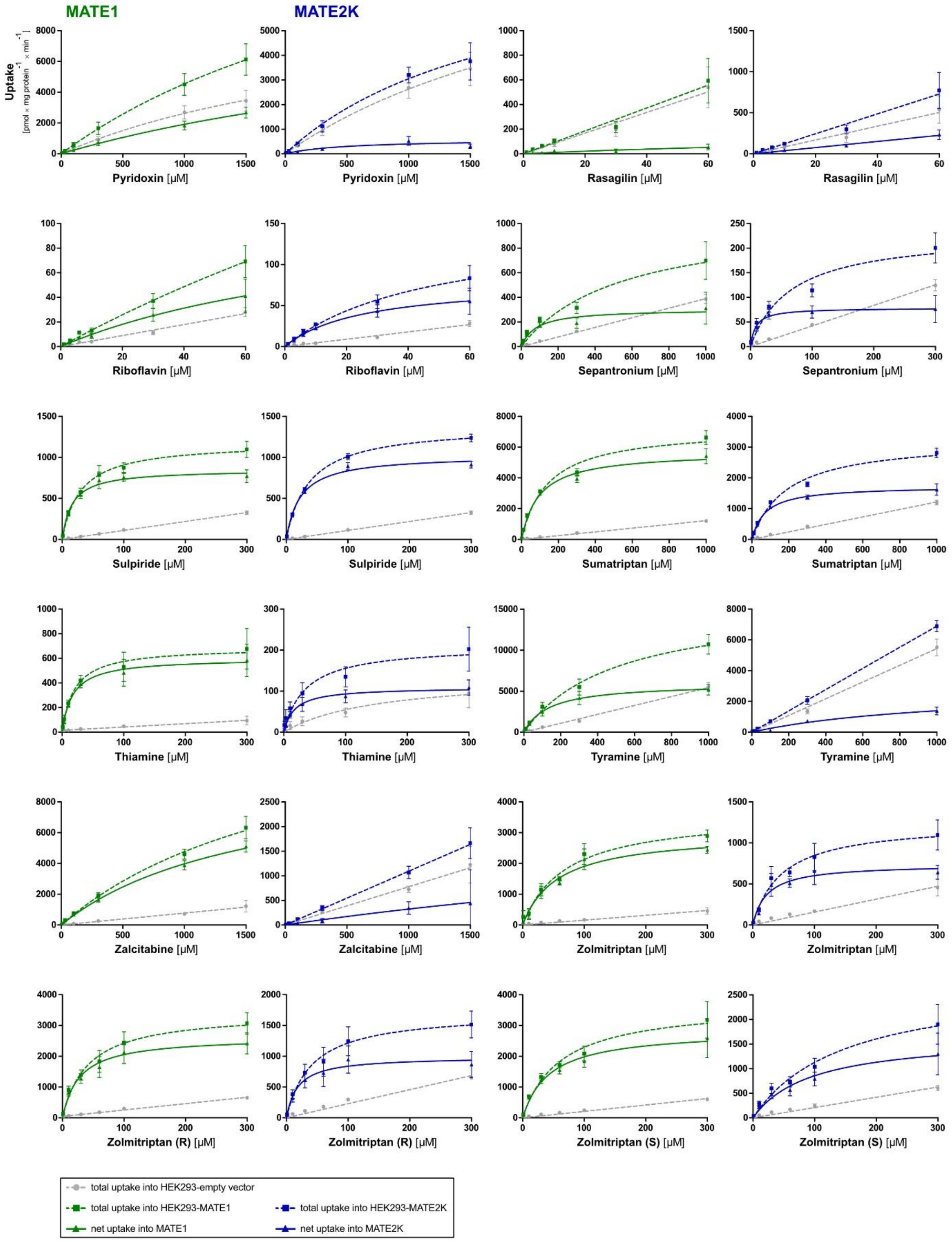

Figure S2: Concentration-dependent uptake via MATE1 and MATE2K.

Concentration-dependent uptake of 40 substances by MATE1 and MATE2K were performed in HEK293 cells overexpressing the respective transporter or the empty pcDNA5 vector with 1 min incubation of the respective substance at various concentrations, and analysed via HPLC-MS/MS. The dashed green or blue curves represent total uptake by cells overexpressing MATE1 or MATE2K, while the grey lines indicate total uptake by empty vector-transfected cells. The solid green or blue curves indicate the net

uptake by the respective transporter. Data are displayed as mean ± SEM of three independent experiments.

Figure S3: Comparison of extrarenal elimination between substrates of the four transporters and shared substrates of two transporters

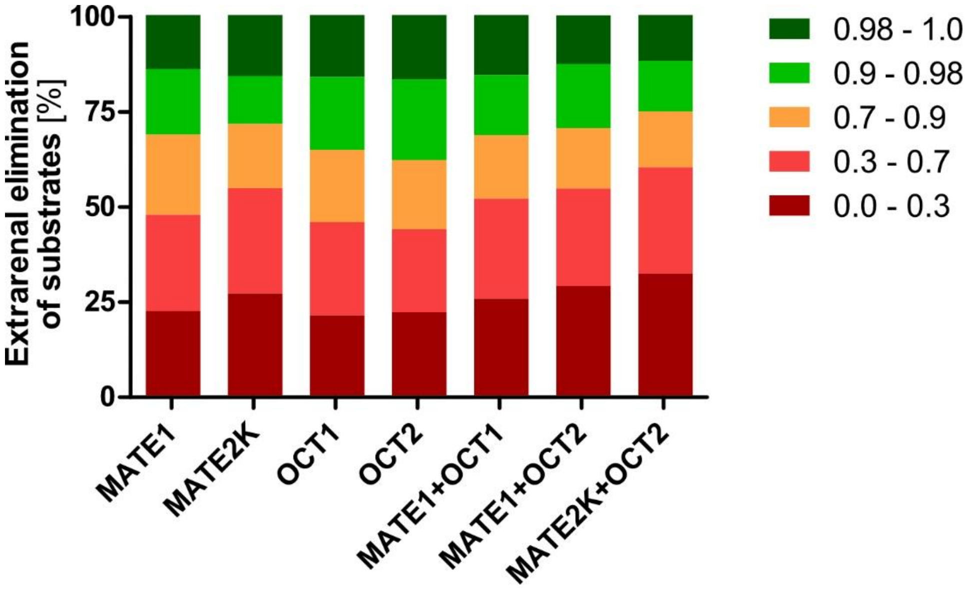

Figure S3: Comparison of extrarenal elimination between substrates of the four tested transporters and shared substrates of two transporters. A high extrarenal elimination above 0.98 indicates that almost nothing is eliminated unchanged via the kidneys. In contrast, an extrarenal elimination below 0.3 indicated that most or almost all of the substance is eliminated unchanged via the kidneys.

Figure S4: Comparison of basic chemical parameters between 590 tested substances and substances listed in DrugBank.

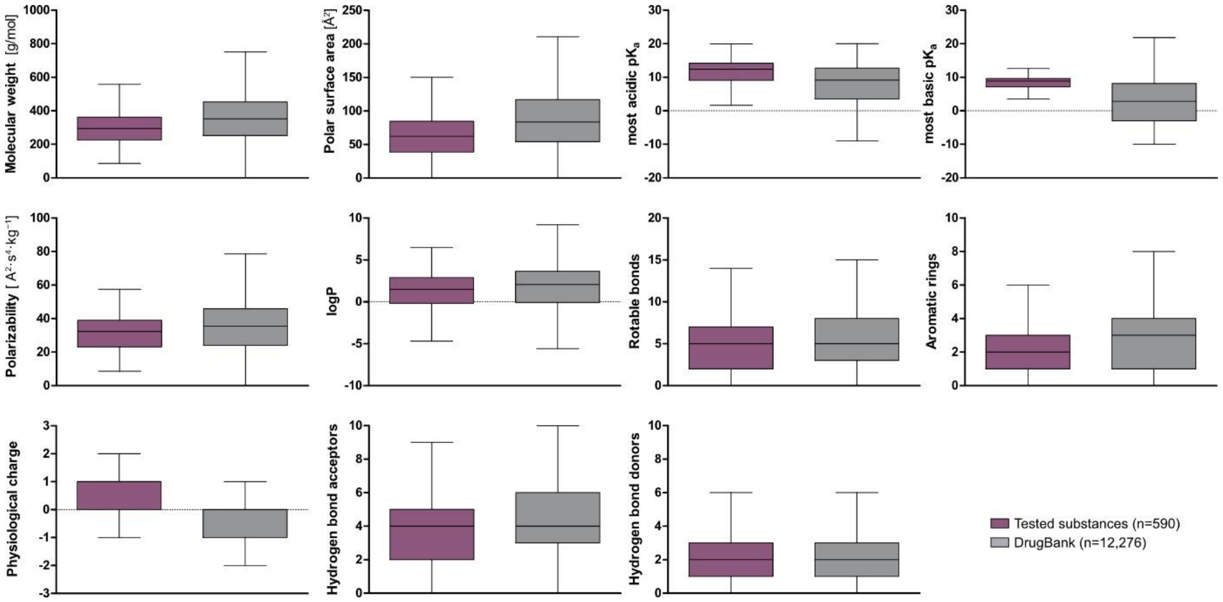

Figure S4: Comparison of eleven basic chemical parameters between all 590 tested substances and the substances listed in DrugBank excluding only those substances listed in drugbank, which have a molecular weight above 2000 Da (mostly antibodies and other therapeutics proteins). For this comparison only the parameters listed in drugbank were used.

Figure S5: Comparison of basic chemical parameters between all substrates of MATE1, MATE2K, OCT1, and OCT2

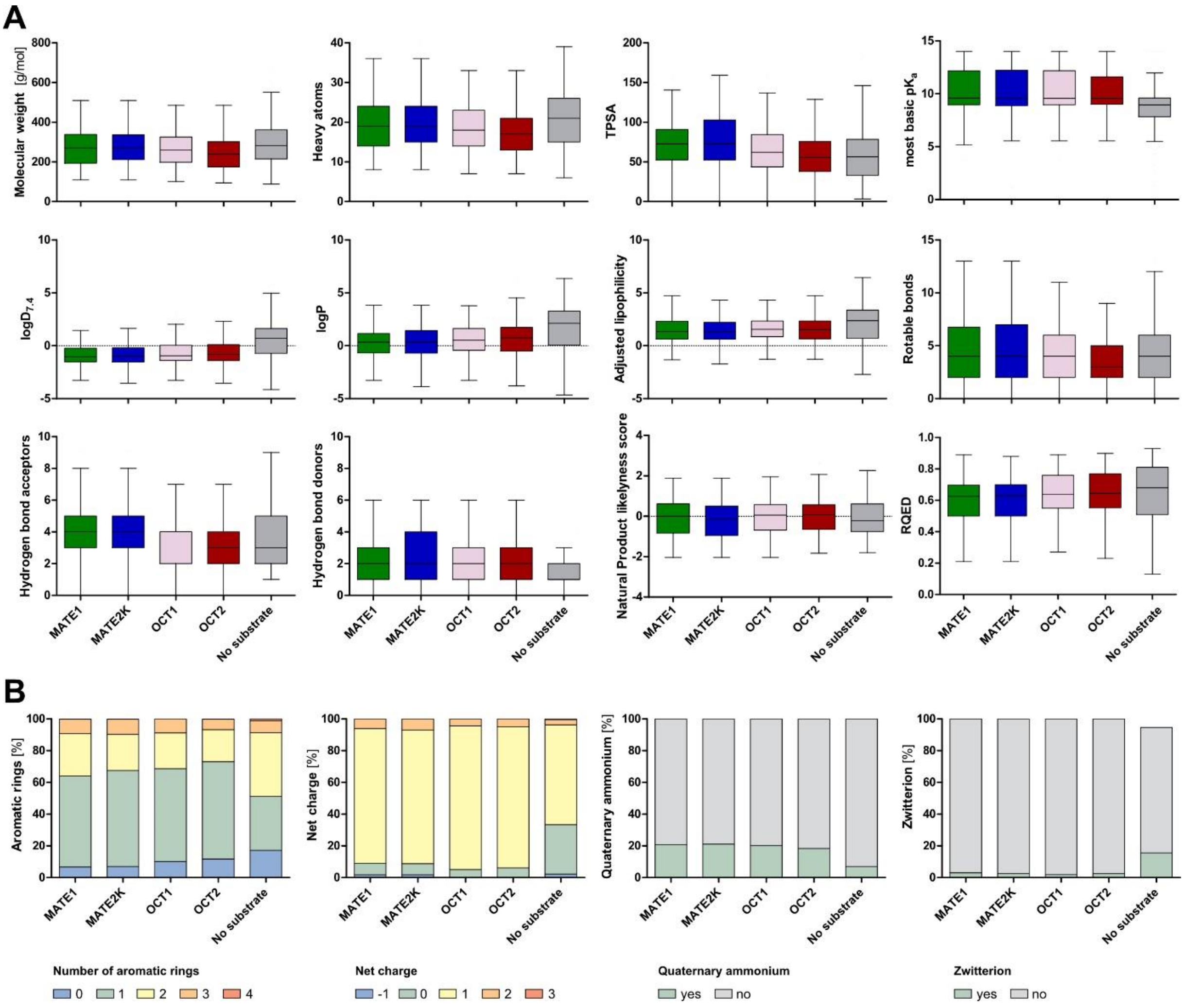

Figure S5: Comparison of basic chemical parameters between all substrates of MATE1, MATE2K, OCT1, and OCT2 as well as non-substrates with twelve parameters as box plots (A) and the percentile distribution of four further parameters in stacked bar charts (B).

Biochemical parameters of the tested compounds were obtained with MarvinSketch and the Instant JChem package from Chemaxon (version 21.2.0, Budapest, Hungary) and visualized using GraphPad Prism software (Version 5.01 for Windows, La Jolla, CA, USA). Natural product likeliness scores and Rqed were retrieved from the ChEMBL database in March 2025.

Figure S6: Sequence alignment of the four SLCs of interest

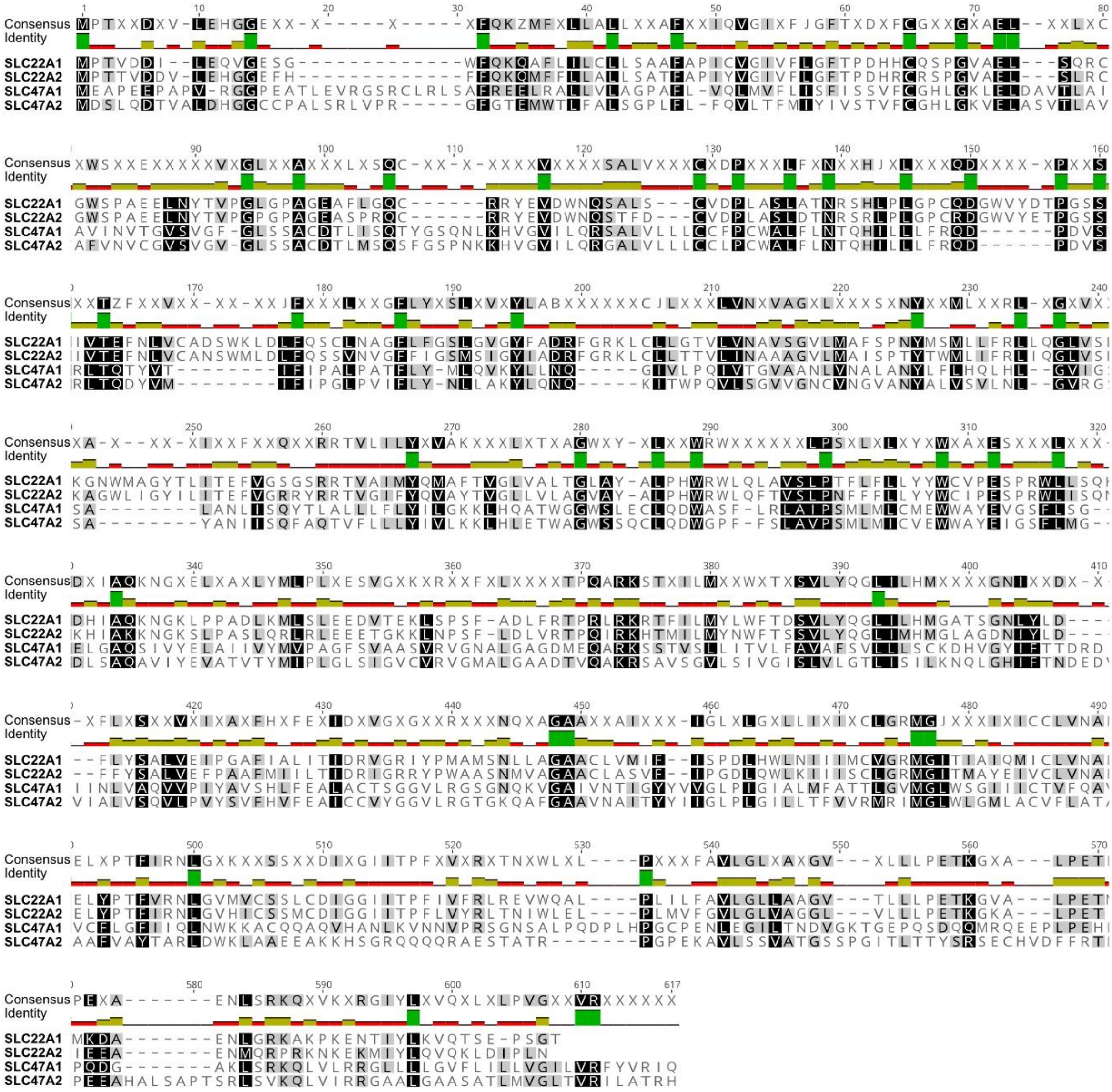

Figure S6: Sequence alignment of the four SLCs of interest.

The sequence homology analysis was performed with Geneious Prime 2023.0.1 using the MUSCLE 5.1 algorithm (https://www.geneious.com). The underlying colors black and grey indicate the grade of similarity between the sequences.

Figure S7: Chemical structures of MATE1 and MATE2K substrates in comparison to non-substrates.

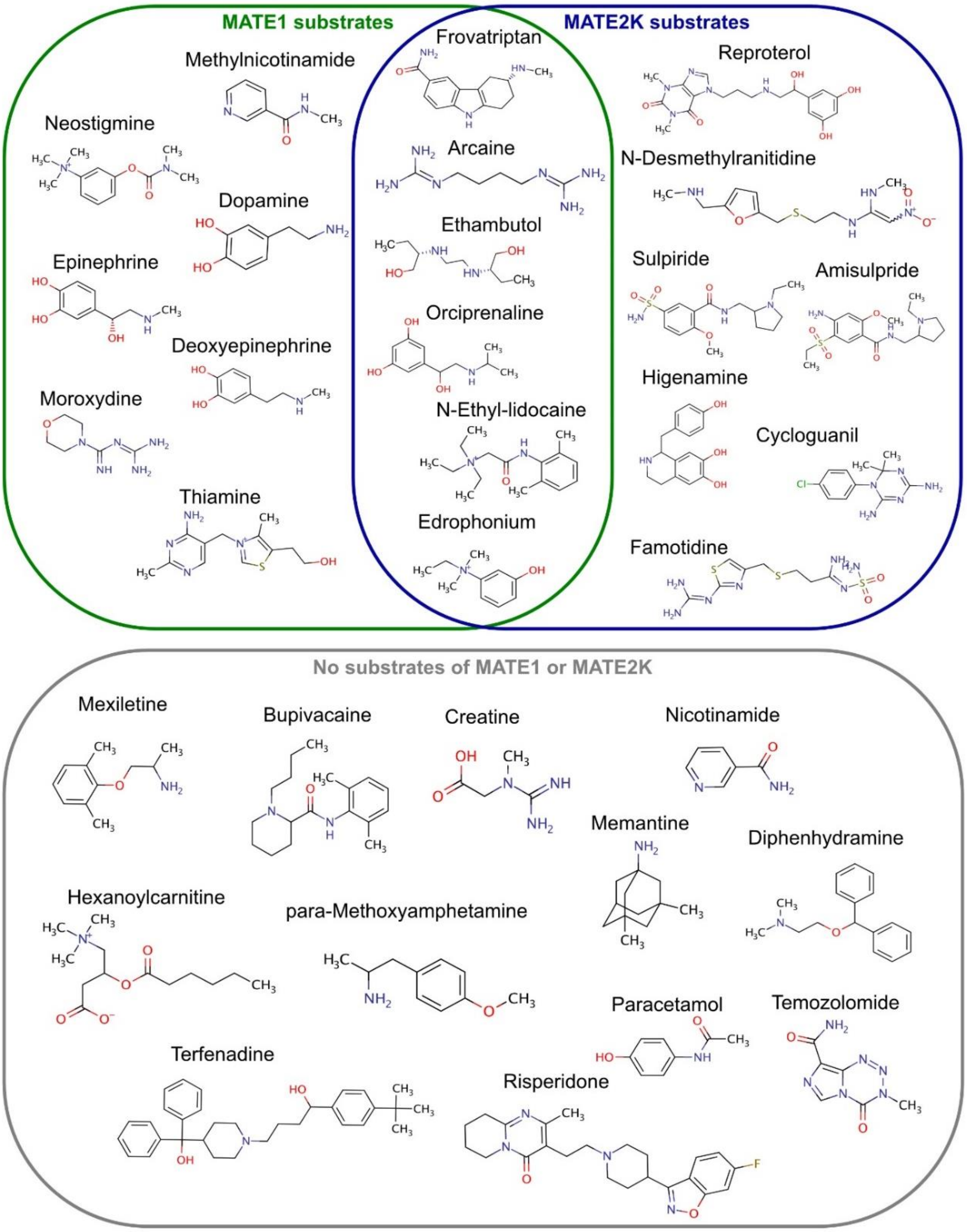

Figure S7: Chemical structures of MATE1 and MATE2K substrates in comparison to non-substrates.

The best twelve substrates for MATE1 and MATE2K are depicted with an overlapping substrate spectrum of 5 substances plus ethambutol. Substances that are no substrates were mostly selected on the basis of ratio between 1.0 and 1.3. Chemical structures were created with MarvinSketch (21.2, Chemaxon, Budapest, Hungary).

Figure S8: Testing of selected substrates for purity using HPLC and UV detection

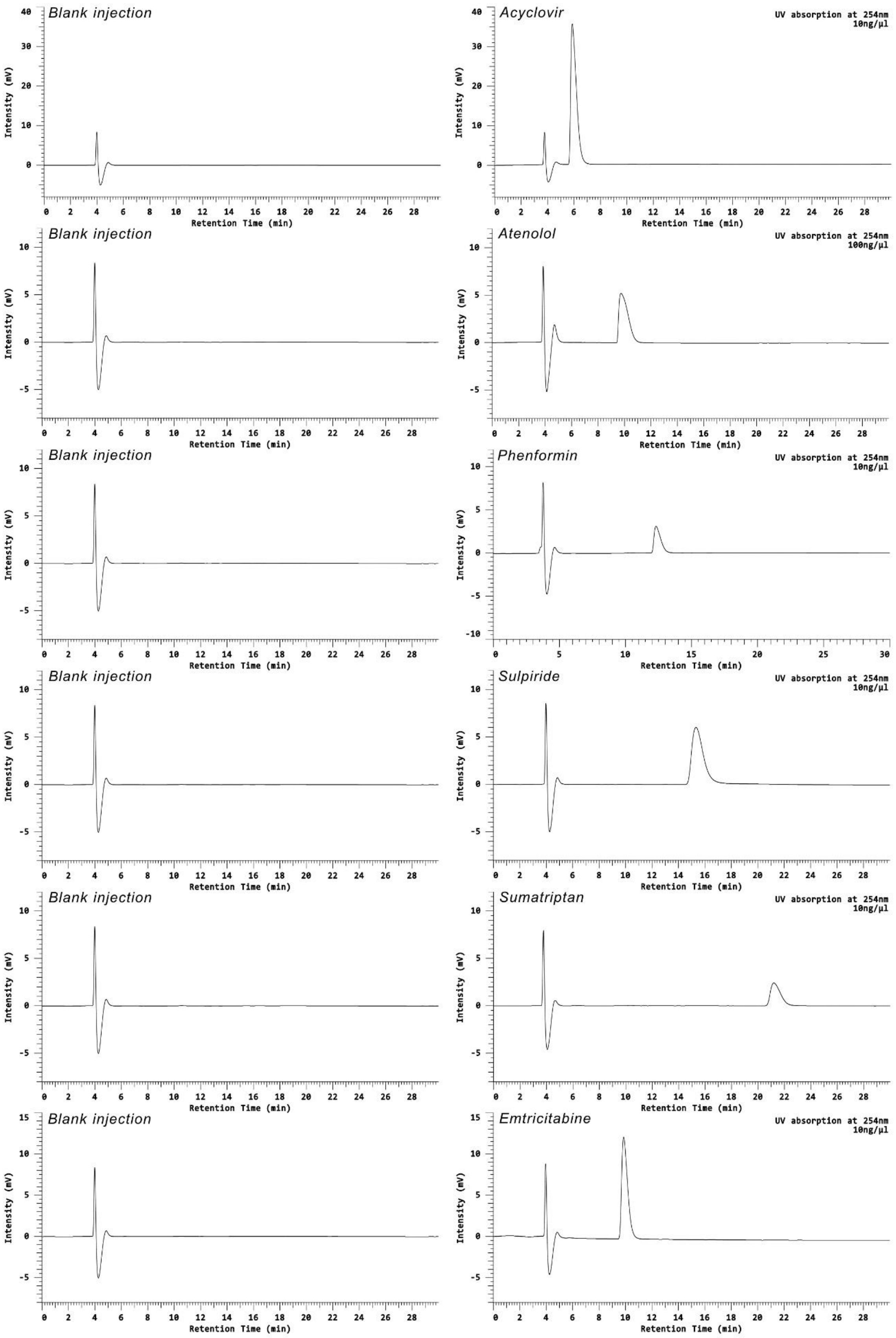

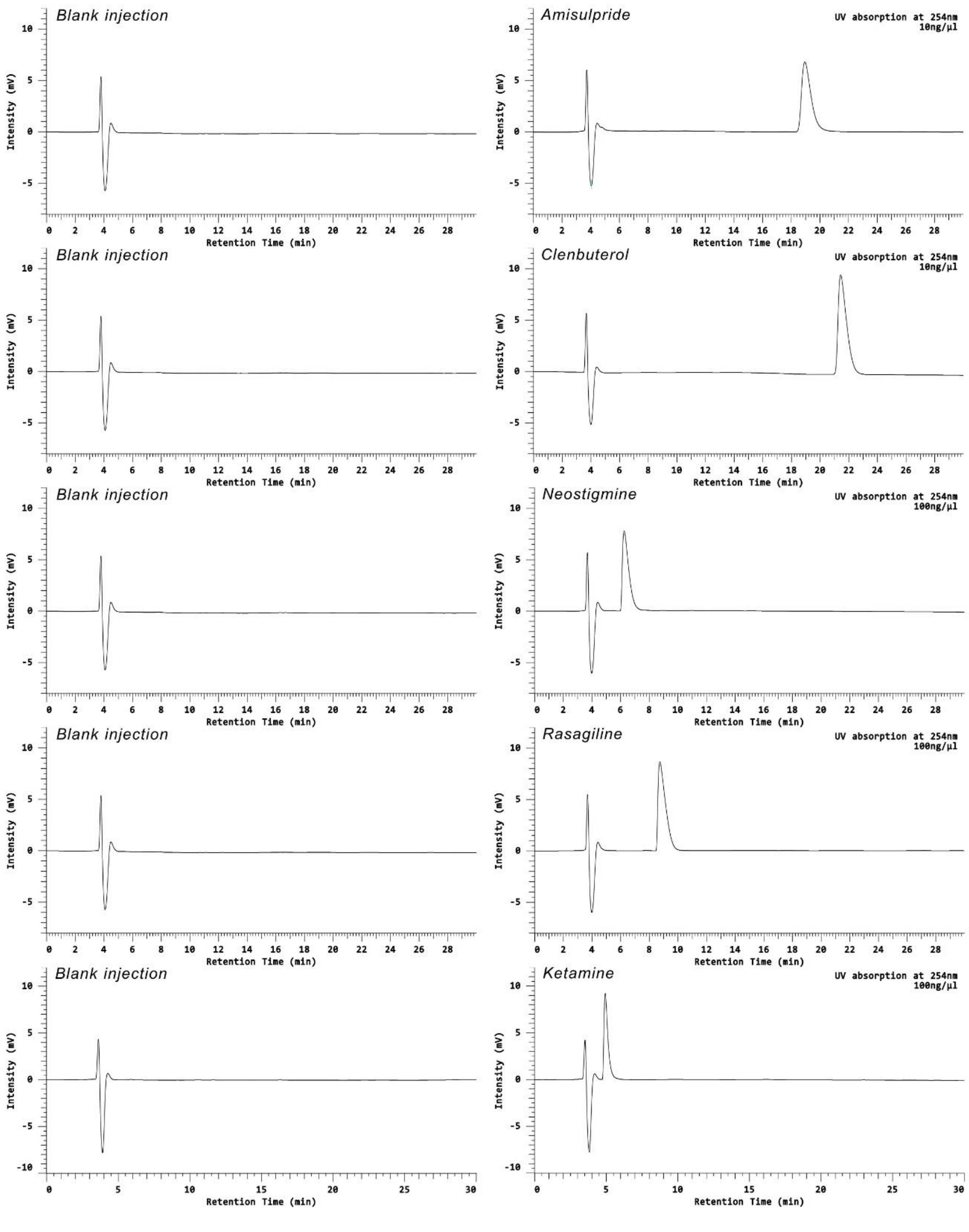

Figure S8: Testing of selected substrates for purity using HPLC and UV detection.

Eleven substances were chosen randomly and tested exemplarily for purity using HPLC and UV detection.

Figure S9: Scree plot for principle component analysis

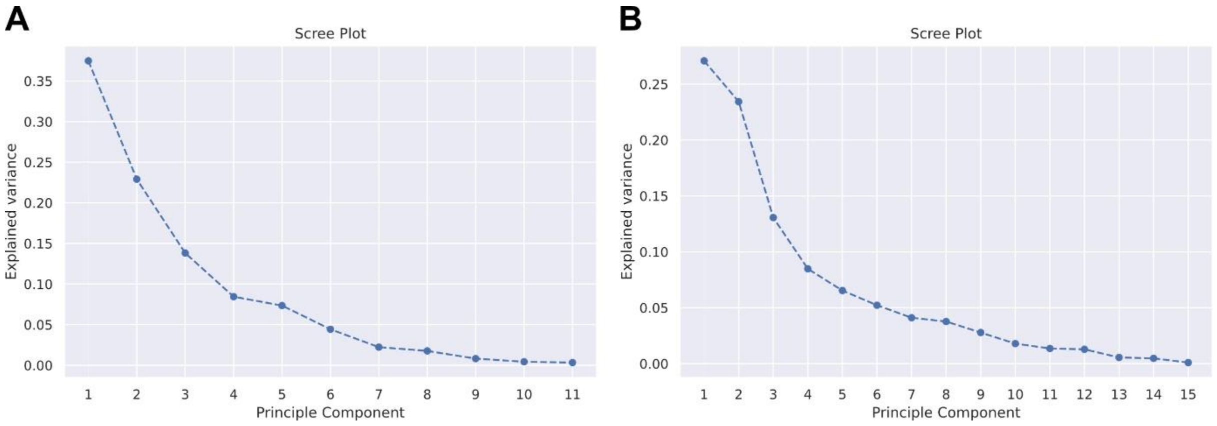

Figure S9: (A) Proportion of total variance explained by each principal component, when including the 11 features available for the 12,276 compounds in Drugbank. The features were Hydrogen bound acceptors, Hydrogen bound donors, LogP, LogS, Molecular weight, Number of rings, Physiological charge, most basic pKa, TPSA, Polarizability, and rotatable bounds.

(B) Explained variance of the principal components when including 15 features for the 590 components experimentally analyzed as MATE substrates. The plot illustrates the proportion of total variance explained by each principal component. The physicochemical predictors available for our dataset tested for 590 substances were not identical with those in the DrugBank data. The features used in our dataset were molecular weight, LogD (pH 7.4), most basic pKa, TPSA, Physiological charge, Quarternary N, Zwitterionic, LogP, Hydrogen bound acceptors, Hydrogen bound donors, Aromatic rings, Heavy atoms, Natural product likelyness score, rqed (weighted), and rotable bounds.

Table S1: Known substrates of MATE1 and MATE2K from literature

Substrates	MATE1	MATE1 ratio in this study	SEM	MATE2K	MATE2K ratio in this study	SEM	Reference
1-Butyl-1-methyl-pyrrolidinium	+						1
1-Butyl-3-methylimidazolium	+						1
1-Methyl-4-phenylpyridinium (MPP)	+	23.35	0.34	+	9.05	0.46	2-6
2-Sulfanylethane sulfonate (Mesna)	+						7
3-Guanidino-propanoate	+						8
4-(4-(Dimethylamino)styryl) -N-methylpyridinium (ASP)	+			+			9
4′,6-Diamino-2-phenylindole (DAPI)	+			+			10
4-Guanidino-propanoate	+						8
5-Methylcytidine	+						8
Acyclovir	+	3.25	0.42	+	2.69	0.42	3
Agmatine	+	1.08	0.18		1.05	0.13	11
Amiloride	+	11.06	1.14		6.80	0.30	12
Amphetamine	+	0.96	0.13	+	1.68	0.51	13
Asymmetric dimethylarginine (ADMA)	+			+			14
Atecegatran	+						15
Atenolol	+	18.66	0.70	+	4.56	1.12	16
Atenolol (R)	+	25.72	2.00	+	6.79	0.47	17
Atenolol (S)	+	25.17	1.04	+	9.28	1.22	17
Baricitinib		0.82	0.15	+	1.04	0.18	18
Berberine	+	8.83	0.12		4.40	0.25	19
Butylscopolamine	+	23.84	1.72	-	1.70	0.09	20
Capecitabine	+						8
Cephalexin	+			-			2, 3, 21
Cephradine	+			-			2, 3
Chloroquine	+	1.40	0.14		1.06	0.14	22
Cimetidine	+	11.52	1.73	+	3.60	0.19	2-4, 23
Cisplatin	+			+			24, 25
Creatinine	+	0.83	0.05	+	1.07	0.07	2-4, 26, 27
Cycloguanil	+	21.02	0.83	+	10.75	0.33	28
Cytarabine	+	0.89	0.02		1.04	0.05	8
Dabigatran	+						29
Debrisoquine	+	8.16	0.33	+	5.05	0.96	30
Dofetilide	+	4.66	0.13		4.15	0.13	31
Emtricitabine	+	3.22	0.16		2.88	0.15	32
Entecavir	+	0.96	0.09	+	1.07	0.09	33
Estrone 3-sulfate	+	4.80	0.41	+	2.08	0.37	3
Ethambutol	+	14.14	2.57		2.78	0.16	34
Fenoterol	+	14.98	0.65	+	5.46	0.40	17
Fexofenadine	+	1.63	0.36		1.30	0.44	35
Flucytosine	+						8
Formoterol	+	3.64	0.49	+	1.38	0.13	17

Substrates	MATE1	MATE1 ratio in this study	SEM	MATE2K	MATE2K ratio in this study	SEM	Reference
Formoterol (R)	+	5.18	0.32	+	1.72	0.10	[17]
Ganciclovir	+	1.38	0.30	+	1.68	0.11	[3]
Gemcitabine	+	1.29	0.18		1.08	0.20	[8]
Guanidine	+			+			[3, 4]
Imatinib	+	1.28	0.16		1.07	0.11	[36]
Imeglimin	+	18.76	0.92	+	4.59	0.26	[37]
Ipratropium	+	24.31	1.93	+	6.36	1.53	[20]
Lamivudine	+	11.10	1.48	+	3.36	0.56	[38]
L-Arginine	+			+			[14]
Levofloxacin	+	1.93	0.09		1.41	0.04	[3]
Memantine	+	1.03	0.08	+	1.18	0.07	[39]
Metamphetamine	+	1.53	0.18	+	1.97	0.26	[13]
Metformin	+	0.71	0.19	+	1.10	0.32	[2-4]
Nadolol	+	9.41	0.81	+	10.12	1.11	[40]
N-Butylpyridinium	+			+			[1]
Nitidine	+						[41]
N-Methylnicotinamide (NMN)		33.89	0.19	+	5.40	0.90	[2, 42]
Oxaliplatin	+			+			[24, 25]
Paraquat	+						[43]
Pramipexole	+			+			[44]
Procainamide	+	2.53	0.19	+	2.18	0.06	[2-4]
Proguanil	+	5.54	0.23		3.98	0.24	[28]
Quercetin	+						[45]
Quinine	+	1.22	0.13	+	0.97	0.14	[3]
Salbutamol	+	7.94	0.45	+	3.73	0.19	[17]
Salbutamol (R)	+	6.98	0.48	+	3.74	0.30	[17]
Salbutamol (S)	+	8.88	0.32	+	5.09	0.42	[17]
Sparteine	+	2.12	0.11	+	1.66	0.13	[30]
Sulpiride	+	23.85	1.07	+	21.71	1.58	[46]
Telbivudine	+						[8]
Testosterone	+						[47]
Tetraethyl ammonium (TEA)	+	25.44	1.15	+	6.73	0.49	[2, 3, 48]
Thiamine	+	33.42	6.84	+	10.63	0.95	[2, 3, 49]
Tofacitinib	+	1.36	0.27		1.41	0.23	[18]
Topotecan	+	2.53	0.24	+	1.61	0.13	[3]
Trospium	+	21.28	0.83	+	5.18	0.15	[50]
Tyramine	+	10.06	1.02		1.39	0.08	[51]
Uridine triacetate	+						[8]
Varenicline	+	1.20	0.09	+	1.30	0.05	[52]
Verapamil		1.22	0.02	+	1.08	0.05	[2, 3]
Zalcitabine	+	12.98	0.90		1.22	0.06	[8]
β-Hydroxycortisol	+			+			[53]
Substrates newly identified here	129			91			
Substrates identified earlier and confirmed here	35			23			
Substrates identified earlier but not confirmed here	17			15			

Substrates	MATE1	MATE1 ratio in this study	SEM	MATE2K	MATE2K ratio in this study	SEM	Reference
Substrates identified earlier but not studied here	29			10			

References for Table S1:

1. Martínez-Guerrero, L. J.; Wright, S. H. Substrate-dependent inhibition of human MATE1 by cationic ionic liquids. *The Journal of pharmacology and experimental therapeutics* **2013,** 346, 495-503.
2. Masuda, S.; Terada, T.; Yonezawa, A.; Tanihara, Y.; Kishimoto, K.; Katsura, T.; Ogawa, O.; Inui, K. Identification and functional characterization of a new human kidney-specific H+/organic cation antiporter, kidney-specific multidrug and toxin extrusion 2. *Journal of the American Society of Nephrology : JASN* **2006,** 17, 2127-35.
3. Tanihara, Y.; Masuda, S.; Sato, T.; Katsura, T.; Ogawa, O.; Inui, K. Substrate specificity of MATE1 and MATE2-K, human multidrug and toxin extrusions/H(+)-organic cation antiporters. *Biochemical pharmacology* **2007,** 74, 359-71.
4. Sato, T.; Masuda, S.; Yonezawa, A.; Tanihara, Y.; Katsura, T.; Inui, K. Transcellular transport of organic cations in double-transfected MDCK cells expressing human organic cation transporters hOCT1/hMATE1 and hOCT2/hMATE1. *Biochemical pharmacology* **2008,** 76, 894-903.
5. Matsumoto, T.; Kanamoto, T.; Otsuka, M.; Omote, H.; Moriyama, Y. Role of glutamate residues in substrate recognition by human MATE1 polyspecific H+/organic cation exporter. *American journal of physiology. Cell physiology* **2008,** 294, C1074-8.
6. Han, Y. H.; Busler, D.; Hong, Y.; Tian, Y.; Chen, C.; Rodrigues, A. D. Transporter studies with the 3-O-sulfate conjugate of 17alpha-ethinylestradiol: assessment of human kidney drug transporters. *Drug metabolism and disposition: the biological fate of chemicals* **2010,** 38, 1064-71.
7. Cutler, M. J.; Urquhart, B. L.; Velenosi, T. J.; Meyer Zu Schwabedissen, H. E.; Dresser, G. K.; Leake, B. F.; Tirona, R. G.; Kim, R. B.; Freeman, D. J. In vitro and in vivo assessment of renal drug transporters in the disposition of mesna and dimesna. *Journal of clinical pharmacology* **2012,** 52, 530-42.
8. Nies, A. T.; König, J.; Leuthold, P.; Damme, K.; Winter, S.; Haag, M.; Masuda, S.; Kruck, S.; Daniel, H.; Spanier, B.; Fromm, M. F.; Bedke, J.; Inui, K. I.; Schwab, M.; Schaeffeler, E. Novel drug transporter substrates identification: An innovative approach based on metabolomic profiling, in silico ligand screening and biological validation. *Pharmacological research* **2023,** 196, 106941.
9. Kido, Y.; Matsson, P.; Giacomini, K. M. Profiling of a prescription drug library for potential renal drug-drug interactions mediated by the organic cation transporter 2. *Journal of medicinal chemistry* **2011,** 54, 4548-58.
10. Yasujima, T.; Ohta, K. Y.; Inoue, K.; Ishimaru, M.; Yuasa, H. Evaluation of 4',6-diamidino-2-phenylindole as a fluorescent probe substrate for rapid assays of the functionality of human multidrug and toxin extrusion proteins. *Drug metabolism and disposition: the biological fate of chemicals* **2010,** 38, 715-21.
11. Winter, T. N.; Elmquist, W. F.; Fairbanks, C. A. OCT2 and MATE1 provide bidirectional agmatine transport. *Molecular pharmaceutics* **2011,** 8, 133-42.
12. Kawasaki, T.; Kaneko, C.; Nakanishi, R.; Moriyama, Y.; Nabekura, T. Amiloride is a suitable fluorescent substrate for the study of the drug transporter human multidrug and toxin extrusion 1 (MATE1). *Biochemical and biophysical research communications* **2022,** 592, 113-118.
13. Wagner, D. J.; Sager, J. E.; Duan, H.; Isoherranen, N.; Wang, J. Interaction and Transport of Methamphetamine and its Primary Metabolites by Organic Cation and Multidrug and Toxin Extrusion Transporters. *Drug metabolism and disposition: the biological fate of chemicals* **2017,** 45, 770-778.

14. Strobel, J.; Müller, F.; Zolk, O.; Endreß, B.; König, J.; Fromm, M. F.; Maas, R. Transport of asymmetric dimethylarginine (ADMA) by cationic amino acid transporter 2 (CAT2), organic cation transporter 2 (OCT2) and multidrug and toxin extrusion protein 1 (MATE1). *Amino acids* **2013,** 45, 989-1002.
15. Matsson, E. M.; Eriksson, U. G.; Palm, J. E.; Artursson, P.; Karlgren, M.; Lazorova, L.; Brännström, M.; Ekdahl, A.; Dunér, K.; Knutson, L.; Johansson, S.; Schützer, K. M.; Lennernäs, H. Combined in vitro-in vivo approach to assess the hepatobiliary disposition of a novel oral thrombin inhibitor. *Molecular pharmaceutics* **2013,** 10, 4252-62.
16. Yin, J.; Duan, H.; Shirasaka, Y.; Prasad, B.; Wang, J. Atenolol Renal Secretion Is Mediated by Human Organic Cation Transporter 2 and Multidrug and Toxin Extrusion Proteins. *Drug metabolism and disposition: the biological fate of chemicals* **2015,** 43, 1872-81.
17. Jensen, O.; Rafehi, M.; Tzvetkov, M. V.; Brockmöller, J. Stereoselective cell uptake of adrenergic agonists and antagonists by organic cation transporters. *Biochemical pharmacology* **2020,** 171, 113731.
18. Amrhein, J.; Drynda, S.; Schlatt, L.; Karst, U.; Lohmann, C. H.; Ciarimboli, G.; Bertrand, J. Tofacitinib and Baricitinib Are Taken up by Different Uptake Mechanisms Determining the Efficacy of Both Drugs in RA. *International journal of molecular sciences* **2020,** 21.
19. Xiao, L.; Xue, Y.; Zhang, C.; Wang, L.; Lin, Y.; Pan, G. The involvement of multidrug and toxin extrusion protein 1 in the distribution and excretion of berberine. *Xenobiotica; the fate of foreign compounds in biological systems* **2018,** 48, 314-323.
20. Chen, J.; Brockmöller, J.; Seitz, T.; König, J.; Tzvetkov, M. V.; Chen, X. Erratum to: Tropane alkaloids as substrates and inhibitors of human organic cation transporters of the SLC22 (OCT) and the SLC47 (MATE) families. *Biol Chem* **2017,** 398, 813.
21. Watanabe, S.; Tsuda, M.; Terada, T.; Katsura, T.; Inui, K. Reduced renal clearance of a zwitterionic substrate cephalexin in MATE1-deficient mice. *The Journal of pharmacology and experimental therapeutics* **2010,** 334, 651-6.
22. Müller, F.; König, J.; Glaeser, H.; Schmidt, I.; Zolk, O.; Fromm, M. F.; Maas, R. Molecular mechanism of renal tubular secretion of the antimalarial drug chloroquine. *Antimicrobial agents and chemotherapy* **2011,** 55, 3091-8.
23. Ohta, K. Y.; Inoue, K.; Yasujima, T.; Ishimaru, M.; Yuasa, H. Functional characteristics of two human MATE transporters: kinetics of cimetidine transport and profiles of inhibition by various compounds. *Journal of pharmacy & pharmaceutical sciences : a publication of the Canadian Society for Pharmaceutical Sciences, Societe canadienne des sciences pharmaceutiques* **2009,** 12, 388-96.
24. Yonezawa, A.; Masuda, S.; Yokoo, S.; Katsura, T.; Inui, K. Cisplatin and oxaliplatin, but not carboplatin and nedaplatin, are substrates for human organic cation transporters (SLC22A1-3 and multidrug and toxin extrusion family). *The Journal of pharmacology and experimental therapeutics* **2006,** 319, 879-86.
25. Yokoo, S.; Yonezawa, A.; Masuda, S.; Fukatsu, A.; Katsura, T.; Inui, K. Differential contribution of organic cation transporters, OCT2 and MATE1, in platinum agent-induced nephrotoxicity. *Biochemical pharmacology* **2007,** 74, 477-87.
26. Lepist, E. I.; Zhang, X.; Hao, J.; Huang, J.; Kosaka, A.; Birkus, G.; Murray, B. P.; Bannister, R.; Cihlar, T.; Huang, Y.; Ray, A. S. Contribution of the organic anion transporter OAT2 to the renal active tubular secretion of creatinine and mechanism for serum creatinine elevations caused by cobicistat. *Kidney international* **2014,** 86, 350-7.
27. Shen, H.; Liu, T.; Morse, B. L.; Zhao, Y.; Zhang, Y.; Qiu, X.; Chen, C.; Lewin, A. C.; Wang, X. T.; Liu, G.; Christopher, L. J.; Marathe, P.; Lai, Y. Characterization of Organic Anion Transporter 2 (SLC22A7): A Highly Efficient Transporter for Creatinine and Species-Dependent Renal Tubular Expression. *Drug metabolism and disposition: the biological fate of chemicals* **2015,** 43, 984-93.
28. van der Velden, M.; Bilos, A.; van den Heuvel, J.; Rijpma, S. R.; Hurkmans, E. G. E.; Sauerwein, R. W.; Russel, F. G. M.; Koenderink, J. B. Proguanil and cycloguanil are organic cation transporter and multidrug and toxin extrusion substrates. *Malaria journal* **2017,** 16, 422.
29. Shen, H.; Yao, M.; Sinz, M.; Marathe, P.; Rodrigues, A. D.; Zhu, M. Renal Excretion of Dabigatran: The Potential Role of Multidrug and Toxin Extrusion (MATE) Proteins. *Molecular pharmaceutics* **2019,** 16, 4065-4076.

30. Neul, C.; Hofmann, U.; Schaeffeler, E.; Winter, S.; Klein, K.; Giacomini, K. M.; Eichelbaum, M.; Schwab, M.; Nies, A. T. Characterization of cytochrome P450 (CYP) 2D6 drugs as substrates of human organic cation transporters and multidrug and toxin extrusion proteins. *British journal of pharmacology* **2021,** 178, 1459-1474.
31. Uddin, M. E.; Eisenmann, E. D.; Li, Y.; Huang, K. M.; Garrison, D. A.; Talebi, Z.; Gibson, A. A.; Jin, Y.; Nepal, M.; Bonilla, I. M.; Fu, Q.; Sun, X.; Millar, A.; Tarasov, M.; Jay, C. E.; Cui, X.; Einolf, H. J.; Pelis, R. M.; Smith, S. A.; Radwański, P. B.; Sweet, D. H.; König, J.; Fromm, M. F.; Carnes, C. A.; Hu, S.; Sparreboom, A. MATE1 Deficiency Exacerbates Dofetilide-Induced Proarrhythmia. *International journal of molecular sciences* **2022,** 23.
32. Reznicek, J.; Ceckova, M.; Cerveny, L.; Müller, F.; Staud, F. Emtricitabine is a substrate of MATE1 but not of OCT1, OCT2, P-gp, BCRP or MRP2 transporters. *Xenobiotica; the fate of foreign compounds in biological systems* **2017,** 47, 77-85.
33. Yang, X.; Ma, Z.; Zhou, S.; Weng, Y.; Lei, H.; Zeng, S.; Li, L.; Jiang, H. Multiple Drug Transporters Are Involved in Renal Secretion of Entecavir. *Antimicrobial agents and chemotherapy* **2016,** 60, 6260-70.
34. Te Brake, L. H.; van den Heuvel, J. J.; Buaben, A. O.; van Crevel, R.; Bilos, A.; Russel, F. G.; Aarnoutse, R. E.; Koenderink, J. B. Moxifloxacin Is a Potent In Vitro Inhibitor of OCT- and MATE-Mediated Transport of Metformin and Ethambutol. *Antimicrobial agents and chemotherapy* **2016,** 60, 7105-7114.
35. Matsushima, S.; Maeda, K.; Inoue, K.; Ohta, K. Y.; Yuasa, H.; Kondo, T.; Nakayama, H.; Horita, S.; Kusuhara, H.; Sugiyama, Y. The inhibition of human multidrug and toxin extrusion 1 is involved in the drug-drug interaction caused by cimetidine. *Drug metabolism and disposition: the biological fate of chemicals* **2009,** 37, 555-9.
36. Schmidt-Lauber, C.; Harrach, S.; Pap, T.; Fischer, M.; Victor, M.; Heitzmann, M.; Hansen, U.; Fobker, M.; Brand, S. M.; Sindic, A.; Pavenstädt, H.; Edemir, B.; Schlatter, E.; Bertrand, J.; Ciarimboli, G. Transport mechanisms and their pathology-induced regulation govern tyrosine kinase inhibitor delivery in rheumatoid arthritis. *PloS one* **2012,** 7, e52247.
37. Clémence, C.; Fouqueray, P.; Sébastien, B. In Vitro Investigation, Pharmacokinetics, and Disposition of Imeglimin, a Novel Oral Antidiabetic Drug, in Preclinical Species and Humans. *Drug metabolism and disposition: the biological fate of chemicals* **2020,** 48, 1330-1346.
38. Müller, F.; König, J.; Hoier, E.; Mandery, K.; Fromm, M. F. Role of organic cation transporter OCT2 and multidrug and toxin extrusion proteins MATE1 and MATE2-K for transport and drug interactions of the antiviral lamivudine. *Biochemical pharmacology* **2013,** 86, 808-15.
39. Müller, F.; Weitz, D.; Derdau, V.; Sandvoss, M.; Mertsch, K.; König, J.; Fromm, M. F. Contribution of MATE1 to Renal Secretion of the NMDA Receptor Antagonist Memantine. *Molecular pharmaceutics* **2017,** 14, 2991-2998.
40. Misaka, S.; Knop, J.; Singer, K.; Hoier, E.; Keiser, M.; Müller, F.; Glaeser, H.; König, J.; Fromm, M. F. The Nonmetabolized β-Blocker Nadolol Is a Substrate of OCT1, OCT2, MATE1, MATE2-K, and P-Glycoprotein, but Not of OATP1B1 and OATP1B3. *Molecular pharmaceutics* **2016,** 13, 512-9.
41. Li, L.; Tu, M.; Yang, X.; Sun, S.; Wu, X.; Zhou, H.; Zeng, S.; Jiang, H. The contribution of human OCT1, OCT3, and CYP3A4 to nitidine chloride-induced hepatocellular toxicity. *Drug metabolism and disposition: the biological fate of chemicals* **2014,** 42, 1227-34.
42. Ito, S.; Kusuhara, H.; Kumagai, Y.; Moriyama, Y.; Inoue, K.; Kondo, T.; Nakayama, H.; Horita, S.; Tanabe, K.; Yuasa, H.; Sugiyama, Y. N-methylnicotinamide is an endogenous probe for evaluation of drug-drug interactions involving multidrug and toxin extrusions (MATE1 and MATE2-K). *Clinical pharmacology and therapeutics* **2012,** 92, 635-41.
43. Chen, Y.; Zhang, S.; Sorani, M.; Giacomini, K. M. Transport of paraquat by human organic cation transporters and multidrug and toxic compound extrusion family. *The Journal of pharmacology and experimental therapeutics* **2007,** 322, 695-700.
44. Knop, J.; Hoier, E.; Ebner, T.; Fromm, M. F.; Müller, F. Renal tubular secretion of pramipexole. *European journal of pharmaceutical sciences : official journal of the European Federation for Pharmaceutical Sciences* **2015,** 79, 73-8.

45. Lee, J. H.; Lee, J. E.; Kim, Y.; Lee, H.; Jun, H. J.; Lee, S. J. Multidrug and toxic compound extrusion protein-1 (MATE1/SLC47A1) is a novel flavonoid transporter. *Journal of agricultural and food chemistry* **2014,** 62, 9690-8.
46. Takano, H.; Ito, S.; Zhang, X.; Ito, H.; Zhang, M. R.; Suzuki, H.; Maeda, K.; Kusuhara, H.; Suhara, T.; Sugiyama, Y. Possible Role of Organic Cation Transporters in the Distribution of [(11)C]Sulpiride, a Dopamine D(2) Receptor Antagonist. *Journal of pharmaceutical sciences* **2017,** 106, 2558-2565.
47. Goda, M.; Ikehara, M.; Sakitani, M.; Oda, K.; Ishizawa, K.; Otsuka, M. Involvement of Human Multidrug and Toxic Compound Extrusion (MATE) Transporters in Testosterone Transport. *Biological & pharmaceutical bulletin* **2021,** 44, 501-506.
48. Otsuka, M.; Matsumoto, T.; Morimoto, R.; Arioka, S.; Omote, H.; Moriyama, Y. A human transporter protein that mediates the final excretion step for toxic organic cations. *Proceedings of the National Academy of Sciences of the United States of America* **2005,** 102, 17923-8.
49. Kato, K.; Mori, H.; Kito, T.; Yokochi, M.; Ito, S.; Inoue, K.; Yonezawa, A.; Katsura, T.; Kumagai, Y.; Yuasa, H.; Moriyama, Y.; Inui, K.; Kusuhara, H.; Sugiyama, Y. Investigation of endogenous compounds for assessing the drug interactions in the urinary excretion involving multidrug and toxin extrusion proteins. *Pharmaceutical research* **2014,** 31, 136-47.
50. Deutsch, B.; Neumeister, C.; Schwantes, U.; Fromm, M. F.; König, J. Interplay of the Organic Cation Transporters OCT1 and OCT2 with the Apically Localized Export Protein MATE1 for the Polarized Transport of Trospium. *Molecular pharmaceutics* **2019,** 16, 510-517.
51. Ma, Y.; Wang, X.; Gou, X.; Wu, X. Identification and characterization of an endogenous biomarker of the renal vectorial transport (OCT2-MATE1). *Biopharmaceutics & drug disposition* **2024,** 45, 43-57.
52. Kajiwara, M.; Masuda, S.; Watanabe, S.; Terada, T.; Katsura, T.; Inui, K. Renal tubular secretion of varenicline by multidrug and toxin extrusion (MATE) transporters. *Drug metabolism and pharmacokinetics* **2012,** 27, 563-9.
53. Imamura, Y.; Murayama, N.; Okudaira, N.; Kurihara, A.; Inoue, K.; Yuasa, H.; Izumi, T.; Kusuhara, H.; Sugiyama, Y. Effect of the fluoroquinolone antibacterial agent DX-619 on the apparent formation and renal clearances of 6β-hydroxycortisol, an endogenous probe for CYP3A4 inhibition, in healthy subjects. *Pharmaceutical research* **2013,** 30, 447-57.

Table S2: Screening of 590 substances as possible substrates of MATE1 and MATE2K

Substance	MATE1 ratio	SEM	MATE2K ratio	SEM	Therapeutic group	SMILES code	Molecular weight	logD 7.4	most basic pKa	TPSA	Charge	TS DPH hCMEC	SEM	TS MDAI hCMEC	SEM
1-(3-Chlorophenyl)piperazine	1.27	0.16	0.89	0.21	Antidepressant	C1CN(CCN1)C2=CC(=CC=C2)Cl	196.7	0.71	8.83	15.3	+1				
1-(4-Chlorophenyl)biguanide	6.97	0.63	3.82	0.47	Antiinfective	C1=CC(=CC=C1N=C(N)N=C(N)N)Cl	211.7	-1.27	11.82	102.8	+2				
1,3-Diphenylguanidine	1.53	0.15	1.27	0.04	Experimental chemical	C1=CC=C(C=C1)NC(=NC2=CC=CC=C2)N	211.3	1.22	10.93	50.4	+1				
10-OH-Nortriptyline	1.17	0.16	1.14	0.11	Antidepressant	CNCC/C=C\1/C2=CC=CC=C2CC(C3=CC=CC=C31)O	279.4	0.35	10.47	32.3	+1				
17-Alpha-methyltestosterone	1.17	0.09	1.22	0.09	Steroid hormone	C[C@]12CCC(=O)C=C1CC[C@@H]3[C@@H]2CC[C@]4([C@H]3CC[C@]4(C)O)C	302.2	3.65		37.3	0				
1-Methyl-4-phenylpyridinium	23.35	0.34	9.05	0.46	Experimental chemical	C[N+]1=CC=C(C=C1)C2=CC=CC=C2	170.2	-1.54		3.9	+1				
1-Methylxanthine	1.08	0.09	0.96	0.04	Xanthine derivative	CN1C(=O)C2=C(NC1=O)N=CN2	166.1	0.08		78.1	0				
2,5-Dimethoxy-4-iodoamphetamine	1.28	0.09	1.16	0.03	Psychostimulant	CC(CC1=CC(=C(C=C1OC)I)OC)N	321.2	0.08	9.83	44.5	+1	0.18	0.00		
2-Dimethylaminoethanol	0.79	0.10	0.91	0.18	Experimental chemical	CN(C)CCO	89.1	-2.13	9.03	23.5	+1				
2-Methylamino-1-(3,4-methylendioxyphenyl)butan	1.35	0.13	1.24	0.04	Psychostimulant	CCC(CC1=CC2=C(C=C1)OCO2)NC	207.3	-0.34	10.28	30.5	+1				
2-Methylbutyrylcarnitine	0.61	0.05	1.24	0.14	Carnitine ester	CCC(C)C(=O)OC(CC(=O)[O-])C[N+](C)(C)C	245.3	-1.99		66.4	+1-1				
2-Phenylethylamine	1.39	0.10	1.43	0.07	Biogenic amine	C1=CC=C(C=C1)CCN	121.2	-0.94	9.82	26.0	+1				
3,4-Methylendioxy-N-methylamphetamine	1.14	0.08	1.18	0.08	Psychostimulant	CC(CC1=CC2=C(C=C1)OCO2)NC	193.2	-0.76	10.14	30.5	+1	0.06	0.01		
3-Iodothyronamine (T1AM)	1.36	0.19	1.07	0.16	Others	C1=CC(=CC=C1O)OC2=C(C=C(C=C2)CCN)I	355.2	1.31	10.00	55.5	+1	0.86	0.02	1.11	0.13
3-Methoxymorphinan	1.14	0.13	1.19	0.18	Opioid	COC1=CC2=C(C[C@@H]3[C@H]4[C@@]2(CCCC4)CCN3)C=C1	257.4	0.43	10.22	21.3	+1				

Substance	MATE1 ratio	SEM	MATE2K ratio	SEM	Therapeutic group	SMILES code	Molecular weight	logD 7.4	most basic pKa	TPSA	Charge	TS DPH hCMEC	SEM	TS MDAI hCMEC	SEM
3-Methoxy-p-tyramine	4.05	0.06	1.39	0.03	Endobiotic	COC1=C(C=CC(=C1)CCN)O	167.2	-1.31	9.62	55.5	+1				
4-Amino-1,8-naphthalimide	1.19	0.06	1.16	0.01	Oncology	C1=CC2=C(C=CC3=C2C(=C1)C(=O)NC3=O)N	212.2	0.84	2.64	72.2	0				
4-Aminoantipyrine	2.16	0.47	1.38	0.10	Analgesic	CC1=C(C(=O)N(N1C)C2=CC=CC=C2)N	203.2	0.33	0.09	49.6	0	1.09	0.07	1.01	0.04
4-Hydroxydebrisoquine	9.59	0.46	3.34	0.05	Antihypertensive	C1C(C2=CC=CC=C2CN1C(=N)N)O	191.2	-2.27	12.34	73.3	+1				
4-Hydroxymexiletine	1.67	0.17	1.42	0.11	Antiarrhythmic	CC1=CC(=CC(=C1OCC(C)N)C)O	195.3	0.23	9.30	55.5	+1				
4-Methoxy-m-tyramine	3.09	0.19	1.19	0.02	Endobiotic	COC1=C(C=C(C=C1)CCN)O	167.2	-1.31	9.57	55.5	+1				
4-Methoxyphenethylamine	1.40	0.15	1.23	0.08	Endobiotic	COC1=CC=C(C=C1)CCN	151.2	-1.06	9.77	35.3	+1				
4-Methylaminoantipyrine	0.21	0.04	0.83	0.19	Analgesic	CC1=C(C(=O)N(N1C)C2=CC=CC=C2)NC	217.3	0.77	1.24	35.6	0	1.20	0.06	1.26	0.05
5,6-Methylenedioxy-2-aminoindane	1.14	0.05	1.24	0.04	Psychostimulant	C1C(CC2=CC3=C(C=C21)OCO3)N	177.2	-1.27	9.89	44.5	+1	0.23	0.04		
5,7-Dihydroxytryptamine	1.13	0.21	0.84	0.14	Experimental chemical	C1=C(C=C(C2=C1C(=CN2)CCN)O)O	192.2	-1.00	10.23	82.3	+1				
5-Aminoindazole	0.99	0.31	0.73	0.09	Experimental chemical	C1=CC2=C(C=C1N)C=NN2	133.2	0.47	3.40	54.7	0				
5-Aminolevulinic acid	0.75	0.05	1.03	0.04	Endobiotic	C(CC(=O)O)C(=O)CN	131.1	-3.37	7.84	80.4	+1-1				
5-Methoxytryptamine	3.49	0.14	1.66	0.02	Endobiotic	COC1=CC2=C(C=C1)NC=C2CCN	190.2	-0.97	9.78	51.0	+1				
6-beta-Naltrexol	1.20	0.07	1.20	0.05	Opioid	C1C[C@]2([C@H]3CC4=C5[C@@]2(CCN3CC6CC6)[C@H]([C@@H]1O)OC5=C(C=C4)O)O	343.4	-0.28	8.69	73.2	+1				
6-Hydroxydopamine	1.05	0.15	1.24	0.04	Experimental chemical	C1=C(C(=CC(=C1O)O)O)CCN	169.2	-1.42	9.17	86.7	+1				
Abacavir	0.94	0.01	1.25	0.10	Antiviral	C1CC1NC2=C3C(=NC(=N2)N)N(C=N3)[C@@H]4C[C@@H](C=C4)CO	286.3	0.35	6.37	101.9	0				
Abemaciclib	1.06	0.10	1.01	0.03	Oncology	CCN1CCN(CC1)CC2=CN=C(C=C2)NC3=NC=C(C(=N3)C4=CC5=C(C(=C4)F)N=C(N5C(C)C)C)F	506.6	3.36	8.40	75.0	+1				

Substance	MATE1 ratio	SEM	MATE2K ratio	SEM	Therapeutic group	SMILES code	Molecular weight	logD 7.4	most basic pKa	TPSA	Charge	TS DPH hCMEC	SEM	TS MDAI hCMEC	SEM
Abrocitinib	1.09	0.01	1.19	0.16	JAK inhibitor	CCCS(=O)(=O)NC1CC(C1)N(C)C2=NC=NC3=C2C=CN3	323.4	0.46	7.55	99.4	0				
Acebutolol	12.97	0.23	9.08	0.26	Beta blocker	CCCC(=O)NC1=CC(=C(C=C1)OCC(CNC(C)C)O)C(=O)C	336.4	-0.31	9.25	87.7	+1	0.84	0.04	0.88	0.04
Aceclidine	1.26	0.20	1.08	0.16	(Anti)cholinergic	CC(=O)OC1CN2CCC1CC2	169.2	-1.48	9.17	29.5	+1				
Acetylcarnitine	0.58	0.02	1.10	0.15	Carnitine ester	CC(=O)O[C@H](CC(=O)[O-])C[N+](C)(C)C	203.2	-3.68		66.4	+1-1				
Aclidinium	4.73	1.31	1.17	0.32	(Anti)cholinergic	C1C[N+]2(CCC1[C@H](C2)OC(=O)C(C3=CC=CS3)(C4=CC=CS4)O)CCCOC5=CC=CC=C5	484.7	0.46		112.2	+Q				
Aclidinium (S)	5.36	0.18	1.64	0.09	(Anti)cholinergic	C1C[N+]2(CCC1[C@H](C2)OC(=O)C(C3=CC=CS3)(C4=CC=CS4)O)CCCOC5=CC=CC=C5	484.7	0.46		112.2	+Q				
Aconitine	1.13	0.06	1.32	0.09	Herbal	CCN1C[C@@]2([C@@H](C[C@@H]([C@@]34[C@@H]2[C@H]([C@@H](C31)[C@@]5([C@@H]6[C@H]4C[C@@]([C@@H]6OC(=O)C7=CC=CC=C7)([C@H]([C@@H]5O)OC)O)OC(=O)C)OC)OC)O)COC	645.7	-2.14	8.99	153.5	+1				
Acyclovir	3.25	0.42	2.69	0.42	Antiviral	C1=NC2=C(N1COCCO)N=C(NC2=O)N	225.2	-1.55	2.97	114.8	0	1.40	0.02	1.39	0.58
Agmatine	1.08	0.18	1.05	0.13	Biogenic amine	C(CCN=C(N)N)CN	130.2	-5.94	12.69	90.4	+2				
Agomelatine	0.95	0.11	1.16	0.04	Antidepressant	CC(=O)NCCC1=CC=CC2=C1C=C(C=C2)OC	243.3	2.04		38.3	0	0.93	0.05	0.55	0.10
Alfuzosine	1.61	0.07	1.26	0.06	Alpha1 blocker	CN(CCCNC(=O)C1CCCO1)C2=NC3=CC(=C(C=C3C(=N2)N)OC)OC	389.5	0.43	8.10	111.8	0	0.86	0.11	1.04	0.18

Substance	MATE1 ratio	SEM	MATE2K ratio	SEM	Therapeutic group	SMILES code	Molecular weight	logD 7.4	most basic pKa	TPSA	Charge	TS DPH hCMEC	SEM	TS MDAI hCMEC	SEM
Aliskiren	1.81	0.14	2.00	0.23	Antihypertensive	CC(C)[C@@H](CC1=CC(=C(C=C1)OC)OCCCOC)C[C@@H]([C@H](C[C@@H](C(C)C)C(=O)NCC(C)(C)C(=O)N)O)N	551.8	1.00	9.57	146.1	+1				
Alizapride	3.68	0.16	2.64	0.22	Others	COC1=CC2=C(C=C1C(=O)NCC3CCCN3CC=C)NN=N2	315.4	0.26	8.58	83.1	+1				
Allopurinol	1.08	0.09	0.85	0.08	Others	C1=NNC2=C1C(=O)NC=N2	136.1	-0.90	2.02	70.1	0				
Almotriptan	4.40	0.25	2.36	0.17	Triptan	CN(C)CCC1=CNC2=C1C=C(C=C2)CS(=O)(=O)N3CCCC3	335.5	-0.63	9.57	64.8	+1				
Alpha-methylnorepinephrine (-)	1.85	0.23	0.72	0.09	Sympathomimetic	CC([C@@H](C1=CC(=C(C=C1)O)O)O)N	183.2	-1.55	8.93	86.7	+1				
Alprenolol	1.19	0.01	1.16	0.02	Beta blocker	CC(C)NCC(COC1=CC=CC=C1CC=C)O	249.4	0.84	9.27	41.5	+1				
Amantadine	1.42	0.13	1.48	0.12	Antiviral	C1C2CC3CC1CC(C2)(C3)N	151.3	-1.28	10.46	26.0	+1				
Ambroxol	1.00	0.17	1.32	0.55	Others	C1CC(CCC1NCC2=C(C(=CC(=C2)Br)Br)N)O	378.1	1.04	9.01	58.3	+1				
Amifampridine	17.06	0.85	7.01	1.41	Others	C1=CN=CC(=C1N)N	109.1	-1.84	9.25	64.9	+1				
Amiloride	11.06	1.14	6.80	0.30	Diuretic	C1(=C(N=C(C(=N1)Cl)N)N)C(=O)N=C(N)N	229.6	-1.14	7.29	159.3	+1				
Amiodarone	1.72	0.28	2.21	0.27	Antiarrhythmic	CCCCC1=C(C2=CC=CC=C2O1)C(=O)C3=CC(=C(C(=C3)I)OCCN(CC)CC)I	645.3	5.95	9.08	42.7	+1	0.49	0.09	0.82	0.08
Amisulpride	11.04	1.00	11.15	0.11	Antipsychotic	CCN1CCCC1CNC(=O)C2=CC(=C(C=C2OC)N)S(=O)(=O)CC	369.5	-0.68	8.28	110.1	+1	0.89	0.09	0.91	0.14
Amisulpride (R)	8.46	0.84	7.40	0.85	Antipsychotic	CCN1CCCC1CNC(=O)C2=CC(=C(C=C2OC)N)S(=O)(=O)CC	369.5	-0.68	8.28	110.1	+1				
Amisulpride (S)	13.70	1.98	7.93	0.46	Antipsychotic	CCN1CCCC1CNC(=O)C2=CC(=C(C=C2OC)N)S(=O)(=O)CC	369.5	-0.68	8.28	110.1	+1				
Amitriptyline	1.13	0.04	1.03	0.04	Antidepressant	CN(C)CCC=C1C2=CC=CC=C2CCC3=CC=CC=C31	277.4	3.15	9.06	3.2	+1	0.15	0.05	0.03	0.02

Substance	MATE1 ratio	SEM	MATE2K ratio	SEM	Therapeutic group	SMILES code	Molecular weight	logD 7.4	most basic pKa	TPSA	Charge	TS DPH hCMEC	SEM	TS MDAI hCMEC	SEM
Amlodipine	1.23	0.02	1.13	0.02	Antihypertensive	CCOC(=O)C1=C(NC(=C(C1C2=CC=CC=C2Cl)C(=O)OC)C)COCCN	408.9	-0.24	9.28	99.9	+1				
Amoxapine	1.32	0.04	1.33	0.08	Antidepressant	C1CN(CCN1)C2=NC3=CC=CC=C3OC4=C2C=C(C=C4)Cl	313.8	1.64	8.83	36.9	+1				
Amoxicillin	1.82	0.12	0.67	0.27	Antibiotic	CC1([C@@H](N2[C@H](S1)[C@@H](C2=O)NC(=O)[C@@H](C3=CC=C(C=C3)O)N)C(=O)O)C	365.4	-2.67	7.22	158.3	-1				
Amphetamine	0.96	0.13	1.68	0.51	Psychostimulant	CC(CC1=CC=CC=C1)N	135.2	-0.69	10.04	26.0	+1	0.05	0.00		
APC366	19.12	1.06	1.41	0.07	Others	C1C[C@H](N(C1)C(=O)[C@H](CCCN=C(N)N)NC(=O)C2=C(C3=CC=CC=C3C=C2)O)C(=O)N	440.5	-0.18	11.37	177.1	+1				
Apixaban	1.24	0.02	1.15	0.13	Anticoagulant	COC1=CC=C(C=C1)N2C3=C(CCN(C3=O)C4=CC=C(C=C4)N5CCCCC5=O)C(=N2)C(=O)N	459.5	1.83		110.8	0				
Apomorphine	1.05	0.13	1.02	0.11	Dopamine agonist	CN1CCC2=C3[C@H]1CC4=C(C3=CC=C2)C(=C(C=C4)O)O	267.3	2.59	7.73	43.7	+1				
Arcaine	40.20	2.16	16.29	0.23	Others	C(CCN=C(N)N)CN=C(N)N	172.2	-6.45	12.41	128.8	+2				
Aripiprazole	1.32	0.05	1.26	0.12	Antipsychotic	C1CC(=O)NC2=C1C=CC(=C2)OCCCCN3CCN(CC3)C4=C(C(=CC=C4)Cl)Cl	448.4	3.26	9.04	44.8	+1	0.72	0.05	0.68	0.21
Articaine	1.26	0.04	1.59	0.20	Local anesthetic	CCCNC(C)C(=O)NC1=C(SC=C1C)C(=O)OC	284.4	1.94	8.68	67.4	+1	0.53	0.07	0.47	0.12
Aspartame	1.26	0.24	1.07	0.18	Others	COC(=O)[C@H](CC1=CC=CC=C1)NC(=O)[C@H](CC(=O)O)N	294.3	-2.25	8.53	118.7	+1-1	1.77	0.05	1.65	0.27
Atenolol	18.66	0.70	4.56	1.12	Beta blocker	CC(C)NCC(COC1=CC=C(C=C1)CC(=O)N)O	266.3	-1.43	9.27	84.6	+1	1.32	0.16	1.22	0.11
Atenolol (R)	25.72	2.00	6.79	0.47	Beta blocker	CC(C)NCC(COC1=CC=C(C=C1)CC(=O)N)O	266.3	-1.43	9.27	84.6	+1				

Substance	MATE1 ratio	SEM	MATE2K ratio	SEM	Therapeutic group	SMILES code	Molecular weight	logD 7.4	most basic pKa	TPSA	Charge	TS DPH hCMEC	SEM	TS MDAI hCMEC	SEM
Atenolol (S)	25.17	1.04	9.28	1.22	Beta blocker	CC(C)NCC(COC1=CC=C(C=C1)CC(=O)N)O	266.3	-1.43	9.27	84.6	+1				
Atomoxetine	1.12	0.09	1.18	0.08	Antidepressant	CC1=CC=CC=C1O[C@H](CCNC)C2=CC=CC=C2	255.4	1.83	9.40	21.3	+1	0.23	0.01	0.25	0.01
Atomoxetine (S)	1.16	0.03	1.03	0.05	Antidepressant	CC1=CC=CC=C1O[C@H](CCNC)C2=CC=CC=C2	255.4	1.83	9.40	21.3	+1				
Atracurium	1.13	0.04	1.10	0.10	Muscle relaxant	C[N+]1(CCC2=CC(=C(C=C2C1CC3=CC(=C(C=C3)OC)OC)OC)OC)CCC(=O)OCCCCCOC(=O)CC[N+]4(CCC5=CC(=C(C=C5C4CC6=CC(=C(C=C6)OC)OC)OC)OC)C	929.2	-0.96		126.4	+Q2				
Atropine	2.27	0.85	1.67	0.29	(Anti)cholinergic	CN1[C@@H]2CC[C@H]1CC(C2)OC(=O)C(CO)C3=CC=CC=C3	289.4	-0.21	9.19	49.8	+1	0.80	0.24	0.64	0.03
Baclofen	1.45	0.28	1.05	0.03	Others	C1=CC(=CC=C1C(CC(=O)O)CN)Cl	213.7	-0.78	9.72	63.3	+1-1				
Bambuterol	1.52	0.11	1.21	0.08	Sympathomimetic	CC(C)(C)NCC(C1=CC(=CC(=C1)OC(=O)N(C)C)OC(=O)N(C)C)O	367.4	-0.70	9.52	91.3	+1				
Baricitinib	0.82	0.15	1.04	0.18	JAK inhibitor	CCS(=O)(=O)N1CC(C1)(CC#N)N2C=C(C=N2)C3=C4C=CNC4=NC=N3	371.4	-0.19	5.01	128.9	0				
Befunolol	1.39	0.02	1.38	0.13	Beta blocker	CC(C)NCC(COC1=CC=CC2=C1OC(=C2)C(=O)C)O	291.3	-0.61	9.26	71.7	+1				
Benzyltriethylammonium	25.63	0.13	4.52	0.09	Experimental chemical	CC[N+](CC)(CC)CC1=CC=CC=C1	192.3	-1.18		0.0	+Q				
Berberine	8.83	0.12	4.40	0.25	Herbal	COC1=C(C2=C[N+]3=C(C=C2C=C1)C4=CC5=C(C=C4CC3)OCO5)OC	336.4	-1.28		40.8	+Q				
Betahistine	1.90	0.06	1.19	0.11	Antihistaminic	CNCCC1=CC=CC=N1	136.2	-1.68	9.77	24.9	+1	0.73	0.08	0.86	0.22
Betaine	0.80	0.05	1.07	0.07	Endobiotic	C[N+](C)(C)CC(=O)[O-]	117.2	-3.72		40.1	+1-1	0.98	0.10	1.09	0.23

Substance	MATE1 ratio	SEM	MATE2K ratio	SEM	Therapeutic group	SMILES code	Molecular weight	logD 7.4	most basic pKa	TPSA	Charge	TS DPH hCMEC	SEM	TS MDAI hCMEC	SEM
Betaxolol	1.37	0.09	1.43	0.28	Beta blocker	CC(C)NCC(COC1=CC=C(C=C1)CCOCC2CC2)O	307.4	0.68	9.27	50.7	+1				
Bethanechol	0.23	0.01	0.97	0.05	(Anti)cholinergic	CC(C[N+](C)(C)C)OC(=O)N	161.2	-4.14		52.3	+Q				
Bicifadine	1.17	0.09	1.31	0.02	Antidepressant	CC1=CC=C(C=C1)C23CC2CNC3	173.3	-0.11	9.60	12.0	+1				
Biotin	1.24	0.18	1.18	0.06	Vitamin	C1[C@H]2[C@@H]([C@@H](S1)CCCCC(=O)O)NC(=O)N2	244.3	-2.57		103.7	-1				
Biperiden	1.24	0.06	1.15	0.02	(Anti)cholinergic	C1CCN(CC1)CCC(C2CC3CC2C=C3)(C4=CC=CC=C4)O	311.5	1.52	9.43	23.5	+1	0.25	0.02	0.26	0.03
Bisnorephedrine	1.42	0.15	1.30	0.10	Sympathomimetic	C1=CC=C(C=C1)C(CN)O	137.2	-1.22	9.10	46.3	+1				
Bisnorephedrine (R)	1.45	0.05	1.16	0.05	Experimental chemical	C1=CC=C(C=C1)C(CN)O	137.2	-1.22	9.10	46.3	+1				
Bisoprolol	1.23	0.04	1.05	0.06	Beta blocker	CC(C)NCC(COC1=CC=C(C=C1)COCCOC(C)C)O	325.4	0.34	9.27	60.0	+1	0.32	0.05	0.35	0.06
Bitolterol	0.38	0.19	0.57	0.16	Beta blocker	CC1=CC=C(C=C1)C(=O)OC2=C(C=C(C=C2)C(CNC(C)(C)C)O)OC(=O)C3=CC=C(C=C3)C	461.6	4.15	9.59	84.9	+1				
Bortezomib	1.20	0.04	1.10	0.04	Oncology	B([C@H](CC(C)C)NC(=O)[C@H](CC1=CC=CC=C1)NC(=O)C2=NC=CN=C2)(O)O	384.2	1.51	0.70	124.4	0				
Brofaromine	1.34	0.06	1.11	0.07	MAO inhibitor	COC1=CC(=C2C(=C1)C=C(O2)C3CCNCC3)Br	310.2	0.33	9.82	34.4	+1				
Bromocriptine	1.58	0.08	1.48	0.05	Dopamine agonist	CC(C)C[C@H]1C(=O)N2CCC[C@H]2[C@]3(N1C(=O)[C@](O3)(C(C)C)NC(=O)[C@H]4CN([C@@H]5CC6=C(NC7=CC=CC(=C67)C5=C4)Br)C)O	654.6	3.74	7.00	118.2	0	1.00	0.07	0.77	0.13
Brucine	1.13	0.08	1.30	0.06	Herbal	COC1=C(C=C2C(=C1)[C@]34CCN5[C@H]3C[C@@H]6[C@@H]7[C@@H]4N	394.5	-0.56	8.55	51.2	+1				

Substance	MATE1 ratio	SEM	MATE2K ratio	SEM	Therapeutic group	SMILES code	Molecular weight	logD 7.4	most basic pKa	TPSA	Charge	TS DPH hCMEC	SEM	TS MDAI hCMEC	SEM
						2C(=O)C[C@@H]7OCC=C6C5)OC									
Buformin	24.80	2.33	2.37	0.16	Antidiabetic	CCCCN=C(N)N=C(N)N	157.2	-4.31	12.49	102.8	+2				
Bumetanide	2.02	0.22	1.18	0.17	Diuretic	CCCCNC1=C(C(=CC(=C1)C(=O)O)S(=O)(=O)N)OC2=CC=CC=C2	364.4	-0.67	1.84	127.1	-1				
Bunitrolol	1.17	0.03	1.31	0.05	Beta blocker	CC(C)(C)NCC(COC1=CC=CC=C1C#N)O	248.3	-0.21	9.36	65.3	+1				
Bupivacaine	1.15	0.08	1.32	0.07	Local anesthetic	CCCCN1CCCCC1C(=O)NC2=C(C=CC=C2C)C	288.4	3.83	7.98	32.3	+1	0.63	0.04	0.67	0.07
Bupivacaine (R)	1.18	0.03	1.31	0.15	Local anesthetic	CCCCN1CCCCC1C(=O)NC2=C(C=CC=C2C)C	288.4	3.83	7.98	32.3	+1	0.59	0.07	0.42	0.15
Bupivacaine (S)	1.00	0.03	1.08	0.08	Local anesthetic	CCCCN1CCCCC1C(=O)NC2=C(C=CC=C2C)C	288.4	3.83	7.98	32.3	+1	0.43	0.01	0.24	0.05
Bupropion	1.23	0.05	1.03	0.08	Antidepressant	CC(C(=O)C1=CC(=CC=C1)Cl)NC(C)(C)C	239.7	2.39	8.22	29.1	+1	0.25	0.07	0.09	0.05
Buspirone	2.36	0.66	1.36	0.06	Others	C1CCC2(C1)CC(=O)N(C(=O)C2)CCCCN3CCN(CC3)C4=NC=CC=N4	385.5	0.61	8.54	69.6	+1	0.56	0.06	0.64	0.08
Butylscopolamine	23.84	1.72	1.70	0.09	(Anti)cholinergic	CCCC[N+]1([C@@H]2CC(C[C@H]1[C@H]3[C@@H]2O3)OC(=O)[C@H](CO)C4=CC=CC=C4)C.[Br-]	360.5	-1.94		59.1	+Q				
Butyrylcarnitine	0.82	0.12	1.09	0.10	Carnitine ester	CCCC(=O)O[C@H](CC(=O)[O-])C[N+](C)(C)C	231.3	-2.53		66.4	+1-1				
Cabergoline	1.20	0.03	1.13	0.09	Dopamine agonist	CCNC(=O)N(CCCN(C)C)C(=O)[C@@H]1C[C@H]2[C@@H](CC3=CNC4=CC=CC2=C34)N(C1)CC=C	451.6	0.28	9.31	71.7	+2	0.38	0.07	0.44	0.08
Cadaverine	0.23	0.04	0.53	0.04	Biogenic amine	C(CCN)CCN	102.2	-5.61	10.51	52.0	+2				

Substance	MATE1 ratio	SEM	MATE2K ratio	SEM	Therapeutic group	SMILES code	Molecular weight	logD 7.4	most basic pKa	TPSA	Charge	TS DPH hCMEC	SEM	TS MDAI hCMEC	SEM
Caffeine	1.25	0.18	0.92	0.12	Xanthine derivative	CN1C=NC2=C1C(=O)N(C(=O)N2C)C	194.2	-0.55		58.4	0				
Capsaicin	1.18	0.16	0.99	0.12	Herbal	CC(C)/C=C/CCCCC(=O)NCC1=CC(=C(C=C1)O)OC	305.4	3.75	0.37	58.6	0				
Captopril	1.11	0.16	1.22	0.23	Antihypertensive	C[C@H](CS)C(=O)N1CCC[C@H]1C(=O)O	217.3	-2.64	0.88	96.4	-1				
Carnitine	1.16	0.00	1.13	0.05	Endobiotic	C[N+](C)(C)C[C@@H](CC(=O)[O-])O	161.2	-4.12		60.4	+1-1				
Carnosine	0.18	0.02	1.03	0.06	Endobiotic	C1=C(NC=N1)C[C@@H](C(=O)O)NC(=O)CCN	226.2	-4.51	9.13	121.1	+1				
Carteolol	7.43	0.77	6.67	0.61	Beta blocker	CC(C)(C)NCC(COC1=CC=CC2=C1CCC(=O)N2)O	292.4	-0.52	9.36	70.6	+1				
Carvedilol (R)	1.17	0.04	1.08	0.03	Beta blocker	COC1=CC=CC=C1OCCNCC(COC2=CC=CC3=C2C4=CC=CC=C4N3)O	406.5	2.92	7.74	75.7	+1				
Carvedilol (S)	1.19	0.06	1.18	0.02	Beta blocker	COC1=CC=CC=C1OCCNCC(COC2=CC=CC3=C2C4=CC=CC=C4N3)O	406.5	2.92	7.74	75.7	+1				
Cathine	1.37	0.07	1.11	0.02	Psychostimulant	C[C@@H]([C@H](C1=CC=CC=C1)O)N	151.2	-1.03	9.35	46.3	+1	0.35	0.03		
Cathinone	1.14	0.08	1.31	0.08	Psychostimulant	C[C@@H](C(=O)C1=CC=CC=C1)N	149.2	0.83	7.48	43.1	+1				
Celiprolol	11.01	0.41	8.76	1.20	Beta blocker	CCN(CC)C(=O)NC1=CC(=C(C=C1)OCC(CNC(C)(C)C)O)C(=O)C	379.5	-0.43	9.35	90.9	+1				
Cetirizine	1.25	0.08	1.19	0.07	Antihistaminic	C1CN(CCN1CCOCC(=O)O)C(C2=CC=CC=C2)C3=CC=C(C=C3)Cl	388.9	0.78	7.97	53.0	+1-1	0.85	0.01	0.99	0.11
Cetirizine (L)	1.26	0.03	0.98	0.12	Antihistaminic	C1CN(CCN1CCOCC(=O)O)C(C2=CC=CC=C2)C3=CC=C(C=C3)Cl	388.9	0.78	7.97	53.0	+1-1	0.91	0.06	1.10	0.12
Cevimeline	1.59	0.33	1.35	0.35	(Anti)cholinergic	C[C@@H]1O[C@]2(CN3CCC2CC3)CS1	199.3	-0.22	8.59	37.8	+1				
Chlomipramine	1.30	0.20	1.09	0.09	Antidepressant	CN(C)CCCN1C2=CC=CC=C2CCC3=C1C=C(C=C3)Cl	314.9	3.09	9.20	6.5	+1				

Substance	MATE1 ratio	SEM	MATE2K ratio	SEM	Therapeutic group	SMILES code	Molecular weight	logD 7.4	most basic pKa	TPSA	Charge	TS DPH hCMEC	SEM	TS MDAI hCMEC	SEM
Chloramphenicol	1.04	0.01	1.03	0.08	Antibiotic	C1=CC(=CC=C1[C@H]([C@@H](CO)NC(=O)C(Cl)Cl)O)[N+](=O)[O-]	323.1	0.88		112.7	+1-1				
Chlorhexidine	1.08	0.24	0.99	0.15	Antiinfective	C1=CC(=CC=C1N/C(=N/C(=NCCCCCCN=C(/N=C(/NC2=CC=C(C=C2)Cl)\N)N)N)/N)Cl	505.5	-1.33	11.97	177.6	+2				
Chloroquine	1.40	0.14	1.06	0.14	Antiinfective	CCN(CC)CCCC(C)NC1=C2C=CC(=CC2=NC=C1)Cl	319.9	0.47	10.92	28.2	+1				
Chlorpheniramine	1.10	0.13	1.45	0.16	Antihistaminic	CN(C)CCC(C1=CC=C(C=C1)Cl)C2=CC=CC=N2	274.8	1.52	9.47	16.1	+1				
Chlorpromazine	1.42	0.11	1.33	0.02	Antipsychotic	CN(C)CCCN1C2=CC=CC=C2SC3=C1C=C(C=C3)Cl	318.9	2.74	9.20	31.8	+1				
Chlorprothixene	1.36	0.11	1.16	0.01	Antipsychotic	CN(C)CC/C=C\1/C2=CC=CC=C2SC3=C1C=C(C=C3)Cl	315.9	3.40	9.06	28.5	+1				
Choline	1.13	0.05	1.09	0.05	Endobiotic	C[N+](C)(C)CCO	104.2	-4.66		20.2	+Q	1.04	0.09	1.27	0.16
Cilastatin	1.13	0.25	0.96	0.17	Others	CC1(C[C@@H]1C(=O)N/C(=C\CCCCSC[C@@H](C(=O)O)N)/C(=O)O)C	358.5	-4.06	9.14	155.0	+1-2				
Cimetidine	11.52	1.73	3.60	0.19	Antihistaminic	CC1=C(N=CN1)CSCCNC(=NC)NC#N	252.3	-0.41	7.11	114.2	0				
Cinchonidine	1.16	0.04	1.14	0.09	Herbal	C=C[C@H]1CN2CC[C@H]1C[C@H]2[C@@H](C3=CC=NC4=CC=CC=C34)O	294.4	0.92	9.15	36.4	+1				
Ciprofloxacin	5.40	0.62	1.94	0.27	Antibiotic	C1CC1N2C=C(C(=O)C3=CC(=C(C=C32)N4CCNCC4)F)C(=O)O	331.3	-0.76	8.69	72.9	+1-1				
Citalopram	1.40	0.15	1.46	0.08	Antidepressant	CN(C)CCCC1(C2=C(CO1)C=C(C=C2)C#N)C3=CC=C(C=C3)F	324.4	0.89	10.38	36.3	+1	0.16	0.04	0.10	0.01
Citalopram (R)	1.33	0.04	1.17	0.06	Antidepressant	CN(C)CCCC1(C2=C(CO1)C=C(C=C2)C#N)C3=CC=C(C=C3)F	324.4	0.89	10.38	36.3	+1				

Substance	MATE1 ratio	SEM	MATE2K ratio	SEM	Therapeutic group	SMILES code	Molecular weight	logD 7.4	most basic pKa	TPSA	Charge	TS DPH hCMEC	SEM	TS MDAI hCMEC	SEM
Citalopram (S)	1.26	0.13	1.27	0.05	Antidepressant	CN(C)CCCC1(C2=C(CO1)C=C(C=C2)C#N)C3=CC=C(C=C3)F	324.4	0.89	10.38	36.3	+1	0.24	0.05	0.13	0.04
Citrulline	0.77	0.06	1.26	0.04	Endobiotic	C(C[C@@H](C(=O)O)N)CNC(=O)N	175.2	-3.94	9.23	118.4	+1-1				
Clarithromycin	1.21	0.04	1.31	0.04	Antibiotic	CC[C@@H]1[C@@]([C@@H]([C@H](C(=O)[C@@H](C[C@@]([C@@H]([C@H]([C@@H]([C@H](C(=O)O1)C)O[C@H]2C[C@@]([C@H]([C@@H](O2)C)O)(C)OC)C)O[C@H]3[C@@H]([C@H](C[C@H](O3)C)N(C)C)O)(C)OC)C)C)O)(C)O	748.0	1.63	9.00	182.9	+1				
Clemastine	1.26	0.06	1.21	0.01	Antihistaminic	C[C@@](C1=CC=CC=C1)(C2=CC=C(C=C2)Cl)OCC[C@H]3CCCN3C	343.9	2.79	9.55	12.5	+1	0.39	0.03	0.47	0.06
Clenbuterol	1.11	0.08	1.18	0.09	Symphatomimetic	CC(C)(C)NCC(C1=CC(=C(C(=C1)Cl)N)Cl)O	277.2	0.14	9.63	58.3	+1	0.36	0.09	0.07	0.01
Clidinium	16.18	0.79	1.79	0.21	(Anti)cholinergic	C[N+]12CCC(CC1)C(C2)OC(=O)C(C3=CC=CC=C3)(C4=CC=CC=C4)O	352.5	-1.08		46.5	+Q				
Clindamycin	1.19	0.02	1.22	0.01	Antibiotic	CCC[C@@H]1C[C@H](N(C1)C)C(=O)N[C@@H]([C@@H]2[C@@H]([C@@H]([C@H]([C@H](O2)SC)O)O)O)[C@H](C)Cl	425.0	0.65	7.55	127.6	+1				
Clonidine	1.11	0.11	1.17	0.23	Alpha2 agonist	C1CN=C(N1)NC2=C(C=CC=C2Cl)Cl	230.1	0.16	10.47	36.4	+1				
Clozapine	1.12	0.03	1.32	0.17	Antipsychotic	CN1CCN(CC1)C2=NC3=C(C=CC(=C3)Cl)NC4=CC=CC=C42	326.8	2.57	8.16	30.9	+1	0.16	0.04	0.03	0.01
Cocaine	1.34	0.14	1.60	0.03	Psychostimulant	CN1[C@H]2CC[C@@H]1[C@H]([C@H](C2)OC(=O)C3=CC=CC=C3)C(=O)OC	303.4	0.73	8.95	55.8	+1	0.16	0.02		

Substance	MATE1 ratio	SEM	MATE2K ratio	SEM	Therapeutic group	SMILES code	Molecular weight	logD 7.4	most basic pKa	TPSA	Charge	TS DPH hCMEC	SEM	TS MDAI hCMEC	SEM
Codeine	1.12	0.09	1.21	0.05	Opioid	CN1CC[C@]23[C@@H]4[C@H]1CC5=C2C(=C(C=C5)OC)O[C@H]3[C@H](C=C4)O	299.4	-0.16	8.89	41.9	+1				
Colterol	10.79	1.25	5.80	0.15	Beta blocker	CC(C)(C)NCC(C1=CC(=C(C=C1)O)O)O	225.3	-0.78	8.98	72.7	+1				
Coluracetam	0.97	0.03	0.97	0.04	Others	CC1=C(OC2=NC3=C(CCCC3)C(=C12)NC(=O)CN4CCCC4=O)C	341.4	1.90	1.23	75.4	0				
Coptisine	9.90	0.69	6.48	0.57	Herbal	C1C[N+]2=C(C=C3C=CC4=C(C3=C2)OCO4)C5=CC6=C(C=C51)OCO6	320.3	-1.34		40.8	+Q				
Corticosterone	1.18	0.03	1.05	0.03	Steroid hormone	C[C@]12CCC(=O)C=C1CC[C@@H]3[C@@H]2[C@H](C[C@]4([C@H]3CC[C@@H]4C(=O)CO)C)O	346.5	2.02		74.6	0				
Cotinine	1.11	0.17	1.20	0.24	Herbal	CN1[C@@H](CCC1=O)C2=CN=CC=C2	176.2	0.21	4.79	33.2	0				
Creatine	1.01	0.04	1.07	0.02	Endobiotic	CN(CC(=O)O)C(=N)N	131.1	-2.86	12.98	90.4	+1-1				
Creatinine	0.83	0.05	1.07	0.07	Endobiotic	CN1CC(=O)N=C1N	113.1	-1.98	4.96	58.7	0				
Crizotinib	1.09	0.11	1.04	0.03	Oncology	C[C@H](C1=C(C=CC(=C1Cl)F)Cl)OC2=C(N=CC(=C2)C3=CN(N=C3)C4CCNCC4)N	450.3	1.00	10.06	78.0	+1				
Crotaline	1.15	0.02	1.16	0.09	Herbal	C[C@H]1C(=O)O[C@@H]2CCN3[C@@H]2C(=CC3)COC(=O)[C@]([C@]1(C)O)(C)O	325.4	-0.37	6.38	96.3	+1				
Cycloguanil	21.02	0.83	10.75	0.33	Antiinfective	CC1(N=C(N=C(N1C2=CC=C(C=C2)Cl)N)N)C	251.7	-0.28	10.65	80.0	+1				
Cyclopentolate	1.09	0.05	1.34	0.05	(Anti)cholinergic	CN(C)CCOC(=O)C(C1=CC=CC=C1)C2(CCCC2)O	291.4	1.26	8.42	49.8	+1				
Cyclophosphamide	1.07	0.07	1.15	0.08	Cytostatic	C1CNP(=O)(OC1)N(CCCl)CCCl	261.1	0.10		51.4	0				
Cycloserine (D)	1.19	0.13	1.14	0.06	Antibiotic	C1[C@H](C(=O)NO1)N	102.1	-2.42	8.21	64.4	+1-1	1.25	0.23	0.79	0.05

Substance	MATE1 ratio	SEM	MATE2K ratio	SEM	Therapeutic group	SMILES code	Molecular weight	logD 7.4	most basic pKa	TPSA	Charge	TS DPH hCMEC	SEM	TS MDAI hCMEC	SEM
Cycloserine (L)	1.19	0.10	1.10	0.02	Antibiotic	C1[C@H](C(=O)NO1)N	102.1	-2.42	8.21	64.4	+1-1	1.34	0.24	0.76	0.04
Cytarabine	0.89	0.02	1.04	0.05	Cytostatic	C1=CN(C(=O)N=C1N)[C@H]2[C@H]([C@@H]([C@H](O2)CO)O)O	243.2	-2.80	3.59	128.6	0				
Cytisine	1.95	0.12	1.17	0.01	Herbal	C1[C@H]2CNC[C@@H]1C3=CC=CC(=O)N3C2	190.3	-2.64	9.82	32.3	+1				
Dapsone	0.89	0.04	0.95	0.07	Antiinfective	C1=CC(=CC=C1N)S(=O)(=O)C2=CC=C(C=C2)N	248.3	1.27	2.39	94.6	0				
Daunorubicin	1.21	0.22	1.01	0.07	Cytostatic	C[C@H]1[C@H]([C@H](C[C@@H](O1)O[C@H]2C[C@@](CC3=C2C(=C4C(=C3O)C(=O)C5=C(C4=O)C(=CC=C5)OC)O)(C(=O)C)O)N)O	527.5	0.37	10.93	185.8	+1	0.86	0.04	1.29	0.14
Debrisoquine	8.16	0.33	5.05	0.96	Antihypertensive	C1CN(CC2=CC=CC=C21)C(=N)N	175.2	-1.35	13.02	53.1	+1				
Decanoylcarnitine	2.74	0.21	0.96	0.07	Carnitine ester	CCCCCCCCCC(=O)O[C@H](CC(=O)[O-])C[N+](C)(C)C	315.5	0.14		66.4	+1-1				
Decitabine	1.13	0.14	1.04	0.22	Cytostatic	C1[C@@H]([C@H](O[C@H]1N2C=NC(=NC2=O)N)CO)O	228.2	-2.16	0.75	120.7	0				
Dehydrocorydaline	9.25	0.20	2.85	0.09	Herbal	CC1=C2C=CC(=C(C2=C[N+]3=C1C4=CC(=C(C=C4CC3)OC)OC)OC)OC	366.4	-0.71		40.8	+Q				
Delavirdine	1.19	0.27	1.09	0.14	Virostatic	CC(C)NC1=C(N=CC=C1)N2CCN(CC2)C(=O)C3=CC4=C(N3)C=CC(=C4)NS(=O)(=O)C	456.6	0.88	6.80	118.8	0				
Denatonium	11.37	1.26	4.15	0.32	Others	CC[N+](CC)(CC1=CC=CC=C1)CC(=O)NC2=C(C=CC=C2C)C	325.5	0.41		29.1	+Q				
Denopamine (R)	2.32	0.06	1.69	0.03	Sympathomimetic	COC1=C(C=C(C=C1)CCNC[C@@H](C2=CC=C(C=C2)O)O)OC	317.4	0.17	9.84	71.0	+1				
Deoxyepinephrine	34.31	5.75	3.40	0.31	Sympathomimetic	[2H]C([2H])([2H])NCCC1=CC(=C(C=C1)O)O	170.2	-1.27	9.35	52.5	+1				

Substance	MATE1 ratio	SEM	MATE2K ratio	SEM	Therapeutic group	SMILES code	Molecular weight	logD 7.4	most basic pKa	TPSA	Charge	TS DPH hCMEC	SEM	TS MDAI hCMEC	SEM
Desipramine	1.27	0.06	1.19	0.05	Antidepressant	CNCCCN1C2=CC=CC=C2CCC3=CC=CC=C31	266.4	1.37	10.02	15.3	+1	0.18	0.06	0.10	0.04
Desvenlafaxine	1.12	0.03	1.09	0.05	Antidepressant	CN(C)CC(C1=CC=C(C=C1)O)C2(CCCCC2)O	263.4	0.98	8.90	43.7	+1	0.49	0.05	0.61	0.20
Dextromethorphan	1.04	0.02	1.14	0.10	Opioid	CN1CC[C@@]23CCCC[C@@H]2[C@@H]1CC4=C3C=C(C=C4)OC	271.4	1.08	9.85	12.5	+1				
Diclofenac	1.00	0.02	0.73	0.01	Analgesic	C1=CC=C(C(=C1)CC(=O)O)NC2=C(C=CC=C2Cl)Cl	296.2	1.10		49.3	-1				
Diethyltryptamine	1.10	0.06	1.30	0.08	Psychedelic	CCN(CC)CCC1=CNC2=CC=CC=C21	216.3	0.37	10.11	19.0	+1	0.20	0.03		
Dihydroergotamine	1.19	0.04	1.19	0.01	Others	C[C@@]1(C(=O)N2[C@H](C(=O)N3CCC[C@H]3[C@@]2(O1)O)CC4=CC=CC=C4)NC(=O)[C@@H]5C[C@H]6[C@@H](CC7=CNC8=CC=CC6=C78)N(C5)C	583.7	2.22	7.96	118.2	+1				
Diltiazem	1.20	0.10	1.24	0.10	Antihypertensive	CC(=O)O[C@@H]1[C@@H](SC2=CC=CC=C2N(C1=O)CCN(C)C)C3=CC=C(C=C3)OC	414.5	1.89	8.18	84.4	+1				
Dimenhydrinate	1.20	0.10	1.43	0.09	Antihistaminic	CN1C2=C(C(=O)N(C1=O)C)NC(=N2)Cl.CN(C)CCOC(C1=CC=CC=C1)C2=CC=CC=C2	470.0	2.17	8.87	12.5	+1			0.36	0.07
Dimethindene	0.99	0.09	1.02	0.09	Antihistaminic	CC(C1=CC=CC=N1)C2=C(CC3=CC=CC=C32)CCN(C)C	292.4	1.46	9.70	16.1	+1				
Dimethylphenylpiperazinium	29.06	2.09	4.30	0.40	Experimental chemical	C[N+]1(CCN(CC1)C2=CC=CC=C2)C	191.3	-2.23		3.2	+Q				
Dimethyltryptamine	0.99	0.09	1.28	0.09	Psychedelic	CN(C)CCC1=CNC2=CC=CC=C21	188.3	0.15	9.57	19.0	+1	0.08	0.02		
Diphenhydramine	1.04	0.14	1.24	0.10	Antihistaminic	CN(C)CCOC(C1=CC=CC=C1)C2=CC=CC=C2	255.4	2.17	8.87	12.5	+1				
Dipyridamole	1.11	0.06	1.13	0.07	Anticoagulant	C1CCN(CC1)C2=NC(=NC3=C2N=C(N=C3N4CCCCC4)N(C	504.6	1.71	6.79	145.4	0				

Substance	MATE1 ratio	SEM	MATE2K ratio	SEM	Therapeutic group	SMILES code	Molecular weight	logD 7.4	most basic pKa	TPSA	Charge	TS DPH hCMEC	SEM	TS MDAI hCMEC	SEM
						CO)CCO)N(CCO)CCO									
Disopyramide	1.26	0.22	1.21	0.04	Antiarrhythmic	CC(C)N(CCC(C1=CC=CC=C1)(C2=CC=CC=N2)C(=O)N)C(C)C	339.5	0.57	10.42	59.2	+1				
Dobutamine	17.41	3.87	2.77	0.37	Sympathomimetic	CC(CCC1=CC=C(C=C1)O)NCCC2=CC(=C(C=C2)O)O	301.4	1.13	9.35	72.7	+1	0.67	0.08	0.99	0.34
Dofetilide	4.66	0.13	4.15	0.13	Antiarrhythmic	CN(CCC1=CC=C(C=C1)NS(=O)(=O)C)CCOC2=CC=C(C=C2)NS(=O)(=O)C	441.6	-1.01	9.26	121.6	+1				
Domperidone	1.27	0.24	1.05	0.29	Antiemetic	C1CN(CCC1N2C3=C(C=C(C=C3)Cl)NC2=O)CCCN4C5=CC=CC=C5NC4=O	425.9	2.18	8.03	67.9	+1				
Donepezil	1.18	0.02	1.19	0.04	Others	COC1=C(C=C2C(=C1)CC(C2=O)CC3CCN(CC3)CC4=CC=CC=C4)OC	379.5	2.48	9.12	38.8	+1				
Dopamine	39.94	0.88	1.97	0.40	Biogenic amine	C1=CC(=C(C=C1CCN)O)O	153.2	-1.41	9.31	66.5	+1				
Doxazosin	1.26	0.01	1.09	0.04	Alpha1 blocker	COC1=C(C=C2C(=C1)C(=NC(=N2)N3CCN(CC3)C(=O)C4COC5=CC=CC=C5O4)N)OC	451.5	1.43	8.04	112.3	+1				
Doxepin	1.34	0.12	1.32	0.02	Antidepressant	CN(C)CC/C=C/1\C2=CC=CC=C2COC3=CC=CC=C31	279.4	2.18	9.06	12.5	+1	0.13	0.03	0.10	0.01
Doxepin (E)	1.26	0.04	1.08	0.07	Antidepressant	CN(C)CC/C=C/1\C2=CC=CC=C2COC3=CC=CC=C31	279.4	2.18	9.06	12.5	+1				
Doxepin (Z)	1.28	0.05	1.09	0.06	Antidepressant	CN(C)CC/C=C/1\C2=CC=CC=C2COC3=CC=CC=C31	279.4	2.18	9.06	12.5	+1				
Doxycycline	1.32	0.09	1.32	0.18	Antibiotic	C[C@@H]1[C@H]2[C@@H]([C@H]3[C@@H](C(=O)C(=C([C@]3(C(=O)C2=C(C4=C1C=CC=C4O)O)O)O)C(=O)N)N(C)C)O	444.4	-5.88	5.98	181.6	-1	1.45	0.36	1.45	0.37

Substance	MATE1 ratio	SEM	MATE2K ratio	SEM	Therapeutic group	SMILES code	Molecular weight	logD 7.4	most basic pKa	TPSA	Charge	TS DPH hCMEC	SEM	TS MDAI hCMEC	SEM
Doxylamine	1.17	0.08	1.27	0.03	Antihistaminic	CC(C1=CC=CC=C1)(C2=CC=CC=N2)OCCN(C)C	270.4	1.48	8.87	25.4	+1	0.42	0.05	0.46	0.11
Doxylamine (R)	1.23	0.10	1.50	0.07	Antihistaminic	CC(C1=CC=CC=C1)(C2=CC=CC=N2)OCCN(C)C	270.4	1.48	8.87	25.4	+1				
Doxylamine (S)	1.12	0.11	1.58	0.13	Antihistaminic	CC(C1=CC=CC=C1)(C2=CC=CC=N2)OCCN(C)C	270.4	1.48	8.87	25.4	+1				
Dropropizine	1.25	0.04	1.45	0.18	Others	C1CN(CCN1CC(CO)O)C2=CC=CC=C2	236.2	-0.31	8.27	46.9	+1				
Dropropizine (D)	1.12	0.14	1.39	0.09	Others	C1CN(CCN1CC(CO)O)C2=CC=CC=C2	236.2	-0.31	8.27	46.9	+1				
Dropropizine (L)	1.14	0.04	1.37	0.06	Others	C1CN(CCN1CC(CO)O)C2=CC=CC=C2	236.2	-0.31	8.27	46.9	+1				
Duloxetine	1.25	0.10	0.99	0.10	Antidepressant	CNCC[C@@H](C1=CC=CS1)OC2=CC=CC3=CC=CC=C32	297.4	2.31	9.30	49.5	+1	0.10	0.01	0.10	0.05
Duloxetine (R)	1.23	0.03	1.07	0.03	Antidepressant	CNCC[C@@H](C1=CC=CS1)OC2=CC=CC3=CC=CC=C32	297.4	2.31	9.30	49.5	+1				
Duloxetine (S)	1.23	0.04	1.06	0.04	Antidepressant	CNCC[C@@H](C1=CC=CS1)OC2=CC=CC3=CC=CC=C32	297.4	2.31	9.30	49.5	+1				
Edrophonium	30.33	3.68	10.98	1.29	Others	CC[N+](C)(C)C1=CC(=CC=C1)O	166.2	-0.96		20.2	+Q				
Efaroxan	1.38	0.07	1.72	0.23	Alpha2 antagonist	CCC1(CC2=CC=CC=C2O1)C3=NCCN3	216.3	0.50	9.06	33.6	+1				
Eletriptan	1.41	0.10	1.22	0.09	Triptan	CN1CCC[C@@H]1CC2=CNC3=C2C=C(C=C3)CCS(=O)(=O)C4=CC=CC=C4	382.5	1.58	9.62	61.6	+1	0.21	0.03	0.46	0.05
Emtricitabine	3.22	0.16	2.88	0.15	Virostatic	C1[C@H](O[C@H](S1)CO)N2C=C(C(=NC2=O)N)F	247.2	-0.90	1.14	113.5	0				
Endoxifen	1.30	0.18	1.21	0.14	Oncology	CC/C(=C(\C1=CC=C(C=C1)O)/C2=CC=C(C=C2)OCCNC)/C3=CC=CC=C3	373.5	4.15	8.78	41.5	+1				
Entecavir	0.96	0.09	1.07	0.09	Virostatic	C=C1[C@H](C[C@@H]([C@H]1CO)O)N2C=NC3=C2N=C(NC3=O)N	277.3	-1.96	3.08	125.8	0				
Ephedrine	1.42	0.11	1.29	0.05	Sympathomimetic	C[C@@H]([C@@H](C1=CC=CC=C1)O)NC	165.2	-0.78	9.52	32.3	+1	0.36	0.02	0.24	0.09

Substance	MATE1 ratio	SEM	MATE2K ratio	SEM	Therapeutic group	SMILES code	Molecular weight	logD 7.4	most basic pKa	TPSA	Charge	TS DPH hCMEC	SEM	TS MDAI hCMEC	SEM
Epiberberine	12.54	0.46	5.89	0.46	Herbal	COC1=C(C=C2C(=C1)CC[N+]3=C2C=C4C=CC5=C(C4=C3)OCO5)OC	336.4	-1.28		40.8	+Q				
Epinephrine (-)	37.78	4.79	3.98	1.00	Sympathomimetic	CNC[C@@H](C1=CC(=C(C=C1)O)O)O	183.2	-1.63	8.91	72.7	+1				
Epinephrine (rac)	18.70	2.49	3.27	0.57	Sympathomimetic	CNC[C@@H](C1=CC(=C(C=C1)O)O)O	183.2	-1.63	8.91	72.7	+1				
Ergometrine	1.41	0.15	1.16	0.07	Herbal	C[C@@H](CO)NC(=O)[C@H]1CN([C@@H]2CC3=CNC4=CC=CC(=C34)C2=C1)C	325.4	0.93	6.98	68.4	0				
Ergothioneine (L)	0.98	0.25	1.16	0.41	Endobiotic	C[N+](C)(C)[C@@H](CC1=CNC(=S)N1)C(=O)[O-]	229.3	-3.26		96.3	+1-1				
Esmolol	1.42	0.06	1.23	0.13	Beta blocker	CC(C)NCC(COC1=CC=C(C=C1)CCC(=O)OC)O	295.4	-0.03	9.27	67.8	+1				
Estrone-3-sulfate	4.80	0.41	2.08	0.37	Steroid hormone	C[C@]12CC[C@H]3[C@H]([C@@H]1CCC2=O)CCC4=C3C=CC(=C4)OS(=O)(=O)O	350.4	1.46		89.1	-1				
Ethambutol	14.14	2.57	2.78	0.16	Antibiotic	CC[C@@H](CO)NCCN[C@@H](CC)CO	204.3	-2.25	9.55	64.5	+1	0.98	0.27	0.95	0.06
Etilefrine	9.81	0.86	2.04	0.25	Sympathomimetic	CCNCC(C1=CC(=CC=C1)O)O	181.2	-1.07	9.73	52.5	+1				
Etomidate	1.17	0.14	1.26	0.16	Others	CCOC(=O)C1=CN=CN1[C@H](C)C2=CC=CC=C2	244.3	2.50	4.82	44.1	0	0.85	0.09	0.87	0.08
Famotidine	23.82	2.01	11.08	0.76	Antihistaminic	C1=C(N=C(S1)N=C(N)N)CSCC/C(=N/S(=O)(=O)N)/N	337.4	-2.84	8.44	237.8	+1				
Fampridine (4-aminopyridine)	1.94	0.23	1.23	0.08	Others	C1=CN=CC=C1N	94.1	-0.97	8.95	38.9	+1				
Fasudil	1.50	0.12	1.80	0.13	Others	C1CNCCN(C1)S(=O)(=O)C2=CC=CC3=C2C=CN=C3	291.4	-0.40	8.04	70.7	+1				
Fedratinib	1.16	0.05	0.87	0.04	Oncology	CC1=CN=C(N=C1NC2=CC(=CC=C2)S(=O)(=O)NC(C)(C)C)NC3=CC=C(C=C3)OCCN4CCCC4	524.7	3.27	8.97	116.9	+1				

Substance	MATE1 ratio	SEM	MATE2K ratio	SEM	Therapeutic group	SMILES code	Molecular weight	logD 7.4	most basic pKa	TPSA	Charge	TS DPH hCMEC	SEM	TS MDAI hCMEC	SEM
Felbamate	0.97	0.06	0.94	0.07	Antiepileptic	C1=CC=C(C=C1)C(COC(=O)N)COC(=O)N	238.2	0.68		104.6	0				
Fenfluramine (R)	1.08	0.07	2.47	0.30	Others	CCNC(C)CC1=CC(=CC=C1)C(F)(F)F	231.3	0.79	10.22	12.0	+1				
Fenfluramine (S)	0.80	0.10	1.82	0.30	Others	CCNC(C)CC1=CC(=CC=C1)C(F)(F)F	231.3	0.79	10.22	12.0	+1				
Fenoldopam	8.11	1.30	4.17	0.84	Dopamine agonist	C1CNCC(C2=CC(=C(C(=C21)Cl)O)O)C3=CC=C(C=C3)O	305.8	0.92	10.30	72.7	+1				
Fenoterol	14.98	0.65	5.46	0.40	Sympathomimetic	CC(CC1=CC=C(C=C1)O)NCC(C2=CC(=CC(=C2)O)O)O	303.4	0.34	10.09	93.0	+1	1.47	0.24	1.16	0.13
Fenpiverinium	15.19	1.98	1.35	0.16	(Anti)cholinergic	C[N+]1(CCCCC1)CCC(C2=CC=CC=C2)(C3=CC=CC=C3)C(=O)N	337.5	-0.56		43.1	+Q				
Fentanyl	1.27	0.09	1.25	0.04	Opioid	CCC(=O)N(C1CCN(CC1)CCC2=CC=CC=C2)C3=CC=CC=C3	336.5	2.71	8.47	23.6	+1				
Fesoterodine	1.09	0.03	1.02	0.10	(Anti)cholinergic	CC(C)C(=O)OC1=C(C=C(C=C1)CO)[C@H](CCN(C(C)C)C(C)C)C2=CC=CC=C2	411.6	2.65	10.64	49.8	+1	0.40	0.03	0.41	0.06
Fexofenadine	1.63	0.36	1.30	0.44	Antihistaminic	CC(C)(C1=CC=C(C=C1)C(CCCN2CCC(CC2)C(C3=CC=CC=C3)(C4=CC=CC=C4)O)O)C(=O)O	501.7	2.94	9.21	81.0	+1-1	1.27	0.19	1.50	0.14
Filgotinib	0.89	0.04	1.01	0.05	JAK inhibitor	C1CC1C(=O)NC2=NN3C(=N2)C=CC=C3C4=CC=C(C=C4)CN5CCS(=O)(=O)CC5	425.5	2.03	2.62	105.1	0				
Fingolimod	1.12	0.02	1.14	0.03	Others	CCCCCCCCC1=CC=C(C=C1)CCC(CO)(CO)N	307.5	2.12	9.38	66.5	+1				
Flecainide	1.13	0.04	1.19	0.09	Antiarrhythmic	C1CCNC(C1)CNC(=O)C2=C(C=CC(=C2)OCC(F)(F)F)OCC(F)(F)F	414.3	1.01	9.62	59.6	+1				
Flunarizine	1.23	0.16	1.10	0.09	Calcium antagonist	C1CN(CCN1C/C=C/C2=CC=CC=C2)C(C3=CC=C(C=C3)F)C4=CC=C(C=C4)F	404.5	5.82	7.49	6.5	+1				

Substance	MATE1 ratio	SEM	MATE2K ratio	SEM	Therapeutic group	SMILES code	Molecular weight	logD 7.4	most basic pKa	TPSA	Charge	TS DPH hCMEC	SEM	TS MDAI hCMEC	SEM
Fluoxetine	1.30	0.07	1.16	0.05	Antidepressant	CNCCC(C1=CC=CC=C1)OC2=CC=C(C=C2)C(F)(F)F	309.3	2.19	9.40	21.3	+1	0.10	0.02	0.13	0.07
Fluoxetine (R)	1.23	0.08	1.05	0.06	Antidepressant	CNCCC(C1=CC=CC=C1)OC2=CC=C(C=C2)C(F)(F)F	309.3	2.19	9.40	21.3	+1				
Fluoxetine (S)	1.18	0.06	1.12	0.03	Antidepressant	CNCCC(C1=CC=CC=C1)OC2=CC=C(C=C2)C(F)(F)F	309.3	2.19	9.40	21.3	+1				
Fluphenazine	1.26	0.10	1.13	0.02	Antipsychotic	C1CN(CCN1CCCN2C3=CC=CC=C3SC4=C2C=C(C=C4)C(F)(F)F)CCO	437.5	2.73	8.61	55.3	+1				
Fluvoxamine	1.59	0.15	1.68	0.13	Antidepressant	COCCCC/C(=N\OCCN)/C1=CC=C(C=C1)C(F)(F)F	318.3	1.42	8.76	56.8	+1				
Folic acid	1.00	0.11	1.13	0.22	Vitamin	C1=CC(=CC=C1C(=O)N[C@@H](CCC(=O)O)C(=O)O)NCC2=CN=C3C(=N2)C(=O)NC(=N3)N	441.4	-6.61	2.82	209.0	-2				
Formoterol	3.64	0.49	1.38	0.13	Sympathomimetic	CC(CC1=CC=C(C=C1)OC)NCC(C2=CC(=C(C=C2)O)NC=O)O	344.4	0.04	9.81	90.8	+1	0.87	0.16	1.10	0.12
Formoterol (R)	5.18	0.32	1.72	0.10	Sympathomimetic	C[C@H](CC1=CC=C(C=C1)OC)NC[C@@H](C2=CC(=C(C=C2)O)NC=O)O	344.4	0.04	9.81	90.8	+1				
Frovatriptan	34.35	1.44	31.50	2.85	Triptan	CN[C@@H]1CCC2=C(C1)C3=C(N2)C=CC(=C3)C(=O)N	243.3	-1.74	10.42	70.9	+1	0.83	0.25	0.88	0.08
Furosemide	1.63	0.23	1.25	0.22	Diuretic	C1=COC(=C1)CNC2=CC(=C(C=C2C(=O)O)S(=O)(=O)N)Cl	330.7	-1.63		131.0	-1				
Gabapentin	0.75	0.10	1.04	0.02	Antiepileptic	C1CCC(CC1)(CC(=O)O)CN	171.2	-1.27	9.91	63.3	+1-1	1.18	0.16	1.31	0.00
Gabexate	5.78	0.26	4.18	0.11	Anticoagulant	CCOC(=O)C1=CC=C(C=C1)OC(=O)CCCCCN=C(N)N	321.4	-0.37	12.21	117.0	+1				
Galantamine	1.18	0.10	1.23	0.15	Cholinesterase inhibitor	CN1CC[C@@]23C=C[C@@H](C[C@@H]2OC4=C(C=CC(=C34)C1)OC)O	287.4	0.76	7.58	41.9	+1	0.80	0.03	0.98	0.00
Gamma-aminobutyric acid	0.90	0.15	0.87	0.06	Endobiotic	C(CC(=O)O)CN	103.1	-2.89	10.22	63.3	+1-1				

Substance	MATE1 ratio	SEM	MATE2K ratio	SEM	Therapeutic group	SMILES code	Molecular weight	logD 7.4	most basic pKa	TPSA	Charge	TS DPH hCMEC	SEM	TS MDAI hCMEC	SEM
Ganciclovir	1.38	0.30	1.68	0.11	Virostatic	C1=NC2=C(N1COC(CO)CO)N=C(NC2=O)N	255.2	-2.18	2.88	135.0	0				
Gefitinib	0.89	0.05	0.88	0.10	Oncology	COC1=C(C=C2C(=C1)N=CN=C2NC3=CC(=C(C=C3)F)Cl)OCCCN4CCOCC4	446.9	3.52	7.25	68.7	0				
Gemcitabine	1.29	0.18	1.08	0.20	Cytostatic	C1=CN(C(=O)N=C1N)[C@H]2C([C@@H]([C@H](O2)CO)O)(F)F	263.2	-1.47	3.05	108.4	0				
Gentian violet	1.40	0.15	1.05	0.12	Experimental chemical	CN(C)C1=CC=C(C=C1)C(=C2C=CC(=[N+](C)C)C=C2)C3=CC=C(C=C3)N(C)C	372.5	1.39	4.83	9.5	+Q				
Glutamic acid (D)	1.17	0.12	0.99	0.22	Amino acid	C(CC(=O)O)[C@@H](C(=O)O)N	147.1	-6.17	9.54	100.6	+1-2				
Glycopyrrolate	10.59	2.06	1.18	0.18	(Anti)cholinergic	C[N+]1(CCC(C1)OC(=O)C(C2CCCC2)(C3=CC=CC=C3)O)C	318.4	-1.41		46.5	+Q				
Granisetron	1.22	0.05	1.25	0.12	5HT3 Antagonist	CN1[C@@H]2CCC[C@H]1CC(C2)NC(=O)C3=NN(C4=CC=CC=C43)C	312.4	0.56	8.70	50.2	+1	0.43	0.04	0.28	0.03
Guanethidine	10.31	0.85	2.49	0.13	Antihypertensive	C1CCCN(CCC1)CCN=C(N)N	198.3	-4.70	12.35	67.6	+2				
Guanfacine	1.60	0.17	1.37	0.08	Antihypertensive	C1=CC(=C(C(=C1)Cl)CC(=O)N=C(N)N)Cl	246.1	0.43	8.30	81.5	+1				
Haloperidol	1.23	0.01	1.15	0.04	Antipsychotic	C1CN(CCC1(C2=CC=C(C=C2)Cl)O)CCCC(=O)C3=CC=C(C=C3)F	375.9	2.85	8.14	40.5	+1	0.57	0.08	0.51	0.07
Halostachine	1.41	0.08	1.52	0.18	Herbal	CNC[C@@H](C1=CC=CC=C1)O	151.2	-1.11	9.43	32.3	+1				
Harmaline	1.19	0.02	1.15	0.04	Herbal	CC1=NCCC2=C1NC3=C2C=CC(=C3)OC	214.3	1.41	7.32	37.4	+1				
Heptylamine	1.18	0.06	1.60	0.21	Experimental chemical	CCCCCCCN	115.2	-0.57	10.21	26.0	+1				
Hexanoylcarnitine	1.02	0.05	1.01	0.06	Carnitine ester	CCCCCC(=O)OC(CC(=O)[O-])C[N+](C)(C)C	259.3	-1.64		66.4	+1-1				
Hexylamine	1.21	0.23	1.08	0.06	Experimental chemical	CCCCCCN	101.2	-1.02	10.21	26.0	+1				

Substance	MATE1 ratio	SEM	MATE2K ratio	SEM	Therapeutic group	SMILES code	Molecular weight	logD 7.4	most basic pKa	TPSA	Charge	TS DPH hCMEC	SEM	TS MDAI hCMEC	SEM
Higenamine	14.55	0.70	11.27	0.44	Sympathomimetic	C1CNC(C2=CC(=C(C=C21)O)O)CC3=CC=C(C=C3)O	271.3	1.41	8.57	72.7	+1				
Histamine	19.90	1.62	1.40	0.13	Biogenic amine	C1=C(NC=N1)CCN	111.1	-3.44	9.74	54.7	+1				
Homoarginine	0.97	0.02	0.97	0.03	Amino acid	C(CCN=C(N)N)C[C@@H](C(=O)O)N	188.2	-4.76	12.39	127.7	+2-1				
Hordenine	1.16	0.07	1.20	0.05	Sympathomimetic	CN(C)CCC1=CC=C(C=C1)O	165.2	0.11	9.08	23.5	+1				
Hydrochlorothiazide	2.15	0.12	2.50	0.36	Diuretic	C1NC2=CC(=C(C=C2S(=O)(=O)N1)S(=O)(=O)N)Cl	297.7	-0.58		135.1	0				
Hydrocodone	1.12	0.04	1.24	0.01	Opioid	CN1CC[C@]23[C@@H]4[C@H]1CC5=C2C(=C(C=C5)OC)O[C@H]3C(=O)CC4	299.4	0.48	8.87	38.8	+1	1.35	0.89	0.46	0.10
Hydromorphone	1.22	0.02	1.17	0.04	Opioid	CN1CC[C@]23[C@@H]4[C@H]1CC5=C2C(=C(C=C5)O)O[C@H]3C(=O)CC4	285.3	0.33	9.42	49.8	+1	0.76	0.04	0.79	0.16
Hydroxybenzylamine	1.48	0.16	1.09	0.06	Herbal	C1=CC(=CC=C1CN)O	123.2	-1.05	9.06	46.3	+1				
Hydroxybupropion	1.09	0.19	1.51	0.20	Antidepressant	CC(C(=O)C1=CC(=CC=C1)Cl)NC(C)(C)CO	255.7	1.78	7.65	49.3	+1				
Hydroxychloroquine (R)	2.22	0.19	1.62	0.03	Antiinfective	CCN(CCCC(C)NC1=C2C=CC(=CC2=NC=C1)Cl)CCO	335.9	0.31	9.76	48.4	+1				
Hydroxychloroquine (S)	2.05	0.06	1.45	0.10	Antiinfective	CCN(CCCC(C)NC1=C2C=CC(=CC2=NC=C1)Cl)CCO	335.9	0.31	9.76	48.4	+1				
Hypaphorine	1.23	0.13	1.03	0.11	Herbal	C[N+](C)(C)[C@@H](CC1=CNC2=CC=CC=C21)C(=O)[O-]	246.3	-1.40		55.9	+1-1				
Ibogaine	1.16	0.06	1.14	0.02	Psychedelic	CC[C@H]1C[C@@H]2C[C@@H]3[C@H]1N(C2)CCC4=C3NC5=C4C=C(C=C5)OC	310.4	1.96	8.97	28.3	+1				
Ibutilide	1.57	0.32	1.21	0.07	Antiarrhythmic	CCCCCCCN(CC)CCCC(C1=CC=C(C=C1)NS(=O)(=O)C)O	384.6	0.64	10.85	78.0	+1				
Ifosfamide	1.24	0.12	1.17	0.15	Oncology	C1CN(P(=O)(OC1)NCCCl)CCCl	261.1	0.10		51.4	0				
Imatinib	1.28	0.16	1.07	0.11	Oncology	CC1=C(C=C(C=C1)NC(=O)C2=CC=C(C	493.6	3.52	8.20	86.3	+1				

Substance	MATE1 ratio	SEM	MATE2K ratio	SEM	Therapeutic group	SMILES code	Molecular weight	logD 7.4	most basic pKa	TPSA	Charge	TS DPH hCMEC	SEM	TS MDAI hCMEC	SEM
						=C2)CN3CCN(CC3)C)NC4=NC=CC(=N4)C5=CN=CC=C5									
Imeglimin	18.76	0.92	4.59	0.26	Antidiabetic	C[C@@H]1N=C(NC(=N1)N(C)C)N	155.2	-4.29	11.60	66.0	+1				
Imipramine	1.26	0.09	1.12	0.04	Antidepressant	CN(C)CCCN1C2=CC=CC=C2CCC3=CC=CC=C31	280.4	2.48	9.20	6.5	+1	0.13	0.01	0.01	0.00
Indacaterol	1.98	0.03	1.88	0.28	Sympathomimetic	CCC1=C(C=C2CC(CC2=C1)NC[C@@H](C3=C4C=CC(=O)NC4=C(C=C3)O)O)CC	392.5	2.32	9.71	81.6	+1				
Ipratropium	24.31	1.93	6.36	1.53	(Anti)cholinergic	CC(C)[N+]1([C@@H]2CC[C@H]1CC(C2)OC(=O)C(CO)C3=CC=CC=C3)C	332.5	-1.82		46.5	+Q	0.77	0.19	0.93	0.10
Irinotecan	1.69	0.09	1.11	0.18	Cytostatic	CCC1=C2CN3C(=CC4=C(C3=O)COC(=O)[C@@]4(CC)O)C2=NC5=C1C=C(C=C5)OC(=O)N6CCC(CC6)N7CCCCC7	586.7	-0.32	9.18	112.5	+1-1				
Isobutyrylcarnitine	1.40	0.06	1.35	0.18	Carnitine ester	CC(C)C(=O)O[C@H](CC(=O)[O-])C[N+](C)(C)C	231.3	-2.43		66.4	+1-1				
Isoetharine	5.65	0.23	1.85	0.31	Sympathomimetic	CCC(C(C1=CC(=C(C=C1)O)O)O)NC(C)C	239.2	-0.22	9.01	72.7	+1				
Isoniazid	0.96	0.05	0.98	0.09	Antibiotic	C1=CN=CC=C1C(=O)NN	137.1	-0.69	3.35	68.0	0	0.96	0.05	1.51	0.24
Isoprenaline	16.78	1.60	4.67	0.71	Sympathomimetic	CC(C)NCC(C1=CC(=C(C=C1)O)O)O	211.3	-0.99	8.96	72.7	+1				
Isovalerylcarnitine	0.91	0.08	1.11	0.09	Carnitine ester	CC(C)CC(=O)OC(CC(=O)[O-])C[N+](C)(C)C	245.3	-2.24		66.4	+1-1				
Ivabradine	1.14	0.05	1.23	0.08	Others	CN(CCCN1CCC2=CC(=C(C=C2CC1=O)OC)OC)C[C@H]3CC4=CC(=C(C=C34)OC)OC	468.6	0.83	9.30	60.5	+1				
Ketamine	1.27	0.13	1.12	0.08	Psychedelic	CNC1(CCCCC1=O)C2=CC=CC=C2Cl	237.7	3.10	7.29	29.1	+1	0.62	0.06	0.47	0.01
Kynurenine	0.84	0.03	1.16	0.04	Endobiotic	C1=CC=C(C(=C1)C(=O)CC(C(=O)O)N)N	208.2	-1.92	8.96	106.4	+1-1				

Substance	MATE1 ratio	SEM	MATE2K ratio	SEM	Therapeutic group	SMILES code	Molecular weight	logD 7.4	most basic pKa	TPSA	Charge	TS DPH hCMEC	SEM	TS MDAI hCMEC	SEM
Labetalol	1.43	0.04	1.38	0.05	Beta blocker	CC(CCC1=CC=CC=C1)NCC(C2=CC(=C(C=C2)O)C(=O)N)O	328.4	1.26	9.80	95.6	+1	0.40	0.03	0.57	0.10
Lafutidine	1.41	0.11	1.48	0.11	Antihistaminic	C1CCN(CC1)CC2=CC(=NC=C2)OC/C=C\CNC(=O)CS(=O)CC3=CC=CO3	431.6	0.51	7.94	103.9	+1				
Lamivudine	11.10	1.48	3.36	0.56	Virostatic	C1[C@H](O[C@H](S1)CO)N2C=CC(=NC2=O)N	229.3	-1.10	3.70	113.5	0				
Lamotrigine	1.46	0.17	1.52	0.09	Antiepileptic	C1=CC(=C(C(=C1)Cl)Cl)C2=C(N=C(N=N2)N)N	256.1	1.93	4.85	90.7	0				
Landiolol	3.68	0.17	2.87	0.25	Beta blocker	CC1(OC[C@H](O1)COC(=O)CCC2=CC=C(C=C2)OC[C@H](CNCCNC(=O)N3CCOCC3)O)C	509.6	-0.68	8.39	127.8	+1				
Lenalidomide	1.78	0.28	1.53	0.32	Oncology	C1CC(=O)NC(=O)C1N2CC3=C(C2=O)C=CC=C3N	259.3	-0.71	2.32	92.5	0				
Leucine	1.18	0.08	1.06	0.15	Amino acid	CC(C)C[C@@H](C(=O)O)N	131.2	-1.59	9.52	63.3	+1-1				
Levetiracetam	1.19	0.04	1.28	0.04	Antiepileptic	CC[C@@H](C(=O)N)N1CCCC1=O	170.2	-0.59	0.86	63.4	0	1.17	0.16	1.19	0.14
Levodopa	0.71	0.09	1.01	0.10	Others	C1=CC(=C(C=C1C[C@@H](C(=O)O)N)O)O	197.2	-1.80	9.06	103.8	+1-1				
Levomepromazine	1.21	0.04	1.19	0.04	Antipsychotic	C[C@@H](CN1C2=CC=CC=C2SC3=C1C=C(C=C3)OC)CN(C)C	328.5	2.24	9.42	15.7	+1	0.31	0.07	0.33	0.08
Lidocaine	1.64	0.22	1.65	0.09	Local anesthetic	CCN(CC)CC(=O)NC1=C(C=CC=C1C)C	234.3	2.33	7.75	32.3	+1	0.88	0.06	0.69	0.02
Lysine	1.13	0.17	1.05	0.15	Amino acid	C(CCN)C[C@@H](C(=O)O)N	146.2	-4.98	10.29	89.3	+2-1				
Maprotiline	1.17	0.15	1.04	0.04	Antidepressant	CNCCCC12CCC(C3=CC=CC=C31)C4=CC=CC=C24	277.4	1.25	11.14	12.0	+1				
Maraviroc	1.58	0.08	1.44	0.03	Virostatic	CC1=NN=C(N1C2C[C@H]3CC[C@@H](C2)N3CC[C@@H](C4=CC=CC=C4)NC(=O)C5CCC(CC5)(F)F)C(C)C	513.7	1.99	9.03	63.1	+1	1.01	0.03	0.57	0.12

Substance	MATE1 ratio	SEM	MATE2K ratio	SEM	Therapeutic group	SMILES code	Molecular weight	logD 7.4	most basic pKa	TPSA	Charge	TS DPH hCMEC	SEM	TS MDAI hCMEC	SEM
Matrine	1.03	0.07	1.27	0.11	Others	C1C[C@@H]2[C@H]3CCCN4[C@H]3[C@@H](CCC4)CN2C(=O)C1	248.4	-1.25	9.75	23.6	+1				
Mecamylamine	1.20	0.19	1.05	0.04	(Anti)cholinergic	CC1(C2CCC(C2)C1(C)NC)C	167.3	-0.81	11.48	12.0	+1				
Medetomidine	1.11	0.08	1.21	0.08	Alpha2 agonist	CC1=C(C(=CC=C1)C(C)C2=CN=CN2)C	200.3	2.96	7.19	28.7	0	0.79	0.01	0.96	0.39
Memantine	1.03	0.08	1.18	0.07	Others	CC12CC3CC(C1)(CC(C3)(C2)N)C	179.3	-0.67	10.45	26.0	+1	0.24	0.01	0.14	0.03
Mepenzolate	23.89	2.23	1.72	0.10	(Anti)cholinergic	C[N+]1(CCCC(C1)OC(=O)C(C2=CC=CC=C2)(C3=CC=CC=C3)O)C	340.4	-0.97		46.5	+Q				
Mepivacaine	1.15	0.07	1.33	0.06	Local anesthetic	CC1=C(C(=CC=C1)C)NC(=O)C2CCCCN2C	246.4	2.97	7.23	32.3	+1	0.81	0.06	0.55	0.02
Meptazinol	1.21	0.01	1.57	0.11	Opioid	CCC1(CCCCN(C1)C)C2=CC(=CC=C2)O	233.4	1.55	9.16	23.5	+1	0.40	0.02	0.41	0.18
Meropenem	1.32	0.05	1.33	0.23	Antibiotic	C[C@@H]1[C@@H]2[C@H](C(=O)N2C(=C1S[C@H]3C[C@H](NC3)C(=O)N(C)C)C(=O)O)[C@@H](C)O	383.5	-4.39	8.39	135.5	+1-1	1.20	0.06	1.00	0.03
Mescaline	4.00	0.74	1.56	0.18	Psychedelic	COC1=CC(=CC(=C1OC)OC)CCN	211.3	-1.32	9.70	53.7	+1	0.41	0.02		
Metamfetamine	1.53	0.18	1.97	0.26	Psychostimulant	C[C@@H](CC1=CC=CC=C1)NC	149.2	-0.44	10.21	12.0	+1	0.05	0.00		
Metanephrine	4.97	0.93	1.71	0.26	Endobiotic	CNCC(C1=CC(=C(C=C1)O)OC)O	197.2	-1.52	9.25	61.7	+1				
Metformin	0.71	0.19	1.10	0.32	Others	CN(C)C(=N)N=C(N)N	129.2	-5.75	12.72	91.5	+2	0.79	0.09	1.10	0.08
Methacholine	21.58	1.43	2.57	0.24	(Anti)cholinergic	CC(C[N+](C)(C)C)OC(=O)C	160.2	-3.80		26.3	+Q				
Methoxytryptophan	0.89	0.02	1.10	0.03	Endobiotic	COC1=CC2=C(C=C1)NC=C2C[C@@H](C(=O)O)N	234.3	-1.25	9.42	88.3	+1-1				
Methyldopa	1.04	0.13	1.15	0.04	Antihypertensive	C[C@](CC1=CC(=C(C=C1)O)O)(C(=O)O)N	211.2	-1.37	9.85	103.8	+1-1				
Methylecgonine	1.54	0.12	1.36	0.20	Herbal	CN1C2CCC1C(C(C2)O)C(=O)OC	199.3	-1.96	9.15	49.8	+1				
Methylene blue	2.36	0.11	1.55	0.04	Experimental chemical	CN(C)C1=CC2=C(C=C1)N=C3C=CC(=[N+](C)C)C=C3S2	284.4	-0.62	3.14	43.9	+1				

Substance	MATE1 ratio	SEM	MATE2K ratio	SEM	Therapeutic group	SMILES code	Molecular weight	logD 7.4	most basic pKa	TPSA	Charge	TS DPH hCMEC	SEM	TS MDAI hCMEC	SEM
Methylenedioxyethamphetamine	1.07	0.07	1.39	0.11	Psychostimulant	CCNC(C)CC1=CC2=C(C=C1)OCO2	207.3	-0.46	10.22	30.5	+1				
Methylergometrine	1.08	0.10	0.99	0.16	Psychedelic	CC[C@@H](CO)NC(=O)[C@H]1CN([C@@H]2CC3=CNC4=CC=CC(=C34)C2=C1)C	339.4	1.45	6.98	68.4	0				
Methylnaltrexone	12.01	0.89	4.43	0.05	Opioid antagonist	C[N+]1(CC[C@]23[C@@H]4C(=O)CC[C@]2([C@H]1CC5=C3C(=C(C=C5)O)O4)O)CC6CC6	356.4	-1.80		66.8	+Q	1.11	0.00	0.84	0.34
Methylnicotinamide	33.89	0.19	5.40	0.90	Endobiotic	CNC(=O)C1=CN=CC=C1	136.2	-0.17	3.62	42.0	0				
Methylphenidate	1.16	0.21	1.40	0.29	Psychostimulant	COC(=O)C(C1CCCCN1)C2=CC=CC=C2	233.3	0.57	9.09	38.3	+1	0.33	0.03	0.24	0.05
Methylscopolamine	21.32	1.66	2.02	0.09	(Anti)cholinergic	C[N+]1([C@@H]2CC(C[C@H]1[C@H]3[C@@H]2O3)OC(=O)[C@H](CO)C4=CC=CC=C4)C	318.4	-3.27		59.1	+Q	0.84	0.20	1.02	0.12
Methysergide	1.06	0.05	1.20	0.06	Others	CC[C@@H](CO)NC(=O)[C@H]1CN([C@@H]2CC3=CN(C4=CC=CC(=C34)C2=C1)C)C	353.5	1.70	6.88	57.5	0				
Metipranolol	1.21	0.03	1.17	0.04	Beta blocker	CC1=CC(=C(C(=C1OC(=O)C)C)C)OCC(CNC(C)C)O	309.4	0.89	9.27	67.8	+1				
Metoclopramide	1.08	0.07	1.26	0.04	Others	CCN(CC)CCNC(=O)C1=CC(=C(C=C1OC)N)Cl	299.8	-0.25	9.04	67.6	+1	0.60	0.10	0.42	0.20
Metoprolol	1.15	0.26	1.09	0.28	Beta blocker	CC(C)NCC(COC1=CC=C(C=C1)CCOC)O	267.4	-0.10	9.27	50.7	+1	0.44	0.05	0.31	0.06
Mexiletine	1.34	0.30	1.51	0.28	Antiarrhythmic	CC1=C(C(=CC=C1)C)OCC(C)N	179.3	0.53	9.37	32.3	+1				
Mexiletine (R)	1.03	0.12	1.39	0.23	Antiarrhythmic	CC1=C(C(=CC=C1)C)OCC(C)N	179.3	0.53	9.37	32.3	+1				
Mexiletine (S)	1.14	0.20	1.61	0.27	Antiarrhythmic	CC1=C(C(=CC=C1)C)OCC(C)N	179.3	0.53	9.37	32.3	+1				
Miconazole	1.33	0.24	1.50	0.30	Antiinfective	C1=CC(=C(C=C1Cl)Cl)COC(CN2C=CN=C2)C3=C(C=C(C=C3)Cl)Cl	416.1	5.84	7.05	27.1	0				

Substance	MATE1 ratio	SEM	MATE2K ratio	SEM	Therapeutic group	SMILES code	Molecular weight	logD 7.4	most basic pKa	TPSA	Charge	TS DPH hCMEC	SEM	TS MDAI hCMEC	SEM
Midodrine	4.53	0.18	1.43	0.04	Sympathomimetic	COC1=CC(=C(C=C1)OC)C(CNC(=O)CN)O	254.3	-1.76	8.14	93.8	+1	1.05	0.24	1.02	0.23
Milnacipran	2.54	0.21	1.22	0.04	Antidepressant	CCN(CC)C(=O)[C@@]1(C[C@@H]1CN)C2=CC=CC=C2	246.4	-0.91	9.83	46.3	+1	0.53	0.12	0.84	0.02
Minocycline	1.15	0.04	1.11	0.01	Antibiotic	CN(C)[C@H]1[C@@H]2C[C@@H]3CC4=C(C=CC(=C4C(=C3C(=O)[C@@]2(C(=C(C1=O)C(=O)N)O)O)O)O)N(C)C	457.5	-4.76	7.64	164.6	+1-1				
Minoxidil	1.56	0.18	1.54	0.14	Antihypertensive	C1CCN(CC1)C2=NC(=N)N(C(=C2)N)O	209.3	-1.63	8.77	88.9	+1				
m-Iodobenzylguanidin	3.53	0.25	2.28	0.19	Others	C1=CC(=CC(=C1)I)CN=C(N)N	275.1	-0.77	12.18	64.4	+1				
Mirabegron	8.19	0.17	4.76	0.40	Sympathomimetic	C1=CC=C(C=C1)[C@H](CNCCC2=CC=C(C=C2)NC(=O)CC3=CSC(=N3)N)O	396.5	0.70	9.62	133.1	+1	0.90	0.06	0.75	0.11
Molsidomine	1.38	0.09	1.55	0.09	Others	CCO/C(=N/C1=C[N+](=NO1)N2CCOCC2)/[O-]	242.2	-0.55		87.0	+Q				
Moroxydine	32.69	2.10	1.51	0.02	Antiinfective	C1COCCN1C(=N)N=C(N)N	171.2	-5.12	12.43	100.7	+2				
Morphine	1.24	0.12	1.25	0.03	Opioid	CN1CC[C@]23[C@@H]4[C@H]1CC5=C2C(=C(C=C5)O)O[C@H]3[C@H](C=C4)O	285.3	-0.31	8.65	52.9	+1	0.97	0.06	0.82	0.19
Moxifloxacin	1.23	0.01	1.03	0.15	Antibiotic	COC1=C2C(=CC(=C1N3C[C@@H]4CCCN[C@@H]4C3)F)C(=O)C(=CN2C5CC5)C(=O)O	401.4	-0.49	9.43	82.1	+1-1	0.92	0.04	0.98	0.03
Moxonidine	2.02	0.09	1.78	0.14	Antihypertensive	CC1=NC(=C(C(=N1)Cl)NC2=NCCN2)OC	241.7	-0.53	9.68	71.4	+1				
m-Tyramine	8.90	0.33	1.31	0.06	Biogenic amine	C1=CC(=CC(=C1)O)CCN	137.2	-1.12	10.04	46.3	+1				
N-Acetyl-L-cysteine	1.02	0.12	0.95	0.10	Others	CC(=O)N[C@@H](CS)C(=O)O	163.2	-4.10	0.17	105.2	-1				
Nadolol	9.41	0.81	10.12	1.11	Beta blocker	CC(C)(C)NCC(COC1=CC=CC2=C1C[C@@H]([C@@H](C2)O)O)O	309.4	-1.08	9.36	82.0	+1				

Substance	MATE1 ratio	SEM	MATE2K ratio	SEM	Therapeutic group	SMILES code	Molecular weight	logD 7.4	most basic pKa	TPSA	Charge	TS DPH hCMEC	SEM	TS MDAI hCMEC	SEM
Nafamostat	5.45	0.50	1.64	0.14	Anticoagulant	C1=CC(=CC=C1C(=O)OC2=CC3=C(C=C2)C=C(C=C3)C(=N)N)N=C(N)N	347.4	-1.70	11.32	140.6	+2				
Nalbuphine	1.20	0.06	1.15	0.01	Opioid	C1CC(C1)CN2CC[C@]34[C@@H]5[C@H](CC[C@]3([C@H]2CC6=C4C(=C(C=C6)O)O5)O)O	357.5	-0.07	9.60	73.2	+1				
Nalmefene	1.07	0.08	1.16	0.11	Opioid antagonist	C=C1CC[C@]2([C@H]3CC4=C5[C@]2([C@H]1OC5=C(C=C4)O)CCN3CC6CC6)O	339.4	0.73	9.55	52.9	+1	0.58	0.05	0.44	0.08
Naloxone	1.36	0.14	1.29	0.07	Opioid antagonist	C=CCN1CC[C@]23[C@@H]4C(=O)CC[C@]2([C@H]1CC5=C3C(=C(C=C5)O)O4)O	327.4	1.13	7.68	70.0	+1	0.78	0.04	0.44	0.07
Naltrexone	1.10	0.11	1.33	0.12	Opioid antagonist	C1CC1CN2CC[C@]34[C@@H]5C(=O)CC[C@]3([C@H]2CC6=C4C(=C(C=C6)O)O5)O	341.4	0.27	8.54	70.0	+1	0.99	0.03	0.74	0.02
Naratriptan	10.81	0.19	8.34	0.27	Triptan	CNS(=O)(=O)CCC1=CC2=C(C=C1)NC=C2C3CCN(CC3)C	335.5	-0.32	9.16	73.6	+1				
N-Desmethylcitalopram	1.09	0.28	1.09	0.18	Antidepressant	CNCCCC1(C2=C(CO1)C=C(C=C2)C#N)C3=CC=C(C=C3)F	310.4	0.26	11.14	45.1	+1				
N-Desmethylranitidine	23.54	2.54	14.02	0.84	Antihistaminic	CNCC1=CC=C(O1)CSCCNC(=C[N+](=O)[O-])NC	300.4	-0.43	9.57	117.7	+2-1				
N-Desmethyltramadol	1.12	0.03	1.19	0.04	Opioid	CNCC1CCCCC1(C2=CC(=CC=C2)OC)O	249.4	-0.35	9.89	41.5	+1	0.37	0.06	0.64	0.06
N-Desmethylvenlafaxine	1.23	0.03	1.32	0.04	Antidepressant	CNCC(C1=CC=C(C=C1)OC)C2(CCCCC2)O	263.4	0.03	9.78	41.5	+1				
Nebivolol	1.41	0.08	1.45	0.05	Beta blocker	C1CC2=C(C=CC(=C2)F)OC1C(CNCC(C3CCC4=C(O3)C=CC(=C4)F)O)O	405.4	2.73	7.70	71.0	+1				
Nefazodone	1.34	0.06	1.24	0.05	Antidepressant	CCC1=NN(C(=O)N1CCOC2=CC=CC=C2)CCCN3CCN(CC3)C4=CC(=CC=C4)Cl	470.0	3.48	8.55	51.6	+1				

Substance	MATE1 ratio	SEM	MATE2K ratio	SEM	Therapeutic group	SMILES code	Molecular weight	logD 7.4	most basic pKa	TPSA	Charge	TS DPH hCMEC	SEM	TS MDAI hCMEC	SEM
Neostigmine	32.31	2.96	5.31	0.71	(Anti)cholinergic	CN(C)C(=O)OC1=CC=CC(=C1)[N+](C)(C)C	223.3	-2.25		29.5	+Q				
N-Ethyl-lidocaine	36.39	2.25	14.68	1.07	Local anesthetic	CC[N+](CC)(CC)CC(=O)NC1=C(C=CC=C1C)C	263.4	-0.96		29.1	+Q				
Niacin	1.06	0.04	0.90	0.01	Vitamin	C1=CC(=CN=C1)C(=O)O	123.1	-3.09	3.47	50.2	-1				
Nicotinamide	1.02	0.03	1.04	0.01	Vitamin	C1=CC(=CN=C1)C(=O)N	122.1	-0.39	3.63	56.0	0				
Nimodipine	1.33	0.23	1.20	0.26	Calcium antagonist	CC1=C(C(C(=C(N1)C)C(=O)OC(C)C)C2=CC(=CC=C2)[N+](=O)[O-])C(=O)OCCOC	418.4	2.54	4.31	117.0	+1-1	1.28	0.08	1.25	0.12
Nizatidine	11.88	1.51	10.42	1.53	Antihistaminic	CN/C(=C\[N+](=O)[O-])/NCCSCC1=CSC(=N1)CN(C)C	331.5	0.72	6.54	136.9	+1-1				
N-Methyl-2-phenylethylamine	1.27	0.17	1.50	0.23	Endobiotic	CNCCC1=CC=CC=C1	135.2	-0.79	10.13	12.0	+1				
N-Methyl-p-Tyramine	3.11	0.13	1.39	0.04	Herbal	CNCCC1=CC=C(C=C1)O	151.2	-1.04	10.39	32.3	+1				
N-Methylserotonin	26.34	1.30	8.85	0.78	Herbal	CNCCC1=CNC2=C1C=C(C=C2)O	190.2	-1.03	10.44	48.1	+1				
N-Methyltryptamine	1.31	0.09	1.34	0.08	Psychedelic	CNCCC1=CNC2=CC=CC=C21	174.2	-0.83	10.31	27.8	+1				
NN-Dimethylarginine	0.97	0.09	0.93	0.04	Endobiotic	CN(C)C(=NCCC[C@@H](C(=O)O)N)N	202.3	-4.35	12.89	104.9	+2-1				
Norepinephrine (L)	12.34	1.33	1.63	0.25	Sympathomimetic	C1=CC(=C(C=C1[C@H](CN)O)O)O	169.2	-1.76	8.81	86.7	+1				
Norepinephrine (S)	11.02	0.87	1.08	0.05	Sympathomimetic	C1=CC(=C(C=C1[C@H](CN)O)O)O	169.2	-1.76	8.81	86.7	+1				
Norepinephrine rac	11.70	1.21	1.63	0.29	Sympathomimetic	C1=CC(=C(C=C1[C@H](CN)O)O)O	169.2	-1.76	8.81	86.7	+1				
Norfenfluramine (+)	1.22	0.23	1.45	0.05	Sympathomimetic	CC(CC1=CC(=CC=C1)C(F)(F)F)N	203.2	0.26	9.94	26.0	+1				
Normetanephrine	1.80	0.24	0.82	0.11	Endobiotic	COC1=C(C=CC(=C1)C(CN)O)O	183.2	-1.64	9.00	75.7	+1				
Noroxycodone	1.39	0.08	1.13	0.03	Opioid	COC1=C2C3=C(C[C@@H]4[C@]5([C@]3(CCN4)[C@@H](O2)C(=O)CC5)O)C=C1	301.3	-1.20	9.26	67.8	+1				
Norphenylephrine	26.13	3.14	1.22	0.20	Sympathomimetic	C1=CC(=CC(=C1)O)C(CN)O	153.2	-1.45	8.86	66.5	+1				

Substance	MATE1 ratio	SEM	MATE2K ratio	SEM	Therapeutic group	SMILES code	Molecular weight	logD 7.4	most basic pKa	TPSA	Charge	TS DPH hCMEC	SEM	TS MDAI hCMEC	SEM
Nortilidine	1.19	0.05	1.42	0.10	Opioid	CCOC(=O)[C@]1(CCC=C[C@@H]1NC)C2=CC=CC=C2	259.3	1.49	8.87	38.3	+1	0.53	0.00	0.52	0.00
Nortriptyline	1.27	0.06	1.10	0.02	Antidepressant	CNCCC=C1C2=CC=CC=C2CCC3=CC=CC=C31	263.4	1.58	10.47	12.0	+1	0.23	0.04	0.12	0.03
Ochratoxin A	3.12	0.30	1.28	0.16	Herbal	C[C@@H]1CC2=C(C=C(C(=C2C(=O)O1)O)C(=O)N[C@@H](CC3=CC=CC=C3)C(=O)O)Cl	403.8	1.01		112.9	-1				
Octopamine	20.87	0.98	1.43	0.13	Sympathomimetic	C1=CC(=CC=C1C(CN)O)O	153.2	-1.49	8.93	66.5	+1				
Ofloxacin	1.53	0.16	1.41	0.06	Antibiotic	CC1COC2=C3N1C=C(C(=O)C3=CC(=C2N4CCN(CC4)C)F)C(=O)O	361.4	-0.82	8.31	73.3	+1-1	0.97	0.08	0.82	0.04
Ofloxacin (L)	1.93	0.09	1.41	0.04	Antibiotic	C[C@H]1COC2=C3N1C=C(C(=O)C3=CC(=C2N4CCN(CC4)C)F)C(=O)O	361.4	-0.82	8.31	73.3	+1-1	0.97	0.08	1.16	0.28
Olanzapine	1.23	0.07	1.25	0.07	Antipsychotic	CC1=CC2=C(S1)NC3=CC=CC=C3N=C2N4CCN(CC4)C	312.4	2.56	8.16	59.1	+1				
Olodaterol	3.34	0.12	6.19	0.23	Sympathomimetic	CC(C)(CC1=CC=C(C=C1)OC)NC[C@@H](C2=C3C(=CC(=C2)O)NC(=O)CO3)O	386.4	-0.18	9.84	100.1	+1				
Ondansetron	1.19	0.03	1.24	0.17	5HT3 Antagonist	CC1=NC=CN1CC2CCC3=C(C2=O)C4=CC=CC=C4N3C	293.4	1.83	7.91	39.8	+1	0.22	0.05	0.22	0.05
Orciprenaline	34.88	1.44	23.10	1.35	Sympathomimetic	CC(C)NCC(C1=CC(=CC(=C1)O)O)O	211.3	-0.94	9.70	72.7	+1				
Ornithine	0.74	0.20	1.09	0.03	Endobiotic	C(C[C@@H](C(=O)O)N)CN	132.2	-5.42	10.29	89.3	+2-1				
Orphenadrine	1.49	0.04	1.48	0.09	Antihistaminic	CC1=CC=CC=C1C(C2=CC=CC=C2)OCCN(C)C	269.4	2.69	8.87	12.5	+1				
Oseltamivir	1.18	0.03	1.04	0.00	Virostatic	CCC(CC)O[C@@H]1C=C(C[C@@H]([C@H]1NC(=O)C)N)C(=O)OCC	312.4	-0.67	9.26	90.7	+1				
Oxiconazole	1.39	0.21	1.30	0.21	Antiinfective	C1=CC(=C(C=C1Cl)Cl)CO/N=C(\CN2C=CN=C2)/C3=C(C=C(C=C3)Cl)Cl	429.1	5.66	7.31	39.4	0				

Substance	MATE1 ratio	SEM	MATE2K ratio	SEM	Therapeutic group	SMILES code	Molecular weight	logD 7.4	most basic pKa	TPSA	Charge	TS DPH hCMEC	SEM	TS MDAI hCMEC	SEM
Oxprenolol	1.05	0.11	1.20	0.06	Beta blocker	CC(C)NCC(COC1=CC=CC=C1OCC=C)O	265.4	0.31	9.27	50.7	+1				
Oxycodone	1.09	0.03	1.21	0.05	Opioid	CN1CC[C@]23[C@@H]4C(=O)CC[C@]2([C@H]1CC5=C3C(=C(C=C5)OC)O4)O	315.4	0.28	8.07	59.0	+1	0.57	0.10	0.39	0.04
Oxymetazoline	5.22	0.66	2.29	0.07	Sympathomimetic	CC1=CC(=C(C(=C1CC2=NCCN2)C)O)C(C)(C)C	260.4	1.20	10.15	44.6	+1				
Oxyphenonium	9.89	1.83	2.04	0.86	(Anti)cholinergic	CC[N+](C)(CC)CCOC(=O)C(C1CCCCC1)(C2=CC=CC=C2)O	348.5	-0.20		46.5	+Q				
Oxytetracycline	1.19	0.16	0.81	0.20	Antibiotic	C[C@@]1([C@H]2[C@@H]([C@H]3[C@@H](C(=O)C(=C([C@]3(C(=O)C2=C(C4=C1C=CC=C4O)O)O)O)C(=O)N)N(C)C)O)O	460.4	-7.30	5.98	201.9	-1				
Paliperidone	1.34	0.22	1.42	0.25	Antipsychotic	CC1=C(C(=O)N2CCCC(C2=N1)O)CCN3CCC(CC3)C4=NOC5=C4C=CC(=C5)F	426.5	0.38	8.76	82.2	+1				
Palmatine	12.74	0.56	2.99	0.09	Herbal	COC1=C(C2=C[N+]3=C(C=C2C=C1)C4=CC(=C(C=C4CC3)OC)OC)OC	352.4	-1.22		40.8	+Q				
Palonosetron	1.30	0.12	1.38	0.08	5HT3 Antagonist	C1C[C@@H]2CN(C(=O)C3=CC=CC(=C23)C1)[C@@H]4CN5CCC4CC5	296.4	1.35	8.57	23.6	+1	0.38	0.04	0.35	0.06
Palonosetron (R,R)	1.11	0.08	1.03	0.03	5HT3 Antagonist	C1C[C@@H]2CN(C(=O)C3=CC=CC(=C23)C1)[C@@H]4CN5CCC4CC5	296.4	1.35	8.57	23.6	+1				
Palonosetron (S,S)	1.35	0.09	1.16	0.07	5HT3 Antagonist	C1C[C@@H]2CN(C(=O)C3=CC=CC(=C23)C1)[C@@H]4CN5CCC4CC5	296.4	1.35	8.57	23.6	+1				
Pancuronium	1.46	0.22	0.95	0.07	Muscle relaxant	CC(=O)O[C@H]1C[C@@H]2CC[C@@H]3[C@@H]([C@]2(C[C@@H]1[N+]4(CCCCC4)C)C)CC[C@]5([C@H]3C[C@	572.9	-3.27		52.6	+2				

Substance	MATE1 ratio	SEM	MATE2K ratio	SEM	Therapeutic group	SMILES code	Molecular weight	logD 7.4	most basic pKa	TPSA	Charge	TS DPH hCMEC	SEM	TS MDAI hCMEC	SEM
						@H]([C@@H]5OC(=O)C)[N+]6(CCCCC6)C)C									
Pantothenic acid	1.48	0.09	1.71	0.30	Vitamin	CC(C)(CO)[C@H](C(=O)NCCC(=O)O)O	219.2	-4.28	0.70	106.9	-1				
Paracetamol	1.00	0.03	0.99	0.02	Analgesic	CC(=O)NC1=CC=C(C=C1)O	151.2	0.90		49.3	0				
para-Methoxyamphetamine	1.07	0.07	1.25	0.13	Psychostimulant	CC(CC1=CC=C(C=C1)OC)N	165.2	-0.81	9.99	35.3	+1	0.39	0.02		
para-Methoxy-N-methylamphetamine	1.17	0.16	1.52	0.07	Psychostimulant	CC(CC1=CC=C(C=C1)OC)NC	179.3	-0.59	10.20	21.3	+1	0.08	0.00		
Paraxanthine	1.00	0.07	0.90	0.10	Xanthine derivative	CN1C=NC2=C1C(=O)N(C(=O)N2)C	180.2	0.11		67.2	0	1.13	0.13	0.93	0.11
Paroxetine	1.92	0.46	1.79	0.46	Antidepressant	C1CNC[C@H]([C@@H]1C2=CC=C(C=C2)F)COC3=CC4=C(C=C3)OCO4	329.4	1.25	9.32	39.7	+1	0.13	0.00	0.07	0.02
Pentamidine	5.93	0.37	2.40	0.20	Antiinfective	C1=CC(=CC=C1C(=N)N)OCCCCCOC2=CC=C(C=C2)C(=N)N	340.4	-2.50	12.13	118.2	+2				
Pentobarbital	1.14	0.05	1.17	0.20	Antiepileptic	CCCC(C)C1(C(=O)NC(=O)NC1=O)CC	226.3	1.76		75.3	0				
Perphenazine	1.09	0.02	0.94	0.01	Antipsychotic	C1CN(CCN1CCCN2C3=CC=CC=C3SC4=C2C=C(C=C4)Cl)CCO	404.0	2.45	8.61	55.3	+1				
Pethidine	1.80	0.58	2.82	0.53	Opioid	CCOC(=O)C1(CCN(CC1)C)C2=CC=CC=C2	247.3	1.64	8.14	29.5	+1				
Phenelzine	1.58	0.29	1.91	0.23	MAO inhibitor	C1=CC=C(C=C1)CCNN	136.2	0.36	8.18	38.1	+1				
Phenformin	19.54	0.77	3.01	0.28	Antidiabetic	C1=CC=C(C=C1)CCN=C(N)N=C(N)N	205.3	-3.55	12.31	102.8	+2				
Phentermine	1.20	0.11	1.76	0.14	Psychostimulant	CC(C)(CC1=CC=CC=C1)N	149.2	-0.56	10.27	26.0	+1	0.02	0.01		
Phenylalanine	0.66	0.00	1.01	0.04	Amino acid	C1=CC=C(C=C1)C[C@@H](C(=O)O)N	165.2	-1.19	9.45	63.3	+1-1				
Phenylephrine	13.78	1.04	1.75	0.02	Symphatomimetic	CNC[C@@H](C1=CC(=CC=C1)O)O	167.2	-1.35	9.69	52.5	+1	0.76	0.06	1.03	0.13
Phenylephrine (L)	18.71	0.16	2.15	0.18	Sympathomimetic	CNC[C@@H](C1=CC(=CC=C1)O)O	167.2	-1.35	9.69	52.5	+1				

Substance	MATE1 ratio	SEM	MATE2K ratio	SEM	Therapeutic group	SMILES code	Molecular weight	logD 7.4	most basic pKa	TPSA	Charge	TS DPH hCMEC	SEM	TS MDAI hCMEC	SEM
Phenylephrine (S)	12.74	1.36	1.44	0.05	Sympathomimetic	CNC[C@@H](C1=CC(=CC=C1)O)O	167.2	-1.35	9.69	52.5	+1				
Phenylethanol	1.39	0.06	1.20	0.03	Experimental chemical	C1=CC=C(C=C1)CCO	122.2	1.49		20.2	0				
Pilocarpine	1.39	0.21	1.39	0.05	(Anti)cholinergic	CC[C@H]1[C@H](COC1=O)CC2=CN=CN2C	208.3	0.86	6.94	44.1	0				
Pimozide	0.98	0.08	0.76	0.03	Antipsychotic	C1CN(CCC1N2C3=CC=CC=C3NC2=O)CCCC(C4=CC=C(C=C4)F)C5=CC=C(C=C5)F	461.6	4.33	8.88	35.6	+1				
Pindolol	1.84	0.14	1.18	0.14	Beta blocker	CC(C)NCC(COC1=CC=CC2=C1C=CN2)O	248.3	-0.16	9.27	57.3	+1	0.59	0.09	0.83	0.03
Pinoline	1.28	0.02	1.21	0.09	Endobiotic	COC1=CC2=C(C=C1)NC3=C2CCNC3	202.3	-0.26	9.10	37.1	+1				
Pioglitazone	1.37	0.22	1.01	0.12	Antidiabetic	CCC1=CN=C(C=C1)CCOC2=CC=C(C=C2)CC3C(=O)NC(=O)S3	356.4	3.28	5.63	93.6	-1				
Pipamperone	1.35	0.07	1.32	0.07	Antipsychotic	C1CCN(CC1)C2(CCN(CC2)CCCC(=O)C3=CC=C(C=C3)F)C(=O)N	375.5	0.81	8.40	66.6	+1	0.42	0.07	0.23	0.06
Piperacillin	1.60	0.16	1.27	0.14	Antibiotic	CCN1CCN(C(=O)C1=O)C(=O)N[C@H](C2=CC=CC=C2)C(=O)N[C@H]3[C@@H]4N(C3=O)[C@H](C(S4)(C)C)C(=O)O	517.6	-3.78		181.7	-1				
Piperazine	0.92	0.09	1.03	0.06	Experimental chemical	C1CNCCN1	86.1	-2.86	9.56	24.1	+1				
Pirbuterol	12.66	1.18	6.00	0.73	Symphatomimetic	CC(C)(C)NCC(C1=NC(=C(C=C1)O)CO)O	240.3	-1.54	9.51	85.6	+1	0.95	0.16	1.31	0.22
Pirenzepine	2.80	0.11	1.36	0.07	(Anti)cholinergic	CN1CCN(CC1)CC(=O)N2C3=CC=CC=C3C(=O)NC4=C2N=CC=C4	351.4	0.40	7.83	68.8	+1				
Piritramide	1.25	0.10	1.17	0.10	Opioid	C1CCN(CC1)C2(CCN(CC2)CCC(C#N)(C3=CC=CC=C3)C4=CC=CC=C4)C(=O)N	430.6	2.08	8.68	73.4	+1	0.80	0.00	0.82	0.01
Pomalidomide	1.08	0.09	0.89	0.09	Oncology	C1CC(=O)NC(=O)C1N2C(=O)C3=C(C2=O)C(=CC=C3)N	273.2	-0.16	1.56	109.6	0				

Substance	MATE1 ratio	SEM	MATE2K ratio	SEM	Therapeutic group	SMILES code	Molecular weight	logD 7.4	most basic pKa	TPSA	Charge	TS DPH hCMEC	SEM	TS MDAI hCMEC	SEM
p-Phenylenediamine	1.04	0.06	1.07	0.09	Experimental chemical	C1=CC(=CC=C1N)N	108.1	0.27	6.46	52.0	0				
Practolol	21.52	2.16	8.46	0.46	Beta blocker	CC(C)NCC(COC1=CC=C(C=C1)NC(=O)C)O	266.3	-1.02	9.27	70.6	+1				
Prazosin	1.14	0.01	1.09	0.06	Alpha1 blocker	COC1=C(C=C2C(=C1)C(=NC(=N2)N3CCN(CC3)C(=O)C4=CC=CO4)N)OC	383.4	0.94	8.04	107.0	+1				
Prenalterol	5.01	0.29	1.62	0.06	Sympathomimetic	CC(C)NC[C@@H](COC1=CC=C(C=C1)O)O	225.3	-0.56	9.18	61.7	+1				
Pridinol	1.18	0.06	1.27	0.06	(Anti)cholinergic	C1CCN(CC1)CCC(C2=CC=CC=C2)(C3=CC=CC=C3)O	295.4	1.65	9.47	23.5	+1				
Primaquine	1.42	0.10	1.19	0.10	Antiinfective	CC(CCCN)NC1=C2C(=CC(=C1)OC)C=CC=N2	259.4	-0.96	10.20	60.2	+1				
Procainamide	2.53	0.19	2.18	0.06	Antiarrhythmic	CCN(CC)CCNC(=O)C1=CC=C(C=C1)N	235.3	-0.70	9.04	58.4	+1				
Proguanil	5.54	0.23	3.98	0.24	Antiinfective	CC(C)N=C(N)/N=C(\N)/NC1=CC=C(C=C1)Cl	253.7	-0.44	13.97	88.8	+2				
Propafenone	1.20	0.10	1.09	0.04	Antiarrhythmic	CCCNCC(COC1=CC=CC=C1C(=O)CCC2=CC=CC=C2)O	341.5	1.63	9.32	58.6	+1				
Propafenone (R)	1.34	0.11	1.14	0.10	Antiarrhythmic	CCCNCC(COC1=CC=CC=C1C(=O)CCC2=CC=CC=C2)O	341.5	1.63	9.32	58.6	+1				
Propafenone (S)	1.31	0.05	1.07	0.05	Antiarrhythmic	CCCNCC(COC1=CC=CC=C1C(=O)CCC2=CC=CC=C2)O	341.5	1.63	9.32	58.6	+1				
Propantheline	10.16	1.10	5.77	0.72	(Anti)cholinergic	CC(C)[N+](C)(CCOC(=O)C1C2=CC=CC=C2OC3=CC=CC=C13)C(C)C	368.5	0.36		35.5	+Q				
Propionylcarnitine	0.39	0.02	0.85	0.14	Carnitine ester	CCC(=O)OC(CC(=O)[O-])C[N+](C)(C)C	217.3	-2.98		66.4	+1-1				
Propiverine	1.20	0.07	1.07	0.09	(Anti)cholinergic	CCCOC(C1=CC=CC=C1)(C2=CC=CC=C2)C(=O)OC3CCN(CC3)C	367.5	2.92	8.72	38.8	+1	0.33	0.04	0.44	0.12
Propofol	0.64	0.16	0.80	0.33	Others	CC(C)C1=C(C(=CC=C1)C(C)C)O	178.3	4.16		20.2	0				

Substance	MATE1 ratio	SEM	MATE2K ratio	SEM	Therapeutic group	SMILES code	Molecular weight	logD 7.4	most basic pKa	TPSA	Charge	TS DPH hCMEC	SEM	TS MDAI hCMEC	SEM
Propranolol	1.23	0.08	1.14	0.04	Beta blocker	CC(C)NCC(COC1=CC=CC2=CC=CC=C21)O	259.3	0.73	9.27	41.5	+1	0.12	0.02	0.08	0.04
Propranolol (R)	1.16	0.12	1.04	0.10	Beta blocker	CC(C)NCC(COC1=CC=CC2=CC=CC=C21)O	259.3	0.73	9.27	41.5	+1				
Propranolol (S)	1.13	0.06	0.96	0.02	Beta blocker	CC(C)NCC(COC1=CC=CC2=CC=CC=C21)O	259.3	0.73	9.27	41.5	+1				
Prostaglandin E2	1.35	0.15	1.21	0.07	Others	CCCCC[C@@H](/C=C/[C@H]1[C@@H](CC(=O)[C@@H]1C/C=C\CCCC(=O)O)O)O	352.5	0.26		94.8	-1				
Prostaglandin F2alpha	1.41	0.29	0.92	0.06	Others	CCCCC[C@@H](/C=C/[C@H]1[C@@H](C[C@@H]([C@@H]1C/C=C\CCCC(=O)O)O)O)O	354.5	-0.31		98.0	-1				
Prucalopride	1.15	0.09	1.07	0.03	Others	COCCCN1CCC(CC1)NC(=O)C2=CC(=C(C3=C2OCC3)N)Cl	367.9	-0.55	8.68	76.8	+1				
Pseudoephedrine	1.19	0.11	1.17	0.09	Sympathomimetic	C[C@@H]([C@H](C1=CC=CC=C1)O)NC	165.2	-0.78	9.52	32.3	+1				
Putrescine	0.36	0.16	0.48	0.04	Biogenic amine	C(CCN)CN	88.2	-6.05	10.51	52.0	+2				
Pyrazinamide	0.97	0.02	1.05	0.05	Antibiotic	C1=CN=C(C=N1)C(=O)N	123.1	-1.23		68.9	0	0.95	0.29	1.08	0.11
Pyridoxal	0.97	0.02	1.14	0.03	Vitamin	CC1=NC=C(C(=C1O)C=O)CO	167.2	-0.08	4.31	70.4	0				
Pyridoxamine	2.68	0.18	1.36	0.11	Vitamin	CC1=NC=C(C(=C1O)CN)CO	168.2	-1.83	9.60	79.4	+1				
Pyridoxin	2.33	0.16	1.18	0.07	Vitamin	CC1=NC=C(C(=C1O)CO)CO	169.2	-0.96	5.19	73.6	0				
Pyrilamine	1.39	0.03	1.32	0.07	Antihistaminic	CN(C)CCN(CC1=CC=C(C=C1)OC)C2=CC=CC=N2	285.4	1.66	8.76	28.6	+1				
Pyrimethamine	1.17	0.05	1.10	0.05	Antiinfective	CCC1=C(C(=NC(=N1)N)N)C2=CC=C(C=C2)Cl	248.7	2.23	7.77	77.8	+1				
Pyrithiamine	20.40	1.83	7.32	0.83	Vitamin	CC1=C(C=CC=[N+]1CC2=CN=C(N=C2N)C)CCO	259.3	-3.87	5.54	75.9	+Q				
Quetiapine	1.10	0.19	1.28	0.11	Antipsychotic	C1CN(CCN1CCOCCO)C2=NC3=CC=C	383.5	2.29	7.76	73.6	+1	0.49	0.03	0.45	0.08

Substance	MATE1 ratio	SEM	MATE2K ratio	SEM	Therapeutic group	SMILES code	Molecular weight	logD 7.4	most basic pKa	TPSA	Charge	TS DPH hCMEC	SEM	TS MDAI hCMEC	SEM
						C=C3SC4=CC=CC=C42									
Quinidine	1.12	0.09	1.15	0.05	Herbal	COC1=CC2=C(C=CN=C2C=C1)[C@@H]([C@H]3C[C@@H]4CCN3C[C@@H]4C=C)O	324.4	1.34	8.55	45.6	+1				
Quinine	1.22	0.13	0.97	0.14	Antiinfective	COC1=CC2=C(C=CN=C2C=C1)[C@H]([C@@H]3C[C@@H]4CCN3C[C@@H]4C=C)O	324.4	1.34	8.55	45.6	+1	1.45	0.08	1.51	0.10
Ractopamine	4.56	0.50	1.40	0.12	Sympathomimetic	CC(CCC1=CC=C(C=C1)O)NCC(C2=CC=C(C=C2)O)O	301.4	0.88	9.89	72.7	+1				
Raloxifene	1.12	0.03	1.12	0.08	Others	C1CCN(CC1)CCOC2=CC=C(C=C2)C(=O)C3=C(SC4=C3C=CC(=C4)O)C5=CC=C(C=C5)O	473.6	4.86	8.38	98.2	+1				
Ranitidine	18.91	1.04	8.62	0.46	Antihistaminic	CN/C(=C\[N+](=O)[O-])/NCCSCC1=CC=C(O1)CN(C)C	314.4	0.04	8.30	108.9	+2-1	1.26	0.39	1.24	0.21
Ranolazine	1.11	0.07	1.30	0.07	Others	CC1=C(C(=CC=C1)C)NC(=O)CN2CCN(CC2)CC(COC3=CC=CC=C3OC)O	427.5	2.34	7.73	74.3	+1				
Rasagiline	1.18	0.15	2.16	0.77	MAO inhibitor	C#CCN[C@@H]1CCC2=CC=CC=C12	171.2	1.83	7.70	12.0	+1	0.54	0.05	0.51	0.08
Rasagiline (S)	1.46	0.21	1.46	0.25	MAO inhibitor	C#CCN[C@@H]1CCC2=CC=CC=C12	171.2	1.83	7.70	12.0	+1				
Reboxetine	0.95	0.15	0.94	0.11	Antidepressant	CCOC1=CC=CC=C1O[C@@H]([C@H]2CNCCO2)C3=CC=CC=C3	313.4	2.93	7.49	39.7	+1				
Remoxipride	1.19	0.04	1.21	0.05	Antipsychotic	CCN1CCC[C@H]1CNC(=O)C2=C(C=CC(=C2OC)Br)OC	371.3	0.74	9.00	60.8	+1				
Reproterol	5.18	0.56	17.96	1.71	Sympathomimetic	CN1C2=C(C(=O)N(C1=O)C)N(C=N2)CCCNCC(C3=CC(=CC(=C3)O)O)O	389.4	-2.34	9.66	131.2	+1				

Substance	MATE1 ratio	SEM	MATE2K ratio	SEM	Therapeutic group	SMILES code	Molecular weight	logD 7.4	most basic pKa	TPSA	Charge	TS DPH hCMEC	SEM	TS MDAI hCMEC	SEM
Reserpine	1.61	0.12	1.08	0.03	Antihypertensive	CO[C@H]1[C@@H](C[C@@H]2CN3CCC4=C([C@H]3C[C@@H]2[C@@H]1C(=O)OC)NC5=C4C=CC(=C5)OC)OC(=O)C6=CC(=C(C(=C6)OC)OC)OC	608.7	3.24	7.39	117.8	+1				
Revefenacin	1.39	0.14	1.17	0.06	(Anti)cholinergic	CN(CCN1CCC(CC1)OC(=O)NC2=CC=CC=C2C3=CC=CC=C3)C(=O)C4=CC=C(C=C4)CN5CCC(CC5)C(=O)N	597.8	1.46	8.85	108.2	+2				
Riboflavin	1.92	0.25	4.44	0.25	Vitamin	CC1=CC2=C(C=C1C)N(C3=NC(=O)NC(=O)C3=N2)C[C@@H]([C@@H]([C@@H](CO)O)O)O	376.4	-2.34	0.77	155.1	-1				
Rifampicin	1.02	0.10	0.93	0.05	Antibiotic	C[C@H]1/C=C/C=C(\C(=O)NC2=C(C(=C3C(=C2O)C(=C(C4=C3C(=O)[C@](O4)(O/C=C/[C@@H]([C@H]([C@H]([C@@H]([C@@H]([C@@H]([C@H]1O)C)O)C)OC(=O)C)C)OC)C)C)O)O)/C=N/N5CCN(CC5)C)/C	823.0	3.46	6.91	220.2	+1-1				
Risperidone	1.07	0.13	1.37	0.16	Antipsychotic	CC1=C(C(=O)N2CCCCC2=N1)CCN3CCC(CC3)C4=NOC5=C4C=CC(=C5)F	410.5	1.25	8.76	61.9	+1	0.53	0.04	0.46	0.07
Ritodrine	8.03	0.65	2.23	0.11	Sympathomimetic	C[C@H]([C@H](C1=CC=C(C=C1)O)O)NCCC2=CC=C(C=C2)O	287.4	0.52	10.30	72.7	+1				
Rivastigmine	0.98	0.22	1.11	0.19	Cholinesterase inhibitor	CCN(C)C(=O)OC1=CC=CC(=C1)[C@H](C)N(C)C	250.3	1.00	8.80	32.8	+1	0.73	0.02	0.43	0.12
Rizatriptan	6.63	0.38	3.35	0.31	Triptan	CN(C)CCC1=CNC2=C1C=C(C=C2)CN3C=NC=N3	269.4	-0.40	9.58	49.7	+1				

Substance	MATE1 ratio	SEM	MATE2K ratio	SEM	Therapeutic group	SMILES code	Molecular weight	logD 7.4	most basic pKa	TPSA	Charge	TS DPH hCMEC	SEM	TS MDAI hCMEC	SEM
Ropinirole	1.43	0.15	1.49	0.12	Dopamine agonist	CCCN(CCC)CCC1=C2CC(=O)NC2=CC=C1	260.4	0.40	10.12	32.3	+1	0.67	0.08	0.58	0.06
Ropivacaine	1.22	0.06	1.49	0.14	Local anesthetic	CCCN1CCCC[C@H]1C(=O)NC2=C(C=CC=C2C)C	274.4	3.53	7.80	32.3	+1	0.15	0.00	0.37	0.08
Rucaparib	3.71	0.08	2.03	0.08	Cytostatic	CNCC1=CC=C(C=C1)C2=C3CCNC(=O)C4=C3C(=CC(=C4)F)N2	323.4	0.55	9.32	56.9	+1				
Ruxolitinib	1.13	0.03	0.99	0.04	JAK inhibitor	C1CCC(C1)[C@@H](CC#N)N2C=C(C=N2)C3=C4C=CNC4=NC=N3	306.4	2.48	5.01	83.2	0				
Salbutamol	7.94	0.45	3.73	0.19	Sympathomimetic	CC(C)(C)NCC(C1=CC(=C(C=C1)O)CO)O	239.2	-1.32	9.40	72.7	+1				
Salbutamol (R)	6.98	0.48	3.74	0.30	Sympathomimetic	CC(C)(C)NCC(C1=CC(=C(C=C1)O)CO)O	239.2	-1.32	9.40	72.7	+1				
Salbutamol (S)	8.88	0.32	5.09	0.42	Sympathomimetic	CC(C)(C)NCC(C1=CC(=C(C=C1)O)CO)O	239.2	-1.32	9.40	72.7	+1				
Salmeterol	1.78	0.07	1.17	0.06	Sympathomimetic	C1=CC=C(C=C1)CCCCOCCCCCCNCC(C2=CC(=C(C=C2)O)CO)O	415.6	1.95	9.40	82.0	+1				
Salsolidine	1.24	0.02	1.46	0.04	Herbal	CC1C2=CC(=C(C=C2CCN1)OC)OC	207.3	0.17	8.89	30.5	+1				
Salsolinol	13.05	1.45	5.20	0.62	Endobiotic	C[C@H]1C2=CC(=C(C=C2CCN1)O)O	179.2	0.15	8.51	52.5	+1				
Sarcosine	0.76	0.07	1.01	0.07	Endobiotic	CNCC(=O)O	89.1	-3.19	10.35	49.3	+1-1				
Scopolamine	1.18	0.08	1.40	0.10	(Anti)cholinergic	CN1[C@@H]2CC(C[C@H]1[C@H]3[C@H]2O3)OC(=O)[C@H](CO)C4=CC=CC=C4	303.4	0.85	6.45	62.3	0	0.63	0.13	0.44	0.02
Selegiline	1.87	0.58	1.68	0.20	MAO inhibitor	C[C@H](CC1=CC=CC=C1)N(C)CC#C	187.3	1.53	8.70	3.2	+1	0.56	0.09	0.50	0.08
Selegiline (R)	1.20	0.07	2.08	0.44	MAO inhibitor	CC(CC1=CC=CC=C1)N(C)CC#C	187.3	1.53	8.70	3.2	+1				
Selegiline (S)	1.84	0.40	1.08	0.16	MAO inhibitor	CC(CC1=CC=CC=C1)N(C)CC#C	187.3	1.53	8.70	3.2	+1				
Sematilide	19.10	1.49	6.42	0.50	Antiarrhythmic	CCN(CC)CCNC(=O)C1=CC=C(C=C1)NS(=O)(=O)C	313.4	-1.38	9.07	86.9	+1				
Sepantronium	6.15	0.52	9.45	1.12	Cytostatic	CC1=[N+](C2=C(N1CCOC)C(=O)C3=CC	363.4	-3.72		78.0	+Q	1.01	0.05	0.99	0.11

Substance	MATE1 ratio	SEM	MATE2K ratio	SEM	Therapeutic group	SMILES code	Molecular weight	logD 7.4	most basic pKa	TPSA	Charge	TS DPH hCMEC	SEM	TS MDAI hCMEC	SEM
						=CC=C3C2=O)CC4=NC=CN=C4									
Serotonin	12.11	1.01	2.26	0.16	Biogenic amine	C1=CC2=C(C=C1O)C(=CN2)CCN	176.2	-1.05	10.02	62.0	+1				
Sertraline	1.28	0.09	1.10	0.08	Antidepressant	CN[C@H]1CC[C@H](C2=CC=CC=C12)C3=CC(=C(C=C3)Cl)Cl	306.2	3.02	9.56	12.0	+1				
Sitagliptin	4.17	1.10	2.77	0.13	Antidiabetic	C1CN2C(=NN=C2C(F)(F)F)CN1C(=O)C[C@@H](CC3=CC(=C(C=C3F)F)F)N	407.3	-0.14	8.78	77.0	+1				
Solifenacin	1.28	0.01	1.22	0.04	(Anti)cholinergic	C1CN2CCC1[C@H](C2)OC(=O)N3CCC4=CC=CC=C4[C@@H]3C5=CC=CC=C5	362.5	2.47	8.88	32.8	+1	0.27	0.04	0.44	0.02
Sotalol	19.70	1.31	1.62	0.06	Antiarrhythmic	CC(C)NCC(C1=CC=C(C=C1)NS(=O)(=O)C)O	272.4	-1.98	9.64	86.8	+1				
Sparteine (-)	2.12	0.11	1.66	0.13	Antiarrhythmic	C1CCN2C[C@@H]3C[C@H]([C@H]2C1)CN4[C@H]3CCCC4	234.4	0.24	9.06	6.5	+1				
Sparteine (+)	1.62	0.26	1.31	0.13	Antiarrhythmic	C1CCN2C[C@H]3C[C@@H]([C@H]2C1)CN4[C@H]3CCCC4	234.4	0.24	9.06	6.5	+1				
Spermidine	0.63	0.31	0.55	0.14	Biogenic amine	C(CCNCCCN)CN	145.3	-7.35	10.61	64.1	+3				
Spermine	1.13	0.11	1.25	0.30	Endobiotic	C(CCNCCCN)CNCCCN	202.4	-8.65	10.70	76.1	+4				
Stachydrine	0.83	0.08	1.31	0.19	Herbal	C[N+]1(CCC[C@H]1C(=O)[O-])C	143.2	-3.11		40.1	+1-1				
Strychnine	1.13	0.07	1.43	0.17	Herbal	C1CN2CC3=CCO[C@H]4CC(=O)N5[C@H]6[C@H]4[C@H]3C[C@H]2[C@@]61C7=CC=CC=C75	334.4	-0.65	8.97	32.8	+1				
Succinylcarnitine	0.54	0.06	1.38	0.16	Carnitine ester	C[N+](C)(C)C[C@@H](CC(=O)[O-])OC(=O)CCC(=O)O	261.3	-6.80		103.7	+1-2				
Sufentanil	1.32	0.07	1.40	0.04	Opioid	CCC(=O)N(C1=CC=CC=C1)C2(CCN(CC2)CCC3=CC=CS3)COC	386.6	2.41	8.56	61.0	+1	0.24	0.04	0.03	0.03

Substance	MATE1 ratio	SEM	MATE2K ratio	SEM	Therapeutic group	SMILES code	Molecular weight	logD 7.4	most basic pKa	TPSA	Charge	TS DPH hCMEC	SEM	TS MDAI hCMEC	SEM
Sulpiride	23.85	1.07	21.71	1.58	Antipsychotic	CCN1CCCC1CNC(=O)C2=C(C=CC(=C2)S(=O)(=O)N)OC	341.4	-1.26	8.97	110.1	+1				
Sulpiride (S)	22.45	1.67	18.54	1.36	Antipsychotic	CCN1CCCC1CNC(=O)C2=C(C=CC(=C2)S(=O)(=O)N)OC	341.4	-1.26	8.97	110.1	+1				
Sumatriptan	15.85	1.62	7.36	0.91	Triptan	CNS(=O)(=O)CC1=CC2=C(C=C1)NC=C2CCN(C)C	295.4	-1.26	9.56	73.6	+1				
Synephrine	6.50	0.97	1.04	0.06	Sympathomimetic	CNCC(C1=CC=C(C=C1)O)O	167.2	-1.39	9.15	52.5	+1				
Talinolol	7.98	0.74	4.06	0.15	Beta blocker	CC(C)(C)NCC(COC1=CC=C(C=C1)NC(=O)NC2CCCCC2)O	363.5	0.86	9.36	82.6	+1				
Tamoxifen	1.33	0.20	1.06	0.05	Oncology	CC/C(=C(\C1=CC=CC=C1)/C2=CC=C(C=C2)OCCN(C)C)/C3=CC=CC=C3	371.5	4.97	8.76	12.5	+1				
Tamsulosin	1.40	0.11	1.16	0.07	Alpha1 blocker	CCOC1=CC=CC=C1OCCN[C@H](C)CC2=CC(=C(C=C2)OC)S(=O)(=O)N	408.5	1.02	8.84	108.3	+1	0.77	0.10	0.88	0.07
Tapentadol	1.18	0.07	1.10	0.08	Opioid	CC[C@@H](C1=CC(=CC=C1)O)[C@@H](C)CN(C)C	221.3	1.07	9.60	23.5	+1				
Taurine	1.23	0.04	1.20	0.02	Endobiotic	C(CS(=O)(=O)O)N	125.1	-2.62	9.34	88.8	+1-1				
Temozolomide	1.03	0.05	1.10	0.04	Cytostatic	CN1C(=O)N2C=NC(=C2N=N1)C(=O)N	194.2	-0.28		105.9	0				
Terazosin	1.17	0.05	1.28	0.10	Alpha1 blocker	COC1=C(C=C2C(=C1)C(=NC(=N2)N3CCN(CC3)C(=O)C4CCCO4)N)OC	387.4	0.47	8.04	103.0	+1	0.79	0.08	0.44	0.11
Terbutaline	6.74	0.14	7.04	0.72	Sympathomimetic	CC(C)(C)NCC(C1=CC(=CC(=C1)O)O)O	225.3	-0.73	9.76	72.7	+1				
Terfenadine	1.07	0.21	0.93	0.15	Antihistaminic	CC(C)(C)C1=CC=C(C=C1)C(CCCN2CCC(CC2)C(C3=CC=CC=C3)(C4=CC=CC=C4)O)O	471.7	4.66	9.22	43.7	+1	0.67	0.06	0.48	0.01
Tetracaine	1.12	0.24	1.30	0.05	Local anesthetic	CCCCNC1=CC=C(C=C1)C(=O)OCCN(C)C	264.4	1.74	8.42	41.6	+1	0.63	0.12	0.39	0.03

Substance	MATE1 ratio	SEM	MATE2K ratio	SEM	Therapeutic group	SMILES code	Molecular weight	logD 7.4	most basic pKa	TPSA	Charge	TS DPH hCMEC	SEM	TS MDAI hCMEC	SEM
Tetracycline	1.23	0.18	1.16	0.10	Antibiotic	C[C@@]1([C@H]2C[C@H]3[C@@H](C(=O)C(=C([C@]3(C(=O)C2=C(C4=C1C=CC=C4O)O)O)O)O)C(=O)N)N(C)C)O	444.4	-6.07	7.97	181.6	-1				
Tetraethylammonium	25.44	1.15	6.73	0.49	Experimental chemical	CC[N+](CC)(CC)CC	130.3	-2.54		0.0	+Q				
Thalidomide	0.85	0.04	2.02	0.18	Others	C1CC(=O)NC(=O)C1N2C(=O)C3=CC=CC=C3C2=O	258.2	0.02		83.6	0				
Thiamine	33.42	6.84	10.63	0.95	Vitamin	CC1=C(SC=[N+]1CC2=CN=C(N=C2N)C)CCO	265.4	-3.10	5.54	104.2	+Q	0.84	0.02	1.60	0.31
Thiamine monophosphate	1.05	0.03	0.89	0.08	Vitamin	CC1=C(SC=[N+]1CC2=CN=C(N=C2N)C)CCOP(=O)(O)O	345.3	-5.14	5.51	160.5	+Q				
Thyrotropin-Releasing-Hormon	1.71	0.36	1.16	0.23	Hormone	C1C[C@H](N(C1)C(=O)[C@H](CC2=CN=CN2)NC(=O)[C@@H]3CCC(=O)N3)C(=O)N	362.4	-3.35	6.86	150.3	0				
Tianeptine	1.08	0.14	0.97	0.06	Antidepressant	CN1C2=CC=CC=C2C(C3=C(S1(=O)=O)C=C(C=C3)Cl)NCCCCCCC(=O)O	437.0	1.43	7.81	86.7	+1-1	1.20	0.05	1.12	0.13
Tiglylcarnitine	0.96	0.06	0.76	0.06	Carnitine ester	C/C=C(\C)/C(=O)OC(CC(=O)[O-])C[N+](C)(C)C	243.3	-1.90		66.4	+1-1				
Timolol	1.39	0.28	1.40	0.27	Beta blocker	CC(C)(C)NC[C@@H](COC1=NSN=C1N2CCOCC2)O	316.4	-0.61	9.36	108.0	+1				
Timolol (R)	0.94	0.11	0.96	0.13	Beta blocker	CC(C)(C)NC[C@@H](COC1=NSN=C1N2CCOCC2)O	316.4	-0.61	9.36	108.0	+1				
Tiotropium	27.40	0.32	1.94	0.15	(Anti)cholinergic	C[N+]1([C@@H]2CC(C[C@H]1[C@H]3[C@@H]2O3)OC(=O)C(C4=CC=CS4)(C5=CC=CS5)O)C	392.5	-1.75		115.5	+Q				
Tofacitinib	1.36	0.27	1.41	0.23	JAK inhibitor	C[C@@H]1CCN(C[C@@H]1N(C)C2=NC=NC3=C2C=CN3)C(=O)CC#N	312.4	0.87	7.53	88.9	+1				

Substance	MATE1 ratio	SEM	MATE2K ratio	SEM	Therapeutic group	SMILES code	Molecular weight	logD 7.4	most basic pKa	TPSA	Charge	TS DPH hCMEC	SEM	TS MDAI hCMEC	SEM
Tolterodine (R)	1.15	0.06	1.06	0.04	(Anti)cholinergic	CC1=CC(=C(C=C1)O)[C@H](CCN(C(C)C)C(C)C)C2=CC=CC=C2	325.5	2.78	10.90	23.5	+1				
Tolterodine (S)	1.16	0.02	1.11	0.08	(Anti)cholinergic	CC1=CC(=C(C=C1)O)[C@H](CCN(C(C)C)C(C)C)C2=CC=CC=C2	325.5	2.78	10.90	23.5	+1				
Topotecan	2.53	0.24	1.61	0.13	Cytostatic	CC[C@@]1(C2=C(COC1=O)C(=O)N3CC4=CC5=C(C=CC(=C5CN(C)C)O)N=C4C3=C2)O	421.5	-2.38	9.12	103.2	+1				
Tramadol	1.13	0.10	1.44	0.21	Opioid	CN(C)C[C@H]1CCCC[C@@]1(C2=CC(=CC=C2)OC)O	263.4	0.48	9.38	32.7	+1				
Tranylcypromine	1.45	0.32	1.98	0.12	MAO inhibitor	C1[C@H]([C@@H]1N)C2=CC=CC=C2	133.2	-0.16	8.90	26.0	+1	0.30	0.08	0.11	0.05
Triethylentetramine	0.65	0.12	1.11	0.04	Others	C(CNCCNCCN)N	146.2	-6.30	9.77	76.1	+2				
Trimethoprim	1.52	0.06	1.23	0.06	Antibiotic	COC1=CC(=CC(=C1OC)OC)CC2=CN=C(N=C2N)N	290.3	1.10	7.16	105.5	0	0.60	0.21	0.85	0.19
Trimipramine	1.19	0.03	1.20	0.04	Antidepressant	CC(CN1C2=CC=CC=C2CCC3=CC=CC=C31)CN(C)C	294.4	2.75	9.42	6.5	+1	0.35	0.05	0.21	0.03
Tropicamide	1.10	0.05	1.08	0.13	(Anti)cholinergic	CCN(CC1=CC=NC=C1)C(=O)C(CO)C2=CC=CC=C2	284.4	1.38	5.02	53.4	0				
Tropisetron	1.22	0.11	1.15	0.09	5HT3 Antagonist	CN1[C@@H]2CC[C@H]1CC(C2)OC(=O)C3=CNC4=CC=CC=C43	284.4	0.89	9.14	45.3	+1				
Trospium	21.28	0.83	5.18	0.15	(Anti)cholinergic	C1CC[N+]2(C1)[C@@H]3CC[C@H]2CC(C3)OC(=O)C(C4=CC=CC=C4)(C5=CC=CC=C5)O	392.5	-0.50		46.5	+Q				
Tryptamine	2.21	0.05	1.16	0.03	Biogenic amine	C1=CC=C2C(=C1)C(=CN2)CCN	160.2	-0.79	9.76	41.8	+1				
Tryptophan	0.82	0.04	1.07	0.04	Amino acid	C1=CC=C2C(=C1)C(=CN2)C[C@@H](C(=O)O)N	204.2	-1.09	9.40	79.1	+1-1				

Substance	MATE1 ratio	SEM	MATE2K ratio	SEM	Therapeutic group	SMILES code	Molecular weight	logD 7.4	most basic pKa	TPSA	Charge	TS DPH hCMEC	SEM	TS MDAI hCMEC	SEM
Tubocurarine	1.94	0.06	1.00	0.18	Muscle relaxant	CN1CCC2=CC(=C3C=C2[C@@H]1CC4=CC=C(C=C4)OC5=C6[C@@H](CC7=CC(=C(C=C7)O)O3)[N+](CCC6=CC(=C5O)OC)(C)C)OC	609.7	2.24		80.6	+Q2				
Tulobuterol	1.31	0.23	1.74	0.30	Sympathomimetic	CC(C)(C)NCC(C1=CC=CC=C1Cl)O	227.7	0.44	9.55	32.3	+1				
Tyramine	10.06	1.02	1.39	0.08	Sympathomimetic	C1=CC(=CC=C1CCN)O	137.2	-1.17	9.48	46.3	+1				
Umeclidinium	4.54	0.58	1.67	0.26	(Anti)cholinergic	C1C[N+]2(CCC1(CC2)C(C3=CC=CC=C3)(C4=CC=CC=C4)O)CCOCC5=CC=CC=C5	428.6	0.68		29.5	+Q				
Upadacitinib	1.51	0.10	1.27	0.05	JAK inhibitor	CC[C@@H]1CN(C[C@@H]1C2=CN=C3N2C4=C(NC=C4)N=C3)C(=O)NCC(F)(F)F	380.4	0.85	3.41	78.3	0				
Urapidil	1.19	0.02	1.17	0.03	Antihypertensive	CN1C(=CC(=O)N(C1=O)C)NCCCN2CCN(CC2)C3=CC=CC=C3OC	387.5	0.22	8.31	68.4	+1	0.60	0.02	0.68	0.24
Varenicline	1.20	0.09	1.30	0.05	Others	C1C2CNCC1C3=CC4=NC=CN=C4C=C23	211.3	-1.27	9.73	37.8	+1				
Vecuronium	8.32	1.00	4.72	0.66	Muscle relaxant	CC(=O)O[C@H]1C[C@@H]2CC[C@@H]3[C@@H]([C@]2(C[C@@H]1N4CCCCC4)C)CC[C@]5([C@H]3C[C@@H]([C@@H]5OC(=O)C)[N+]6(CCCCC6)C)C	557.8	-1.34	9.65	55.8	+Q2				
Venlafaxine	1.22	0.05	1.30	0.04	Antidepressant	CN(C)CC(C1=CC=C(C=C1)OC)C2(CCCCC2)O	277.4	1.12	9.01	32.7	+1	0.38	0.04	0.24	0.02
Venlafaxine (R)	1.14	0.01	1.36	0.06	Antidepressant	CN(C)CC(C1=CC=C(C=C1)OC)C2(CCCCC2)O	277.4	1.12	9.01	32.7	+1				
Venlafaxine (S)	1.22	0.02	1.44	0.04	Antidepressant	CN(C)CC(C1=CC=C(C=C1)OC)C2(CCCCC2)O	277.4	1.12	9.01	32.7	+1				

Substance	MATE1 ratio	SEM	MATE2K ratio	SEM	Therapeutic group	SMILES code	Molecular weight	logD 7.4	most basic pKa	TPSA	Charge	TS DPH hCMEC	SEM	TS MDAI hCMEC	SEM
Veralipride	6.39	0.18	4.21	0.38	Antipsychotic	COC1=CC(=CC(=C1OC)C(=O)NCC2CCCN2CC=C)S(=O)(=O)N	383.5	-0.46	8.36	119.3	+1				
Verapamil	1.22	0.02	1.08	0.05	Antiarrhythmic	CC(C)C(CCCN(C)CCC1=CC(=C(C=C1)OC)OC)(C#N)C2=CC(=C(C=C2)OC)OC	454.6	2.86	9.61	64.0	+1				
Verapamil (R)	1.17	0.04	1.14	0.03	Antiarrhythmic	CC(C)C(CCCN(C)CCC1=CC(=C(C=C1)OC)OC)(C#N)C2=CC(=C(C=C2)OC)OC	454.6	2.86	9.61	64.0	+1				
Verapamil (S)	1.16	0.06	1.19	0.04	Antiarrhythmic	CC(C)C(CCCN(C)CCC1=CC(=C(C=C1)OC)OC)(C#N)C2=CC(=C(C=C2)OC)OC	454.6	2.86	9.61	64.0	+1				
Vildagliptin	1.48	0.07	1.06	0.07	Antidiabetic	C1C[C@H](N(C1)C(=O)CNC23CC4CC(C2)CC(C4)(C3)O)C#N	303.4	-1.61	8.78	76.4	+1				
Viloxazine	1.12	0.05	1.38	0.06	Antidepressant	CCOC1=CC=CC=C1OCC2CNCCO2	237.3	0.95	7.79	39.7	+1				
Xamoterol	7.62	1.07	6.13	0.35	Sympathomimetic	C1COCCN1C(=O)NCCNCC(COC2=CC=C(C=C2)O)O	339.4	-1.60	8.38	103.3	+1				
Ximelagatran	1.19	0.11	1.20	0.07	Anticoagulant	CCOC(=O)CN[C@H](C1CCCCC1)C(=O)N2CC[C@H]2C(=O)NCC3=CC=C(C=C3)/C(=N/O)/N	473.6	0.77	6.65	146.4	0				
Xylometazoline	1.55	0.16	1.11	0.08	Sympathomimetic	CC1=CC(=CC(=C1CC2=NCCN2)C)C(C)(C)C	244.4	1.49	10.29	24.4	+1				
Yohimbin	1.11	0.11	1.35	0.10	Herbal	COC(=O)[C@H]1[C@H](CC[C@@H]2[C@@H]1C[C@H]3C4=C(CCN3C2)C5=CC=CC=C5N4)O	354.5	1.76	7.48	65.6	+1				
Zalcitabine	12.98	0.90	1.22	0.06	Virostatic	C1C[C@@H](O[C@@H]1CO)N2C=CC(=NC2=O)N	211.2	-1.19	3.88	88.2	0				
Zebularine	1.12	0.13	0.90	0.09	Cytostatic	C1=CN(C(=O)N=C1)[C@H]2[C@@H]([C@@H]([C@H](O2)CO)O)O	228.2	-2.21		102.6	0				

Substance	MATE1 ratio	SEM	MATE2K ratio	SEM	Therapeutic group	SMILES code	Molecular weight	logD 7.4	most basic pKa	TPSA	Charge	TS DPH hCMEC	SEM	TS MDAI hCMEC	SEM
Ziprasidone	1.20	0.09	1.25	0.04	Antipsychotic	C1CN(CCN1CCC2=C(C=C3C(=C2)CC(=O)N3)Cl)C4=NSC5=CC=CC=C54	412.9	2.83	8.87	76.7	+1				
Zolmitriptan	11.83	0.56	5.51	0.56	Triptan	CN(C)CCC1=CNC2=C1C=C(C=C2)C[C@H]3COC(=O)N3	287.4	-0.11	9.57	57.4	+1	1.03	0.33	1.45	0.35
Zolmitriptan (R)	12.88	0.36	6.48	0.04	Triptan	CN(C)CCC1=CNC2=C1C=C(C=C2)C[C@H]3COC(=O)N3	287.4	-0.11	9.57	57.4	+1				
Zolmitriptan (S)	13.34	0.71	5.38	0.27	Triptan	CN(C)CCC1=CNC2=C1C=C(C=C2)C[C@H]3COC(=O)N3	287.4	-0.11	9.57	57.4	+1				
Zotepin	0.51	0.04	1.34	0.26	Antipsychotic	CN(C)CCOC1=CC2=CC=CC=C2SC3=C1C=C(C=C3)Cl	331.9	2.98	8.92	37.8	+1				

MATE1 ratio and MATE2K ratio refer to the ratio of uptake into HEK293 cells overexpressing these transporters over HEK293 cells treated with the same but empty vector. SEM is the respective standard error of the mean. TPSA refers to topological polar surface area. TS DPH hCMEC and TS MDAI hCMEC refer to trans-stimulation (antiport) experiments in which the antiport of the respective substrates and diphenhydramine (DPH) or 5,6-Methylenedioxy-2-aminoindane (MDAI) was tested in the immortalized cell line from human cerebral vascular endothelial cells. Ratios below 0.7 indicate substrates of the proton organic cation transporters expressed in this hCMEC cell line. [**Ali Khan, C.**, Kirsch, N., Brockmöller, J., & Redeker, K. M. (**2024**). An extended substrate spectrum of the proton organic cation antiporter and relation to other cation transporters. *Basic & clinical pharmacology & toxicology*, 135(6), 720–742. https://doi.org/10.1111/bcpt.14090; **Doetsch, D. A.**, Ansari, S., Jensen, O., Gebauer, L., Dücker, C., Brockmöller, J., & Sachkova, A. (**2022**). Substrates of the Human Brain Proton-Organic Cation Antiporter and Comparison with Organic Cation Transporter 1 Activities. *International journal of molecular sciences*, 23(15), 8430. https://doi.org/10.3390/ijms23158430].

Table S3: Uptake ratio of racemates and single enantiomers by MATE1 and MATE2K

		R-enantiomere				S-enantiomere				Racemate			
**Substance**	**Enantiomeric specification**	**MATE1 mean**	SEM	**MATE2K mean**	SEM	**MATE1 mean**	SEM	**MATE2K mean**	SEM	**MATE1 mean**	SEM	**MATE2K mean**	SEM
Aclidinium		4.73	1.31	1.17	0.32	5.36	0.18	1.64	0.09				
Amisulpride		8.46	0.84	7.40	0.85	13.70	1.98	7.93	0.46	11.04	1.00	11.15	0.11
Atenolol		25.72	2.00	6.79	0.47	25.17	1.04	9.28	1.22	18.66	0.70	4.56	1.12
Atomoxetine		1.12	0.09	1.18	0.08	1.16	0.03	1.03	0.05				
Bisnorephedrine		1.45	0.05	1.16	0.05					1.42	0.15	1.30	0.10
Bupivacaine		1.18	0.03	1.31	0.15	1.00	0.03	1.08	0.08	1.15	0.08	1.32	0.07
Carvedilol		1.17	0.04	1.08	0.03	1.19	0.06	1.18	0.02				
Cetirizin	L-	1.26	0.027	0.98	0.125					1.247	0.076	1.190	0.066
Citalopram		1.33	0.04	1.17	0.06	1.26	0.13	1.27	0.05	1.40	0.15	1.46	0.08
Cycloserine	D- / L-	1.19	0.13	1.14	0.06	1.19	0.10	1.10	0.02				
Doxepine	E- / Z-	1.26	0.04	1.08	0.07	1.28	0.05	1.09	0.06	1.34	0.12	1.32	0.02
Doxylamine		1.23	0.10	1.50	0.07	1.12	0.11	1.58	0.13	1.17	0.08	1.27	0.03
Dropropizine		1.12	0.14	1.39	0.09	1.14	0.04	1.37	0.06	1.25	0.04	1.45	0.18
Duloxetine		1.23	0.03	1.07	0.03	1.23	0.04	1.06	0.04	1.25	0.10	0.99	0.10
Epinephrine		37.78	4.79	3.98	1.00					18.70	2.49	3.27	0.57
Fenfluramine		1.08	0.07	2.47	0.30	0.80	0.10	1.82	0.30				
Fluoxetine		1.23	0.08	1.05	0.06	1.18	0.06	1.12	0.03	1.30	0.07	1.16	0.05
Formoterol		5.18	0.32	1.72	0.10					3.64	0.49	1.38	0.13
Hydroxychloroquine		2.22	0.19	1.62	0.03	2.05	0.06	1.45	0.10				
Mexiletine		1.03	0.12	1.39	0.23	1.14	0.20	1.61	0.27	1.34	0.30	1.51	0.28
Norepinephrine		12.34	1.33	1.63	0.25	11.02	0.87	1.08	0.05	11.70	1.21	1.63	0.29
Ofloxacin		1.53	0.16	1.41	0.06	1.93	0.09	1.41	0.04				
Palonosetron	R,R- / S,S-	1.11	0.08	1.03	0.03	1.35	0.09	1.16	0.07	1.30	0.12	1.38	0.08
Phenylephrine		18.71	0.16	2.15	0.18	12.74	1.36	1.44	0.05	13.78	1.04	1.75	0.02
Propafenone		1.34	0.11	1.14	0.10	1.31	0.05	1.07	0.05	1.20	0.10	1.09	0.04

		R-enantiomere				S-enantiomere				Racemate			
**Substance**	**Enantiomeric specification**	**MATE1 mean**	SEM	**MATE2K mean**	SEM	**MATE1 mean**	SEM	**MATE2K mean**	SEM	**MATE1 mean**	SEM	**MATE2K mean**	SEM
Propranolol		1.16	0.12	1.04	0.10	1.13	0.06	0.96	0.02	1.23	0.08	1.14	0.04
Quinine/Quinidine		1.22	0.13	0.97	0.14	1.12	0.09	1.15	0.05				
Rasagiline		1.18	0.15	2.16	0.77	1.46	0.21	1.46	0.25				
Salbutamol		6.98	0.48	3.74	0.30	8.88	0.32	5.09	0.42	7.94	0.45	3.73	0.19
Selegiline		1.20	0.07	2.08	0.44	1.84	0.40	1.08	0.16	1.87	0.580	1.68	0.203
Sparteine	(+) / (-)	1.62	0.26	1.31	0.13	2.12	0.11	1.66	0.13				
Sulpiride						22.45	1.67	18.54	1.36	23.85	1.07	21.71	1.58
Timolol		0.94	0.11	0.96	0.13	1.39	0.28	1.40	0.27				
Tolterodine		1.15	0.06	1.06	0.04	1.16	0.02	1.11	0.08				
Venlafaxine		1.14	0.01	1.36	0.06	1.22	0.02	1.44	0.04	1.22	0.05	1.30	0.04
Verapamil		1.17	0.04	1.14	0.03	1.16	0.06	1.19	0.04	1.22	0.02	1.08	0.05
Zolmitriptan		12.88	0.36	6.48	0.04	13.34	0.71	5.38	0.27	11.83	0.56	5.51	0.56

Table S4: Biochemical parameters and substrate data of 590 tested substances

Substance	MATE1 substrate*	MATE2 substrate*	OCT1 substrate*	OCT2 substrate*	Therapeutic group	SMILES	Molecular weight	logD at pH 7.4	pKa (basic)	TPSA	Net Charge	Quarternary N	Zwitterionic	ChEMBL ID	AlogP	Hydrogen bound	Hydrogen bound	Aromatic rings	Heavy atoms	Natprod likeness score	Rqed (weighted)	Rotable bounds
1-(3-Chlorophenyl)piperazine	0	0			Antidepressant	C1CN(CCN1)C2=CC(=CC=C2)Cl	196.7	0.71	8.83	15.3	1	0	0	CHEMBL478	1.75	2	1	1	13	-1.66	0.74	1
1-(4-Chlorophenyl)biguanide	1	1	1	1	Antiinfective	C1=CC(=CC=C1N=C(N)N=C(N)N)Cl	211.7	-1.27	11.82	102.8	2	0	0	CHEMBL1256027	1.17	5	3	1	14	-1.14	0.36	1
1,3-Diphenylguanidine	0	0	1	1	Experimental chemical	C1=CC=C(C=C1)NC(=NC2=CC=CC=C2)N	211.3	1.22	10.93	50.4	1	0	0	CHEMBL77675	3.15	1	3	2	16	-0.41	0.53	2
10-OH-Nortriptyline	0	0	0	0	Antidepressant	CNCC/C=C\1/C2=CC=CC=C2CC(C3=CC=CC=C31)O	279.4	0.35	10.47	32.3	1	0	0	CHEMBL1201156	3.83	2	2	2	21	0.35	0.83	3
17-Alpha-methyltestosterone	0	0		0	Steroid hormone	C[C@]12CCC(=O)C=C1CC[C@@H]3[C@@H]2CC[C@]4([C@H]3CC[C@]4(C)O)C	302.2	3.65	-0.53	37.3	0	0	0	CHEMBL1395	4.27	2	1	0	22	2.12	0.73	0
1-Methyl-4-phenylpyridinium	1	1	1	1	Experimental chemical	C[N+]1=CC=C(C=C1)C2=CC=CC=C2	170.2	-1.54	14.00	3.9	1	1	0	CHEMBL311617	2.18	0	0	2	13	-0.19	0.58	1
1-Methylxanthine	0	0	0	0	Xanthine derivative	CN1C(=O)C2=C(NC1=O)N=CN2	166.1	0.08	-1.20	78.1	0	0	0	CHEMBL1250	-1.05	4	2	2	12	-0.27	0.52	0
2,5-Dimethoxy-4-iodoamphetamine	0	0	0		Psychostimulant	CC(CC1=CC(=C(C=C1OC)I)OC)N	321.2	0.08	9.83	44.5	1	0	0	CHEMBL6616	2.2	3	1	1	15	-0.04	0.86	4
2-Dimethylaminoethanol	0	0	0		Experimental chemical	CN(C)CCO	89.1	-2.13	9.03	23.5	1	0	0	CHEMBL1135	-0.46	2	1	0	6	-0.68	0.49	2
2-Methylamino-1-(3,4-methylendioxyphenyl)butan	0	0	0	0	Psychostimulant	CCC(CC1=CC2=C(C=C1)OCO2)NC	207.3	-0.34	10.28	30.5	1	0	0	CHEMBL225806	3.12	3	1	1	15	-0.46	0.67	4
2-Methylbutyrylcarnitine	0	0	0	0	Carnitine ester	CCC(C)C(=O)OC(CC(=O)[O-])C[N+](C)(C)C	245.3	-1.99	14.00	66.4	0	1	1	#NV	-2.7	7	0	0	17	#NV	#NV	8
2-Phenylethylamine	0	0	0	1	Biogenic amine	C1=CC=C(C=C1)CCN	121.2	-0.94	9.82	26.0	1	0	0	CHEMBL610	1.19	1	1	1	9	0.11	0.62	2
3,4-Methylendioxy-N-methylamphetamine	0	0	1	1	Psychostimulant	CC(CC1=CC2=C(C=C1)OCO2)NC	193.2	-0.76	10.14	30.5	1	0	0	CHEMBL43048	1.57	3	1	1	14	0.11	0.79	3

Substance	MATE1 substrate*	MATE2 substrate*	OCT1 substrate*	OCT2 substrate*	Therapeutic group	SMILES	Molecular weight	logD at pH 7.4	pKa (basic)	TPSA	Net Charge	Quaternary N	Zwitterionic	ChEMBL ID	AlogP	Hydrogen bound	Hydrogen bound	Aromatic rings	Heavy atoms	Natprod likeness score	Rqed (weighted)	Rotable bounds
3-Iodothyronamine (T1AM)	0	0	0	0	Others	C1=CC(=CC=C1O)OC2=C(C=C(C=C2)CCN)I	355.2	1.31	10.00	55.5	1	0	0	CHEMBL1182312	3.29	3	2	2	18	0.28	0.83	4
3-Methoxymorphinan	0	0	0	0	Opioid	COC1=CC2=C(C[C@@H]3[C@H]4[C@@]2(CCCC4)CCN3)C=C1	257.4	0.43	10.22	21.3	1	0	0	CHEMBL1623871	3.04	2	1	1	19	1.25	0.83	1
3-Methoxy-p-tyramine	1	0	1		Endobiotic	COC1=C(C=CC(=C1)CCN)O	167.2	-1.31	9.62	55.5	1	0	0	CHEMBL11608	0.89	3	2	1	12	0.76	0.64	3
4-Amino-1,8-naphthalimide	0	0	0	0	Oncology	C1=CC2=C(C=CC3=C2C(=C1)C(=O)NC3=O)N	212.2	0.84	2.64	72.2	0	0	0	CHEMBL338790	1.31	3	2	2	16	-0.16	0.51	0
4-Aminoantipyrine	1	0	0	0	Analgesic	CC1=C(C(=O)N(N1C)C2=CC=CC=C2)N	203.2	0.33	0.09	49.6	0	0	0	CHEMBL1165011	1.07	4	1	2	15	-1.06	0.75	1
4-Hydroxydebrisoquine	1	1	1	1	Antihypertensive	C1C(C2=CC=CC=C2CN1C(=N)N)O	191.2	-2.27	12.34	73.3	1	0	0	CHEMBL1710849	0.43	2	3	1	14	0.13	0.41	0
4-Hydroxymexiletine	0	0	1	1	Antiarrhythmic	CC1=CC(=CC(=C1OCC(C)N)C)O	195.3	0.23	9.30	55.5	1	0	0	CHEMBL2018453	1.74	3	2	1	14	0.28	0.77	3
4-Methoxy-m-tyramine	1	0	0		Endobiotic	COC1=C(C=C(C=C1)CCN)O	167.2	-1.31	9.57	55.5	1	0	0	CHEMBL12720	1.15	3	2	1	12	-0.18	0.66	3
4-Methoxyphenethylamine	0	0	0	1	Endobiotic	COC1=CC=C(C=C1)CCN	151.2	-1.06	9.77	35.3	1	0	0	CHEMBL2108676	-1.22	2	1	1	11	N/A	N/A	3
4-Methylaminoantipyrine	0	0	0	0	Analgesic	CC1=C(C(=O)N(N1C)C2=CC=CC=C2)NC	217.3	0.77	1.24	35.6	0	0	0	CHEMBL1164701	1.53	4	1	2	16	-1.4	0.83	2
5,6-Methylenedioxy-2-aminoindane	0	0	0	0	Psychostimulant	C1C(CC2=CC3=C(C=C21)OCO3)N	177.2	-1.27	9.89	44.5	1	0	0	CHEMBL1082953	0.57	2	2	1	13	0.34	0.66	1
5,7-Dihydroxytryptamine	0	0			Experimental chemical	C1=C(C=C(C2=C1C(=CN2)CCN)O)O	192.2	-1.00	10.23	82.3	1	0	0	CHEMBL26726	1.08	3	4	2	14	1.09	0.57	2
5-Aminoindazole	0	0	0		Experimental chemical	C1=CC2=C(C=C1N)C=NN2	133.2	0.47	3.40	54.7	0	0	0	CHEMBL17551	1.15	2	2	2	10	-1.71	0.53	0
5-Aminolevulinic acid	0	0	0	0	Endobiotic	C(CC(=O)O)C(=O)CN	131.1	-3.37	7.84	80.4	0	0	1	CHEMBL601	-0.62	3	2	0	9	0.68	0.53	4
5-Methoxytryptamine	1	0	1	1	Endobiotic	COC1=CC2=C(C=C1)NC=C2CCN	190.2	-0.97	9.78	51.0	1	0	0	CHEMBL8165	1.68	2	2	2	14	0.01	0.77	3
6-beta-Naltrexol	0	0	0	1	Opioid	C1C[C@]2([C@H]3CC4=C5[C@@]2(CCN3CC6CC6)[C@H]([C@@H]1O)OC5=C(C=C4)O)O	343.4	-0.28	8.69	73.2	1	0	0	CHEMBL558140	1.32	5	3	1	25	1.92	0.76	2
6-Hydroxydopamine	0	0		0	Experimental chemical	C1=C(C(=CC(=C1O)O)O)CCN	169.2	-1.42	9.17	86.7	1	0	0	CHEMBL337702	0.3	4	4	1	12	1.31	0.38	2

Substance	MATE1 substrate*	MATE2 substrate*	OCT1 substrate*	OCT2 substrate*	Therapeutic group	SMILES	Molecular weight	logD at pH 7.4	pKa (basic)	TPSA	Net Charge	Quaternary N	Zwitterionic	ChEMBL ID	AlogP	Hydrogen bound	Hydrogen bound	Aromatic rings	Heavy atoms	Natprod likeness score	Rqed (weighted)	Rotable bounds
Abacavir	0	0		0	Antiviral	C1CC1NC2=C3C(=NC(=N2)N)N(C=N3)[C@@H]4C[C@@H](C=C4)CO	286.3	0.35	6.37	101.9	0	0	0	CHEMBL4303288	1.09	7	3	2	21	0.03	0.73	4
Abemaciclib	0	0			Oncology	CCN1CCN(CC1)CC2=CN=C(C=C2)NC3=NC=C(C(=N3)C4=CC5=C(C(=C4)F)N=C(N5C(C)C)C)F	506.6	3.36	8.40	75.0	1	0	0	CHEMBL3301610	4.94	8	1	4	37	-1.56	0.38	7
Abrocitinib	0	0			JAK inhibitor	CCCS(=O)(=O)NC1CC(C1)N(C)C2=NC=NC3=C2C=CN3	323.4	0.46	7.55	99.4	0	0	0	CHEMBL3655081	1.25	5	2	2	22	-1.27	0.83	6
Acebutolol	1	1	1	0	Beta blocker	CCCC(=O)NC1=CC(=C(C=C1)OCC(CNC(C)C)O)C(=O)C	336.4	-0.31	9.25	87.7	1	0	0	CHEMBL642	2.37	5	3	1	24	-0.95	0.57	10
Aceclidine	0	0	1	1	(Anti)cholinergic	CC(=O)OC1CN2CCC1CC2	169.2	-1.48	9.17	29.5	1	0	0	CHEMBL4303558	0.64	3	0	0	12	1.28	0.54	1
Acetylcarnitine	0	0	0	0	Carnitine ester	CC(=O)O[C@H](CC(=O)[O-])C[N+](C)(C)C	203.2	-3.68	14.00	66.4	0	1	1	CHEMBL1697733	-1.24	4	0	0	14	0.98	0.41	5
Aclidinium	1	0	1	1	(Anti)cholinergic	C1C[N+]2(CCC1[C@H](C2)OC(=O)C(C3=CC=CS3)(C4=CC=CS4)O)CCCOC5=CC=CC=C5	484.7	0.46	14.00	112.2	1	1	0	CHEMBL551466	4.67	6	1	3	33	-0.04	0.27	9
Aclidinium (S)	1	0	1		(Anti)cholinergic	C1C[N+]2(CCC1[C@H](C2)OC(=O)C(C3=CC=CS3)(C4=CC=CS4)O)CCCOC5=CC=CC=C5	484.7	0.46	14.00	112.2	1	1	0	CHEMBL551466	4.67	6	1	3	33	-0.04	0.27	9
Aconitine	0	0			Herbal	CCN1C[C@@]2([C@@H](C[C@@H]([C@@]34[C@@H]2[C@H]([C@@H](C31)[C@@]5([C@@H]6[C@H]4C[C@@]([C@@H]6OC(=O)C7=CC=CC=C7)([C@H]([C@@H]5O)OC)O)OC(=O)C)OC)OC)O)COC	645.7	-2.14	8.99	153.5	1	0	0	CHEMBL5314360	0.65	12	3	1	46	3.03	0.32	9
Acyclovir	1	1	0	0	Antiviral	C1=NC2=C(N1COCCO)N=C(NC2=O)N	225.2	-1.55	2.97	114.8	0	0	0	CHEMBL1200380	-1.33	7	3	2	16	-0.1	0.55	4
Agmatine	0	0	0	0	Biogenic amine	C(CCN=C(N)N)CN	130.2	-5.94	12.69	90.4	2	0	0	CHEMBL1256618	-1	2	3	0	9	1.46	0.26	4
Agomelatine	0	0			Antidepressant	CC(=O)NCCC1=CC=CC2=C1C=C(C=C2)OC	243.3	2.04	-1.60	38.3	0	0	0	CHEMBL10878	2.53	2	1	2	18	-0.52	0.9	4

Substance	MATE1 substrate*	MATE2 substrate*	OCT1 substrate*	OCT2 substrate*	Therapeutic group	SMILES	Molecular weight	logD at pH 7.4	pKa (basic)	TPSA	Net Charge	Quarternary N	Zwitterionic	ChEMBL ID	AlogP	Hydrogen bound	Hydrogen bound	Aromatic rings	Heavy atoms	Natprod likeness score	Rqed (weighted)	Rotable bounds
Alfuzosine	0	0	0	0	Alpha1 blocker	CN(CCCNC(=O)C1CCCO1)C2=NC3=CC(=C(C=C3C(=N2)N)OC)OC	389.5	0.43	8.10	111.8	0	0	0	CHEMBL1723	1.35	8	2	2	28	-0.8	0.65	8
Aliskiren	0	0	0	0	Antihypertensive	CC(C)[C@@H](CC1=CC(=C(C=C1)OC)OCCCOC)C[C@@H]([C@H](C[C@@H](C(C)C)C(=O)NCC(C)(C)C(=O)N)O)N	551.8	1.00	9.57	146.1	1	0	0	CHEMBL1639	3.29	7	4	1	39	0.21	0.19	19
Alizapride	1	1	0	1	Others	COC1=CC2=C(C=C1C(=O)NCC3CCCN3CC=C)NN=N2	315.4	0.26	8.58	83.1	1	0	0	CHEMBL1896987	1.35	5	2	2	23	-1.34	0.79	6
Allopurinol	0	0			Others	C1=NNC2=C1C(=O)NC=N2	136.1	-0.90	2.02	70.1	0	0	0	CHEMBL1467	0.06	4	2	2	10	-1.78	0.54	0
Almotriptan	1	1	1	1	Triptan	CN(C)CCC1=CNC2=C1C=C(C=C2)CS(=O)(=O)N3CCCC3	335.5	-0.63	9.57	64.8	1	0	0	CHEMBL1505	2.2	3	1	2	23	-1.25	0.88	6
Alpha-methylnorepinephrine (-)	0	0		0	Sympathomimetic	CC([C@@H](C1=CC(=C(C=C1)O)O)O)N	183.2	-1.55	8.93	86.7	1	0	0	CHEMBL1256471	0.48	4	4	1	13	1.36	0.5	2
Alprenolol	0	0	0	0	Beta blocker	CC(C)NCC(COC1=CC=CC=C1CC=C)O	249.4	0.84	9.27	41.5	1	0	0	CHEMBL1256179	2.15	3	2	1	18	-0.24	0.69	8
Amantadine	0	0	0	1	Antiviral	C1C2CC3CC1CC(C2)(C3)N	151.3	-1.28	10.46	26.0	1	0	0	CHEMBL660	1.91	1	1	0	11	0.55	0.56	0
Ambroxol	0	0	0	0	Others	C1CC(CCC1NCC2=C(C(=CC(=C2)Br)Br)N)O	378.1	1.04	9.01	58.3	1	0	0	CHEMBL153479	3.19	3	3	1	18	-0.34	0.71	3
Amifampridine	1	1	1	1	Others	C1=CN=CC(=C1N)N	109.1	-1.84	9.25	64.9	1	0	0	CHEMBL3301611	0.25	3	2	1	8	-0.66	0.5	0
Amiloride	1	1	1	1	Diuretic	C1(=C(N=C(C(=N1)Cl)N)N)C(=O)N=C(N)N	229.6	-1.14	7.29	159.3	1	0	0	CHEMBL945	-1.08	6	5	1	15	-0.47	0.3	1
Amiodarone	0	1	0	0	Antiarrhythmic	CCCCC1=C(C2=CC=CC=C2O1)C(=O)C3=CC(=C(C(=C3)I)OCCN(CC)CC)I	645.3	5.95	9.08	42.7	1	0	0	CHEMBL1083993	6.94	4	0	3	31	-0.38	0.17	11
Amisulpride	1	1	1	0	Antipsychotic	CCN1CCCC1CNC(=O)C2=CC(=C(C=C2OC)N)S(=O)(=O)CC	369.5	-0.68	8.28	110.1	1	0	0	CHEMBL243712	1.29	6	2	1	25	-1.38	0.7	7
Amisulpride (R)	1	1	1	1	Antipsychotic	CCN1CCCC1CNC(=O)C2=CC(=C(C=C2OC)N)S(=O)(=O)CC	369.5	-0.68	8.28	110.1	1	0	0	CHEMBL243712	1.29	6	2	1	25	-1.38	0.7	7

Substance	MATE1 substrate*	MATE2 substrate*	OCT1 substrate*	OCT2 substrate*	Therapeutic group	SMILES	Molecular weight	logD at pH 7.4	pKa (basic)	TPSA	Net Charge	Quaternary N	Zwitterionic	ChEMBL ID	AlogP	Hydrogen bound	Hydrogen bound	Aromatic rings	Heavy atoms	Natprod likeness score	Rqed (weighted)	Rotable bounds
Amisulpride (S)	1	1	1	0	Antipsychotic	CCN1CCCC1CNC(=O)C2=CC(=C(C=C2OC)N)S(=O)(=O)CC	369.5	-0.68	8.28	110.1	1	0	0	CHEMBL243712	1.29	6	2	1	25	-1.38	0.7	7
Amitriptyline	0	0	0	0	Antidepressant	CN(C)CCC=C1C2=CC=CC=C2CCC3=CC=CC=C31	277.4	3.15	9.06	3.2	1	0	0	CHEMBL1200964	4.17	1	0	2	21	-0.11	0.81	3
Amlodipine	0	0	0	0	Antihypertensive	CCOC(=O)C1=C(NC(=C(C1C2=CC=CC=C2Cl)C(=O)OC)C)COCCN	408.9	-0.24	9.28	99.9	1	0	0	CHEMBL1200984	2.27	7	2	1	28	-0.78	0.5	8
Amoxapine	0	0	0	0	Antidepressant	C1CN(CCN1)C2=NC3=CC=CC=C3OC4=C2C=C(C=C4)Cl	313.8	1.64	8.83	36.9	1	0	0	CHEMBL1113	3.43	4	1	2	22	-0.58	0.81	0
Amoxicillin	0	0			Antibiotic	CC1([C@@H](N2[C@H](S1)[C@@H](C2=O)NC(=O)[C@@H](C3=CC=C(C=C3)O)N)C(=O)O)C	365.4	-2.67	7.22	158.3	-1	0	0	CHEMBL2105950	0.02	6	4	1	25	0.94	0.55	4
Amphetamine	0	0	0	1	Psychostimulant	CC(CC1=CC=CC=C1)N	135.2	-0.69	10.04	26.0	1	0	0	CHEMBL405	1.58	1	1	1	10	-0.02	0.65	2
APC366	1	0		0	Others	C1C[C@H](N(C1)C(=O)[C@H](CCCN=C(N)N)NC(=O)C2=C(C3=CC=CC=C3C=C2)O)C(=O)N	440.5	-0.18	11.37	177.1	1	0	0	CHEMBL423874	0.38	5	6	2	32	-0.24	0.2	8
Apixaban	0	0			Anticoagulant	COC1=CC=C(C=C1)N2C3=C(CCN(C3=O)C4=CC=C(C=C4)N5CCCCC5=O)C(=N2)C(=O)N	459.5	1.83	-1.60	110.8	0	0	0	CHEMBL231779	2.7	6	1	3	34	-1.04	0.63	5
Apomorphine	0	0	0	0	Dopamine agonist	CN1CCC2=C3[C@H]1CC4=C(C3=CC=C2)C(=C(C=C4)O)O	267.3	2.59	7.73	43.7	1	0	0	CHEMBL3187985	2.85	3	2	2	20	1.45	0.72	0
Arcaine	1	1	1	1	Others	C(CCN=C(N)N)CN=C(N)N	172.2	-6.45	12.41	128.8	2	0	0	CHEMBL1256017	-1.27	2	6	0	12	0.12	0.18	5
Aripiprazole	0	0			Antipsychotic	C1CC(=O)NC2=C1C=CC(=C2)OCCCCN3CCN(CC3)C4=C(C(=CC=C4)Cl)Cl	448.4	3.26	9.04	44.8	1	0	0	CHEMBL1112	4.86	4	1	2	30	-1.16	0.61	7
Articaine	0	0			Local anesthetic	CCCNC(C)C(=O)NC1=C(SC=C1C)C(=O)OC	284.4	1.94	8.68	67.4	1	0	0	CHEMBL1316368	2.17	5	2	1	19	-1.23	0.79	6
Aspartame	0	0	0	1	Others	COC(=O)[C@H](CC1=CC=CC=C1)NC(=O)[C@H](CC(=O)O)N	294.3	-2.25	8.53	118.7	0	0	1	CHEMBL171679	-0.31	5	3	1	21	0.26	0.59	7

Substance	MATE1 substrate*	MATE2 substrate*	OCT1 substrate*	OCT2 substrate*	Therapeutic group	SMILES	Molecular weight	logD at pH 7.4	pKa (basic)	TPSA	Net Charge	Quaternary N	Zwitterionic	ChEMBL ID	AlogP	Hydrogen bound	Hydrogen bound	Aromatic rings	Heavy atoms	Natprod likeness score	Rqed (weighted)	Rotable bounds
Atenolol	1	1	1	1	Beta blocker	CC(C)NCC(COC1=CC=C(C=C1)CC(=O)N)O	266.3	-1.43	9.27	84.6	1	0	0	CHEMBL24	0.45	4	3	1	19	-0.62	0.64	8
Atenolol (R)	1	1	1	1	Beta blocker	CC(C)NCC(COC1=CC=C(C=C1)CC(=O)N)O	266.3	-1.43	9.27	84.6	1	0	0	CHEMBL1230004	0.45	4	3	1	19	-0.62	0.64	8
Atenolol (S)	1	1	1	1	Beta blocker	CC(C)NCC(COC1=CC=C(C=C1)CC(=O)N)O	266.3	-1.43	9.27	84.6	1	0	0	CHEMBL1230004	0.45	4	3	1	19	-0.62	0.64	8
Atomoxetine	0	0	0	0	Antidepressant	CC1=CC=CC=C1O[C@H](CCNC)C2=CC=CC=C2	255.4	1.83	9.40	21.3	1	0	0	CHEMBL1702	3.72	2	1	2	19	-0.06	0.85	6
Atomoxetine (S)	0	0	0		Antidepressant	CC1=CC=CC=C1O[C@H](CCNC)C2=CC=CC=C2	255.4	1.83	9.40	21.3	1	0	0	CHEMBL1702	3.72	2	1	2	19	-0.06	0.85	6
Atracurium	0	0			Muscle relaxant	C[N+]1(CCC2=CC(=C(C=C2C1CC3=CC(=C(C=C3)OC)OC)OC)OC)CCC(=O)OCCCCCOC(=O)CC[N+]4(CCC5=CC(=C(C=C5C4CC6=CC(=C(C=C6)OC)OC)OC)OC)C	929.2	-0.96	14.00	126.4	2	1	0	CHEMBL1200527	8.07	12	0	4	67	0.36	0.04	24
Atropine	1	0	1	1	(Anti)cholinergic	CN1[C@@H]2CC[C@H]1CC(C2)OC(=O)C(CO)C3=CC=CC=C3	289.4	-0.21	9.19	49.8	1	0	0	CHEMBL4557433	1.93	4	1	1	21	0.79	0.86	4
Baclofen	0	0			Others	C1=CC(=CC=C1C(CC(=O)O)CN)Cl	213.7	-0.78	9.72	63.3	0	0	1	CHEMBL1897150	1.86	2	2	1	14	0.05	0.8	4
Bambuterol	0	0	0	0	Sympathomimetic	CC(C)(C)NCC(C1=CC(=CC(=C1)OC(=O)N(C)C)OC(=O)N(C)C)O	367.4	-0.70	9.52	91.3	1	0	0	CHEMBL521589	2.23	6	2	1	26	-0.24	0.83	5
Baricitinib	0	0	0		JAK inhibitor	CCS(=O)(=O)N1CC(C1)(CC#N)N2C=C(C=N2)C3=C4C=CNC4=NC=N3	371.4	-0.19	5.01	128.9	0	0	0	CHEMBL2105759	1.1	7	1	3	26	-1.11	0.72	5
Befunolol	0	0	0	0	Beta blocker	CC(C)NCC(COC1=CC=CC2=C1OC(=C2)C(=O)C)O	291.3	-0.61	9.26	71.7	1	0	0	CHEMBL153984	2.37	5	2	2	21	-0.23	0.77	7
Benzyltriethylammonium	1	1	1	1	Experimental chemical	CC[N+](CC)(CC)CC1=CC=CC=C1	192.3	-1.18	14.00	0.0	1	1	0	CHEMBL1231492	3.06	0	0	1	14	-0.16	0.63	5
Berberine	1	1	1	1	Herbal	COC1=C(C2=C[N+]3=C(C=C2C=C1)C4=CC5=C(C=C4CC3)OCO5)OC	336.4	-1.28	14.00	40.8	1	1	0	CHEMBL295124	3.1	4	0	3	25	1.3	0.67	2
Betahistine	0	0			Antihistaminic	CNCCC1=CC=CC=N1	136.2	-1.68	9.77	24.9	1	0	0	CHEMBL24441	0.84	2	1	1	10	-1.3	0.67	3

Substance	MATE1 substrate*	MATE2 substrate*	OCT1 substrate*	OCT2 substrate*	Therapeutic group	SMILES	Molecular weight	logD at pH 7.4	pKa (basic)	TPSA	Net Charge	Quarternary N	Zwitterionic	ChEMBL ID	AlogP	Hydrogen bound	Hydrogen bound	Aromatic rings	Heavy atoms	Natprod likeness score	Rqed (weighted)	Rotable bounds
Betaine	0	0	0		Endobiotic	C[N+](C)(C)CC(=O)[O-]	117.2	-3.72	14.00	40.1	0	1	1	CHEMBL95889	-0.22	1	1	0	8	0.88	0.51	2
Betaxolol	0	0	0	0	Beta blocker	CC(C)NCC(COC1=CC=C(C=C1)CCOCC2CC2)O	307.4	0.68	9.27	50.7	1	0	0	CHEMBL1691	2.39	4	2	1	22	-0.56	0.62	11
Bethanechol	0	0	0	0	(Anti)cholinergic	CC(C[N+](C)(C)C)OC(=O)N	161.2	-4.14	14.00	52.3	1	1	0	CHEMBL1482	0.18	2	1	0	11	0.89	0.6	3
Bicifadine	0	0	0	1	Antidepressant	CC1=CC=C(C=C1)C23CC2CNC3	173.3	-0.11	9.60	12.0	1	0	0	CHEMBL544862	1.86	1	1	1	13	0.78	0.68	1
Biotin	0	0			Vitamin	C1[C@H]2[C@@H]([C@@H](S1)CCCCC(=O)O)NC(=O)N2	244.3	-2.57	-1.90	103.7	-1	0	0	CHEMBL857	0.8	3	3	0	16	0.02	0.49	5
Biperiden	0	0	0	0	(Anti)cholinergic	C1CCN(CC1)CCC(C2CC3CC2C=C3)(C4=CC=CC=C4)O	311.5	1.52	9.43	23.5	1	0	0	CHEMBL1201035	3.96	2	1	1	23	-0.28	0.83	5
Bisnorephedrine	0	0	0	1	Sympathomimetic	C1=CC=C(C=C1)C(CN)O	137.2	-1.22	9.10	46.3	1	0	0	#NV	-1.03	2	2	1	10	#NV	#NV	2
Bisnorephedrine (R)	0	0			Experimental chemical	C1=CC=C(C=C1)C(CN)O	137.2	-1.22	9.10	46.3	1	0	0	CHEMBL89731	-1.03	2	2	1	10	0.26	0.41	2
Bisoprolol	0	0	0	0	Beta blocker	CC(C)NCC(COC1=CC=C(C=C1)COCCOC(C)C)O	325.4	0.34	9.27	60.0	1	0	0	CHEMBL645	2.37	5	2	1	23	-0.86	0.58	12
Bitolterol	0	0	0	1	Beta blocker	CC1=CC=C(C=C1)C(=O)OC2=C(C=C(C=C2)C(CNC(C)(C)C)O)OC(=O)C3=CC=C(C=C3)C	461.6	4.15	9.59	84.9	1	0	0	CHEMBL1201295	5.16	6	2	3	34	-0.19	0.37	7
Bortezomib	0	0			Oncology	B([C@H](CC(C)C)NC(=O)[C@H](CC1=CC=CC=C1)NC(=O)C2=NC=CN=C2)(O)O	384.2	1.51	0.70	124.4	0	0	0	CHEMBL325041	1.51	6	4	2	28			9
Brofaromine	0	0	0	0	MAO inhibitor	COC1=CC(=C2C(=C1)C=C(O2)C3CCNCC3)Br	310.2	0.33	9.82	34.4	1	0	0	CHEMBL160347	3.67	3	1	2	18	0.28	0.92	2
Bromocriptine	0	0	0	0	Dopamine agonist	CC(C)C[C@H]1C(=O)N2CCC[C@H]2[C@]3(N1C(=O)[C@](O3)(C(C)C)NC(=O)[C@H]4CN([C@@H]5CC6=C(NC7=CC=CC(=C67)C5=C4)Br)C)O	654.6	3.74	7.00	118.2	0	0	0	CHEMBL1255752	3.19	6	3	2	43	1.29	0.46	5

Substance	MATE1 substrate*	MATE2 substrate*	OCT1 substrate*	OCT2 substrate*	Therapeutic group	SMILES	Molecular weight	logD at pH 7.4	pKa (basic)	TPSA	Net Charge	Quaternary N	Zwitterionic	ChEMBL ID	AlogP	Hydrogen bound	Hydrogen bound	Aromatic rings	Heavy atoms	Natprod likeness score	Rqed (weighted)	Rotable bounds
Brucine	0	0		0	Herbal	COC1=C(C=C2C(=C1)[C@]34CCN5[C@H]3C[C@@H]6[C@@H]7[C@@H]4N2C(=O)C[C@@H]7OCC=C6C5)OC	394.5	-0.56	8.55	51.2	1	0	0	CHEMBL501756	2.11	5	0	1	29	2.77	0.72	2
Buformin	1	1	1	1	Antidiabetic	CCCCN=C(N)N=C(N)N	157.2	-4.31	12.49	102.8	2	0	0	CHEMBL39736	-0.21	2	5	0	11	-0.03	0.22	3
Bumetanide	1	0	0	0	Diuretic	CCCCNC1=C(C(=CC(=C1)C(=O)O)S(=O)(=O)N)OC2=CC=CC=C2	364.4	-0.67	1.84	127.1	-1	0	0	CHEMBL1072	3.04	5	3	2	25	-0.97	0.62	8
Bunitrolol	0	0	1	1	Beta blocker	CC(C)(C)NCC(COC1=CC=CC=C1C#N)O	248.3	-0.21	9.36	65.3	1	0	0	CHEMBL418134	1.69	4	2	1	18	-1.11	0.83	5
Bupivacaine	0	0	0	0	Local anesthetic	CCCCN1CCCCC1C(=O)NC2=C(C=CC=C2C)C	288.4	3.83	7.98	32.3	1	0	0	CHEMBL1098	3.9	2	1	1	21	-1.36	0.89	5
Bupivacaine (R)	0	0			Local anesthetic	CCCCN1CCCCC1C(=O)NC2=C(C=CC=C2C)C	288.4	3.83	7.98	32.3	1	0	0	CHEMBL1200396	3.9	2	1	1	21	-1.36	0.89	5
Bupivacaine (S)	0	0	0	1	Local anesthetic	CCCCN1CCCCC1C(=O)NC2=C(C=CC=C2C)C	288.4	3.83	7.98	32.3	1	0	0	CHEMBL1200396	3.9	2	1	1	21	-1.36	0.89	5
Bupropion	0	0	0	0	Antidepressant	CC(C(=O)C1=CC(=CC=C1)Cl)NC(C)(C)C	239.7	2.39	8.22	29.1	1	0	0	CHEMBL1201735	3.3	2	1	1	16	-0.88	0.82	3
Buspirone	1	0	1	0	Others	C1CCC2(C1)CC(=O)N(C(=O)C2)CCCCN3CCN(CC3)C4=NC=CC=N4	385.5	0.61	8.54	69.6	1	0	0	CHEMBL1200399	2.09	6	0	1	28	-1.1	0.55	6
Butylscopolamine	1	0	1	1	(Anti)cholinergic	CCCC[N+]1([C@@H]2CC(C[C@H]1[C@H]3[C@@H]2O3)OC(=O)[C@H](CO)C4=CC=CC=C4)C.[Br-]	360.5	-1.94	14.00	59.1	1	1	0	CHEMBL1618102	2.23	4	1	1	26	1	0.46	7
Butyrylcarnitine	0	0	0		Carnitine ester	CCCC(=O)O[C@H](CC(=O)[O-])C[N+](C)(C)C	231.3	-2.53	14.00	66.4	0	0	1	CHEMBL2074693	-0.46	4	0	0	16	0.65	0.44	7
Cabergoline	0	0	0	0	Dopamine agonist	CCNC(=O)N(CCCN(C)C)C(=O)[C@@H]1C[C@H]2[C@@H](CC3=CNC4=CC=CC2=C34)N(C1)CC=C	451.6	0.28	9.31	71.7	2	0	0	CHEMBL1201087	3.19	4	2	2	33	0.03	0.6	8
Cadaverine	0	0	0	0	Biogenic amine	C(CCN)CCN	102.2	-5.61	10.51	52.0	2	0	0	CHEMBL3351096	0.07	2	2	0	7	0.64	0.49	4
Caffeine	0	0	0	0	Xanthine derivative	CN1C=NC2=C1C(=O)N(C(=O)N2C)C	194.2	-0.55	-1.20	58.4	0	0	0	CHEMBL113	-1.03	6	0	2	14	-1.09	0.54	0

Substance	MATE1 substrate*	MATE2 substrate*	OCT1 substrate*	OCT2 substrate*	Therapeutic group	SMILES	Molecular weight	logD at pH 7.4	pKa (basic)	TPSA	Net Charge	Quaternary N	Zwitterionic	ChEMBL ID	AlogP	Hydrogen bound	Hydrogen bound	Aromatic rings	Heavy atoms	Natprod likeness score	Rqed (weighted)	Rotable bounds
Capsaicin	0	0			Herbal	CC(C)/C=C/CCCCC(=O)NCC1=CC(=C(C=C1)O)OC	305.4	3.75	0.37	58.6	0	0	0	CHEMBL294199	3.79	3	2	1	22	0.54	0.54	9
Captopril	0	0	0	0	Antihypertensive	C[C@H](CS)C(=O)N1CCC[C@H]1C(=O)O	217.3	-2.64	0.88	96.4	-1	0	0	CHEMBL434965	0.63	3	2	0	14	0.11	0.68	3
Carnitine	0	0	0	0	Endobiotic	C[N+](C)(C)C[C@@H](CC(=O)[O-])O	161.2	-4.12	14.00	60.4	0	1	1	CHEMBL172513	-1.81	3	1	0	11	0.61	0.49	4
Carnosine	0	0	0		Endobiotic	C1=C(NC=N1)C[C@@H](C(=O)O)NC(=O)CCN	226.2	-4.51	9.13	121.1	1	0	0	CHEMBL242948	-1.13	4	4	1	16	0.01	0.49	6
Carteolol	1	1	1	1	Beta blocker	CC(C)(C)NCC(COC1=CC=CC2=C1CCC(=O)N2)O	292.4	-0.52	9.36	70.6	1	0	0	CHEMBL1201002	1.7	4	3	1	21	-0.34	0.77	5
Carvedilol (R)	0	0		0	Beta blocker	COC1=CC=CC=C1OCCNCC(COC2=CC=CC3=C2C4=CC=CC=C4N3)O	406.5	2.92	7.74	75.7	1	0	0	CHEMBL1201167	3.74	5	3	4	30	-0.12	0.35	10
Carvedilol (S)	0	0		0	Beta blocker	COC1=CC=CC=C1OCCNCC(COC2=CC=CC3=C2C4=CC=CC=C4N3)O	406.5	2.92	7.74	75.7	1	0	0	CHEMBL1201167	3.74	5	3	4	30	-0.12	0.35	10
Cathine	0	0	0	1	Psychostimulant	C[C@@H]([C@H](C1=CC=CC=C1)O)N	151.2	-1.03	9.35	46.3	1	0	0	CHEMBL1412041	1.07	2	2	1	11	0.57	0.66	2
Cathinone	0	0	0	1	Psychostimulant	C[C@@H](C(=O)C1=CC=CC=C1)N	149.2	0.83	7.48	43.1	1	0	0	CHEMBL2104047	1.22	2	1	1	11	-0.08	0.64	2
Celiprolol	1	1	0	0	Beta blocker	CCN(CC)C(=O)NC1=CC(=C(C=C1)OCC(CNC(C)(C)C)O)C(=O)C	379.5	-0.43	9.35	90.9	1	0	0	CHEMBL1742424	2.89	5	3	1	27	-1.22	0.57	9
Cetirizine	0	0			Antihistaminic	C1CN(CCN1CCOCC(=O)O)C(C2=CC=CC=C2)C3=CC=C(C=C3)Cl	388.9	0.78	7.97	53.0	0	0	1	CHEMBL1607273	3.15	4	1	2	27	-1.21	0.7	8
Cetirizine (L)	0	0	0	0	Antihistaminic	C1CN(CCN1CCOCC(=O)O)C(C2=CC=CC=C2)C3=CC=C(C=C3)Cl	388.9	0.78	7.97	53.0	0	0	1	CHEMBL1607273	3.15	4	1	2	27	-1.21	0.7	8
Cevimeline	0	0	1	1	(Anti)cholinergic	C[C@@H]1O[C@]2(CN3CCC2CC3)CS1	199.3	-0.22	8.59	37.8	1	0	0	CHEMBL168815	1.56	3	0	0	13	1.15	0.59	0
Chloramphenicol	0	0			Antibiotic	C1=CC(=CC=C1[C@H]([C@@H](CO)NC(=O)C(Cl)Cl)O)[N+](=O)[O-]	323.1	0.88	14.00	112.7	0	1	1	CHEMBL130	0.91	5	3	1	20	-0.11	0.41	6
Chlorhexidine	0	0	0	0	Antiinfective	C1=CC(=CC=C1N/C(=N/C(=NCCCCCCN=C(/N=C(/NC2=CC=C(C=C2)Cl)\N)N)N)/N)Cl	505.5	-1.33	11.97	177.6	2	0	0	CHEMBL1628	4.18	4	10	2	34	-0.36	0.14	9

Substance	MATE1 substrate*	MATE2 substrate*	OCT1 substrate*	OCT2 substrate*	Therapeutic group	SMILES	Molecular weight	logD at pH 7.4	pKa (basic)	TPSA	Net Charge	Quaternary N	Zwitterionic	ChEMBL ID	AlogP	Hydrogen bound	Hydrogen bound	Aromatic rings	Heavy atoms	Natprod likeness score	Rqed (weighted)	Rotable bounds
Chloroquine	0	0	0	0	Antiinfective	CCN(CC)CCCC(C)NC1=C2C=CC(=CC2=NC=C1)Cl	319.9	0.47	10.92	28.2	1	0	0	CHEMBL76	4.81	3	1	2	22	-1.27	0.76	8
Chlorpheniramine	0	0	0	1	Antihistaminic	CN(C)CCC(C1=CC=C(C=C1)Cl)C2=CC=CC=N2	274.8	1.52	9.47	16.1	1	0	0	CHEMBL180454	3.82	2	0	2	19	-1.38	0.82	5
Chlorpromazine	0	0	0	0	Antipsychotic	CN(C)CCCN1C2=CC=CC=C2SC3=C1C=C(C=C3)Cl	318.9	2.74	9.20	31.8	1	0	0	CHEMBL71	4.89	3	0	2	21	-1.54	0.79	4
Chlorprothixene	0	0	0	0	Antipsychotic	CN(C)CC/C=C\1/C2=CC=CC=C2SC3=C1C=C(C=C3)Cl	315.9	3.40	9.06	28.5	1	0	0	CHEMBL1256658	5.19	2	0	2	21	-0.83	0.65	3
Choline	0	0	0	0	Endobiotic	C[N+](C)(C)CCO	104.2	-4.66	14.00	20.2	1	1	0	CHEMBL920	-0.32	1	1	0	7	1.44	0.48	2
Cilastatin	0	0			Others	CC1(C[C@@H]1C(=O)N/C(=C\CCCCSC[C@@H](C(=O)O)N)/C(=O)O)C	358.5	-4.06	9.14	155.0	-1	0	1	CHEMBL1201057	1.43	5	4	0	24	0.74	0.32	11
Cimetidine	1	1	1	1	Antihistaminic	CC1=C(N=CN1)CSCCNC(=NC)NC#N	252.3	-0.41	7.11	114.2	0	0	0	CHEMBL1201051	0.6	4	3	1	17	-1.18	0.23	5
Cinchonidine	0	0	0	0	Herbal	C=C[C@H]1CN2CC[C@H]1C[C@H]2[C@@H](C3=CC=NC4=CC=CC=C34)O	294.4	0.92	9.15	36.4	1	0	0	CHEMBL533841	3.16	3	1	2	22	0.88	0.88	3
Ciprofloxacin	1	0	0	0	Antibiotic	C1CC1N2C=C(C(=O)C3=CC(=C(C=C32)N4CCNCC4)F)C(=O)O	331.3	-0.76	8.69	72.9	0	0	1	CHEMBL8	1.58	5	2	2	24	-0.72	0.89	3
Citalopram	0	0	0	0	Antidepressant	CN(C)CCCC1(C2=C(CO1)C=C(C=C2)C#N)C3=CC=C(C=C3)F	324.4	0.89	10.38	36.3	1	0	0	CHEMBL1200781	3.81	3	0	2	24	-0.47	0.84	5
Citalopram (R)	0	0			Antidepressant	CN(C)CCCC1(C2=C(CO1)C=C(C=C2)C#N)C3=CC=C(C=C3)F	324.4	0.89	10.38	36.3	1	0	0	CHEMBL1200781	3.81	3	0	2	24	-0.47	0.84	5
Citalopram (S)	0	0	0	0	Antidepressant	CN(C)CCCC1(C2=C(CO1)C=C(C=C2)C#N)C3=CC=C(C=C3)F	324.4	0.89	10.38	36.3	1	0	0	CHEMBL1200781	3.81	3	0	2	24	-0.47	0.84	5
Citrulline	0	0	0	0	Endobiotic	C(C[C@@H](C(=O)O)N)CNC(=O)N	175.2	-3.94	9.23	118.4	0	0	1	CHEMBL444814	-1.15	3	4	0	12	0.24	0.39	5
Clarithromycin	0	0	0	0	Antibiotic	CC[C@@H]1[C@@]([C@@H]([C@H](C(=O)[C@@H](C[C@@]([C@@	748.0	1.63	9.00	182.9	1	0	0	CHEMBL3937847	2.44	14	4	0	52	1.77	0.27	8

Substance	MATE1 substrate*	MATE2 substrate*	OCT1 substrate*	OCT2 substrate*	Therapeutic group	SMILES	Molecular weight	logD at pH 7.4	pKa (basic)	TPSA	Net Charge	Quaternary N	Zwitterionic	ChEMBL ID	AlogP	Hydrogen bound	Hydrogen bound	Aromatic rings	Heavy atoms	Natprod likeness score	Rqed (weighted)	Rotable bounds
						H]([C@H]([C@@H]([C@H](C(=O)O1)C)O[C@H]2C[C@@]([C@H]([C@@H](O2)C)O)(C)OC)C)O[C@H]3[C@@H]([C@H](C[C@H](O3)C)N(C)C)O)(C)OC)C)C)O)(C)O																
Clemastine	0	0			Antihistaminic	C[C@@](C1=CC=CC=C1)(C2=CC=C(C=C2)Cl)OCC[C@H]3CCCN3C	343.9	2.79	9.55	12.5	1	0	0	CHEMBL1200795	5.1	2	0	2	24	-0.4	0.72	6
Clenbuterol	0	0	0	0	Symphatomimetic	CC(C)(C)NCC(C1=CC(=C(C(=C1)Cl)N)Cl)O	277.2	0.14	9.63	58.3	1	0	0	CHEMBL1330729	3	3	3	1	17	-0.32	0.74	3
Clidinium	1	0	1	1	(Anti)cholinergic	C[N+]12CCC(CC1)C(C2)OC(=O)C(C3=CC=CC=C3)(C4=CC=CC=C4)O	352.5	-1.08	14.00	46.5	1	1	0	CHEMBL620	2.7	3	1	2	26	0.4	0.68	4
Clindamycin	0	0			Antibiotic	CCC[C@@H]1C[C@H](N(C1)C)C(=O)N[C@@H]([C@@H]2[C@@H]([C@@H]([C@H]([C@H](O2)SC)O)O)O)[C@H](C)Cl	425.0	0.65	7.55	127.6	1	0	0	CHEMBL1200588	0.39	7	4	0	27	0.6	0.44	7
Clomipramine	0	0			Antidepressant	CN(C)CCCN1C2=CC=CC=C2CCC3=C1C=C(C=C3)Cl	314.9	3.09	9.20	6.5	1	0	0	#NV	2.72	2	0	2	22	#NV	#NV	4
Clonidine	0	0	0	1	Alpha2 agonist	C1CN=C(N1)NC2=C(C=CC=C2Cl)Cl	230.1	0.16	10.47	36.4	1	0	0	CHEMBL1705	2.36	3	2	1	14	-0.82	0.78	1
Clozapine	0	0	0	0	Antipsychotic	CN1CCN(CC1)C2=NC3=C(C=CC(=C3)Cl)NC4=CC=CC=C42	326.8	2.57	8.16	30.9	1	0	0	CHEMBL42	3.72	4	1	2	23	-0.78	0.8	0
Cocaine	0	0	0	0	Psychostimulant	CN1[C@H]2CC[C@@H]1[C@H]([C@H](C2)OC(=O)C3=CC=CC=C3)C(=O)OC	303.4	0.73	8.95	55.8	1	0	0	CHEMBL370805	1.87	5	0	1	22	0.93	0.8	3
Codeine	0	0	0	0	Opioid	CN1CC[C@]23[C@@H]4[C@H]1CC5=C2C(=C(C=C5)OC)O[C@H]3[C@H](C=C4)O	299.4	-0.16	8.89	41.9	1	0	0	CHEMBL1201099	1.5	4	1	1	22	2.27	0.8	1
Colterol	1	1	1	1	Beta blocker	CC(C)(C)NCC(C1=CC(=C(C=C1)O)O)O	225.3	-0.78	8.98	72.7	1	0	0	CHEMBL2106067	1.52	4	4	1	16	0.47	0.59	3

Substance	MATE1 substrate*	MATE2 substrate*	OCT1 substrate*	OCT2 substrate*	Therapeutic group	SMILES	Molecular weight	logD at pH 7.4	pKa (basic)	TPSA	Net Charge	Quaternary N	Zwitterionic	ChEMBL ID	AlogP	Hydrogen bound	Hydrogen bound	Aromatic rings	Heavy atoms	Natprod likeness score	Rqed (weighted)	Rotable bounds
Coluracetam	0	0			Others	CC1=C(OC2=NC3=C(CCCC3)C(=C12)NC(=O)CN4CCCC4=O)C	341.4	1.90	1.23	75.4	0	0	0	CHEMBL37935	2.88	4	1	2	25	-1.38	0.93	3
Coptisine	1	1	1	1	Herbal	C1C[N+]2=C(C=C3C=CC4=C(C3=C2)OCO4)C5=CC6=C(C=C51)OCO6	320.3	-1.34	14.00	40.8	1	1	0	CHEMBL498722	2.81	4	0	3	24	1.44	0.6	0
Corticosterone	0	0	0	1	Steroid hormone	C[C@]12CCC(=O)C=C1CC[C@@H]3[C@@H]2[C@H](C[C@]4([C@H]3CC[C@@H]4C(=O)CO)C)O	346.5	2.02	-0.26	74.6	0	0	0	CHEMBL110739	2.67	4	2	0	25	2.6	0.81	2
Cotinine	0	0	0	0	Herbal	CN1[C@@H](CCC1=O)C2=CN=CC=C2	176.2	0.21	4.79	33.2	0	0	0	CHEMBL578211	1.37	2	0	1	13	-0.42	0.65	1
Creatine	0	0	0	0	Endobiotic	CN(CC(=O)O)C(=N)N	131.1	-2.86	12.98	90.4	0	0	1	CHEMBL283800	-1.1	2	3	0	9	0.32	0.33	2
Creatinine	0	0	0	0	Endobiotic	CN1CC(=O)N=C1N	113.1	-1.98	4.96	58.7	0	0	0	CHEMBL65567	-1.02	2	2	0	8	-0.32	0.42	0
Crizotinib	0	0	0	0	Oncology	C[C@H](C1=C(C=CC(=C1Cl)F)Cl)OC2=C(N=CC(=C2)C3=CN(N=C3)C4CCNCC4)N	450.3	1.00	10.06	78.0	1	0	0	CHEMBL601719	5.04	6	2	3	30	-0.99	0.53	5
Crotaline	0	0			Herbal	C[C@H]1C(=O)O[C@@H]2CCN3[C@@H]2C(=CC3)COC(=O)[C@]([C@]1(C)O)(C)O	325.4	-0.37	6.38	96.3	1	0	0	CHEMBL521035	-0.39	7	2	0	23	2.65	0.46	0
Cycloguanil	1	1	1	1	Antiinfective	CC1(N=C(N=C(N1C2=CC=C(C=C2)Cl)N)N)C	251.7	-0.28	10.65	80.0	1	0	0	CHEMBL3989825	1.53	5	2	1	17	-0.61	0.79	1
Cyclopentolate	0	0	0	0	(Anti)cholinergic	CN(C)CCOC(=O)C(C1=CC=CC=C1)C2(CCCC2)O	291.4	1.26	8.42	49.8	1	0	0	CHEMBL1201338	2.18	4	1	1	21	0.11	0.82	6
Cyclophosphamide	0	0	0	0	Cytostatic	C1CNP(=O)(OC1)N(CCCl)CCCl	261.1	0.10	-1.00	51.4	0	0	0	CHEMBL1200796	1.88	2	1	0	14	-0.08	0.61	5
Cycloserine (D)	0	0			Antibiotic	C1[C@H](C(=O)NO1)N	102.1	-2.42	8.21	64.4	0	0	1	CHEMBL771	-1.62	3	2	0	7	1.9	0.39	0
Cycloserine (L)	0	0			Antibiotic	C1[C@H](C(=O)NO1)N	102.1	-2.42	8.21	64.4	0	0	1	CHEMBL448864	-1.62	3	2	0	7	1.9	0.39	0
Cytarabine	0	0			Cytostatic	C1=CN(C(=O)N=C1N)[C@H]2[C@H]([C@@H]([C@H](O2)CO)O)O	243.2	-2.80	3.59	128.6	0	0	0	CHEMBL2447907	-2.56	8	4	1	17	1.65	0.45	2

Substance	MATE1 substrate*	MATE2 substrate*	OCT1 substrate*	OCT2 substrate*	Therapeutic group	SMILES	Molecular weight	logD at pH 7.4	pKa (basic)	TPSA	Net Charge	Quaternary N	Zwitterionic	ChEMBL ID	AlogP	Hydrogen bound	Hydrogen bound	Aromatic rings	Heavy atoms	Natprod likeness score	Rqed (weighted)	Rotable bounds
Cytisine	0	0	0	0	Herbal	C1[C@H]2CNC[C@@H]1C3=CC=CC(=O)N3C2	190.3	-2.64	9.82	32.3	1	0	0	CHEMBL497939	0.56	3	1	1	14	-0.21	0.65	0
Dapsone	0	0			Antiinfective	C1=CC(=CC=C1N)S(=O)(=O)C2=CC=C(C=C2)N	248.3	1.27	2.39	94.6	0	0	0	CHEMBL1043	1.68	4	2	2	17	-0.74	0.79	2
Daunorubicin	0	0	0	0	Cytostatic	C[C@H]1[C@H]([C@H](C[C@@H](O1)O[C@H]2C[C@@](CC3=C2C(=C4C(=C3O)C(=O)C5=C(C4=O)C(=CC=C5)OC)O)(C(=O)C)O)N)O	527.5	0.37	10.93	185.8	1	0	0	CHEMBL1200475	1.03	11	5	2	38	1.75	0.31	4
Debrisoquine	1	1	1	1	Antihypertensive	C1CN(CC2=CC=CC=C21)C(=N)N	175.2	-1.35	13.02	53.1	1	0	0	CHEMBL169901	0.94	1	2	1	13	-0.75	0.46	0
Decanoylcarnitine	1	0			Carnitine ester	CCCCCCCCCC(=O)O[C@H](CC(=O)[O-])C[N+](C)(C)C	315.5	0.14	14.00	66.4	0	1	1	CHEMBL2216788	1.89	4	0	0	22	0.57	0.3	13
Decitabine	0	0			Cytostatic	C1[C@@H]([C@H](O[C@H]1N2C=NC(=NC2=O)N)CO)O	228.2	-2.16	0.75	120.7	0	0	0	CHEMBL1201129	-2.14	8	3	1	16	1.11	0.53	2
Dehydrocorydaline	1	1	1	1	Herbal	CC1=C2C=CC(=C(C2=C[N+]3=C1C4=CC(=C(C=C4CC3)OC)OC)OC)OC	366.4	-0.71	14.00	40.8	1	1	0	CHEMBL1917176	3.69	4	0	3	27	1.13	0.66	4
Delavirdine	0	0			Virostatic	CC(C)NC1=C(N=CC=C1)N2CCN(CC2)C(=O)C3=CC4=C(N3)C=CC(=C4)NS(=O)(=O)C	456.6	0.88	6.80	118.8	0	0	0	CHEMBL593	2.72	6	3	3	32	-1.75	0.53	6
Denatonium	1	1	1	1	Others	CC[N+](CC)(CC1=CC=CC=C1)CC(=O)NC2=C(C=CC=C2C)C	325.5	0.41	14.00	29.1	1	1	0	CHEMBL1738972	4.3	1	1	2	24	-0.62	0.76	7
Denopamine (R)	1	0		0	Sympathomimetic	COC1=C(C=C(C=C1)CCNC[C@@H](C2=CC=C(C=C2)O)O)OC	317.4	0.17	9.84	71.0	1	0	0	CHEMBL493682	2.28	5	3	2	23	0.08	0.65	8
Deoxyepinephrine	1	1	1		Sympathomimetic	CNCCC1=CC(=C(C=C1)O)O	167.2	-1.27	9.35	52.5	1	0	0	CHEMBL31088	0.86	3	3	1	12	0.99	0.59	3
Desipramine	0	0	0	0	Antidepressant	CNCCCN1C2=CC=CC=C2CCC3=CC=CC=C31	266.4	1.37	10.02	15.3	1	0	0	CHEMBL72	3.53	2	1	2	20	-0.44	0.85	4
Desvenlafaxine	0	0	1	0	Antidepressant	CN(C)CC(C1=CC=C(C=C1)O)C2(CCCCC2)O	263.4	0.98	8.90	43.7	1	0	0	CHEMBL1201728	2.73	3	2	1	19	0.44	0.88	4

Substance	MATE1 substrate*	MATE2 substrate*	OCT1 substrate*	OCT2 substrate*	Therapeutic group	SMILES	Molecular weight	logD at pH 7.4	pKa (basic)	TPSA	Net Charge	Quaternary N	Zwitterionic	ChEMBL ID	AlogP	Hydrogen bound	Hydrogen bound	Aromatic rings	Heavy atoms	Natprod likeness score	Rqed (weighted)	Rotable bounds
Dextromethorphan	0	0	0	0	Opioid	CN1CC[C@@]23CCCC[C@@H]2[C@@H]1CC4=C3C=C(C=C4)OC	271.4	1.08	9.85	12.5	1	0	0	CHEMBL52440	3.38	2	0	1	20	1.15	0.78	1
Diclofenac	0	0		0	Analgesic	C1=CC=C(C(=C1)CC(=O)O)NC2=C(C=CC=C2Cl)Cl	296.2	1.10	-2.10	49.3	-1	0	0	CHEMBL139	4.36	2	2	2	19	-0.87	0.88	4
Diethyltryptamine	0	0	0	0	Psychedelic	CCN(CC)CCC1=CNC2=CC=CC=C21	216.3	0.37	10.11	19.0	1	0	0	CHEMBL142936	3.05	1	1	2	16	-0.77	0.81	5
Dihydroergotamine	0	0			Others	C[C@@]1(C(=O)N2[C@H](C(=O)N3CCC[C@H]3[C@@]2(O1)O)CC4=CC=CC=C4)NC(=O)[C@@H]5C[C@H]6[C@@H](CC7=CNC8=CC=CC6=C78)N(C5)C	583.7	2.22	7.96	118.2	1	0	0	CHEMBL1200517	2.08	6	3	3	43	0.83	0.43	4
Diltiazem	0	0	0	0	Antihypertensive	CC(=O)O[C@@H]1[C@@H](SC2=CC=CC=C2N(C1=O)CCN(C)C)C3=CC=C(C=C3)OC	414.5	1.89	8.18	84.4	1	0	0	CHEMBL1200805	3.37	6	0	2	29	-0.13	0.68	6
Dimenhydrinate	0	0			Antihistaminic	CN1C2=C(C(=O)N(C1=O)C)NC(=N2)Cl.CN(C)CCOC(C1=CC=CC=C1)C2=CC=CC=C2	470.0	2.17	8.87	12.5	1	0	0	CHEMBL1200406	3.35	2	0	2	19	-0.52	0.78	6
Dimethindene	0	0			Antihistaminic	CC(C1=CC=CC=N1)C2=C(CC3=CC=CC=C32)CCN(C)C	292.4	1.46	9.70	16.1	1	0	0	CHEMBL1870768	4.15	2	0	2	22	-0.3	0.82	5
Dimethylphenylpiperazinium	1	1	1	1	Experimental chemical	C[N+]1(CCN(CC1)C2=CC=CC=C2)C	191.3	-2.23	14.00	3.2	1	1	0	CHEMBL134752	1.58	1	0	1	14	-0.58	0.61	1
Dimethyltryptamine	0	0	0	1	Psychedelic	CN(C)CCC1=CNC2=CC=CC=C21	188.3	0.15	9.57	19.0	1	0	0	CHEMBL12420	2.27	1	1	2	14	-0.34	0.78	3
Diphenhydramine	0	0	0	0	Antihistaminic	CN(C)CCOC(C1=CC=CC=C1)C2=CC=CC=C2	255.4	2.17	8.87	12.5	1	0	0	CHEMBL1201089	3.35	2	0	2	19	-0.52	0.78	6
Dipyridamole	0	0		0	Anticoagulant	C1CCN(CC1)C2=NC(=NC3=C2N=C(N=C3N4CCCCC4)N(CCO)CCO)N(CCO)CCO	504.6	1.71	6.79	145.4	0	0	0	CHEMBL932	-0.02	12	4	2	36	-0.52	0.31	12
Disopyramide	0	0	0	1	Antiarrhythmic	CC(C)N(CCC(C1=CC=CC=C1)(C2=CC=CC=N2)C(=O)N)C(C)C	339.5	0.57	10.42	59.2	1	0	0	CHEMBL1201020	3.36	3	1	2	25	-0.74	0.8	8

Substance	MATE1 substrate*	MATE2 substrate*	OCT1 substrate*	OCT2 substrate*	Therapeutic group	SMILES	Molecular weight	logD at pH 7.4	pKa (basic)	TPSA	Net Charge	Quarternary N	Zwitterionic	ChEMBL ID	AlogP	Hydrogen bound	Hydrogen bound	Aromatic rings	Heavy atoms	Natprod likeness score	Rqed (weighted)	Rotable bounds
Dobutamine	1	1	1	1	Sympathomimetic	CC(CCC1=CC=C(C=C1)O)NCCC2=CC(=C(C=C2)O)O	301.4	1.13	9.35	72.7	1	0	0	CHEMBL1200418	2.96	4	4	2	22	0.39	0.59	7
Dofetilide	1	1	1	0	Antiarrhythmic	CN(CCC1=CC=C(C=C1)NS(=O)(=O)C)CCOC2=CC=C(C=C2)NS(=O)(=O)C	441.6	-1.01	9.26	121.6	1	0	0	CHEMBL473	1.98	6	2	2	29	-1.07	0.55	11
Domperidone	0	0	0	0	Antiemetic	C1CN(CCC1N2C3=C(C=C(C=C3)Cl)NC2=O)CCCN4C5=CC=CC=C5NC4=O	425.9	2.18	8.03	67.9	1	0	0	CHEMBL219916	3.35	5	2	4	30	-1.54	0.51	5
Donepezil	0	0			Others	COC1=C(C=C2C(=C1)CC(C2=O)CC3CCN(CC3)CC4=CC=CC=C4)OC	379.5	2.48	9.12	38.8	1	0	0	CHEMBL502	4.36	4	0	2	28	-0.06	0.75	6
Dopamine	1	0	0		Biogenic amine	C1=CC(=C(C=C1CCN)O)O	153.2	-1.41	9.31	66.5	1	0	0	CHEMBL1557	0.6	3	3	1	11	1.15	0.54	2
Doxazosin	0	0	0	0	Alpha1 blocker	COC1=C(C=C2C(=C1)C(=NC(=N2)N3CCN(CC3)C(=O)C4COC5=CC=CC=C5O4)N)OC	451.5	1.43	8.04	112.3	1	0	0	CHEMBL1200561	1.72	9	1	3	33	-0.79	0.63	4
Doxepin	0	0	0	0	Antidepressant	CN(C)CC/C=C/1\C2=CC=CC=C2COC3=CC=CC=C31	279.4	2.18	9.06	12.5	1	0	0	CHEMBL860	3.96	2	0	2	21	-0.05	0.84	3
Doxepin (E)	0	0			Antidepressant	CN(C)CC/C=C/1\C2=CC=CC=C2COC3=CC=CC=C31	279.4	2.18	9.06	12.5	1	0	0	CHEMBL860	3.96	2	0	2	21	-0.05	0.84	3
Doxepin (Z)	0	0			Antidepressant	CN(C)CC/C=C/1\C2=CC=CC=C2COC3=CC=CC=C31	279.4	2.18	9.06	12.5	1	0	0	CHEMBL860	3.96	2	0	2	21	-0.05	0.84	3
Doxycycline	0	0		0	Antibiotic	C[C@@H]1[C@H]2[C@@H]([C@H]3[C@@H](C(=O)C(=C([C@]3(C(=O)C2=C(C4=C1C=CC=C4O)O)O)O)C(=O)N)N(C)C)O	444.4	-5.88	5.98	181.6	-1	0	0	CHEMBL1200699	-0.35	9	6	1	32	1.81	0.33	2
Doxylamine	0	0	0	0	Antihistaminic	CC(C1=CC=CC=C1)(C2=CC=CC=N2)OCCN(C)C	270.4	1.48	8.87	25.4	1	0	0	CHEMBL1004	2.92	3	0	2	20	-0.73	0.81	6

Substance	MATE1 substrate*	MATE2 substrate*	OCT1 substrate*	OCT2 substrate*	Therapeutic group	SMILES	Molecular weight	logD at pH 7.4	pKa (basic)	TPSA	Net Charge	Quarternary N	Zwitterionic	ChEMBL ID	AlogP	Hydrogen bound	Hydrogen bound	Aromatic rings	Heavy atoms	Natprod likeness score	Rqed (weighted)	Rotable bounds
Doxylamine (R)	0	0	0		Antihistaminic	CC(C1=CC=CC=C1)(C2=CC=CC=N2)OCCN(C)C	270.4	1.48	8.87	25.4	1	0	0	CHEMBL1004	2.92	3	0	2	20	-0.73	0.81	6
Doxylamine (S)	0	0	0		Antihistaminic	CC(C1=CC=CC=C1)(C2=CC=CC=N2)OCCN(C)C	270.4	1.48	8.87	25.4	1	0	0	CHEMBL1004	2.92	3	0	2	20	-0.73	0.81	6
Dropropizine	0	0			Others	C1CN(CCN1CC(CO)O)C2=CC=CC=C2	236.2	-0.31	8.27	46.9	1	0	0	CHEMBL151445	0.16	4	2	1	17	-1.09	0.78	4
Dropropizine (D)	0	0			Others	C1CN(CCN1CC(CO)O)C2=CC=CC=C2	236.2	-0.31	8.27	46.9	1	0	0	CHEMBL151445	0.16	4	2	1	17	-1.09	0.78	4
Dropropizine (L)	0	0	0	1	Others	C1CN(CCN1CC(CO)O)C2=CC=CC=C2	236.2	-0.31	8.27	46.9	1	0	0	CHEMBL151445	0.16	4	2	1	17	-1.09	0.78	4
Duloxetine	0	0	0	0	Antidepressant	CNCC[C@@H](C1=CC=CS1)OC2=CC=CC3=CC=CC=C32	297.4	2.31	9.30	49.5	1	0	0	CHEMBL1200328	4.63	3	1	3	21	-0.54	0.72	6
Duloxetine (R)	0	0			Antidepressant	CNCC[C@@H](C1=CC=CS1)OC2=CC=CC3=CC=CC=C32	297.4	2.31	9.30	49.5	1	0	0	CHEMBL336920	4.63	3	1	3	21	-0.54	0.72	6
Duloxetine (S)	0	0			Antidepressant	CNCC[C@@H](C1=CC=CS1)OC2=CC=CC3=CC=CC=C32	297.4	2.31	9.30	49.5	1	0	0	CHEMBL1200328	4.63	3	1	3	21	-0.54	0.72	6
Edrophonium	1	1	1	1	Others	CC[N+](C)(C)C1=CC(=CC=C1)O	166.2	-0.96	14.00	20.2	1	1	0	CHEMBL1104	1.98	1	1	1	12	0.56	0.67	2
Efaroxan	0	0	1	1	Alpha2 antagonist	CCC1(CC2=CC=CC=C2O1)C3=NCCN3	216.3	0.50	9.06	33.6	1	0	0	CHEMBL539313	1.77	3	1	1	16	0.39	0.82	2
Eletriptan	0	0	0	0	Triptan	CN1CCC[C@@H]1CC2=CNC3=C2C=C(C=C3)CCS(=O)(=O)C4=CC=CC=C4	382.5	1.58	9.62	61.6	1	0	0	CHEMBL1510	3.82	3	1	3	27	-0.72	0.71	6
Emtricitabine	1	1	1	1	Virostatic	C1[C@H](O[C@H](S1)CO)N2C=C(C(=NC2=O)N)F	247.2	-0.90	1.14	113.5	0	0	0	CHEMBL885	-0.46	7	2	1	16	0.7	0.73	2
Endoxifen	0	0	0	0	Oncology	CC/C(=C(\C1=CC=C(C=C1)O)/C2=CC=C(C=C2)OCCNC)/C3=CC=CC=C3	373.5	4.15	8.78	41.5	1	0	0	CHEMBL1093458	5.36	3	2	3	28	0.12	0.41	8
Entecavir	0	0	0	0	Virostatic	C=C1[C@H](C[C@@H]([C@H]1CO)O)N2C=NC3=C2N=C(NC3=O)N	277.3	-1.96	3.08	125.8	0	0	0	CHEMBL713	-0.83	7	4	2	20	1.03	0.53	2

Substance	MATE1 substrate*	MATE2 substrate*	OCT1 substrate*	OCT2 substrate*	Therapeutic group	SMILES	Molecular weight	logD at pH 7.4	pKa (basic)	TPSA	Net Charge	Quarternary N	Zwitterionic	ChEMBL ID	AlogP	Hydrogen bound	Hydrogen bound	Aromatic rings	Heavy atoms	Natprod likeness score	Rqed (weighted)	Rotable bounds
Ephedrine	0	0	0	1	Sympathomimetic	C[C@@H]([C@@H](C1=CC=CC=C1)O)NC	165.2	-0.78	9.52	32.3	1	0	0	CHEMBL1523964	1.33	2	2	1	12	0.43	0.71	3
Epiberberine	1	1	1	1	Herbal	COC1=C(C=C2C(=C1)CC[N+]3=C2C=C4C=CC5=C(C4=C3)OCO5)OC	336.4	-1.28	14.00	40.8	1	1	0	CHEMBL1197637	3.1	4	0	3	25	1.37	0.67	2
Epinephrine (-)	1	1			Sympathomimetic	CNC[C@@H](C1=CC(=C(C=C1)O)O)O	183.2	-1.63	8.91	72.7	1	0	0	CHEMBL1256958	0.35	4	4	1	13	1.12	0.51	3
Epinephrine (rac)	1	1	0	0	Sympathomimetic	CNC[C@@H](C1=CC(=C(C=C1)O)O)O	183.2	-1.63	8.91	72.7	1	0	0	CHEMBL1256958	0.35	4	4	1	13	1.12	0.51	3
Ergometrine	0	0			Herbal	C[C@@H](CO)NC(=O)[C@H]1CN([C@@H]2CC3=CNC4=CC=CC(=C34)C2=C1)C	325.4	0.93	6.98	68.4	0	0	0	CHEMBL119443	1.53	3	3	2	24	0.86	0.8	3
Ergothioneine (L)	0	0	0	0	Endobiotic	C[N+](C)(C)[C@@H](CC1=CNC(=S)N1)C(=O)[O-]	229.3	-3.26	14.00	96.3	0	1	1	CHEMBL4303270	0.77	2	3	1	15	0.65	0.53	4
Esmolol	0	0	0	0	Beta blocker	CC(C)NCC(COC1=CC=C(C=C1)CCC(=O)OC)O	295.4	-0.03	9.27	67.8	1	0	0	CHEMBL1201115	1.53	5	2	1	21	-0.24	0.68	9
Estrone-3-sulfate	1	1	0	0	Steroid hormone	C[C@]12CC[C@H]3[C@H]([C@@H]1CCC2=O)CCC4=C3C=CC(=C4)OS(=O)(=O)O	350.4	1.46	-7.50	89.1	-1	0	0	CHEMBL494753	3.29	4	1	1	24	1.7	0.83	2
Ethambutol	1	1	1	1	Antibiotic	CC[C@@H](CO)NCCN[C@@H](CC)CO	204.3	-2.25	9.55	64.5	1	0	0	CHEMBL44884	-0.29	4	4	0	14	0.06	0.39	9
Etilefrine	1	1	1	1	Sympathomimetic	CCNCC(C1=CC(=CC=C1)O)O	181.2	-1.07	9.73	52.5	1	0	0	CHEMBL3187408	1.04	3	3	1	13	0.26	0.65	4
Etomidate	0	0	0	0	Others	CCOC(=O)C1=CN=CN1[C@H](C)C2=CC=CC=C2	244.3	2.50	4.82	44.1	0	0	0	CHEMBL681	2.67	4	0	2	18	-0.96	0.78	4
Famotidine	1	1	1	1	Antihistaminic	C1=C(N=C(S1)N=C(N)N)CSCC/C(=N/S(=O)(=O)N)/N	337.4	-2.84	8.44	237.8	1	0	0	CHEMBL902	-0.77	6	4	1	20	-1.83	0.29	7
Fampridine (4-aminopyridine)	0	0	0	1	Others	C1=CN=CC=C1N	94.1	-0.97	8.95	38.9	1	0	0	CHEMBL284348	0.66	2	1	1	7	-0.66	0.51	0
Fasudil	0	0	0	1	Others	C1CNCCN(C1)S(=O)(=O)C2=CC=CC3=C2C=CN=C3	291.4	-0.40	8.04	70.7	1	0	0	CHEMBL38380	1.22	4	1	2	20	-1.64	0.9	2
Fedratinib	0	0			Oncology	CC1=CN=C(N=C1NC2=CC(=CC=C2)S(=O)(=O)	524.7	3.27	8.97	116.9	1	0	0	CHEMBL4297216	4.82	8	3	3	37	-1.71	0.35	10

Substance	MATE1 substrate*	MATE2 substrate*	OCT1 substrate*	OCT2 substrate*	Therapeutic group	SMILES	Molecular weight	logD at pH 7.4	pKa (basic)	TPSA	Net Charge	Quarternary N	Zwitterionic	ChEMBL ID	AlogP	Hydrogen bound	Hydrogen bound	Aromatic rings	Heavy atoms	Natprod likeness score	Rqed (weighted)	Rotable bounds
						NC(C)(C)C)NC3=CC=C(C=C3)OCCN4CCCC4																
Felbamate	0	0			Antiepileptic	C1=CC=C(C=C1)C(COC(=O)N)COC(=O)N	238.2	0.68	-2.00	104.6	0	0	0	CHEMBL1094	0.96	4	2	1	17	0.07	0.8	5
Fenfluramine (R)	0	1	1	0	Others	CCNC(C)CC1=CC(=CC=C1)C(F)(F)F	231.3	0.79	10.22	12.0	1	0	0	CHEMBL2106217	3.25	1	1	1	16	-0.98	0.84	4
Fenfluramine (S)	0	0	0	1	Others	CCNC(C)CC1=CC(=CC=C1)C(F)(F)F	231.3	0.79	10.22	12.0	1	0	0	CHEMBL2106217	3.25	1	1	1	16	-0.98	0.84	4
Fenoldopam	1	1	0	0	Dopamine agonist	C1CNCC(C2=CC(=C(C(=C21)Cl)O)O)C3=CC=C(C=C3)O	305.8	0.92	10.30	72.7	1	0	0	CHEMBL1256646	2.73	4	4	2	21	0.95	0.61	1
Fenoterol	1	1	1	1	Sympathomimetic	CC(CC1=CC=C(C=C1)O)NCC(C2=CC(=CC(=C2)O)O)O	303.4	0.34	10.09	93.0	1	0	0	CHEMBL32800	2.06	5	5	2	22	0.43	0.56	6
Fenpiverinium	1	0	1	1	(Anti)cholinergic	C[N+]1(CCCCC1)CCC(C2=CC=CC=C2)(C3=CC=CC=C3)C(=O)N	337.5	-0.56	14.00	43.1	1	1	0	CHEMBL1884833	3.48	1	1	2	25	-0.02	0.81	6
Fentanyl	0	0	0	0	Opioid	CCC(=O)N(C1CCN(CC1)CCC2=CC=CC=C2)C3=CC=CC=C3	336.5	2.71	8.47	23.6	1	0	0	CHEMBL1201159	4.14	2	0	2	25	-1.26	0.79	6
Fesoterodine	0	0		0	(Anti)cholinergic	CC(C)C(=O)OC1=C(C=C(C=C1)CO)[C@H](CCN(C(C)C)C(C)C)C2=CC=CC=C2	411.6	2.65	10.64	49.8	1	0	0	CHEMBL1201764	5.38	4	1	2	30	-0.01	0.42	10
Fexofenadine	0	0	0	0	Antihistaminic	CC(C)(C1=CC=C(C=C1)C(CCCN2CCC(CC2)C(C3=CC=CC=C3)(C4=CC=CC=C4)O)O)C(=O)O	501.7	2.94	9.21	81.0	0	0	1	CHEMBL1200618	5.51	4	3	3	37	-0.33	0.34	10
Filgotinib	0	0	1		JAK inhibitor	C1CC1C(=O)NC2=NN3C(=N2)C=CC=C3C4=CC=C(C=C4)CN5CCS(=O)(=O)CC5	425.5	2.03	2.62	105.1	0	0	0	CHEMBL3301607	1.98	7	1	3	30	-2	0.67	5
Fingolimod	0	0			Others	CCCCCCCCC1=CC=C(C=C1)CCC(CO)(CO)N	307.5	2.12	9.38	66.5	1	0	0	CHEMBL314854	3.2	3	3	1	22	0.66	0.52	12
Flecainide	0	0	1	0	Antiarrhythmic	C1CCNC(C1)CNC(=O)C2=C(C=CC(=C2)OCC(F)(F)F)OCC(F)(F)F	414.3	1.01	9.62	59.6	1	0	0	CHEMBL1200822	3.44	4	2	1	28	-0.9	0.67	7
Flunarizine	0	0			Calcium antagonist	C1CN(CCN1C/C=C/C2=CC=CC=C2)C(C3=CC=	404.5	5.82	7.49	6.5	1	0	0	CHEMBL552659	5.39	2	0	3	30	-0.98	0.54	6

Substance	MATE1 substrate*	MATE2 substrate*	OCT1 substrate*	OCT2 substrate*	Therapeutic group	SMILES	Molecular weight	logD at pH 7.4	pKa (basic)	TPSA	Net Charge	Quaternary N	Zwitterionic	ChEMBL ID	AlogP	Hydrogen bound	Hydrogen bound	Aromatic rings	Heavy atoms	Natprod likeness score	Rqed (weighted)	Rotable bounds
						C(C=C3)F)C4=CC=C(C=C4)F																
Fluoxetine	0	0	0	0	Antidepressant	CNCCC(C1=CC=CC=C1)OC2=CC=C(C=C2)C(F)(F)F	309.3	2.19	9.40	21.3	1	0	0	CHEMBL1201082	4.44	2	1	2	22	-0.35	0.85	6
Fluoxetine (R)	0	0			Antidepressant	CNCCC(C1=CC=CC=C1)OC2=CC=C(C=C2)C(F)(F)F	309.3	2.19	9.40	21.3	1	0	0	CHEMBL1256757	4.44	2	1	2	22	-0.35	0.85	6
Fluoxetine (S)	0	0			Antidepressant	CNCCC(C1=CC=CC=C1)OC2=CC=C(C=C2)C(F)(F)F	309.3	2.19	9.40	21.3	1	0	0	CHEMBL1257031	4.44	2	1	2	22	-0.35	0.85	6
Fluphenazine	0	0	0	0	Antipsychotic	C1CN(CCN1CCCN2C3=CC=CC=C3SC4=C2C=C(C=C4)C(F)(F)F)CCO	437.5	2.73	8.61	55.3	1	0	0	CHEMBL726	4.31	5	1	2	30	-1.28	0.73	6
Fluvoxamine	0	0	0	0	Antidepressant	COCCCC/C(=N\OCCN)/C1=CC=C(C=C1)C(F)(F)F	318.3	1.42	8.76	56.8	1	0	0	CHEMBL814	3.2	4	1	1	22	-0.69	0.43	9
Folic acid	0	0			Vitamin	C1=CC(=CC=C1C(=O)N[C@@H](CCC(=O)O)C(=O)O)NCC2=CN=C3C(=N2)C(=O)NC(=N3)N	441.4	-6.61	2.82	209.0	-2	0	0	CHEMBL1622	-0.04	9	6	3	32	-0.4	0.26	9
Formoterol	1	0	1	0	Sympathomimetic	CC(CC1=CC=C(C=C1)OC)NCC(C2=CC(=C(C=C2)O)NC=O)O	344.4	0.04	9.81	90.8	1	0	0	CHEMBL1256786	2.22	5	4	2	25	-0.07	0.41	9
Formoterol (R)	1	0			Sympathomimetic	C[C@H](CC1=CC=C(C=C1)OC)NC[C@@H](C2=CC(=C(C=C2)O)NC=O)O	344.4	0.04	9.81	90.8	1	0	0	CHEMBL1363	2.22	5	4	2	25	-0.07	0.41	9
Frovatriptan	1	1	1	1	Triptan	CN[C@@H]1CCC2=C(C1)C3=C(N2)C=CC(=C3)C(=O)N	243.3	-1.74	10.42	70.9	1	0	0	CHEMBL1279	1.34	2	3	2	18	-0.45	0.74	2
Furosemide	0	0			Diuretic	C1=COC(=C1)CNC2=CC(=C(C=C2C(=O)O)S(=O)(=O)N)Cl	330.7	-1.63	-1.50	131.0	-1	0	0	CHEMBL35	1.89	5	3	2	21	-1.77	0.77	5
Gabapentin	0	0	0	0	Antiepileptic	C1CCC(CC1)(CC(=O)O)CN	171.2	-1.27	9.91	63.3	0	0	1	CHEMBL940	1.37	2	2	0	12	0.69	0.67	3
Gabexate	1	1	1	1	Anticoagulant	CCOC(=O)C1=CC=C(C=C1)OC(=O)CCCCCN=C(N)N	321.4	-0.37	12.21	117.0	1	0	0	CHEMBL87563	1.81	5	3	1	23	-0.14	0.21	9

Substance	MATE1 substrate*	MATE2 substrate*	OCT1 substrate*	OCT2 substrate*	Therapeutic group	SMILES	Molecular weight	logD at pH 7.4	pKa (basic)	TPSA	Net Charge	Quaternary N	Zwitterionic	ChEMBL ID	AlogP	Hydrogen bound	Hydrogen bound	Aromatic rings	Heavy atoms	Natprod likeness score	Rqed (weighted)	Rotable bounds
Galantamine	0	0	0	0	Cholinesterase inhibitor	CN1CC[C@@]23C=C[C@@H](C[C@@H]2OC4=C(C=CC(=C34)C1)OC)O	287.4	0.76	7.58	41.9	1	0	0	CHEMBL659	1.85	4	1	1	21	2.11	0.8	1
Gamma-aminobutyric acid	0	0		1	Endobiotic	C(CC(=O)O)CN	103.1	-2.89	10.22	63.3	0	0	1	CHEMBL96	-0.19	2	2	0	7	0.98	0.52	3
Ganciclovir	0	0	0	0	Virostatic	C1=NC2=C(N1COC(CO)CO)N=C(NC2=O)N	255.2	-2.18	2.88	135.0	0	0	0	CHEMBL1200850	-1.97	8	4	2	18	0.38	0.49	5
Gefitinib	0	0		0	Oncology	COC1=C(C=C2C(=C1)N=CN=C2NC3=CC(=C(C=C3)F)Cl)OCCCN4CCOCC4	446.9	3.52	7.25	68.7	0	0	0	CHEMBL939	4.28	7	1	3	31	-1.75	0.52	8
Gemcitabine	0	0			Cytostatic	C1=CN(C(=O)N=C1N)[C@H]2C([C@@H]([C@H](O2)CO)O)(F)F	263.2	-1.47	3.05	108.4	0	0	0	CHEMBL888	-1.29	7	3	1	18	1.3	0.61	2
Gentian violet	0	0	0	0	Experimental chemical	CN(C)C1=CC=C(C=C1)C(=C2C=CC(=[N+](C)C)C=C2)C3=CC=C(C=C3)N(C)C	372.5	1.39	4.83	9.5	1	1	0	CHEMBL64894	4.46	2	0	2	28	-0.31	0.73	4
Glutamic acid (D)	0	0	0	0	Amino acid	C(CC(=O)O)[C@@H](C(=O)O)N	147.1	-6.17	9.54	100.6	-1	0	1	CHEMBL575060	-0.74	3	3	0	10	1.32	0.49	4
Glycopyrrolate	1	0	1	1	(Anti)cholinergic	C[N+]1(CCC(C1)OC(=O)C(C2CCCC2)(C3=CC=CC=C3)O)C	318.4	-1.41	14.00	46.5	1	1	0	CHEMBL1201335	2.46	3	1	1	23	0.36	0.69	4
Granisetron	0	0	0	0	5HT3 Antagonist	CN1[C@@H]2CCC[C@H]1CC(C2)NC(=O)C3=NN(C4=CC=CC=C43)C	312.4	0.56	8.70	50.2	1	0	0	CHEMBL1237080	2.32	4	1	2	23	-1.06	0.93	2
Guanethidine	1	1	1	1	Antihypertensive	C1CCCN(CCC1)CCN=C(N)N	198.3	-4.70	12.35	67.6	2	0	0	CHEMBL1345	0.74	2	3	0	14	-0.88	0.46	3
Guanfacine	0	0	1	1	Antihypertensive	C1=CC(=C(C(=C1)Cl)CC(=O)N=C(N)N)Cl	246.1	0.43	8.30	81.5	1	0	0	CHEMBL862	1.55	2	3	1	15	-1.12	0.55	2
Haloperidol	0	0	0	0	Antipsychotic	C1CN(CCC1(C2=CC=C(C=C2)Cl)O)CCCC(=O)C3=CC=C(C=C3)F	375.9	2.85	8.14	40.5	1	0	0	CHEMBL54	4.43	3	1	2	26	-0.94	0.76	6
Halostachine	0	0	0	1	Herbal	CNC[C@@H](C1=CC=CC=C1)O	151.2	-1.11	9.43	32.3	1	0	0	CHEMBL1241267	0.94	2	2	1	11	0.39	0.67	3
Harmaline	0	0	1	1	Herbal	CC1=NCCC2=C1NC3=C2C=CC(=C3)OC	214.3	1.41	7.32	37.4	1	0	0	CHEMBL340807	2.54	2	1	2	16	0.72	0.78	1
Heptylamine	0	0	0	1	Experimental chemical	CCCCCCCN	115.2	-0.57	10.21	26.0	1	0	0	CHEMBL339253	5.16	1	1	0	8	0.29	0.54	5

Substance	MATE1 substrate*	MATE2 substrate*	OCT1 substrate*	OCT2 substrate*	Therapeutic group	SMILES	Molecular weight	logD at pH 7.4	pKa (basic)	TPSA	Net Charge	Quarternary N	Zwitterionic	ChEMBL ID	AlogP	Hydrogen bound	Hydrogen bound	Aromatic rings	Heavy atoms	Natprod likeness score	Rqed (weighted)	Rotable bounds
Hexanoylcarnitine	0	0	0	0	Carnitine ester	CCCCCC(=O)OC(CC(=O)[O-])C[N+](C)(C)C	259.3	-1.64	14.00	66.4	0	1	1	#NV	-2.5	3	0	0	18	#NV	#NV	10
Hexylamine	0	0	1	0	Experimental chemical	CCCCCCN	101.2	-1.02	10.21	26.0	1	0	0	CHEMBL3098428	3.34	1	1	0	7	-0.48	0.83	4
Higenamine	1	1	1	1	Sympathomimetic	C1CNC(C2=CC(=C(C=C21)O)O)CC3=CC=C(C=C3)O	271.3	1.41	8.57	72.7	1	0	0	CHEMBL19344	2.23	4	4	2	20	1.38	0.63	2
Histamine	1	0	0	1	Biogenic amine	C1=C(NC=N1)CCN	111.1	-3.44	9.74	54.7	1	0	0	CHEMBL1533310	-0.09	2	2	1	8	0	0.56	2
Homoarginine	0	0			Amino acid	C(CCN=C(N)N)C[C@@H](C(=O)O)N	188.2	-4.76	12.39	127.7	1	0	1	CHEMBL589752	-0.95	3	5	0	13	0.98	0.21	6
Hordenine	0	0	1	1	Sympathomimetic	CN(C)CCC1=CC=C(C=C1)O	165.2	0.11	9.08	23.5	1	0	0	CHEMBL505789	1.5	2	1	1	12	0.09	0.73	3
Hydrochlorothiazide	1	1			Diuretic	C1NC2=CC(=C(C=C2S(=O)(=O)N1)S(=O)(=O)N)Cl	297.7	-0.58	-2.70	135.1	0	0	0	CHEMBL435	-0.35	5	3	1	17	-1.23	0.65	1
Hydrocodone	0	0	0	0	Opioid	CN1CC[C@]23[C@@H]4[C@H]1CC5=C2C(=C(C=C5)OC)O[C@H]3C(=O)CC4	299.4	0.48	8.87	38.8	1	0	0	CHEMBL3989677	1.93	4	0	1	22	1.74	0.79	1
Hydromorphone	0	0	0	1	Opioid	CN1CC[C@]23[C@@H]4[C@H]1CC5=C2C(=C(C=C5)O)O[C@H]3C(=O)CC4	285.3	0.33	9.42	49.8	1	0	0	CHEMBL1237055	1.63	4	1	1	21	2.07	0.79	0
Hydroxybenzylamine	0	0	1	1	Herbal	C1=CC(=CC=C1CN)O	123.2	-1.05	9.06	46.3	1	0	0	CHEMBL3114402	0.85	2	2	1	9	0.41	0.58	1
Hydroxybupropion	0	0	0	0	Antidepressant	CC(C(=O)C1=CC(=CC=C1)Cl)NC(C)(C)CO	255.7	1.78	7.65	49.3	1	0	0	CHEMBL3544616	2.27	3	2	1	17	-0.65	0.79	5
Hydroxychloroquine (R)	1	0	0	0	Antiinfective	CCN(CCCC(C)NC1=C2C=CC(=CC2=NC=C1)Cl)CCO	335.9	0.31	9.76	48.4	1	0	0	CHEMBL4799846	3.78	4	2	2	23	-1.23	0.73	9
Hydroxychloroquine (S)	1	0	0	0	Antiinfective	CCN(CCCC(C)NC1=C2C=CC(=CC2=NC=C1)Cl)CCO	335.9	0.31	9.76	48.4	1	0	0	CHEMBL1535	3.78	4	2	2	23	-1.23	0.73	9
Hypaphorine	0	0	0	0	Herbal	C[N+](C)(C)[C@@H](CC1=CNC2=CC=CC=C21)C(=O)[O-]	246.3	-1.40	14.00	55.9	0	1	1	CHEMBL503867	0.54	2	1	2	18	0.31	0.8	4
Ibogaine	0	0	0		Psychedelic	CC[C@H]1C[C@@H]2C[C@@H]3[C@H]1N(C2)	310.4	1.96	8.97	28.3	1	0	0	CHEMBL1215855	3.94	2	1	2	23	1	0.91	2

Substance	MATE1 substrate*	MATE2 substrate*	OCT1 substrate*	OCT2 substrate*	Therapeutic group	SMILES	Molecular weight	logD at pH 7.4	pKa (basic)	TPSA	Net Charge	Quaternary N	Zwitterionic	ChEMBL ID	AlogP	Hydrogen bound	Hydrogen bound	Aromatic rings	Heavy atoms	Natprod likeness score	Rqed (weighted)	Rotable bounds
						CCC4=C3NC5=C4C=C(C=C5)OC																
Ibutilide	0	0			Antiarrhythmic	CCCCCCCN(CC)CCCC(C1=CC=C(C=C1)NS(=O)(=O)C)O	384.6	0.64	10.85	78.0	1	0	0	CHEMBL2355456	4.16	4	2	1	26	-0.75	0.47	14
Ifosfamide	0	0	0	0	Oncology	C1CN(P(=O)(OC1)NCCCl)CCCl	261.1	0.10	-2.00	51.4	0	0	0	CHEMBL1024	1.88	2	1	0	14	-0.4	0.61	5
Imatinib	0	0	0	0	Oncology	CC1=C(C=C(C=C1)NC(=O)C2=CC=C(C=C2)CN3CCN(CC3)C)NC4=NC=CC(=N4)C5=CN=CC=C5	493.6	3.52	8.20	86.3	1	0	0	CHEMBL941	4.59	7	2	4	37	-1.8	0.39	7
Imeglimin	1	1	1	1	Antidiabetic	C[C@@H]1N=C(NC(=N1)N(C)C)N	155.2	-4.29	11.60	66.0	1	0	0	CHEMBL4297514	-0.83	5	2	0	11	-0.11	0.48	0
Imipramine	0	0	0	0	Antidepressant	CN(C)CCCN1C2=CC=CC=C2CCC3=CC=CC=C31	280.4	2.48	9.20	6.5	1	0	0	CHEMBL1692	3.88	2	0	2	21	-0.77	0.84	4
Indacaterol	0	0	0	1	Sympathomimetic	CCC1=C(C=C2CC(CC2=C1)NC[C@@H](C3=C4C=CC(=O)NC4=C(C=C3)O)O)CC	392.5	2.32	9.71	81.6	1	0	0	CHEMBL1095777	3.15	4	4	3	29	0.35	0.52	6
Ipratropium	1	1	1	1	(Anti)cholinergic	CC(C)[N+]1([C@@H]2CC[C@H]1CC(C2)OC(=O)C(CO)C3=CC=CC=C3)C	332.5	-1.82	14.00	46.5	1	1	0	CHEMBL1621597	2.85	3	1	1	24	0.73	0.67	5
Irinotecan	0	0	0	0	Cytostatic	CCC1=C2CN3C(=CC4=C(C3=O)COC(=O)[C@@]4(CC)O)C2=NC5=C1C=C(C=C5)OC(=O)N6CCC(CC6)N7CCCCC7	586.7	-0.32	9.18	112.5	0	0	1	CHEMBL3989514	4.09	9	1	3	43	0.26	0.36	4
Isobutyrylcarnitine	0	0			Carnitine ester	CC(C)C(=O)O[C@H](CC(=O)[O-])C[N+](C)(C)C	231.3	-2.43	14.00	66.4	0	1	1	#NV	-3.13	3	0	0	16	#NV	#NV	7
Isoetharine	1	0	1	1	Sympathomimetic	CCC(C(C1=CC(=C(C=C1)O)O)O)NC(C)C	239.2	-0.22	9.01	72.7	1	0	0	CHEMBL1201213	1.91	4	4	1	17	0.58	0.59	5
Isoniazid	0	0	0	0	Antibiotic	C1=CN=CC=C1C(=O)NN	137.1	-0.69	3.35	68.0	0	0	0	CHEMBL64	-0.31	3	2	1	10	-1.62	0.32	1
Isoprenaline	1	1	1	0	Sympathomimetic	CC(C)NCC(C1=CC(=C(C=C1)O)O)O	211.3	-0.99	8.96	72.7	1	0	0	CHEMBL1160723	1.13	4	4	1	15	0.5	0.56	4
Isovalerylcarnitine	0	0			Carnitine ester	CC(C)CC(=O)OC(CC(=O)[O-])C[N+](C)(C)C	245.3	-2.24	14.00	66.4	0	1	1	#NV	-2.97	3	0	0	17	#NV	#NV	8

Substance	MATE1 substrate*	MATE2 substrate*	OCT1 substrate*	OCT2 substrate*	Therapeutic group	SMILES	Molecular weight	logD at pH 7.4	pKa (basic)	TPSA	Net Charge	Quaternary N	Zwitterionic	ChEMBL ID	AlogP	Hydrogen bound	Hydrogen bound	Aromatic rings	Heavy atoms	Natprod likeness score	Rqed (weighted)	Rotable bounds
Ivabradine	0	0			Others	CN(CCCN1CCC2=CC(=C(C=C2CC1=O)OC)OC)C[C@H]3CC4=CC(=C(C=C34)OC)OC	468.6	0.83	9.30	60.5	1	0	0	CHEMBL471737	3.31	6	0	2	34	-0.24	0.53	10
Ketamine	0	0	0	0	Psychedelic	CNC1(CCCCC1=O)C2=CC=CC=C2Cl	237.7	3.10	7.29	29.1	1	0	0	CHEMBL742	2.9	2	1	1	16	0.02	0.86	2
Kynurenine	0	0	0	0	Endobiotic	C1=CC=C(C(=C1)C(=O)CC(C(=O)O)N)N	208.2	-1.92	8.96	106.4	0	0	1	CHEMBL1377927	0.25	4	3	1	15	0.67	0.49	4
Labetalol	0	0	0	0	Beta blocker	CC(CCC1=CC=CC=C1)NCC(C2=CC(=C(C=C2)O)C(=O)N)O	328.4	1.26	9.80	95.6	1	0	0	CHEMBL429	2.14	4	4	2	24	-0.21	0.6	8
Lafutidine	0	0	0	0	Antihistaminic	C1CCN(CC1)CC2=CC(=NC=C2)OC/C=C\CNC(=O)CS(=O)CC3=CC=CO3	431.6	0.51	7.94	103.9	1	0	0	CHEMBL1742461	2.66	6	1	2	30	-1.11	0.43	11
Lamivudine	1	1	1	1	Virostatic	C1[C@H](O[C@H](S1)CO)N2C=CC(=NC2=O)N	229.3	-1.10	3.70	113.5	0	0	0	CHEMBL18314	-0.59	7	2	1	15	1.46	0.7	2
Lamotrigine	0	0	0	1	Antiepileptic	C1=CC(=C(C(=C1)Cl)Cl)C2=C(N=C(N=N2)N)N	256.1	1.93	4.85	90.7	0	0	0	CHEMBL741	2.01	5	2	2	16	-1.11	0.81	1
Landiolol	1	1	0	0	Beta blocker	CC1(OC[C@H](O1)COC(=O)CCC2=CC=C(C=C2)OC[C@H](CNCCNC(=O)N3CCOCC3)O)C	509.6	-0.68	8.39	127.8	1	0	0	CHEMBL1742466	0.69	9	3	1	36	-0.54	0.26	13
Lenalidomide	0	0	0	0	Oncology	C1CC(=O)NC(=O)C1N2CC3=C(C2=O)C=CC=C3N	259.3	-0.71	2.32	92.5	0	0	0	CHEMBL848	0.03	4	2	1	19	0.28	0.55	1
Leucine	0	0	0	0	Amino acid	CC(C)C[C@@H](C(=O)O)N	131.2	-1.59	9.52	63.3	0	0	1	CHEMBL291962	0.44	2	2	0	9	0.98	0.58	3
Levetiracetam	0	0	0	0	Antiepileptic	CC[C@@H](C(=O)N)N1CCCC1=O	170.2	-0.59	0.86	63.4	0	0	0	CHEMBL1286	-0.13	2	1	0	12	-0.76	0.64	3
Levodopa	0	0	0	0	Others	C1=CC(=C(C=C1C[C@@H](C(=O)O)N)O)O	197.2	-1.80	9.06	103.8	0	0	1	CHEMBL1009	0.05	4	4	1	14	1.19	0.51	3
Levomepromazine	0	0			Antipsychotic	C[C@@H](CN1C2=CC=CC=C2SC3=C1C=C(C=C3)OC)CN(C)C	328.5	2.24	9.42	15.7	1	0	0	CHEMBL2104973	4.5	4	0	2	23	-1	0.8	5
Lidocaine	0	0	0	0	Local anesthetic	CCN(CC)CC(=O)NC1=C(C=CC=C1C)C	234.3	2.33	7.75	32.3	1	0	0	CHEMBL1200409	2.58	2	1	1	17	-1.69	0.85	5
Lysine	0	0	0	0	Amino acid	C(CCN)C[C@@H](C(=O)O)N	146.2	-4.98	10.29	89.3	1	0	1	CHEMBL8085	-0.47	3	3	0	10	1.45	0.46	5

Substance	MATE1 substrate*	MATE2 substrate*	OCT1 substrate*	OCT2 substrate*	Therapeutic group	SMILES	Molecular weight	logD at pH 7.4	pKa (basic)	TPSA	Net Charge	Quarternary N	Zwitterionic	ChEMBL ID	AlogP	Hydrogen bound	Hydrogen bound	Aromatic rings	Heavy atoms	Natprod likeness score	Rqed (weighted)	Rotable bounds
Maprotiline	0	0	0	0	Antidepressant	CNCCCC12CCC(C3=CC=CC=C31)C4=CC=CC=C24	277.4	1.25	11.14	12.0	1	0	0	CHEMBL21731	4.21	1	1	2	21	0.83	0.83	4
Maraviroc	0	0			Virostatic	CC1=NN=C(N1C2C[C@H]3CC[C@@H](C2)N3CC[C@@H](C4=CC=CC=C4)NC(=O)C5CCC(CC5)(F)F)C(C)C	513.7	1.99	9.03	63.1	1	0	0	CHEMBL1201187	5.95	5	1	2	37	-0.85	0.47	8
Matrine	0	0	1	0	Others	C1C[C@@H]2[C@H]3CCCN4[C@H]3[C@@H](CCC4)CN2C(=O)C1	248.4	-1.25	9.75	23.6	1	0	0	CHEMBL204860	1.87	2	0	0	18	1.17	0.65	0
Mecamylamine	0	0	1	1	(Anti)cholinergic	CC1(C2CCC(C2)C1(C)NC)C	167.3	-0.81	11.48	12.0	1	0	0	CHEMBL1237082	2.42	1	1	0	12	1.56	0.63	1
Medetomidine	0	0	0	0	Alpha2 agonist	CC1=C(C(=CC=C1)C(C)C2=CN=CN2)C	200.3	2.96	7.19	28.7	0	0	0	CHEMBL537161	3.18	1	1	2	15	-0.44	0.79	2
Memantine	0	0	0	0	Others	CC12CC3CC(C1)(CC(C3)(C2)N)C	179.3	-0.67	10.45	26.0	1	0	0	CHEMBL807	2.69	1	1	0	13	0.53	0.61	0
Mepenzolate	1	0	1	1	(Anti)cholinergic	C[N+]1(CCCC(C1)OC(=O)C(C2=CC=CC=C2)(C3=CC=CC=C3)O)C	340.4	-0.97	14.00	46.5	1	1	0	CHEMBL524004	2.7	3	1	2	25	0.3	0.69	4
Mepivacaine	0	0	1	0	Local anesthetic	CC1=C(C(=CC=C1)C)NC(=O)C2CCCCN2C	246.4	2.97	7.23	32.3	1	0	0	CHEMBL1087	2.73	2	1	1	18	-1.02	0.87	2
Meptazinol	0	0	0	0	Opioid	CCC1(CCCCN(C1)C)C2=CC(=CC=C2)O	233.4	1.55	9.16	23.5	1	0	0	CHEMBL1477274	3.16	2	1	1	17	0.45	0.85	2
Meropenem	0	0	0	0	Antibiotic	C[C@@H]1[C@@H]2[C@H](C(=O)N2C(=C1S[C@H]3C[C@H](NC3)C(=O)N(C)C)C(=O)O)[C@@H](C)O	383.5	-4.39	8.39	135.5	0	0	1	CHEMBL127	-0.31	6	3	0	26	0.78	0.56	5
Mescaline	1	0	1	0	Psychedelic	COC1=CC(=CC(=C1OC)OC)CCN	211.3	-1.32	9.70	53.7	1	0	0	CHEMBL26687	1.21	4	1	1	15	0.52	0.8	5
Metamfetamine	0	0	0		Psychostimulant	C[C@@H](CC1=CC=CC=C1)NC	149.2	-0.44	10.21	12.0	1	0	0	CHEMBL1201201	1.84	1	1	1	11	-0.21	0.69	3
Metanephrine	1	0	1	1	Endobiotic	CNCC(C1=CC(=C(C=C1)O)OC)O	197.2	-1.52	9.25	61.7	1	0	0	CHEMBL775	0.65	4	3	1	14	0.93	0.66	4
Metformin	0	0	0	1	Others	CN(C)C(=N)N=C(N)N	129.2	-5.75	12.72	91.5	2	0	0	CHEMBL1431	-1.03	2	4	0	9	0.07	0.25	0
Methacholine	1	1	1	1	(Anti)cholinergic	CC(C[N+](C)(C)C)OC(=O)C	160.2	-3.80	14.00	26.3	1	1	0	CHEMBL978	0.64	2	0	0	11	1.13	0.45	3

Substance	MATE1 substrate*	MATE2 substrate*	OCT1 substrate*	OCT2 substrate*	Therapeutic group	SMILES	Molecular weight	logD at pH 7.4	pKa (basic)	TPSA	Net Charge	Quaternary N	Zwitterionic	ChEMBL ID	AlogP	Hydrogen bound	Hydrogen bound	Aromatic rings	Heavy atoms	Natprod likeness score	Rqed (weighted)	Rotable bounds
Methoxytryptophan (5-)	0	0			Endobiotic	COC1=CC2=C(C=C1)NC=C2C[C@@H](C(=O)O)N	234.3	-1.25	9.42	88.3	0	0	1	CHEMBL3707060	-2.21	4	3	2	17	0.7	0.18	4
Methyldopa	0	0	0	0	Antihypertensive	C[C@](CC1=CC(=C(C=C1)O)O)(C(=O)O)N	211.2	-1.37	9.85	103.8	0	0	1	CHEMBL459	0.44	4	4	1	15	1.09	0.54	3
Methylecgonine	0	0	0		Herbal	CN1C2CCC1C(C(C2)O)C(=O)OC	199.3	-1.96	9.15	49.8	1	0	0	CHEMBL305331	0	4	1	0	14	1.38	0.6	1
Methylene blue	1	0	1	1	Experimental chemical	CN(C)C1=CC2=C(C=C1)N=C3C=CC(=[N+](C)C)C=C3S2	284.4	-0.62	3.14	43.9	1	0	0	CHEMBL550495	2.5	3	0	1	20	-1.55	0.51	1
Methylenedioxyethylamphetamine	0	0	0		Psychostimulant	CCNC(C)CC1=CC2=C(C=C1)OCO2	207.3	-0.46	10.22	30.5	1	0	0	CHEMBL126279	1.96	3	1	1	15	0.04	0.82	4
Methylergometrine	0	0			Psychedelic	CC[C@@H](CO)NC(=O)[C@H]1CN([C@@H]2CC3=CNC4=CC=CC(=C34)C2=C1)C	339.4	1.45	6.98	68.4	0	0	0	CHEMBL1201356	1.92	3	3	2	25	0.91	0.8	4
Methylnaltrexone	1	1	1	1	Opioid antagonist	C[N+]1(CC[C@]23[C@@H]4C(=O)CC[C@]2([C@@H]1CC5=C3C(=C(C=C5)O)O4)O)CC6CC6	356.4	-1.80	14.00	66.8	1	1	0	CHEMBL1186579	1.67	4	2	1	26	1.89	0.79	2
Methylnicotinamide	1	1	1	1	Endobiotic	CNC(=O)C1=CN=CC=C1	136.2	-0.17	3.62	42.0	0	0	0	CHEMBL11978	0.44	2	1	1	10	-1.56	0.61	1
Methylphenidate	0	0	0	1	Psychostimulant	COC(=O)C(C1CCCCN1)C2=CC=CC=C2	233.3	0.57	9.09	38.3	1	0	0	CHEMBL796	2.09	3	1	1	17	0.18	0.81	3
Methylscopolamine	1	1	1	1	(Anti)cholinergic	C[N+]1([C@@H]2CC(C[C@H]1[C@H]3[C@@H]2O3)OC(=O)[C@H](CO)C4=CC=CC=C4)C	318.4	-3.27	14.00	59.1	1	1	0	CHEMBL3140030	1.06	4	1	1	23	1.14	0.51	4
Methysergide	0	0	0	0	Others	CC[C@@H](CO)NC(=O)[C@H]1CN([C@@H]2CC3=CN(C4=CC=CC(=C34)C2=C1)C)C	353.5	1.70	6.88	57.5	0	0	0	CHEMBL1065	1.94	4	2	2	26	0.74	0.88	4
Metipranolol	0	0	0	0	Beta blocker	CC1=CC(=C(C(=C1OC(=O)C)C)C)OCC(CNC(C)C)O	309.4	0.89	9.27	67.8	1	0	0	CHEMBL1291	2.27	5	2	1	22	0.01	0.6	7
Metoclopramide	0	0	0	0	Others	CCN(CC)CCNC(=O)C1=CC(=C(C=C1OC)N)Cl	299.8	-0.25	9.04	67.6	1	0	0	CHEMBL1200940	2	4	2	1	20	-1.54	0.76	7
Metoprolol	0	0	0	0	Beta blocker	CC(C)NCC(COC1=CC=C(C=C1)CCOC)O	267.4	-0.10	9.27	50.7	1	0	0	CHEMBL2356097	1.61	4	2	1	19	-0.38	0.71	9

Substance	MATE1 substrate*	MATE2 substrate*	OCT1 substrate*	OCT2 substrate*	Therapeutic group	SMILES	Molecular weight	logD at pH 7.4	pKa (basic)	TPSA	Net Charge	Quaternary N	Zwitterionic	ChEMBL ID	AlogP	Hydrogen bound	Hydrogen bound	Aromatic rings	Heavy atoms	Natprod likeness score	Rqed (weighted)	Rotable bounds
Mexiletine	0	0	0	0	Antiarrhythmic	CC1=C(C(=CC=C1)C)OCC(C)N	179.3	0.53	9.37	32.3	1	0	0	CHEMBL558	2.03	2	1	1	13	-0.42	0.77	3
Mexiletine (R)	0	0			Antiarrhythmic	CC1=C(C(=CC=C1)C)OCC(C)N	179.3	0.53	9.37	32.3	1	0	0	CHEMBL558	2.03	2	1	1	13	-0.42	0.77	3
Mexiletine (S)	0	0			Antiarrhythmic	CC1=C(C(=CC=C1)C)OCC(C)N	179.3	0.53	9.37	32.3	1	0	0	CHEMBL558	2.03	2	1	1	13	-0.42	0.77	3
Miconazole	0	0	0	0	Antiinfective	C1=CC(=C(C=C1Cl)Cl)COC(CN2C=CN=C2)C3=C(C=C(C=C3)Cl)Cl	416.1	5.84	7.05	27.1	0	0	0	CHEMBL1559	6.45	3	0	3	25	-0.92	0.46	6
Midodrine	1	0	0	0	Sympathomimetic	COC1=CC(=C(C=C1)OC)C(CNC(=O)CN)O	254.3	-1.76	8.14	93.8	1	0	0	CHEMBL1201212	-0.19	5	3	1	18	-0.59	0.65	6
Milnacipran	1	0	1	1	Antidepressant	CCN(CC)C(=O)[C@@]1(C[C@@H]1CN)C2=CC=CC=C2	246.4	-0.91	9.83	46.3	1	0	0	CHEMBL4297064	1.77	2	1	1	18	-0.1	0.86	5
Minocycline	0	0	0	0	Antibiotic	CN(C)[C@H]1[C@@H]2C[C@@H]3CC4=C(C=CC(=C4C(=C3C(=O)[C@@]2(C(=C(C1=O)C(=O)N)O)O)O)O)N(C)C	457.5	-4.76	7.64	164.6	0	0	1	CHEMBL1200881	0.19	9	5	1	33	1.3	0.4	3
Minoxidil	0	0	0	1	Antihypertensive	C1CCN(CC1)C2=NC(=N)N(C(=C2)N)O	209.3	-1.63	8.77	88.9	1	0	0	CHEMBL802	-0.13	5	2	1	15	-1.22	0.5	1
m-Iodobenzylguanidin	1	1	1	1	Others	C1=CC(=CC(=C1)I)CN=C(N)N	275.1	-0.77	12.18	64.4	1	0	0	CHEMBL4584538	1.27	1	3	1	12	-1.2	0.43	2
Mirabegron	1	1	0	0	Sympathomimetic	C1=CC=C(C=C1)[C@H](CNCCC2=CC=C(C=C2)NC(=O)CC3=CSC(=N3)N)O	396.5	0.70	9.62	133.1	1	0	0	CHEMBL2095212	2.77	6	4	3	28	-1.43	0.42	9
Molsidomine	0	0			Others	CCO/C(=N/C1=C[N+](=NO1)N2CCOCC2)/[O-]	242.2	-0.55	14.00	87.0	1	1	0	CHEMBL1329455	0.09	6	0	1	17	-0.64	0.7	3
Moroxydine	1	0	1	1	Antiinfective	C1COCCN1C(=N)N=C(N)N	171.2	-5.12	12.43	100.7	2	0	0	CHEMBL1490434	-1.26	3	4	0	12	-1.02	0.27	0
Morphine	0	0	1	0	Opioid	CN1CC[C@]23[C@@H]4[C@H]1CC5=C2C(=C(C=C5)O)O[C@H]3[C@H](C=C4)O	285.3	-0.31	8.65	52.9	1	0	0	CHEMBL2103744	1.2	4	2	1	21	2.59	0.7	0
Moxifloxacin	0	0			Antibiotic	COC1=C2C(=CC(=C1N3C[C@@H]4CCCN[C@@H]4C3)F)C(=O)C(=CN2C5CC5)C(=O)O	401.4	-0.49	9.43	82.1	0	0	1	CHEMBL1200735	2.37	6	2	2	29	0.08	0.82	4

Substance	MATE1 substrate*	MATE2 substrate*	OCT1 substrate*	OCT2 substrate*	Therapeutic group	SMILES	Molecular weight	logD at pH 7.4	pKa (basic)	TPSA	Net Charge	Quaternary N	Zwitterionic	ChEMBL ID	AlogP	Hydrogen bound	Hydrogen bound	Aromatic rings	Heavy atoms	Natprod likeness score	Rqed (weighted)	Rotable bounds
Moxonidine	1	0			Antihypertensive	CC1=NC(=C(C(=N1)Cl)NC2=NCCN2)OC	241.7	-0.53	9.68	71.4	1	0	0	CHEMBL1256287	0.82	6	2	1	16	-0.89	0.75	2
m-Tyramine	1	0	1	1	Biogenic amine	C1=CC(=CC(=C1)O)CCN	137.2	-1.12	10.04	46.3	1	0	0	CHEMBL145584	0.89	2	2	1	10	0.77	0.64	2
N-Acetyl-L-cysteine	0	0			Others	CC(=O)N[C@@H](CS)C(=O)O	163.2	-4.10	0.17	105.2	-1	0	0	CHEMBL600	-0.49	3	3	0	10	0.22	0.49	3
Nadolol	1	1	1	1	Beta blocker	CC(C)(C)NCC(COC1=CC=CC2=C1C[C@@H]([C@@H](C2)O)O)O	309.4	-1.08	9.36	82.0	1	0	0	CHEMBL521606	0.63	5	4	1	22	0.26	0.64	5
Nafamostat	1	0	0	0	Anticoagulant	C1=CC(=CC=C1C(=O)OC2=CC3=C(C=C2)C=C(C=C3)C(=N)N)N=C(N)N	347.4	-1.70	11.32	140.6	2	0	0	CHEMBL3989553	2.65	4	5	3	26	-0.39	0.21	4
Nalbuphine	0	0			Opioid	C1CC(C1)CN2CC[C@]34[C@@H]5[C@H](CC[C@]3([C@H]2CC6=C4C(=C(C=C6)O)O5)O)O	357.5	-0.07	9.60	73.2	1	0	0	CHEMBL895	1.71	5	3	1	26	1.87	0.75	2
Nalmefene	0	0		0	Opioid antagonist	C=C1CC[C@]2([C@H]3CC4=C5[C@]2([C@H]1OC5=C(C=C4)O)CCN3CC6CC6)O	339.4	0.73	9.55	52.9	1	0	0	CHEMBL982	2.51	4	2	1	25	1.84	0.81	2
Naloxone	0	0			Opioid antagonist	C=CCN1CC[C@]23[C@@H]4C(=O)CC[C@]2([C@@H]1CC5=C3C(=C(C=C5)O)O4)O	327.4	1.13	7.68	70.0	1	0	0	CHEMBL3249799	1.3	5	2	1	24	1.94	0.8	2
Naltrexone	0	0	0	0	Opioid antagonist	C1CC1CN2CC[C@]34[C@@H]5C(=O)CC[C@]3([C@H]2CC6=C4C(=C(C=C6)O)O5)O	341.4	0.27	8.54	70.0	1	0	0	CHEMBL1201149	1.53	5	2	1	25	1.72	0.85	2
Naratriptan	1	1		0	Triptan	CNS(=O)(=O)CCC1=CC2=C(C=C1)NC=C2C3CCN(CC3)C	335.5	-0.32	9.16	73.6	1	0	0	CHEMBL1200601	2.07	3	2	2	23	-0.93	0.88	5
N-Desmethylcitalopram	0	0	0		Antidepressant	CNCCCC1(C2=C(CO1)C=C(C=C2)C#N)C3=CC=C(C=C3)F	310.4	0.26	11.14	45.1	1	0	0	CHEMBL2074679	3.47	3	1	2	23	-0.19	0.86	5
N-Desmethylranitidine	1	1	1	1	Antihistaminic	CNCC1=CC=C(O1)CSCCNC(=C[N+](=O)[O-])NC	300.4	-0.43	9.57	117.7	1	0	1	CHEMBL2178720	-2.34	5	3	1	20	1.31	0.38	10
N-Desmethyltramadol	0	0	0		Opioid	CNCC1CCCCC1(C2=CC(=CC=C2)OC)O	249.4	-0.35	9.89	41.5	1	0	0	CHEMBL4466163	2.29	3	2	1	18	0.91	0.86	4

Substance	MATE1 substrate*	MATE2 substrate*	OCT1 substrate*	OCT2 substrate*	Therapeutic group	SMILES	Molecular weight	logD at pH 7.4	pKa (basic)	TPSA	Net Charge	Quarternary N	Zwitterionic	ChEMBL ID	AlogP	Hydrogen bound	Hydrogen bound	Aromatic rings	Heavy atoms	Natprod likeness score	Rqed (weighted)	Rotable bounds
N-Desmethylvenlafaxine	0	0	0		Antidepressant	CNCC(C1=CC=C(C=C1)OC)C2(CCCCC2)O	263.4	0.03	9.78	41.5	1	0	0	CHEMBL1628258	2.69	3	2	1	19	0.64	0.86	5
Nebivolol	0	0	0	0	Beta blocker	C1CC2=C(C=CC(=C2)F)OC1C(CNCC(C3CCC4=C(O3)C=CC(=C4)F)O)O	405.4	2.73	7.70	71.0	1	0	0	CHEMBL434394	2.36	5	3	2	29	0.07	0.69	6
Nefazodone	0	0		0	Antidepressant	CCC1=NN(C(=O)N1CCOC2=CC=CC=C2)CCCN3CCN(CC3)C4=CC(=CC=C4)Cl	470.0	3.48	8.55	51.6	1	0	0	CHEMBL1200492	3.55	7	0	3	33	-1.96	0.45	10
Neostigmine	1	1	1	1	(Anti)cholinergic	CN(C)C(=O)OC1=CC=CC(=C1)[N+](C)(C)C	223.3	-2.25	14.00	29.5	1	1	0	CHEMBL278020	1.94	2	0	1	16	-0.26	0.72	2
N-Ethyl-lidocaine	1	1	1	1	Local anesthetic	CC[N+](CC)(CC)CC(=O)NC1=C(C=CC=C1C)C	263.4	-0.96	14.00	29.1	1	1	0	CHEMBL1180496	3.12	1	1	1	19	-0.78	0.79	6
Niacin	0	0			Vitamin	C1=CC(=CN=C1)C(=O)O	123.1	-3.09	3.47	50.2	-1	0	0	CHEMBL573	0.78	2	1	1	9	-0.71	0.6	1
Nicotinamide	0	0	0	0	Vitamin	C1=CC(=CN=C1)C(=O)N	122.1	-0.39	3.63	56.0	0	0	0	CHEMBL1140	0.18	2	1	1	9	-1.3	0.58	1
Nimodipine	0	0			Calcium antagonist	CC1=C(C(C(=C(N1)C)C(=O)OC(C)C)C2=CC(=CC=C2)[N+](=O)[O-])C(=O)OCCOC	418.4	2.54	4.31	117.0	0	0	1	CHEMBL1514440	2.97	8	1	1	30	-1.02	0.3	8
Nizatidine	1	1	1	0	Antihistaminic	CN/C(=C\[N+](=O)[O-])/NCCSCC1=CSC(=N1)CN(C)C	331.5	0.72	6.54	136.9	0	0	1	CHEMBL3183075	1.32	8	2	1	21	-2.04	0.38	10
N-Methyl-2-phenylethylamine	0	0	0		Endobiotic	CNCCC1=CC=CC=C1	135.2	-0.79	10.13	12.0	1	0	0	CHEMBL45763	1.45	1	1	1	10	0.03	0.66	3
N-Methyl-p-Tyramine	1	0	1		Herbal	CNCCC1=CC=C(C=C1)O	151.2	-1.04	10.39	32.3	1	0	0	CHEMBL5169925	1.15	2	2	1	11	0.62	0.68	3
N-Methylserotonin	1	1	1	1	Herbal	CNCCC1=CNC2=C1C=C(C=C2)O	190.2	-1.03	10.44	48.1	1	0	0	CHEMBL1256710	1.76	2	3	2	14	0.47	0.68	2
N-Methyltryptamine	0	0	1		Psychedelic	CNCCC1=CNC2=CC=CC=C21	174.2	-0.83	10.31	27.8	1	0	0	CHEMBL348588	1.93	1	2	2	13	0	0.73	3
NN-Dimethylarginine	0	0			Endobiotic	CN(C)C(=NCCC[C@@H](C(=O)O)N)N	202.3	-4.35	12.89	104.9	1	0	1	CHEMBL457530	-0.74	3	4	0	14	0.8	0.27	5
Norepinephrine (L)	1	0			Sympathomimetic	C1=CC(=C(C=C1[C@H](CN)O)O)O	169.2	-1.76	8.81	86.7	1	0	0	CHEMBL5087452	0.09	4	4	1	12	1.19	0.47	2
Norepinephrine (S)	1	0			Sympathomimetic	C1=CC(=C(C=C1[C@H](CN)O)O)O	169.2	-1.76	8.81	86.7	1	0	0	CHEMBL432	0.09	4	4	1	12	1.19	0.47	2

Substance	MATE1 substrate*	MATE2 substrate*	OCT1 substrate*	OCT2 substrate*	Therapeutic group	SMILES	Molecular weight	logD at pH 7.4	pKa (basic)	TPSA	Net Charge	Quarternary N	Zwitterionic	ChEMBL ID	AlogP	Hydrogen bound	Hydrogen bound	Aromatic rings	Heavy atoms	Natprod likeness score	Rqed (weighted)	Rotable bounds
Norepinephrine rac	1	0	0		Sympathomimetic	C1=CC(=C(C=C1[C@H](CN)O)O)O	169.2	-1.76	8.81	86.7	1	0	0	CHEMBL432	0.09	4	4	1	12	1.19	0.47	2
Norfenfluramine (+)	0	0	0	1	Sympathomimetic	CC(CC1=CC(=CC=C1)C(F)(F)F)N	203.2	0.26	9.94	26.0	1	0	0	CHEMBL253811	2.6	1	1	1	14	-0.9	0.79	2
Normetanephrine	0	0	1	0	Endobiotic	COC1=C(C=CC(=C1)C(CN)O)O	183.2	-1.64	9.00	75.7	1	0	0	CHEMBL2447959	0.39	4	3	1	13	0.96	0.63	3
Noroxycodone	0	0	1	0	Opioid	COC1=C2C3=C(C[C@@H]4[C@]5([C@]3(CCN4)[C@@H](O2)C(=O)CC5)O)C=C1	301.3	-1.20	9.26	67.8	1	0	0	CHEMBL3527426	0.71	5	2	1	22	1.95	0.8	1
Norphenylephrine	1	0	1	1	Sympathomimetic	C1=CC(=CC(=C1)O)C(CN)O	153.2	-1.45	8.86	66.5	1	0	0	CHEMBL446333	0.38	3	3	1	11	0.78	0.58	2
Nortilidine	0	0	0	0	Opioid	CCOC(=O)[C@]1(CCC=C[C@@H]1NC)C2=CC=CC=C2	259.3	1.49	8.87	38.3	1	0	0	CHEMBL1614654	2.43	3	1	1	19	0.86	0.67	4
Nortriptyline	0	0	0	0	Antidepressant	CNCCC=C1C2=CC=CC=C2CCC3=CC=CC=C31	263.4	1.58	10.47	12.0	1	0	0	CHEMBL1201156	3.83	1	1	2	20	0.35	0.83	3
Ochratoxin A	1	0			Herbal	C[C@@H]1CC2=C(C=C(C(=C2C(=O)O1)O)C(=O)N[C@@H](CC3=CC=CC=C3)C(=O)O)Cl	403.8	1.01	12.00	112.9	-1	0	0	CHEMBL589366	2.57	5	3	2	28	0.27	0.66	5
Octopamine	1	0	1	1	Sympathomimetic	C1=CC(=CC=C1C(CN)O)O	153.2	-1.49	8.93	66.5	1	0	0	CHEMBL1367565	0.38	3	3	1	11	0.89	0.58	2
Ofloxacin	0	0	0		Antibiotic	CC1COC2=C3N1C=C(C(=O)C3=CC(=C2N4CCN(CC4)C)F)C(=O)O	361.4	-0.82	8.31	73.3	0	0	1	CHEMBL4	1.54	6	1	2	26	-0.21	0.87	2
Ofloxacin (L)	0	0	0	0	Antibiotic	C[C@H]1COC2=C3N1C=C(C(=O)C3=CC(=C2N4CCN(CC4)C)F)C(=O)O	361.4	-0.82	8.31	73.3	0	0	1	CHEMBL4	1.54	6	1	2	26	-0.21	0.87	2
Olanzapine	0	0			Antipsychotic	CC1=CC2=C(S1)NC3=CC=CC=C3N=C2N4CCN(CC4)C	312.4	2.56	8.16	59.1	1	0	0	CHEMBL3989694	3.44	5	1	2	22	-0.99	0.81	0
Olodaterol	1	1	1	0	Sympathomimetic	CC(C)(CC1=CC=C(C=C1)OC)NC[C@@H](C2=C3C(=CC(=C2)O)NC(=O)CO3)O	386.4	-0.18	9.84	100.1	1	0	0	CHEMBL2105743	2.38	6	4	2	28	0.15	0.58	7
Ondansetron	0	0	1	0	5HT3 Antagonist	CC1=NC=CN1CC2CCC3=C(C2=O)C4=CC=CC=C4N3C	293.4	1.83	7.91	39.8	1	0	0	CHEMBL3186492	3.13	4	0	3	22	-0.54	0.73	2

Substance	MATE1 substrate*	MATE2 substrate*	OCT1 substrate*	OCT2 substrate*	Therapeutic group	SMILES	Molecular weight	logD at pH 7.4	pKa (basic)	TPSA	Net Charge	Quarternary N	Zwitterionic	ChEMBL ID	AlogP	Hydrogen bound	Hydrogen bound	Aromatic rings	Heavy atoms	Natprod likeness score	Rqed (weighted)	Rotable bounds
Orciprenaline	1	1	1	1	Sympathomimetic	CC(C)NCC(C1=CC(=CC(=C1)O)O)O	211.3	-0.94	9.70	72.7	1	0	0	CHEMBL1628446	1.13	4	4	1	15	0.55	0.6	4
Ornithine	0	0	0		Endobiotic	C(C[C@@H](C(=O)O)N)CN	132.2	-5.42	10.29	89.3	1	1	1	CHEMBL446143	-0.86	3	3	0	9	1.59	0.46	4
Orphenadrine	0	0	0	0	Antihistaminic	CC1=CC=CC=C1C(C2=CC=CC=C2)OCCN(C)C	269.4	2.69	8.87	12.5	1	0	0	CHEMBL900	3.66	2	0	2	20	-0.72	0.79	6
Oseltamivir	0	0	0	0	Virostatic	CCC(CC)O[C@@H]1C=C(C[C@@H]([C@H]1NC(=O)C)N)C(=O)OCC	312.4	-0.67	9.26	90.7	1	0	0	CHEMBL1229	1.29	5	2	0	22	1.02	0.69	7
Oxiconazole	0	0	0	0	Antiinfective	C1=CC(=C(C=C1Cl)Cl)CO/N=C(\CN2C=CN=C2)/C3=C(C=C(C=C3)Cl)Cl	429.1	5.66	7.31	39.4	0	0	0	CHEMBL1262	6.12	4	0	3	26	-1.47	0.35	6
Oxprenolol	0	0	0	0	Beta blocker	CC(C)NCC(COC1=CC=CC=C1OCC=C)O	265.4	0.31	9.27	50.7	1	0	0	CHEMBL1200745	1.99	4	2	1	19	-0.33	0.67	9
Oxycodone	0	0	0	0	Opioid	CN1CC[C@]23[C@@H]4C(=O)CC[C@]2([C@H]1CC5=C3C(=C(C=C5)OC)O4)O	315.4	0.28	8.07	59.0	1	0	0	CHEMBL656	1.05	5	1	1	23	1.91	0.84	1
Oxymetazoline	1	1	1	1	Sympathomimetic	CC1=CC(=C(C(=C1CC2=NCCN2)C)O)C(C)(C)C	260.4	1.20	10.15	44.6	1	0	0	CHEMBL1200791	2.85	3	2	1	19	0.26	0.86	2
Oxyphenonium	1	1	1	1	(Anti)cholinergic	CC[N+](C)(CC)CCOC(=O)C(C1CCCCC1)(C2=CC=CC=C2)O	348.5	-0.20	14.00	46.5	1	1	0	CHEMBL1201286	3.48	3	1	1	25	0.09	0.58	8
Oxytetracycline	0	0	0		Antibiotic	C[C@@]1([C@H]2[C@@H]([C@H]3[C@@H](C(=O)C(=C([C@]3(C(=O)C2=C(C4=C1C=CC=C4O)O)O)O)O)C(=O)N)N(C)C)O)O	460.4	-7.30	5.98	201.9	-1	0	0	CHEMBL1517	-1.24	10	7	1	33	1.83	0.26	2
Paliperidone	0	0	0	0	Antipsychotic	CC1=C(C(=O)N2CCCC(C2=N1)O)CCN3CCC(CC3)C4=NOC5=C4C=CC(=C5)F	426.5	0.38	8.76	82.2	1	0	0	CHEMBL1621	3.08	7	1	3	31	-0.89	0.69	4
Palmatine	1	1	1	1	Herbal	COC1=C(C2=C[N+]3=C(C=C2C=C1)C4=CC(=C(C=C4CC3)OC)OC)OC	352.4	-1.22	14.00	40.8	1	1	0	CHEMBL206106	3.38	4	0	3	26	1.14	0.68	4
Palonosetron	0	0	0	0	5HT3 Antagonist	C1C[C@@H]2CN(C(=O)C3=CC=CC(=C23)C1)[C@@H]4CN5CCC4CC5	296.4	1.35	8.57	23.6	1	0	0	CHEMBL1189679	2.66	2	0	1	22	0.12	0.8	1

Substance	MATE1 substrate*	MATE2 substrate*	OCT1 substrate*	OCT2 substrate*	Therapeutic group	SMILES	Molecular weight	logD at pH 7.4	pKa (basic)	TPSA	Net Charge	Quarternary N	Zwitterionic	ChEMBL ID	AlogP	Hydrogen bound	Hydrogen bound	Aromatic rings	Heavy atoms	Natprod likeness score	Rqed (weighted)	Rotable bounds
Palonosetron (R,R)	0	0	0		5HT3 Antagonist	C1C[C@@H]2CN(C(=O)C3=CC=CC(=C23)C1)[C@@H]4CN5CCC4CC5	296.4	1.35	8.57	23.6	1	0	0	CHEMBL1720	2.66	2	0	1	22	0.12	0.8	1
Palonosetron (S,S)	0	0	0		5HT3 Antagonist	C1C[C@@H]2CN(C(=O)C3=CC=CC(=C23)C1)[C@@H]4CN5CCC4CC5	296.4	1.35	8.57	23.6	1	0	0	CHEMBL1720	2.66	2	0	1	22	0.12	0.8	1
Pancuronium	0	0			Muscle relaxant	CC(=O)O[C@H]1C[C@@H]2CC[C@@H]3[C@@H]([C@]2(C[C@@H]1[N+]4(CCCCC4)C)C)CC[C@]5([C@H]3C[C@@H]([C@@H]5OC(=O)C)[N+]6(CCCCC6)C)C	572.9	-3.27	14.00	52.6	2	1	0	CHEMBL1200757	6.11	4	0	0	41	1.46	0.3	4
Pantothenic acid	0	0			Vitamin	CC(C)(CO)[C@H](C(=O)NCCC(=O)O)O	219.2	-4.28	0.70	106.9	-1	0	0	CHEMBL1594	-1.04	4	4	0	15	0.5	0.46	6
Paracetamol	0	0	0	0	Analgesic	CC(=O)NC1=CC=C(C=C1)O	151.2	0.90	-4.40	49.3	0	0	0	CHEMBL112	1.35	2	2	1	11	-0.64	0.6	1
para-Methoxyamphetamine	0	0	0		Psychostimulant	CC(CC1=CC=C(C=C1)OC)N	165.2	-0.81	9.99	35.3	1	0	0	CHEMBL278663	1.58	2	1	1	12	-0.02	0.74	3
para-Methoxy-N-methylamphetamine	0	0	0	1	Psychostimulant	CC(CC1=CC=C(C=C1)OC)NC	179.3	-0.59	10.20	21.3	1	0	0	#NV	-0.75	2	1	1	13	#NV	#NV	4
Paraxanthine	0	0	0	0	Xanthine derivative	CN1C=NC2=C1C(=O)N(C(=O)N2)C	180.2	0.11	-1.20	67.2	0	0	0	CHEMBL1158	-1.04	5	1	2	13	-0.69	0.56	0
Paroxetine	0	0	0	0	Antidepressant	C1CNC[C@H]([C@@H]1C2=CC=C(C=C2)F)COC3=CC4=C(C=C3)OCO4	329.4	1.25	9.32	39.7	1	0	0	CHEMBL1708	3.33	4	1	2	24	-0.12	0.93	4
Pentamidine	1	1	0	0	Antiinfective	C1=CC(=CC=C1C(=N)N)OCCCCCOC2=CC=C(C=C2)C(=N)N	340.4	-2.50	12.13	118.2	2	0	0	CHEMBL55	2.88	4	4	2	25	-0.14	0.3	10
Pentobarbital	0	0			Antiepileptic	CCCC(C)C1(C(=O)NC(=O)NC1=O)CC	226.3	1.76	12.00	75.3	0	0	0	CHEMBL448	1.18	3	2	0	16	0.29	0.71	4
Perphenazine	0	0	0	0	Antipsychotic	C1CN(CCN1CCCN2C3=CC=CC=C3SC4=C2C=C(C=C4)Cl)CCO	404.0	2.45	8.61	55.3	1	0	0	CHEMBL567	3.94	5	1	2	27	-1.27	0.79	6
Pethidine	0	1	0	0	Opioid	CCOC(=O)C1(CCN(CC1)C)C2=CC=CC=C2	247.3	1.64	8.14	29.5	1	0	0	CHEMBL607	2.21	3	0	1	18	-0.56	0.77	3
Phenelzine	0	0	0	1	MAO inhibitor	C1=CC=C(C=C1)CCNN	136.2	0.36	8.18	38.1	1	0	0	CHEMBL1089	0.69	2	2	1	10	-0.43	0.47	3

Substance	MATE1 substrate*	MATE2 substrate*	OCT1 substrate*	OCT2 substrate*	Therapeutic group	SMILES	Molecular weight	logD at pH 7.4	pKa (basic)	TPSA	Net Charge	Quaternary N	Zwitterionic	ChEMBL ID	AlogP	Hydrogen bound	Hydrogen bound	Aromatic rings	Heavy atoms	Natprod likeness score	Rqed (weighted)	Rotable bounds
Phenformin	1	1	1	1	Antidiabetic	C1=CC=C(C=C1)CCN=C(N)N=C(N)N	205.3	-3.55	12.31	102.8	2	0	0	CHEMBL170988	0.24	2	5	1	15	-0.14	0.36	3
Phentermine	0	0	0	1	Psychostimulant	CC(C)(CC1=CC=CC=C1)N	149.2	-0.56	10.27	26.0	1	0	0	CHEMBL1574	1.97	1	1	1	11	0.11	0.68	2
Phenylalanine	0	0	0		Amino acid	C1=CC=C(C=C1)C[C@@H](C(=O)O)N	165.2	-1.19	9.45	63.3	0	0	1	CHEMBL301523	0.64	2	2	1	12	0.48	0.69	3
Phenylephrine	1	0	1	1	Symphatomimetic	CNC[C@@H](C1=CC(=CC=C1)O)O	167.2	-1.35	9.69	52.5	1	0	0	CHEMBL1200339	0.65	3	3	1	12	0.74	0.62	3
Phenylephrine (L)	1	1		1	Sympathomimetic	CNC[C@@H](C1=CC(=CC=C1)O)O	167.2	-1.35	9.69	52.5	1	0	0	CHEMBL1200339	0.65	3	3	1	12	0.74	0.62	3
Phenylephrine (S)	1	0			Sympathomimetic	CNC[C@@H](C1=CC(=CC=C1)O)O	167.2	-1.35	9.69	52.5	1	0	0	CHEMBL1200339	0.65	3	3	1	12	0.74	0.62	3
Phenylethanol	0	0			Experimental chemical	C1=CC=C(C=C1)CCO	122.2	1.49	-2.50	20.2	0	0	0	CHEMBL448500	1.22	1	1	1	9	0.27	0.62	2
Pilocarpine	0	0	0	1	(Anti)cholinergic	CC[C@H]1[C@H](COC1=O)CC2=CN=CN2C	208.3	0.86	6.94	44.1	0	0	0	CHEMBL1213136	1.16	4	0	1	15	1.25	0.7	3
Pimozide	0	0			Antipsychotic	C1CN(CCC1N2C3=CC=CC=C3NC2=O)CCCC(C4=CC=C(C=C4)F)C5=CC=C(C=C5)F	461.6	4.33	8.88	35.6	1	0	0	CHEMBL1423	5.86	3	1	4	34	-1.03	0.37	7
Pindolol	0	0	0	1	Beta blocker	CC(C)NCC(COC1=CC=CC2=C1C=CN2)O	248.3	-0.16	9.27	57.3	1	0	0	CHEMBL1530633	1.91	3	3	2	18	-0.38	0.73	6
Pinoline	0	0	0	1	Endobiotic	COC1=CC2=C(C=C1)NC3=C2CCNC3	202.3	-0.26	9.10	37.1	1	0	0	CHEMBL266084	1.82	2	2	2	15	-0.06	0.74	1
Pioglitazone	0	0	0	0	Antidiabetic	CCC1=CN=C(C=C1)CCOC2=CC=C(C=C2)CC3C(=O)NC(=O)S3	356.4	3.28	5.63	93.6	-1	0	0	CHEMBL595	3.16	5	1	2	25	-0.47	0.83	7
Pipamperone	0	0	0	0	Antipsychotic	C1CCN(CC1)C2(CCN(CC2)CCCC(=O)C3=CC=C(C=C3)F)C(=O)N	375.5	0.81	8.40	66.6	1	0	0	CHEMBL440294	2.59	4	1	1	27	-0.94	0.74	7
Piperacillin	0	0			Antibiotic	CCN1CCN(C(=O)C1=O)C(=O)N[C@H](C2=CC=CC=C2)C(=O)N[C@H]3[C@@H]4N(C3=O)[C@H](C(S4)(C)C)C(=O)O	517.6	-3.78	-4.30	181.7	-1	0	0	CHEMBL1200820	-0.24	7	3	1	36	0.03	0.34	6
Piperazine	0	0		0	Experimental chemical	C1CNCCN1	86.1	-2.86	9.56	24.1	1	0	0	CHEMBL1412	-0.82	2	2	0	6	0.06	0.4	0
Pirbuterol	1	1	1	1	Symphatomimetic	CC(C)(C)NCC(C1=NC(=C(C=C1)O)CO)O	240.3	-1.54	9.51	85.6	1	0	0	CHEMBL1094966	0.7	5	4	1	17	0.2	0.62	4

Substance	MATE1 substrate*	MATE2 substrate*	OCT1 substrate*	OCT2 substrate*	Therapeutic group	SMILES	Molecular weight	logD at pH 7.4	pKa (basic)	TPSA	Net Charge	Quaternary N	Zwitterionic	ChEMBL ID	AlogP	Hydrogen bound	Hydrogen bound	Aromatic rings	Heavy atoms	Natprod likeness score	Rqed (weighted)	Rotable bounds
Pirenzepine	1	0	1	0	(Anti)cholinergic	CN1CCN(CC1)CC(=O)N2C3=CC=CC=C3C(=O)NC4=C2N=CC=C4	351.4	0.40	7.83	68.8	1	0	0	CHEMBL1531864	1.56	5	1	2	26	-1.04	0.89	2
Piritramide	0	0	0	0	Opioid	C1CCN(CC1)C2(CCN(CC2)CCC(C#N)(C3=CC=CC=C3)C4=CC=CC=C4)C(=O)N	430.6	2.08	8.68	73.4	1	0	0	CHEMBL559288	3.69	4	1	2	32	-0.58	0.73	7
Pomalidomide	0	0			Oncology	C1CC(=O)NC(=O)C1N2C(=O)C3=C(C2=O)C(=CC=C3)N	273.2	-0.16	1.56	109.6	0	0	0	CHEMBL43452	-0.33	5	2	1	20	-0.21	0.54	1
p-Phenylenediamine	0	0	0	0	Experimental chemical	C1=CC(=CC=C1N)N	108.1	0.27	6.46	52.0	0	0	0	CHEMBL403741	0.85	2	2	1	8	-0.08	0.48	0
Practolol	1	1	1	1	Beta blocker	CC(C)NCC(COC1=CC=C(C=C1)NC(=O)C)O	266.3	-1.02	9.27	70.6	1	0	0	CHEMBL6995	1.38	4	3	1	19	-0.91	0.7	7
Prazosin	0	0	0	0	Alpha1 blocker	COC1=C(C=C2C(=C1)C(=NC(=N2)N3CCN(CC3)C(=O)C4=CC=CO4)N)OC	383.4	0.94	8.04	107.0	1	0	0	CHEMBL1558	1.78	8	1	3	28	-1.29	0.73	4
Prenalterol	1	0	1	1	Sympathomimetic	CC(C)NC[C@@H](COC1=CC=C(C=C1)O)O	225.3	-0.56	9.18	61.7	1	0	0	CHEMBL3085478	1.13	4	3	1	16	-0.13	0.68	6
Pridinol	0	0			(Anti)cholinergic	C1CCN(CC1)CCC(C2=CC=CC=C2)(C3=CC=CC=C3)O	295.4	1.65	9.47	23.5	1	0	0	CHEMBL404215	3.8	2	1	2	22	-0.6	0.91	5
Primaquine	0	0	0	0	Antiinfective	CC(CCCN)NC1=C2C(=CC(=C1)OC)C=CC=N2	259.4	-0.96	10.20	60.2	1	0	0	CHEMBL506	2.78	4	2	2	19	-0.43	0.84	6
Procainamide	1	1	1	1	Antiarrhythmic	CCN(CC)CCNC(=O)C1=CC=C(C=C1)N	235.3	-0.70	9.04	58.4	1	0	0	CHEMBL640	1.34	3	2	1	17	-1.4	0.73	6
Proguanil	1	1	1	1	Antiinfective	CC(C)N=C(N)/N=C(\N)/NC1=CC=C(C=C1)Cl	253.7	-0.44	13.97	88.8	2	0	0	CHEMBL1377	2.21	2	5	1	17	-0.85	0.41	2
Propafenone	0	0	0	0	Antiarrhythmic	CCCNCC(COC1=CC=CC=C1C(=O)CCC2=CC=CC=C2)O	341.5	1.63	9.32	58.6	1	0	0	CHEMBL1201063	3.24	4	2	2	25	-0.36	0.49	11
Propafenone (R)	0	0	0		Antiarrhythmic	CCCNCC(COC1=CC=CC=C1C(=O)CCC2=CC=CC=C2)O	341.5	1.63	9.32	58.6	1	0	0	CHEMBL1201063	3.24	4	2	2	25	-0.36	0.49	11
Propafenone (S)	0	0	0		Antiarrhythmic	CCCNCC(COC1=CC=CC=C1C(=O)CCC2=CC=CC=C2)O	341.5	1.63	9.32	58.6	1	0	0	CHEMBL1256654	3.24	4	2	2	25	-0.36	0.49	11

Substance	MATE1 substrate*	MATE2 substrate*	OCT1 substrate*	OCT2 substrate*	Therapeutic group	SMILES	Molecular weight	logD at pH 7.4	pKa (basic)	TPSA	Net Charge	Quaternary N	Zwitterionic	ChEMBL ID	AlogP	Hydrogen bound	Hydrogen bound	Aromatic rings	Heavy atoms	Natprod likeness score	Rqed (weighted)	Rotable bounds
Propantheline	1	1	1	1	(Anti)cholinergic	CC(C)[N+](C)(CCOC(=O)C1C2=CC=CC=C2OC3=CC=CC=C13)C(C)C	368.5	0.36	14.00	35.5	1	1	0	CHEMBL1180725	4.73	3	0	2	27	0.1	0.55	6
Propionylcarnitine	0	0	0	0	Carnitine ester	CCC(=O)OC(CC(=O)[O-])C[N+](C)(C)C	217.3	-2.98	14.00	66.4	0	1	1	CHEMBL4743301	0.49	3	1	0	15	1.02	0.52	6
Propiverine	0	0		0	(Anti)cholinergic	CCCOC(C1=CC=CC=C1)(C2=CC=CC=C2)C(=O)OC3CCN(CC3)C	367.5	2.92	8.72	38.8	1	0	0	CHEMBL1078261	3.99	4	0	2	27	-0.2	0.69	7
Propofol	0	0	0	0	Others	CC(C)C1=C(C(=CC=C1)C(C)C)O	178.3	4.16	-5.00	20.2	0	0	0	CHEMBL526	3.64	1	1	1	13	0.28	0.73	2
Propranolol	0	0	0	0	Beta blocker	CC(C)NCC(COC1=CC=CC2=CC=CC=C21)O	259.3	0.73	9.27	41.5	1	0	0	CHEMBL1671	2.58	3	2	2	19	-0.47	0.84	6
Propranolol (R)	0	0	0		Beta blocker	CC(C)NCC(COC1=CC=CC2=CC=CC=C21)O	259.3	0.73	9.27	41.5	1	0	0	CHEMBL1358662	2.58	3	2	2	19	-0.47	0.84	6
Propranolol (S)	0	0			Beta blocker	CC(C)NCC(COC1=CC=CC2=CC=CC=C21)O	259.3	0.73	9.27	41.5	1	0	0	CHEMBL1671	2.58	3	2	2	19	-0.47	0.84	6
Prostaglandin E2	0	0	0		Others	CCCCC[C@@H](/C=C/[C@H]1[C@@H](CC(=O)[C@@H]1C/C=C\CCCC(=O)O)O)O	352.5	0.26	-1.60	94.8	-1	0	0	ChEMBL548	3.25	4	3	0	25	2.03	0.37	12
Prostaglandin F2alpha	0	0	0		Others	CCCCC[C@@H](/C=C/[C@H]1[C@@H](C[C@@H]([C@@H]1C/C=C\CCCC(=O)O)O)O)O	354.5	-0.31	-1.60	98.0	-1	0	0	CHEMBL815	3.04	4	4	0	25	1.94	0.32	12
Prucalopride	0	0			Others	COCCCN1CCC(CC1)NC(=O)C2=CC(=C(C3=C2OCC3)N)Cl	367.9	-0.55	8.68	76.8	1	0	0	CHEMBL117287	2.09	5	2	1	25	-1.22	0.59	6
Pseudoephedrine	0	0	0		Sympathomimetic	C[C@@H]([C@H](C1=CC=CC=C1)O)NC	165.2	-0.78	9.52	32.3	1	0	0	CHEMBL1590	1.33	2	2	1	12	0.43	0.71	3
Putrescine	0	0	0	0	Biogenic amine	C(CCN)CN	88.2	-6.05	10.51	52.0	2	0	0	CHEMBL1528028	-0.32	2	2	0	6	0.57	0.46	3
Pyrazinamide	0	0	0	0	Antibiotic	C1=CN=C(C=N1)C(=O)N	123.1	-1.23	-0.55	68.9	0	0	0	CHEMBL614	-0.42	3	1	1	9	-1.41	0.55	1
Pyridoxal	0	0	0	0	Vitamin	CC1=NC=C(C(=C1O)C=O)CO	167.2	-0.08	4.31	70.4	0	0	0	CHEMBL102970	0.4	4	2	1	12	0.7	0.63	2
Pyridoxamine	1	0	0	0	Vitamin	CC1=NC=C(C(=C1O)CN)CO	168.2	-1.83	9.60	79.4	1	0	0	CHEMBL2361504	0.05	4	3	1	12	0.51	0.58	2
Pyridoxin	1	0	0	0	Vitamin	CC1=NC=C(C(=C1O)CO)CO	169.2	-0.96	5.19	73.6	0	0	0	CHEMBL1364	0.08	4	3	1	12	0.64	0.59	2

Substance	MATE1 substrate*	MATE2 substrate*	OCT1 substrate*	OCT2 substrate*	Therapeutic group	SMILES	Molecular weight	logD at pH 7.4	pKa (basic)	TPSA	Net Charge	Quarternary N	Zwitterionic	ChEMBL ID	AlogP	Hydrogen bound	Hydrogen bound	Aromatic rings	Heavy atoms	Natprod likeness score	Rqed (weighted)	Rotable bounds
Pyrilamine	0	0	1	1	Antihistaminic	CN(C)CCN(CC1=CC=C(C=C1)OC)C2=CC=CC=N2	285.4	1.66	8.76	28.6	1	0	0	CHEMBL1201006	2.66	4	0	2	21	-1.16	0.78	7
Pyrimethamine	0	0	0	0	Antiinfective	CCC1=C(C(=NC(=N1)N)N)C2=CC=C(C=C2)Cl	248.7	2.23	7.77	77.8	1	0	0	CHEMBL36	2.52	4	2	2	17	-0.93	0.86	2
Pyrithiamine	1	1	1	1	Vitamin	CC1=C(C=CC=[N+]1CC2=CN=C(N=C2N)C)CCO	259.3	-3.87	5.54	75.9	1	1	0	CHEMBL1229798	0.55	4	2	2	19	-0.05	0.79	4
Quetiapine	0	0			Antipsychotic	C1CN(CCN1CCOCCO)C2=NC3=CC=CC=C3SC4=CC=CC=C42	383.5	2.29	7.76	73.6	1	0	0	CHEMBL3188993	2.86	6	1	2	27	-0.87	0.8	5
Quinidine	0	0	0	0	Herbal	COC1=CC2=C(C=CN=C2C=C1)[C@@H]([C@H]3C[C@@H]4CCN3C[C@@H]4C=C)O	324.4	1.34	8.55	45.6	1	0	0	CHEMBL1200437	3.17	4	1	2	24	0.83	0.88	4
Quinine	0	0	0	0	Antiinfective	COC1=CC2=C(C=CN=C2C=C1)[C@H]([C@@H]3C[C@@H]4CCN3C[C@@H]4C=C)O	324.4	1.34	8.55	45.6	1	0	0	CHEMBL2359966	3.17	4	1	2	24	0.83	0.88	4
Ractopamine	1	0	1	1	Sympathomimetic	CC(CCC1=CC=C(C=C1)O)NCC(C2=CC=C(C=C2)O)O	301.4	0.88	9.89	72.7	1	0	0	CHEMBL2105358	2.74	4	4	2	22	0.13	0.63	7
Raloxifene	0	0			Others	C1CCN(CC1)CCOC2=CC=C(C=C2)C(=O)C3=C(SC4=C3C=CC(=C4)O)C5=CC=C(C=C5)O	473.6	4.86	8.38	98.2	1	0	0	CHEMBL81	6.08	6	2	4	34	-0.54	0.32	7
Ranitidine	1	1	1	1	Antihistaminic	CN/C(=C\[N+](=O)[O-])/NCCSCC1=CC=C(O1)CN(C)C	314.4	0.04	8.30	108.9	1	0	1	CHEMBL2110372	1.46	7	2	1	21	-1.29	0.38	10
Ranolazine	0	0			Others	CC1=C(C(=CC=C1)C)NC(=O)CN2CCN(CC2)CC(COC3=CC=CC=C3OC)O	427.5	2.34	7.73	74.3	1	0	0	CHEMBL1526084	2.31	6	2	2	31	-1.32	0.64	9
Rasagiline	0	1	0	1	MAO inhibitor	C#CCN[C@@H]1CCC2=CC=CC=C12	171.2	1.83	7.70	12.0	1	0	0	CHEMBL887	1.9	1	1	1	13	-0.63	0.67	2
Rasagiline (S)	0	0			MAO inhibitor	C#CCN[C@@H]1CCC2=CC=CC=C12	171.2	1.83	7.70	12.0	1	0	0	CHEMBL887	1.9	1	1	1	13	-0.63	0.67	2
Reboxetine	0	0		0	Antidepressant	CCOC1=CC=CC=C1O[C@@H]([C@H]2CNCCO2)C3=CC=CC=C3	313.4	2.93	7.49	39.7	1	0	0	CHEMBL383921	3.19	4	1	2	23	-0.17	0.89	6

Substance	MATE1 substrate*	MATE2 substrate*	OCT1 substrate*	OCT2 substrate*	Therapeutic group	SMILES	Molecular weight	logD at pH 7.4	pKa (basic)	TPSA	Net Charge	Quarternary N	Zwitterionic	ChEMBL ID	AlogP	Hydrogen bound	Hydrogen bound	Aromatic rings	Heavy atoms	Natprod likeness score	Rqed (weighted)	Rotable bounds
Remoxipride	0	0	1	1	Antipsychotic	CCN1CCC[C@H]1CNC(=O)C2=C(C(=CC(=C2OC)Br)OC	371.3	0.74	9.00	60.8	1	0	0	CHEMBL3989556	2.68	4	1	1	22	-0.68	0.84	6
Reproterol	1	1	1	0	Sympathomimetic	CN1C2=C(C(=O)N(C1=O)C)N(C=N2)CCCNCC(C3=CC(=CC(=C3)O)O)O	389.4	-2.34	9.66	131.2	1	0	0	CHEMBL1095607	-0.44	10	4	3	28	-0.6	0.4	7
Reserpine	0	0	0	0	Antihypertensive	CO[C@H]1[C@@H](C[C@@H]2CN3CCC4=C([C@H]3C[C@@H]2[C@@H]1C(=O)OC)NC5=C4C=CC(=C5)OC)OC(=O)C6=CC(=C(C(=C6)OC)OC)OC	608.7	3.24	7.39	117.8	1	0	0	CHEMBL772	4.17	10	1	3	44	0.93	0.37	8
Revefenacin	0	0			(Anti)cholinergic	CN(CCN1CCC(CC1)OC(=O)NC2=CC=CC=C2C3=CC=CC=C3)C(=O)C4=CC=C(C=C4)CN5CCC(CC5)C(=O)N	597.8	1.46	8.85	108.2	2	0	0	CHEMBL3833319	4.84	6	2	3	44	-1.3	0.35	10
Riboflavin	0	1		0	Vitamin	CC1=CC2=C(C=C1C)N(C3=NC(=O)NC(=O)C3=N2)C[C@@H]([C@@H]([C@@H](CO)O)O)O	376.4	-2.34	0.77	155.1	-1	0	0	CHEMBL1534	-1.72	9	5	1	27	0.07	0.33	5
Rifampicin	0	0		0	Antibiotic	C[C@H]1/C=C/C=C(\C(=O)NC2=C(C(=C3C(=C2O)C(=C(C4=C3C(=O)[C@](O4)(O/C=C/[C@@H]([C@H]([C@H]([C@@H]([C@@H]([C@@H]([C@H]1O)C)O)C)OC(=O)C)C)OC)C)C)O)O)/C=N/N5CCN(CC5)C)/C	823.0	3.46	6.91	220.2	0	0	1	CHEMBL374478	4.34	15	6	2	59	1.66	0.11	4
Risperidone	0	0	0	1	Antipsychotic	CC1=C(C(=O)N2CCCCC2=N1)CCN3CCC(CC3)C4=NOC5=C4C=CC(=C5)F	410.5	1.25	8.76	61.9	1	0	0	CHEMBL85	3.59	6	0	3	30	-1.47	0.66	4
Ritodrine	1	1	1	1	Sympathomimetic	C[C@H]([C@H](C1=CC=C(C=C1)O)O)NCCC2=CC=C(C=C2)O	287.4	0.52	10.30	72.7	1	0	0	CHEMBL489553	2.35	4	4	2	21	0.23	0.66	6

Substance	MATE1 substrate*	MATE2 substrate*	OCT1 substrate*	OCT2 substrate*	Therapeutic group	SMILES	Molecular weight	logD at pH 7.4	pKa (basic)	TPSA	Net Charge	Quaternary N	Zwitterionic	ChEMBL ID	AlogP	Hydrogen bound	Hydrogen bound	Aromatic rings	Heavy atoms	Natprod likeness score	Rqed (weighted)	Rotable bounds
Rivastigmine	0	0	0	0	Cholinesterase inhibitor	CCN(C)C(=O)OC1=CC=CC(=C1)[C@H](C)N(C)C	250.3	1.00	8.80	32.8	1	0	0	CHEMBL636	2.76	3	0	1	18	-0.98	0.82	4
Rizatriptan	1	1	1	1	Triptan	CN(C)CCC1=CNC2=C1C=C(C=C2)CN3C=NC=N3	269.4	-0.40	9.58	49.7	1	0	0	CHEMBL1201032	1.91	4	1	3	20	-1.44	0.77	5
Ropinirole	0	0			Dopamine agonist	CCCN(CCC)CCC1=C2CC(=O)NC2=CC=C1	260.4	0.40	10.12	32.3	1	0	0	CHEMBL1200411	2.85	2	1	1	19	-0.47	0.82	7
Ropivacaine	0	0			Local anesthetic	CCCN1CCCC[C@H]1C(=O)NC2=C(C=CC=C2C)C	274.4	3.53	7.80	32.3	1	0	0	CHEMBL1077896	3.51	2	1	1	20	-1.37	0.91	4
Rucaparib	1	1	1	0	Cytostatic	CNCC1=CC=C(C=C1)C2=C3CCNC(=O)C4=C3C(=CC(=C4)F)N2	323.4	0.55	9.32	56.9	1	0	0	CHEMBL1173055	2.98	2	3	3	24	0.04	0.69	3
Ruxolitinib	0	0			JAK inhibitor	C1CCC(C1)[C@@H](CC#N)N2C=C(C=N2)C3=C4C=CNC4=NC=N3	306.4	2.48	5.01	83.2	0	0	0	CHEMBL1789941	3.47	5	1	3	23	-0.9	0.8	4
Salbutamol	1	1	1	1	Sympathomimetic	CC(C)(C)NCC(C1=CC(=C(C=C1)O)CO)O	239.2	-1.32	9.40	72.7	1	0	0	CHEMBL714	1.31	4	4	1	17	0.56	0.64	4
Salbutamol (R)	1	1	1	1	Sympathomimetic	CC(C)(C)NCC(C1=CC(=C(C=C1)O)CO)O	239.2	-1.32	9.40	72.7	1	0	0	CHEMBL714	1.31	4	4	1	17	0.56	0.64	4
Salbutamol (S)	1	1	1	1	Sympathomimetic	CC(C)(C)NCC(C1=CC(=C(C=C1)O)CO)O	239.2	-1.32	9.40	72.7	1	0	0	CHEMBL714	1.31	4	4	1	17	0.56	0.64	4
Salmeterol	0	0	0	0	Sympathomimetic	C1=CC=C(C=C1)CCCCOCCCCCCNCC(C2=CC(=C(C=C2)O)CO)O	415.6	1.95	9.40	82.0	1	0	0	CHEMBL1263	4.11	5	4	2	30	0.2	0.31	16
Salsolidine	0	0	1	1	Herbal	CC1C2=CC(=C(C=C2CCN1)OC)OC	207.3	0.17	8.89	30.5	1	0	0	CHEMBL1196026	1.91	3	1	1	15	1.08	0.8	2
Salsolinol	1	1	1	1	Endobiotic	C[C@H]1C2=CC(=C(C=C2CCN1)O)O	179.2	0.15	8.51	52.5	1	0	0	CHEMBL1193327	1.3	3	3	1	13	1.75	0.53	0
Sarcosine	0	0	0	0	Endobiotic	CNCC(=O)O	89.1	-3.19	10.35	49.3	0	0	1	CHEMBL304383	-0.71	2	2	0	6	0.52	0.47	2
Scopolamine	0	0	0	0	(Anti)cholinergic	CN1[C@@H]2CC(C[C@H]1[C@H]3[C@H]2O3)OC(=O)[C@H](CO)C4=CC=CC=C4	303.4	0.85	6.45	62.3	0	0	0	CHEMBL2251240	0.92	5	1	1	22	1.36	0.66	4
Selegiline	0	0	0	0	MAO inhibitor	C[C@H](CC1=CC=CC=C1)N(C)CC#C	187.3	1.53	8.70	3.2	1	0	0	CHEMBL1200904	2.18	1	0	1	14	-0.95	0.65	4
Selegiline (R)	0	1		0	MAO inhibitor	CC(CC1=CC=CC=C1)N(C)CC#C	187.3	1.53	8.70	3.2	1	0	0	CHEMBL972	2.18	1	0	1	14	-0.95	0.65	4

Substance	MATE1 substrate*	MATE2 substrate*	OCT1 substrate*	OCT2 substrate*	Therapeutic group	SMILES	Molecular weight	logD at pH 7.4	pKa (basic)	TPSA	Net Charge	Quarternary N	Zwitterionic	ChEMBL ID	AlogP	Hydrogen bound	Hydrogen bound	Aromatic rings	Heavy atoms	Natprod likeness score	Rqed (weighted)	Rotable bounds
Selegiline (S)	0	0			MAO inhibitor	CC(CC1=CC=CC=C1)N(C)CC#C	187.3	1.53	8.70	3.2	1	0	0	CHEMBL1200904	2.18	1	0	1	14	-0.95	0.65	4
Sematilide	1	1	1	0	Antiarrhythmic	CCN(CC)CCNC(=O)C1=CC=C(C=C1)NS(=O)(=O)C	313.4	-1.38	9.07	86.9	1	0	0	CHEMBL95804	1.13	4	2	1	21	-1.83	0.75	8
Sepantronium	1	1	1	1	Cytostatic	CC1=[N+](C2=C(N1CCOC)C(=O)C3=CC=CC=C3C2=O)CC4=NC=CN=C4	363.4	-3.72	14.00	78.0	1	1	0	CHEMBL2110734	1.34	6	0	3	27	-0.55	0.5	5
Serotonin	1	1	1	0	Biogenic amine	C1=CC2=C(C=C1O)C(=CN2)CCN	176.2	-1.05	10.02	62.0	1	0	0	CHEMBL535832	1.37	2	3	2	13	0.64	0.65	2
Sertraline	0	0		0	Antidepressant	CN[C@H]1CC[C@H](C2=CC=CC=C12)C3=CC(=C(C=C3)Cl)Cl	306.2	3.02	9.56	12.0	1	0	0	CHEMBL809	5.18	1	1	2	20	0.12	0.81	2
Sitagliptin	1	1		0	Antidiabetic	C1CN2C(=NN=C2C(F)(F)F)CN1C(=O)C[C@@H](CC3=CC(=C(C=C3F)F)F)N	407.3	-0.14	8.78	77.0	1	0	0	CHEMBL1201174	2.02	5	1	2	28	-1.4	0.62	4
Solifenacin	0	0			(Anti)cholinergic	C1CN2CCC1[C@H](C2)OC(=O)N3CCC4=CC=CC=C4[C@@H]3C5=CC=CC=C5	362.5	2.47	8.88	32.8	1	0	0	CHEMBL1200803	3.86	3	0	2	27	-0.06	0.81	2
Sotalol	1	0	1	1	Antiarrhythmic	CC(C)NCC(C1=CC=C(C=C1)NS(=O)(=O)C)O	272.4	-1.98	9.64	86.8	1	0	0	CHEMBL471	1.09	4	3	1	18	-0.9	0.72	6
Sparteine (-)	1	0	1	1	Antiarrhythmic	C1CCN2C[C@@H]3C[C@H]([C@H]2C1)CN4[C@H]3CCCC4	234.4	0.24	9.06	6.5	1	0	0	CHEMBL1908348	2.35	2	0	0	17	0.61	0.63	0
Sparteine (+)	0	0	1	1	Antiarrhythmic	C1CCN2C[C@H]3C[C@@H]([C@H]2C1)CN4[C@H]3CCCC4	234.4	0.24	9.06	6.5	1	0	0	CHEMBL1908348	2.35	2	0	0	17	0.61	0.63	0
Spermidine	0	0	0	0	Biogenic amine	C(CCNCCCN)CN	145.3	-7.35	10.61	64.1	3	0	0	CHEMBL19612	-0.34	3	3	0	10	0.22	0.43	7
Spermine	0	0			Endobiotic	C(CCNCCCN)CNCCCN	202.4	-8.65	10.70	76.1	4	0	0	CHEMBL23194	-0.36	4	4	0	14	0.09	0.35	11
Stachydrine	0	0	0	0	Herbal	C[N+]1(CCC[C@H]1C(=O)[O-])C	143.2	-3.11	14.00	40.1	0	1	1	CHEMBL166795	-1.02	2	0	0	10	1.35	0.44	1
Strychnine	0	0	0	0	Herbal	C1CN2CC3=CCO[C@H]4CC(=O)N5[C@H]6[C@H]4[C@H]3C[C@H]2[	334.4	-0.65	8.97	32.8	1	0	0	CHEMBL2103753	2.09	3	0	1	25	3.05	0.68	0

Substance	MATE1 substrate*	MATE2 substrate*	OCT1 substrate*	OCT2 substrate*	Therapeutic group	SMILES	Molecular weight	logD at pH 7.4	pKa (basic)	TPSA	Net Charge	Quarternary N	Zwitterionic	ChEMBL ID	AlogP	Hydrogen bound	Hydrogen bound	Aromatic rings	Heavy atoms	Natprod likeness score	Rqed (weighted)	Rotable bounds
						C@@]61C7=CC=CC=C75																
Succinylcarnitine	0	0	0		Carnitine ester	C[N+](C)(C)C[C@@H](CC(=O)[O-])OC(=O)CCC(=O)O	261.3	-6.80	14.00	103.7	-1	1	1	#NV	-7.5	5	1	0	18	#NV	#NV	9
Sufentanil	0	0	0	0	Opioid	CCC(=O)N(C1=CC=CC=C1)C2(CCN(CC2)CCC3=CC=CS3)COC	386.6	2.41	8.56	61.0	1	0	0	CHEMBL1201163	4.21	4	0	2	27	-1.18	0.68	8
Sulpiride	1	1	1	1	Antipsychotic	CCN1CCCC1CNC(=O)C2=C(C=CC(=C2)S(=O)(=O)N)OC	341.4	-1.26	8.97	110.1	1	0	0	CHEMBL26	0.56	5	2	1	23	-1.62	0.79	6
Sulpiride (S)	1	1	1	1	Antipsychotic	CCN1CCCC1CNC(=O)C2=C(C=CC(=C2)S(=O)(=O)N)OC	341.4	-1.26	8.97	110.1	1	0	0	CHEMBL26	0.56	5	2	1	23	-1.62	0.79	6
Sumatriptan	1	1	1	1	Triptan	CNS(=O)(=O)CC1=CC2=C(C=C1)NC=C2CCN(C)C	295.4	-1.26	9.56	73.6	1	0	0	CHEMBL1201150	1.32	3	2	2	20	-1.1	0.84	6
Synephrine	1	0	1	1	Sympathomimetic	CNCC(C1=CC=C(C=C1)O)O	167.2	-1.39	9.15	52.5	1	0	0	CHEMBL33720	0.64	3	3	1	12	0.84	0.62	3
Talinolol	1	1	0	0	Beta blocker	CC(C)(C)NCC(COC1=CC=C(C=C1)NC(=O)NC2CCCCC2)O	363.5	0.86	9.36	82.6	1	0	0	CHEMBL152067	3.27	4	4	1	26	-1.11	0.6	7
Tamoxifen	0	0	0	0	Oncology	CC/C(=C(\C1=CC=CC=C1)/C2=CC=C(C=C2)OCCN(C)C)/C3=CC=CC=C3	371.5	4.97	8.76	12.5	1	0	0	CHEMBL83	6	2	0	3	28	-0.4	0.45	8
Tamsulosin	0	0	1	0	Alpha1 blocker	CCOC1=CC=CC=C1OCCN[C@H](C)CC2=CC(=C(C=C2)OC)S(=O)(=O)N	408.5	1.02	8.84	108.3	1	0	0	CHEMBL1200914	2.34	6	2	2	28	-1.02	0.55	11
Tapentadol	0	0	0	0	Opioid	CC[C@@H](C1=CC(=CC=C1)O)[C@@H](C)CN(C)C	221.3	1.07	9.60	23.5	1	0	0	CHEMBL1201776	3.08	2	1	1	16	-0.02	0.83	5
Taurine	0	0	0	0	Endobiotic	C(CS(=O)(=O)O)N	125.1	-2.62	9.34	88.8	0	0	1	CHEMBL239243	-1.17	3	2	0	7	0.61	0.46	2
Temozolomide	0	0	0		Cytostatic	CN1C(=O)N2C=NC(=C2N=N1)C(=O)N	194.2	-0.28	-3.60	105.9	0	0	0	CHEMBL810	-2.08	7	1	2	14	-1.63	0.56	1
Terazosin	0	0	0	0	Alpha1 blocker	COC1=C(C=C2C(=C1)C(=NC(=N2)N3CCN(CC3)C(=O)C4CCCO4)N)OC	387.4	0.47	8.04	103.0	1	0	0	CHEMBL3989562	1.06	8	1	2	28	-0.89	0.83	4

Substance	MATE1 substrate*	MATE2 substrate*	OCT1 substrate*	OCT2 substrate*	Therapeutic group	SMILES	Molecular weight	logD at pH 7.4	pKa (basic)	TPSA	Net Charge	Quarternary N	Zwitterionic	ChEMBL ID	AlogP	Hydrogen bound	Hydrogen bound	Aromatic rings	Heavy atoms	Natprod likeness score	Rqed (weighted)	Rotable bounds
Terbutaline	1	1	1	1	Sympathomimetic	CC(C)(C)NCC(C1=CC(=CC(=C1)O)O)O	225.3	-0.73	9.76	72.7	1	0	0	CHEMBL1315867	1.52	4	4	1	16	0.52	0.63	3
Terfenadine	0	0			Antihistaminic	CC(C)(C)C1=CC=C(C=C1)C(CCCN2CCC(CC2)C(C3=CC=CC=C3)(C4=CC=CC=C4)O)O	471.7	4.66	9.22	43.7	1	0	0	CHEMBL17157	6.45	3	2	3	35	-0.46	0.4	8
Tetracaine	0	0	0		Local anesthetic	CCCCNC1=CC=C(C=C1)C(=O)OCCN(C)C	264.4	1.74	8.42	41.6	1	0	0	CHEMBL1255654	2.62	4	1	1	19	-0.97	0.58	8
Tetracycline	0	0	0	0	Antibiotic	C[C@@]1([C@H]2C[C@H]3[C@@H](C(=O)C(=C([C@]3(C(=O)C2=C(C4=C1C=CC=C4O)O)O)O)C(=O)N)N(C)C)O	444.4	-6.07	7.97	181.6	-1	0	0	CHEMBL454950	-0.21	9	6	1	32	1.7	0.34	2
Tetraethylammonium	1	1	1	1	Experimental chemical	CC[N+](CC)(CC)CC	130.3	-2.54	14.00	0.0	1	1	0	CHEMBL9324	1.88	0	0	0	9	-0.08	0.51	4
Thalidomide	0	1	0	0	Others	C1CC(=O)NC(=O)C1N2C(=O)C3=CC=CC=C3C2=O	258.2	0.02	-6.40	83.6	0	0	0	CHEMBL426123	0.09	4	1	1	19	-0.24	0.72	1
Thiamine	1	1	1	1	Vitamin	CC1=C(SC=[N+]1CC2=CN=C(N=C2N)C)CCO	265.4	-3.10	5.54	104.2	1	1	0	CHEMBL1547	0.61	5	2	2	18	-0.1	0.79	4
Thiamine monophosphate	0	0	0	0	Vitamin	CC1=C(SC=[N+]1CC2=CN=C(N=C2N)C)CCOP(=O)(O)O	345.3	-5.14	5.51	160.5	1	1	0	CHEMBL2106811	0.72	6	3	2	22	0.02	0.52	6
Thyrotropin-Releasing-Hormon	0	0	0	0	Hormone	C1C[C@H](N(C1)C(=O)[C@H](CC2=CN=CN2)NC(=O)[C@@H]3CCC(=O)N3)C(=O)N	362.4	-3.35	6.86	150.3	0	0	0	CHEMBL1472	-1.81	5	4	1	26	-0.41	0.47	6
Tianeptine	0	0	0	0	Antidepressant	CN1C2=CC=CC=C2C(C3=C(S1(=O)=O)C=C(C=C3)Cl)NCCCCCCC(=O)O	437.0	1.43	7.81	86.7	0	0	1	CHEMBL1289110	4.19	4	2	2	29	-0.52	0.61	8
Tiglylcarnitine	0	0	0	0	Carnitine ester	C/C=C(\C)/C(=O)OC(CC(=O)[O-])C[N+](C)(C)C	243.3	-1.90	14.00	66.4	0	1	1	#NV	-2.24	3	0	0	17	#NV	#NV	7
Timolol	0	0	0	1	Beta blocker	CC(C)(C)NC[C@@H](COC1=NSN=C1N2CCOCC2)O	316.4	-0.61	9.36	108.0	1	0	0	CHEMBL1200870	0.5	8	2	1	21	-1.47	0.79	6
Timolol (R)	0	0			Beta blocker	CC(C)(C)NC[C@@H](COC1=NSN=C1N2CCOCC2)O	316.4	-0.61	9.36	108.0	1	0	0	CHEMBL1200870	0.5	8	2	1	21	-1.47	0.79	6

Substance	MATE1 substrate*	MATE2 substrate*	OCT1 substrate*	OCT2 substrate*	Therapeutic group	SMILES	Molecular weight	logD at pH 7.4	pKa (basic)	TPSA	Net Charge	Quarternary N	Zwitterionic	ChEMBL ID	AlogP	Hydrogen bound	Hydrogen bound	Aromatic rings	Heavy atoms	Natprod likeness score	Rqed (weighted)	Rotable bounds
Tiotropium	1	0	1	1	(Anti)cholinergic	C[N+]1([C@@H]2CC(C[C@H]1[C@H]3[C@@H]2O3)OC(=O)C(C4=CC=CS4)(C5=CC=CS5)O)C	392.5	-1.75	14.00	115.5	1	1	0	CHEMBL1900528	2.35	6	1	2	26	0.51	0.49	4
Tofacitinib	0	0	0	0	JAK inhibitor	C[C@@H]1CCN(C[C@@H]1N(C)C2=NC=NC3=C2C=CN3)C(=O)CC#N	312.4	0.87	7.53	88.9	1	0	0	CHEMBL2103743	1.54	5	1	2	23	-1.14	0.93	3
Tolterodine (R)	0	0		0	(Anti)cholinergic	CC1=CC(=C(C=C1)O)[C@H](CCN(C(C)C)C(C)C)C2=CC=CC=C2	325.5	2.78	10.90	23.5	1	0	0	CHEMBL1382	5.34	2	1	2	24	-0.36	0.74	7
Tolterodine (S)	0	0			(Anti)cholinergic	CC1=CC(=C(C=C1)O)[C@H](CCN(C(C)C)C(C)C)C2=CC=CC=C2	325.5	2.78	10.90	23.5	1	0	0	CHEMBL1200871	5.34	2	1	2	24	-0.36	0.74	7
Topotecan	1	0	0	0	Cytostatic	CC[C@@]1(C2=C(COC1=O)C(=O)N3CC4=CC5=C(C=CC(=C5CN(C)C)O)N=C4C3=C2)O	421.5	-2.38	9.12	103.2	1	0	0	CHEMBL305801	1.85	8	2	3	31	0.9	0.49	3
Tramadol	0	0	0	0	Opioid	CN(C)C[C@H]1CCCC[C@@]1(C2=CC(=CC=C2)OC)O	263.4	0.48	9.38	32.7	1	0	0	CHEMBL4059722	2.63	3	1	1	19	0.43	0.91	4
Tranylcypromine	0	0	0	0	MAO inhibitor	C1[C@H]([C@@H]1N)C2=CC=CC=C2	133.2	-0.16	8.90	26.0	1	0	0	CHEMBL1255743	1.5	1	1	1	10	0.42	0.62	1
Triethylentetramine	0	0	0	0	Others	C(CNCCNCCN)N	146.2	-6.30	9.77	76.1	2	0	0	CHEMBL609	-1.92	4	4	0	10	-0.09	0.32	7
Trimethoprim	0	0	1	0	Antibiotic	COC1=CC(=CC(=C1OC)OC)CC2=CN=C(N=C2N)N	290.3	1.10	7.16	105.5	0	0	0	CHEMBL1201080	1.26	7	2	2	21	0.07	0.85	5
Trimipramine	0	0	0	0	Antidepressant	CC(CN1C2=CC=CC=C2CCC3=CC=CC=C31)CN(C)C	294.4	2.75	9.42	6.5	1	0	0	CHEMBL1200948	4.12	2	0	2	22	-0.6	0.84	4
Tropicamide	0	0	0	0	(Anti)cholinergic	CCN(CC1=CC=NC=C1)C(=O)C(CO)C2=CC=CC=C2	284.4	1.38	5.02	53.4	0	0	0	CHEMBL1200604	2.21	3	1	2	21	-0.87	0.88	6
Tropisetron	0	0	1	0	5HT3 Antagonist	CN1[C@@H]2CC[C@H]1CC(C2)OC(=O)C3=CNC4=CC=CC=C43	284.4	0.89	9.14	45.3	1	0	0	CHEMBL4303202	2.95	3	1	2	21	0.37	0.86	2
Trospium	1	1	1	1	(Anti)cholinergic	C1CC[N+]2(C1)[C@@H]3CC[C@H]2CC(C3)OC(=O)C(C4=CC=CC=C4)(C5=CC=CC=C5)O	392.5	-0.50	14.00	46.5	1	1	0	CHEMBL1888176	3.77	3	1	2	29	0.25	0.64	4

Substance	MATE1 substrate*	MATE2 substrate*	OCT1 substrate*	OCT2 substrate*	Therapeutic group	SMILES	Molecular weight	logD at pH 7.4	pKa (basic)	TPSA	Net Charge	Quaternary N	Zwitterionic	ChEMBL ID	AlogP	Hydrogen bound	Hydrogen bound	Aromatic rings	Heavy atoms	Natprod likeness score	Rqed (weighted)	Rotable bounds
Tryptamine	1	0	1	1	Biogenic amine	C1=CC=C2C(=C1)C(=CN2)CCN	160.2	-0.79	9.76	41.8	1	0	0	CHEMBL6640	1.67	1	2	2	12	0.05	0.69	2
Tryptophan	0	0	0	0	Amino acid	C1=CC=C2C(=C1)C(=CN2)C[C@@H](C(=O)O)N	204.2	-1.09	9.40	79.1	0	0	1	CHEMBL54976	1.12	2	3	2	15	0.39	0.7	3
Tubocurarine	0	0			Muscle relaxant	CN1CCC2=CC(=C3C=C2[C@@H]1CC4=CC=C(C=C4)OC5=C6[C@@H](CC7=CC(=C(C=C7)O)O3)[N+](CCC6=CC(=C5O)OC)(C)C)OC	609.7	2.24	14.00	80.6	2	1	0	CHEMBL339427	6.7	7	2	4	45	2.26	0.24	2
Tulobuterol	0	0	0	0	Sympathomimetic	CC(C)(C)NCC(C1=CC=CC=C1Cl)O	227.7	0.44	9.55	32.3	1	0	0	CHEMBL1256478	2.76	2	2	1	15	-0.66	0.83	3
Tyramine	1	0	1	1	Sympathomimetic	C1=CC(=CC=C1CCN)O	137.2	-1.17	9.48	46.3	1	0	0	CHEMBL11608	0.89	2	2	1	10	0.76	0.64	2
Umeclidinium	1	0	1	0	(Anti)cholinergic	C1C[N+]2(CCC1(CC2)C(C3=CC=CC=C3)(C4=CC=CC=C4)O)CCOCC5=CC=CC=C5	428.6	0.68	14.00	29.5	1	1	0	CHEMBL1187833	5.14	2	1	3	32	-0.04	0.4	8
Upadacitinib	0	0			JAK inhibitor	CC[C@@H]1CN(C[C@@H]1C2=CN=C3N2C4=C(NC=C4)N=C3)C(=O)NCC(F)(F)F	380.4	0.85	3.41	78.3	0	0	0	CHEMBL3622821	2.91	4	2	3	27	-1.1	0.73	3
Urapidil	0	0	0	0	Antihypertensive	CN1C(=CC(=O)N(C1=O)C)NCCCN2CCN(CC2)C3=CC=CC=C3OC	387.5	0.22	8.31	68.4	1	0	0	CHEMBL1256716	0.72	8	1	2	28	-1.38	0.71	7
Varenicline	0	0	0	0	Others	C1C2CNCC1C3=CC4=NC=CN=C4C=C23	211.3	-1.27	9.73	37.8	1	0	0	CHEMBL1076903	1.8	3	1	2	16	0.25	0.72	0
Vecuronium	1	1	0	0	Muscle relaxant	CC(=O)O[C@H]1C[C@@H]2CC[C@@H]3[C@@H]([C@]2(C[C@@H]1N4CCCCC4)C)CC[C@]5([C@H]3C[C@@H]([C@@H]5OC(=O)C)[N+]6(CCCCC6)C)C	557.8	-1.34	9.65	55.8	2	1	0	CHEMBL1200629	5.97	5	0	0	40	1.54	0.32	4
Venlafaxine	0	0	0	0	Antidepressant	CN(C)CC(C1=CC=C(C=C1)OC)C2(CCCCC2)O	277.4	1.12	9.01	32.7	1	0	0	CHEMBL1201066	3.04	3	1	1	20	0.13	0.9	5
Venlafaxine (R)	0	0			Antidepressant	CN(C)CC(C1=CC=C(C=C1)OC)C2(CCCCC2)O	277.4	1.12	9.01	32.7	1	0	0	CHEMBL1201066	3.04	3	1	1	20	0.13	0.9	5

Substance	MATE1 substrate*	MATE2 substrate*	OCT1 substrate*	OCT2 substrate*	Therapeutic group	SMILES	Molecular weight	logD at pH 7.4	pKa (basic)	TPSA	Net Charge	Quarternary N	Zwitterionic	ChEMBL ID	AlogP	Hydrogen bound	Hydrogen bound	Aromatic rings	Heavy atoms	Natprod likeness score	Rqed (weighted)	Rotable bounds
Venlafaxine (S)	0	0	0		Antidepressant	CN(C)CC(C1=CC=C(C=C1)OC)C2(CCCCC2)O	277.4	1.12	9.01	32.7	1	0	0	CHEMBL1201066	3.04	3	1	1	20	0.13	0.9	5
Veralipride	1	1	1	0	Antipsychotic	COC1=CC(=CC(=C1OC)C(=O)NCC2CCCN2CC=C)S(=O)(=O)N	383.5	-0.46	8.36	119.3	1	0	0	CHEMBL2105581	0.73	6	2	1	26	-1.21	0.64	8
Verapamil	0	0	0	0	Antiarrhythmic	CC(C)C(CCCN(C)CCC1=CC(=C(C=C1)OC)OC)(C#N)C2=CC(=C(C=C2)OC)OC	454.6	2.86	9.61	64.0	1	0	0	CHEMBL6966	5.09	6	0	2	33	-0.07	0.42	13
Verapamil (R)	0	0			Antiarrhythmic	CC(C)C(CCCN(C)CCC1=CC(=C(C=C1)OC)OC)(C#N)C2=CC(=C(C=C2)OC)OC	454.6	2.86	9.61	64.0	1	0	0	CHEMBL570489	5.09	6	0	2	33	-0.07	0.42	13
Verapamil (S)	0	0			Antiarrhythmic	CC(C)C(CCCN(C)CCC1=CC(=C(C=C1)OC)OC)(C#N)C2=CC(=C(C=C2)OC)OC	454.6	2.86	9.61	64.0	1	0	0	CHEMBL6966	5.09	6	0	2	33	-0.07	0.42	13
Vildagliptin	0	0	1	1	Antidiabetic	C1C[C@H](N(C1)C(=O)CNC23CC4CC(C2)CC(C4)(C3)O)C#N	303.4	-1.61	8.78	76.4	1	0	0	CHEMBL142703	1.17	4	2	0	22	-0.5	0.82	3
Viloxazine	0	0	1	0	Antidepressant	CCOC1=CC=CC=C1OCC2CNCCO2	237.3	0.95	7.79	39.7	1	0	0	CHEMBL2106483	1.45	4	1	1	17	-0.37	0.84	5
Xamoterol	1	1	1	0	Sympathomimetic	C1COCCN1C(=O)NCCNCC(COC2=CC=C(C=C2)O)O	339.4	-1.60	8.38	103.3	1	0	0	CHEMBL1329567	-0.24	6	4	1	24	-1.1	0.49	8
Ximelagatran	0	0	0	0	Anticoagulant	CCOC(=O)CN[C@H](C1CCCCC1)C(=O)N2CC[C@H]2C(=O)NCC3=CC=C(C=C3)/C(=N/O)/N	473.6	0.77	6.65	146.4	0	0	0	CHEMBL522038	1.1	7	4	1	34	-0.82	0.13	10
Xylometazoline	0	0	1	1	Sympathomimetic	CC1=CC(=CC(=C1CC2=NCCN2)C)C(C)(C)C	244.4	1.49	10.29	24.4	1	0	0	CHEMBL1256400	3.15	2	1	1	18	-0.34	0.85	2
Yohimbin	0	0	0	0	Herbal	COC(=O)[C@H]1[C@H](CC[C@@H]2[C@@H]1C[C@H]3C4=C(CCN3C2)C5=CC=CC=C5N4)O	354.5	1.76	7.48	65.6	1	0	0	CHEMBL537669	2.65	4	2	2	26	1.17	0.77	1
Zalcitabine	1	0	1	1	Virostatic	C1C[C@@H](O[C@@H]1CO)N2C=CC(=NC2=O)N	211.2	-1.19	3.88	88.2	0	0	0	CHEMBL853	-0.5	6	2	1	15	1.28	0.69	2

Substance	MATE1 substrate*	MATE2 substrate*	OCT1 substrate*	OCT2 substrate*	Therapeutic group	SMILES	Molecular weight	logD at pH 7.4	pKa (basic)	TPSA	Net Charge	Quarternary N	Zwitterionic	ChEMBL ID	AlogP	Hydrogen bound	Hydrogen bound	Aromatic rings	Heavy atoms	Natprod likeness score	Rqed (weighted)	Rotable bounds
Zebularine	0	0			Cytostatic	C1=CN(C(=O)N=C1)[C@H]2[C@@H]([C@@H]([C@H](O2)CO)O)O	228.2	-2.21	0.30	102.6	0	0	0	CHEMBL504567	-2.15	7	3	1	16	0.98	0.53	2
Ziprasidone	0	0	0	0	Antipsychotic	C1CN(CCN1CCC2=C(C=C3C(=C2)CC(=O)N3)Cl)C4=NSC5=CC=CC=C54	412.9	2.83	8.87	76.7	1	0	0	CHEMBL1375743	3.81	5	1	3	28	-1.57	0.71	4
Zolmitriptan	1	1	1	0	Triptan	CN(C)CCC1=CNC2=C1C=C(C=C2)C[C@H]3COC(=O)N3	287.4	-0.11	9.57	57.4	1	0	0	CHEMBL1185	1.92	3	2	2	21	0.14	0.88	5
Zolmitriptan (R)	1	1	1	1	Triptan	CN(C)CCC1=CNC2=C1C=C(C=C2)C[C@H]3COC(=O)N3	287.4	-0.11	9.57	57.4	1	0	0	CHEMBL1185	1.92	3	2	2	21	0.14	0.88	5
Zolmitriptan (S)	1	1	1	0	Triptan	CN(C)CCC1=CNC2=C1C=C(C=C2)C[C@H]3COC(=O)N3	287.4	-0.11	9.57	57.4	1	0	0	CHEMBL1185	1.92	3	2	2	21	0.14	0.88	5
Zotepin	0	0	0		Antipsychotic	CN(C)CCOC1=CC2=CC=CC=C2SC3=C1C=C(C=C3)Cl	331.9	2.98	8.92	37.8	1	0	0	CHEMBL285802	4.88	3	0	2	22	-0.77	0.79	4

* 1 = substrate; 0 = no substrate

Table S5: All 590 substances tested in the present study

Substance	salt	Company	Purchase number
1-(3-Chlorophenyl)piperazine-d8	hydrochloride	Sigma-Aldrich	C-112-1ML
1-(4-Chlorphenyl)biguanidine	hydrate	Sigma-Aldrich	S447366-1G
1,3-Diphenylguanidine		Sigma-Aldrich	D207756-500G
10-OH-Nortriptyline		TRC	H948815-2.5MG
17α-Methyltestosterone		Sigma-Aldrich	69240-5G
1-Methyl-4-phenylpyridinium	dihydrochloride	Sigma-Aldrich	M7068-10MG
1-Methylxanthine		Sigma-Aldrich	M3275
2,5-Dimethoxy-4-iodoamphetamine (DOI)	hydrochloride	Cayman Chemicals	13885
2-Dimethylaminoethanol		Sigma-Aldrich	471453-100ML
2-Methylamino-1-(3,4-methylendioxyphenyl)butan (MBDB)	hydrochloride	Sigma-Aldrich	M-102-1ML
2-Phenylethylamine	hydrochloride	Sigma-Aldrich	P6513-25G
3,4-Methylendioxy-N-methylamphetamine (MDMA)		Sigma-Aldrich	M-013-1ML
3-Iodothyronamine (T1AM)	hydrocholride	Sigma-Aldrich	1924
3-Methoxymorphinan	hydrochloride	Sigma-Aldrich	M187-25MG
3-Methoxy-p-tyramine		TRC	M332320-100MG
4-Amino-1,8-naphthalimide		Sigma-Aldrich	1028336
4-Aminoantipyrine		Sigma-Aldrich	43827-100MG
4-Hydroxydebrisoquine	hemisulfate	Santa Cruz	SC-212710
4-Hydroxymexiletine		TRC	H948020
4-Methoxy-m-tyramine	hydrochloride	Sigma-Aldrich	H3132
4-Methoxyphenethylamine		Sigma-Aldrich	187305-25G
4-Methylaminoantipyrine	hydrochloride	Sigma-Aldrich	92939-25MG
5,6-methylenedioxy-2-aminoindane	hydrochloride	Cerilliant	M-144
5,7-Dihydroxytryptamine		Sigma-Aldrich	SML2058-5MG
5-Aminoindazole		Sigma-Aldrich	A59557-5G
5-Aminolevulinic acid		Sigma-Aldrich	1248020500
5-Methoxytryptamine		Sigma-Aldrich	286583-100MG
6-Hydroxydopamine	hydrobromide	TRC	H941730
6β-Naltrexol	hydrate	Sigma-Aldrich	N9412-10MG
Abacavir	sulfate	Sigma-Aldrich	PHR1256-500MG
Abemaciclib		Biomol GmbH	LKT-A044176.5
Abrocitinib		Biomol GmbH	Cay34869-5
Acebutolol	hydrochloride	Sigma-Aldrich	A3669-1G
Aceclidine	hydrochloride	Sigma-Aldrich	SML0180-10MG
Aclidinium	bromide	Sigma-Aldrich	SML2868-5MG
Aclidinium (S)	bromide	TRC	A190150
Aconitine		Sigma-Aldrich	A8001-25MG
Acyclovir		Adooq Bioscience	A10037-50mg
Agmatine	sulfate	Sigma-Aldrich	A7127-1G
Agomelatin		Sigma-Aldrich	A1362-5MG
Alfuzosine	hydrochloride	Sigma-Aldrich	PHR1638-1G
Aliskiren	hemifurate	Sigma-Aldrich	SML2077-10MG
Alizaprid	hydochloride	Biomol	Cay25645-50
Allopurinol		Sigma-Aldrich	PHR1377-1G
Almotriptan	malate	Sigma-Aldrich	SML1210-10MG
Alpha-methyl norepinephrine (-)		Sigma-Aldrich	SML0675-10MG
alpha-Methyldopa		APExBIO	B1064, 1g
Alprenolol	hydrochloride	Sigma-Aldrich	A0360000

Substance	salt	Company	Purchase number
Amantadine	hydrochloride	Sigma-Aldrich	A1260-5G
Ambroxol	hydrochloride	Sigma-Aldrich	PHR2063-300MG
Amifampridine		Sigma-Aldrich	D-7148-1G
Amikacin	disulfate salt	Sigma-Aldrich	A1774-250MG
Amiloride		Sigma-Aldrich	A7410-1G
Amiodarone	hydrochloride	Sigma-Aldrich	A8423-1G
Amisulpride		Santa Cruz	SC-203510
Amisulpride (R)		TRC	A633255
Amisulpride (S)		TRC	A633260
Amitriptyline	hydrochloride	Sigma-Aldrich	A8404-10G
Amitriptyline-d6	hydrochloride	TRC	A633351-5MG
Amlodipine	besylate	Sigma-Aldrich	PHR1185-1G
Amoxapine		Sigma-Aldrich	A129-100MG
Amoxicillin	hydrate	Biomol	Cay19188-5
Amphetamine	hydrochloride	Cayman Chemicals	14203
APC366	trifluoroacetate	Sigma-Aldrich	SML2450-25MG
Apixaban		Sigma-Aldrich	SML3285-10mg
Apomorphin	hydrochloride hydrate	Biomol	Cay16094-10
Arcaine	sulfate salt	Sigma-Aldrich	A0384-50MG
Aripiprazole		Biomol	Cay19989-10
Articain	hydrochloride	Cayman Chemicals	17883
Aspartame		Merck	47135
Atenolol		Sigma-Aldrich	A7655-1G
Atenolol (R)		Sigma-Aldrich	330884-100MG
Atenolol (S)		Sigma-Aldrich	A143-10MG
Atomoxetine	hydrochloride	Sigma-Aldrich	PHR1679-500MG
Atomoxetine (S)	hydrochloride	TRC	A791405
Atropine		Sigma-Aldrich	A0132-1G
Baclofen		Biomol	Cay18600-1
Bambuterol	hydrochloride	Sigma-Aldrich	B8684-10MG
Baricitinib		Biomol GmbH	Cay16707-25
Befunolol		TRC	B131100
Benzyltriethylammonium	chloride	Sigma-Aldrich	146552-25G
Berberine	chloride	Sigma-Aldrich	B3251-5G
Betahistin	dihydrochloride	Sigma-Aldrich	B4638-5G
Betaine		Sigma-Aldrich	B2629-50G
Betaxolol	hydrochloride	TRC	B327980
Bethanechol		Cayman Chemicals	Cay23830-1G
Bicifadine	hydrochloride	TRC	B382250
Biotin		Sigma-Aldrich	1400-100MG
Biperiden	hydrochloride	Biotrend	BG0108
Bisnorephedrine		Sigma-Aldrich	A72405-10G
Bisnorephedrine (R)		Biotrend	A577265-50MG
Bisoprolol		Sigma-Aldrich	50787
Bitolterol	mesylate	TRC	B591400
Bortezomib		Biomol	Cay10008822-5
Brofaromine		ChemScene	CS-6637
Bromocriptin	mesylate	USP	1076501
Brucine	salt hydrate	Sigma-Aldrich	B0378-25G
Buformin	hydrochloride	Wako	2810052-25G
Bumetanide		Sigma-Aldrich	B3023-250MG
Bunitrolol		TRC	B689440
Bupivacaine	hydrochloride	Sigma-Aldrich	B5274-1G
Bupivacaine (R)	hydrochloride	TRC	B689545
Bupivacaine (S)	hydrochloride	TRC	B689675
Bupropion		Sigma-Aldrich	B102-50MG
Buspirone	hydrochloride	Sigma-Aldrich	B7148-1G
Butylscopolamine	bromide	Sigma-Aldrich	S7882-1G

Substance	salt	Company	Purchase number
Butyrylcarnitine		Sigma-Aldrich	42623-10MG
Cabergolin		Sigma-Aldrich	C0246-10MG
Cadaverine		Sigma-Aldrich	C8561-1G
Caffeine		Sigma-Aldrich	C1778-1VL
Capsaicin		Sigma-Aldrich	M2028-50MG
Captopril		Sigma-Aldrich	C4042-1G
Carnitine-d9	chloride	TRC	TRC-C184102
Carnosine (L)		Sigma-Aldrich	C9625-5G
Carteolol	hydrochloride	Sigma-Aldrich	BP567
Carvedilol (R)		Roche	12040190-200MG
Carvedilol (S)		Roche	12040189-200MG
Cathine	hydrochloride	Sigma-Aldrich	C222
Cathinone (S)	hydrochloride	Sigma-Aldrich	C3196-10MG
Celiprolol	hydrochloride	Sigma-Aldrich	SML2617-5MG
Cetirizin	dihydrochloride	Sigma-Aldrich	PHR1656-1G
Cevimeline	hydrochloride hemihydrate	Sigma-Aldrich	SML0007-5MG
Chlomipramine		BioTrend	BG0150-500MG
Chloramphenicol		AppliChem	A1806,0025-25G
Chlorhexidine		Sigma-Aldrich	PHR1421-1G
Chloroquine	sulfate	Sigma-Aldrich	C1650000
Chlorpheniramine	maleate salt	Sigma-Aldrich	C3025-25G
Chlorpromazine	hydrochloride	Sigma-Aldrich	C8138-5G
Chlorprothixene	hydrochloride	Sigma-Aldrich	C1671-1G
Choline-d4	chloride	Hycultec	HY-B1337S-10MG
Choline-d9	chloride	Biomol	Cay36454-500
Cilastatin		Biomol	Cay23511-10
Cimetidine		Sigma-Aldrich	C4522-5G
Cinchonidin (-)		Sigma-Aldrich	C80407-10G
Ciprofloxacin		Sigma-Aldrich	17850-5G-F
Citalopram	hydrobromide	Cayman Chemicals	23252
Citalopram (R)	oxalate	Santa Cruz	SC-219751
Citalopram (S)	oxalate	Sigma-Aldrich	PHR1733-1G
Citrulline (L)		Sigma-Aldrich	C7629-1G
Clarithromycin		Sigma-Aldrich	PHR1038-500MG
Clemastin	fumarate	Sigma-Aldrich	1134506-250MG
Clenbuterol	hydrochloride	Sigma-Aldrich	C5423
Clidinium	bromide	Sigma-Aldrich	C0414-1G
Clindamycin	hydochloride	Sigma-Aldrich	PHR1159-1G
Clonidine	hydrochloride	Sigma-Aldrich	C7897-100MG
Clozapine		Sigma-Aldrich	C6305-25MG
Cocaine	hydrochloride	Sigma-Aldrich	C5776-1G
Codeine		Sigma-Aldrich	C-015-1ML
Colterol	hydrochloride	TRC	C215850
Coptisine	chloride	MedChem Express	HY-N0736R
Corticosterone		Sigma-Aldrich	27840-100MG
Cotinine (-)		Sigma-Aldrich	28,471-8-250MG
Creatine		Sigma-Aldrich	C0780-50G
Creatinine		Sigma-Aldrich	C4255-10G
Crizotinib		Angene	AG0036BC
Crotaline		Sigma-Aldrich	C2401-500MG
Cycloguanil	hydrochloride	Santa Cruz	C-015-1ML
Cyclopentolate		Sigma-Aldrich	CDS021533-25MG
Cyclophosphamide	monohydrate	Sigma-Aldrich	C0768-1G
Cycloserine (D)		Sigma-Aldrich	C6880-1G
Cycloserine (L)		Sigma-Aldrich	C1159-25MG
Cytarabine		Cayman Chemicals	Cay16069-500
Cytisine		Sigma-Aldrich	712264-100MG

Substance	salt	Company	Purchase number
Dapson		MedChemExpress	HY-B0688
Daunorubicin	hydrochloride	Calbiochem	251800-5MG
Debrisoquine	sulfate	Sigma-Aldrich	D1306-100MG
Decanoyl-L-carnitine-d3	chloride	Biomol	Cay26568-1
Decitabine		Absource	SC-S1200
Dehydrocorydaline		Sigma-Aldrich	SML3501-5MG
Delavirdine	mesylate	Sigma-Aldrich	PZ0180-5MG
Denatonium	benzoate	Sigma-Aldrich	D5765-1G
Denopamine (R)		Sigma-Aldrich	D7815-5MG
Deoxyepinephrine	hydrochloride	TRC	D232920
Deprenyl (R)	hydrochloride	Sigma-Aldrich	M003-250MG
Deprenyl (S)	hydrochloride	TRC	D288595
Desipramine	hydrochloride	BioTrend	BG0162-1G
Desvenlafaxine	hydrochloride	Santa Cruz	SC-255071
Dextromethorphan	hydrobromide	Sigma-Aldrich	D074000
Diclofenac		Sigma-Aldrich	D6899-10G
Diethyltryptamine	hydrochloride	TRC	D445285
Dihydroergotamine	mesylate	MedChemExpress	HY-B0670A
Diltiazem	hydrochloride	Sigma-Aldrich	D2521-1G
Diltiazem		MedChemExpress	HY-B0632
Dimenhydrinat		Sigma-Aldrich	D2396-10G
Dimethylphenylpiperazinium	jodid	Sigma-Aldrich	D5891
Dimethyltryptamine		Sigma-Aldrich	SML0791
Dimetindin		Sigma-Aldrich	D2205000
Diphenhydramine	hydrochloride	Sigma-Aldrich	D3630-5G
Dipyridamole		ChemCruz	sc-200717
Disopyramide	phosphate salt	Sigma-Aldrich	D6035-1G
Dobutamine	hydrochloride	RBI	D-172
Dofetilide		Biomol	Cay15045-10
Domperidone		Sigma-Aldrich	D122-100MG
Donepezil		MedChemExpress	HY-B0034
Dopamine	hydrochloride	Sigma-Aldrich	PHR1090-1G
Doxazosin	mesylate	Sigma-Aldrich	D9815-50MG
Doxepin	hydrochloride	Boehringer	86473342-1G
Doxepine (E)	hydrochloride	TRC	D550005
Doxepine (Z)	hydrochloride	TRC	D550015
Doxycycline	hyclate	Sigma-Aldrich	D891-1G
Doxylamin	succinate	Sigma-Aldrich	D3775-50G
Doxylamine (R)		TRC	D561995
Doxylamine (S)		TRC	D562035
Dropropizine		Sigma-Aldrich	D4285-5G
Dropropizine (D)		TRC	D681500
Dropropizine (L)		Sigma-Aldrich	SML2511-50MG
Duloxetine	hydrochloride	Eli Lilly	032JD0
Duloxetine (R)	hydrochloride	TRC	D720990
Duloxetine (S)	hydrochloride	hellobio	HB1806-10mg
Edrophonium	chloride	Santa Cruz	sc-207610
Efaroxan	hydrochloride	Sigma-Aldrich	E3263-10MG
Eletriptan	hydrobromide	Sigma-Aldrich	PZ0011-5MG
Emtricitabine		Sigma-Aldrich	PHR2120-500MG
Endoxifen	hydrochloride	Sigma-Aldrich	E8284-5MG
Entecavir		Sigma-Aldrich	SML1103-10MG
Ephedrine	hydrochloride	Sigma-Aldrich	285749-5G
Epiberberine	chloride	Biozol	TOR-E578300-10MG
Epinephrine (-)		Sigma-Aldrich	E4250-1G
Epinephrine rac		Sigma-Aldrich	E4642-5G
Ergometrin	maleate	Sigma-Aldrich	BP405

Substance	salt	Company	Purchase number
Ergothioneine (L)		Sigma-Aldrich	E7521-5MG
Esmolol	hydrochloride	Sigma-Aldrich	E8031-10MG
Estrone-3-sulfate	potassium salt	Sigma-Aldrich	E9145-100MG
Ethambutol	dihydrochloride	Sigma-Aldrich	E4630-25G
Etilefrine	hydrochloride	Santa Cruz	sc-294579a
Etomidate		LGC Standards	LGCFOR1264.00
Famotidine		Santa Cruz	sc-205691-500MG
Fampridine (4-aminopyridine)		Sigma-Aldrich	A78403-25G
Fasudil	hydrochloride	Sigma-Aldrich	CDS021620-10MG
Fedratinib		Biomol GmbH	Cay16289-5
Felbamat		MedChemExpress	HY-B0184
Fenfluramine (R)	hydrochloride	TRC	F247595
Fenfluramine (S)	hydrochloride	TRC	F247596
Fenoldopam	mesylate	Sigma-Aldrich	SML0198-10MG
Fenoterol	hydrobromide	Sigma-Aldrich	F1016-1G
Fenpiverinium		Santa Cruz	sc-211504-10MG
Fentanyl		Sigma-Aldrich	F3886-25MG
Fesoterodin	fumarate	Sigma-Aldrich	SML2483
Fexofenadine	hydrochloride	Sigma-Aldrich	Y0000789
Filgotinib		Biomol GmbH	Cay17669-10
Fingolimod		MedChemExpress	HY-11063
Flecainide	acetate	Sigma-Aldrich	PHR2921-500MG
Flunarizine	dihydrochloride	Sigma-Aldrich	F8257-1G
Fluoxetine	hydrochloride	Eli Lilly	4NH27
Fluoxetine (R)	hydrochloride	Sigma-Aldrich	F1678-5mg
Fluoxetine (S)	hydrochloride	Sigma-Aldrich	F1553-5mg
Fluphenazine	dihydrochloride	Sigma-Aldrich	F4765-1G
Fluvoxamine	maleate	Biomol	LKT-F4783
Folic acid		Sigma-Aldrich	F7876-1G
Formoterol	fumarate dihydrochloride	Sigma-Aldrich	F9552-10MG
Formoterol (R) Arformoterol	tartrate	Sigma-Aldrich	SML1667-5MG
Frovatriptan	succinate monohydrate	Sigma-Aldrich	SML1291-10MG
Furosemide		Sigma-Aldrich	F4381-1G
Gabapentin		Sigma-Aldrich	PHR1049-1G
Gabexate	mesylate	Sigma-Aldrich	SML2964-5MG
Galantamine	hydrobromide	SantaCruz	sc-200179A
Gamma Aminobutyric acid		Sigma-Aldrich	A2129-1G
Ganciclovir		Sigma-Aldrich	PHR1593-1G
Gefitinib		MedChemExpress	HY-50895
Gemcitabine	sulfate	MedChemExpress	HY-A0276
Gentian violet		Sigma-Aldrich	G2039-25G
Glutamic acid-d5 (D)		Biomol	Cay34840-10
Glycopyrrolate		Sigma-Aldrich	SML0025-5MG
Granisetron		Santa Cruz	sc-203983-25MG
Guanethidine	monosulfate	Sigma-Aldrich	BP181-100MG
Guanfacin	hydrochloride	Sigma-Aldrich	G1043-10MG
Haloperidol		Sigma-Aldrich	H1512-5G
Halostachine		Sigma-Aldrich	209848-10G
Harmaline		Sigma-Aldrich	51330-1G
Heptylamine		Sigma-Aldrich	126802-25G
Hexanoylcarnitine		Sigma-Aldrich	07439-10MG
Hexylamine		Sigma-Aldrich	219703-5ML
Higenamine	hydrochloride	Sigma-Aldrich	SML2313-5MG
Histamine	dihydrochloride	Sigma-Aldrich	53300-1G
Hordenine		Sigma-Aldrich	04476-100MG
Hydrochlorothiazide		Sigma-Aldrich	H2910-5G

Substance	salt	Company	Purchase number
Hydrocodone	hydrochloride	Sigma-Aldrich	D-019-1ML
Hydromorphone	hydrochloride	Sigma-Aldrich	H5136
Hydroxybenzylamine		Sigma-Aldrich	CDS003156-1G
Hydroxybupropion		Sigma-Aldrich	H-066-1ML
Hydroxychloroquine (R)		TRC	C373710
Hydroxychloroquine (S)		TRC	C373715
Hypaphorine		Biorbyt	orb701587_25mg
Ibogaine	hydrochloride	TRC	TRC-I123000-5mg
Ibutilid	fumarate	MedChemExpress	HY-B0387
Ifosfamide		Sigma-Aldrich	PHR3187-1G
Imatinib		Santa Cruz	sc-202180
Imeglimin	hydrochloride	Sigma-Aldrich	SML2401-5MG
Imipramine	hydrochloride	Sigma-Aldrich	I7379-5G
Indacaterol		Sigma-Aldrich	SML3057-10MG
Ipratropium		Santa Cruz	sc-252911-250MG
Irinotecan		Santa Cruz	sc-269253
Isobutyrylcarnitine-d6	chloride	TRC	I780502-2.5MG
Isoetharine	mesylate salt	Sigma-Aldrich	I3639-1G
Isoniazid		Sigma-Aldrich	I3377-50G
Isoprenaline		Sigma-Aldrich	I5627-5G
Isovalerylcarnitine		Sigma-Aldrich	51371-10MG
Ivabradine	hydochloride	MedChemExpress	HY-B0162A
Ketamine	hydrochloride	Sigma-Aldrich	K2753-1G
Kynurenine		Sigma-Aldrich	61250-250MG
Labetalol	hydrochloride	Sigma-Aldrich	L-1011-5G
Lafutidine		Sigma-Aldrich	SML1611-10MG
Lamivudine		Sigma-Aldrich	PHR1365-1G
Lamotrigine		Sigma-Aldrich	PHR1392-1G
Landiolol	hydrochloride	Sigma-Aldrich	SML1785-5MG
Lenalidomide		Sigma-Aldrich	SML2283-100MG
Leucine-d10 (L)		Biomol	Cay34843-10
Levetiracetam		Sigma-Aldrich	PHR1447-1G
Cetirizin (L)	dihydrochloride	Sigma-Aldrich	L7795-10MG
Levodopa		Sigma-Aldrich	PHR1271-500MG
Levofloxacin		Sigma-Aldrich	PHR1697-1G
Levomepromazin	hydrochloride	Sigma-Aldrich	L0500000
Lidocain		Sigma-Aldrich	L7757-25G
Lysin-d3 (L)	hydrochloride	Biomol	Cay34844-10
Maprotiline	hydrochloride	Sigma-Aldrich	M9651-1G
Maraviroc		Cayman Chemicals	14641
Matrine		Sigma-Aldrich	Y0002013
Mecamylamine		Sigma-Aldrich	M9020-5MG
Medetomidine	hydrochloride	Cayman Chemicals	16454
Memantin	hydrochloride	Sigma-Aldrich	M9292-25MG
Mepenzolate	bromide	Sigma-Aldrich	M5651-5G
Mepivacaine	hydrochloride	Sigma-Aldrich	SML1444
Meptazinol	hydrochloride	Sigma-Aldrich	M2824-10MG
Meropenem	trihydrate	Sigma-Aldrich	M2574
Mescaline	hydrochloride	Sigma-Aldrich	M-047-1ML
Metanephrine	hydrochloride salt	Sigma-Aldrich	SC-208277
Metaproterenol	hemisulfate salt	Sigma-Aldrich	M2398-1G
Metformin	hydrochloride	Sigma-Aldrich	PHR1084-500MG
Methacholine		Sigma-Aldrich	A2251-25G
Methoxytryptophan		Sigma-Aldrich	M4001-100MG
Methylamphetamine	hydrochloride	Sigma-Aldrich	M8750
Methylbutyrylcarnitine		Sigma-Aldrich	50405-10MG
Methylecgonin		Sigma-Aldrich	E-001-1ML
Methylene blue		Sigma-Aldrich	M9140-25G

Substance	salt	Company	Purchase number
Methylenedioxyethylamphetamine	hydrochloride	Cayman Chemicals	14085
Methylergometrine	maleate	Sigma-Aldrich	Y0000776
Methylnaltrexone	bromide	Sigma-Aldrich	SML0277-5MG
Methylnicotinamide	chloride	Sigma-Aldrich	SML0704-10MG
Methylphenidate	hydrochloride	Sigma-Aldrich	M2892-100MG
Methylscopolamine	bromide	Sigma-Aldrich	S8502-1G
Methysergide	maleate	Biomol	Cay27658
Metipranolol		Sigma-Aldrich	BP642-100MG
Metoclopramide	hydrochloride	Sigma-Aldrich	M0763-10G
Metoprolol	tartrate salt	Fluka	80337-100MG
Mexiletine	hydrochloride	Boehringer	KOE 1307CL-100MG
Mexiletine (R)	hydrochloride	TRC	M340790
Mexiletine (S)		TRC	M340795
Miconazole	nitrate salt	Sigma-Aldrich	M3512-1G
Midodrine	hydrochloride	Sigma-Aldrich	M8277-50MG
Milnacipran	hydrochloride	Santa Cruz	sc-204086
Minocyclin	hydrochloride hydrate	Cayman Chemicals	14454-25mg
Minoxidil		Sigma-Aldrich	M4145-25MG
m-Iodobenzylguanidin	hemisulfate salt	Sigma-Aldrich	I9890-5MG
Mirabegron		Sigma-Aldrich	SML2480-5MG
Molsidomine		MedChemExpress	HY-B1069
Moroxydine	hydrochloride	Sigma-Aldrich	278610-1G
Morphine		Sigma-Aldrich	M-030-1ML
Moxifloxacin	hydrochloride	Sigma-Aldrich	PHR1542-1G
Moxonidine		Biomol GmbH	Cay25639-10
m-Tyramine		TRC	T898505-100MG
N-Acetyl-L-cysteine		Sigma-Aldrich	A7250-10G
Nadolol		Sigma-Aldrich	N1892-1G
Nafamostat	mesylate	Selleckchem	S1386
Nalbuphine	hydrochloride hydrate	Sigma-Aldrich	N4396-25MG
Nalmefene	hydrochloride	TOCRIS	4085
Naloxone	hydrochloride dihydrate	Sigma-Aldrich	N7768-250MG
Naltrexon	hydrochloride	Sigma-Aldrich	N3136-100MG
Naratriptan	hydrochloride	Sigma-Aldrich	N1415-10MG
N-Desmethylcitalopram	hydrochloride	Sigma-Aldrich	D-047-1ML
N-Desmethylranitidine		TRC	D292160-1MG
N-Desmethyltramadol	hydrochloride	LGC Standards	LGCFOR000
N-Desmethylvenlafaxine		Wyeth	1497
Nebivolol	hydrochloride	Sigma-Aldrich	N1915-10MG
Nefazodone		Sigma-Aldrich	MJ13754
Neostigmine		Sigma-Aldrich	N2001-1G
N-Ethyl-lidocaine		Sigma-Aldrich	L5783-50MG
Niacin		LKT Laboratories	N3301-10G
Nicotinamide		Sigma-Aldrich	72340-100G
Nimodipine		Sigma-Aldrich	N149-100MG
Nizatidine		Sigma-Aldrich	N7035-5G
N-Methyl-2-phenylethylamine	hydrochloride	LGC Standards	LGCFOR1275.54
N-Methyl-p-Tyramine	hydrochloride	Santa Cruz	sc-391686-1G
N-Methylserotonin		TRC	M326595
N-Methyltryptamine	hydrochloride	TRC	M331920
NN-Dimethylarginine	dihydrochloride	Sigma-Aldrich	D4268-50MG
Norepinephrine (L,-)		Santa Cruz	sc357366-1G
Norepinephrine (S,+)	tartrate hydrate	Sigma-Aldrich	392480-1G
Norepinephrine rac	bitartrate salt	Sigma-Aldrich	A0937-1G
Norfenfluramine (+)		Sigma-Aldrich	N3288-10MG
Normetanephrine		Sigma-Aldrich	N7127-100MG

Substance	salt	Company	Purchase number
Noroxycodone	hydrochloride	Sigma-Aldrich	N-011-1ML
Norphenylephrine	hydrochloride	Sigma-Aldrich	113727-10G
Nortilidine	hydrochloride	Biozol	TOR-N831000
Nortriptyline	hydrochloride	Sigma-Aldrich	N7261-10G
O-Acetyl-L-Carnitine	hydrochloride	Sigma-Aldrich	A6706-1G
Ochratoxin A		Biomol	Cay11439-5
Octopamine	hydrochloride	Sigma-Aldrich	O0250-1G
Ofloxacin		Sigma-Aldrich	O8757-1G
Olanzapin		MedChemExpress	HY-14541
Olodaterol	hydrochloride	TRC	O262000
Ondansetron	hydrochloride	Sigma-Aldrich	PHR1141-1G
Ornithine	hydrochloride	Sigma-Aldrich	O2375-25G
Orphenadrine	citrate salt	Sigma-Aldrich	PHR1777-1G
Oseltamivir	phosphate	Sigma-Aldrich	SML1606-100MG
Oxiconazole	nitrate	Sigma-Aldrich	SML1474-10MG
Oxprenolol	hydrochloride	Tocris	3288-100MG
Oxycodone		Sigma-Aldrich	O-002-1ML
Oxymetazoline	hydrochloride	Sigma-Aldrich	O2378-5G
Oxyphenonium	bromide	Sigma-Aldrich	O5501-1G
Oxytetracyclin		MedChemExpress	HY-B0275
Paliperidone		TRC	P141000
Palmatin	chloride	Sigma-Aldrich	SMB00472-100MG
Palonosetron	hydrochloride	Sigma-Aldrich	SML1195-10MG
Palonosetron (R,R)	hydrochloride	TRC	P165805
Palonosetron (S,S)	hydrochloride	TRC	P165800
Pancuronium		Sigma-Aldrich	P1918-10MG
Pantothenic acid		Cayman Chemicals	Cay17288-25G
Paracetamol		Sigma-Aldrich	A7085-100G
Paraxanthine		Sigma-Aldrich	IMPC-051-03-1ML
Paroxetine	hydroxychloride hemihydrate	SmithKline Beecham	BRL 29060A
Pentamidine	isethionate salt	Sigma-Aldrich	P0547-100MG
Pentorbital		LGC Standards	LGCFOR1357.00
Perphenazine		Sigma-Aldrich	P6402-1G
Pethidine		LGC Standards	LGCAMP0315.11
Phenelzin	sulfate	Sigma-Aldrich	P6777-5g
Phenformin	hydrochloride	Sigma-Aldrich	P7045-10G
Phentermine	hydrochloride	Sigma-Aldrich	SML1968
Phenylalanin (L)		Sigma-Aldrich	P2126-100G
Phenylephrine	hydrochloride	TRC	P320635
Phenylephrine (L)	hydrochloride	Sigma-Aldrich	P6126-5G
Phenylephrine (S)	hydrochloride	TRC	P320630
Phenylethanol		Sigma-Aldrich	P13800
Pilocarpine	nitrate	Sigma-Aldrich	PHR1494-500MG
Pimozide		Sigma-Aldrich	P1793-500MG
Pindolol		Sigma-Aldrich	P0778-250MG
Pinoline		Sigma-Aldrich	291552-100MG
Pioglitazone	hydrochloride	Sigma-Aldrich	E6910-10MG
Pipamperone	dihydrochloride	Sigma-Aldrich	P3246-100MG
Piperacillin		Biomol GmbH	Cay20766-500
Piperazine		Sigma-Aldrich	P45907-5G
Pirbuterol	acetate	Sigma-Aldrich	32142-10MG
Pirenzepine	dihydrochloride monohydrate	Sigma-Aldrich	Y0000038
Piritramide		TRC	P508800
p-Methoxyamphetamin	hydrochloride	Cayman Chemicals	12041
p-Methoxymetamphetamine	hydrochloride	Cayman Chemicals	11562
Pomalidomide		Biomol	CAY19877-5

Substance	salt	Company	Purchase number
p-Phenylenediamine		Sigma-Aldrich	P6001-50G
Practolol		Sigma-Aldrich	SML1462-5MG
Prazosine	hydrochloride	Sigma-Aldrich	P7791-50MG
Prenalterol (S)		Santa Cruz	sc-280023A-5MG
Primaquine	phosphate	Sigma-Aldrich	PHR3108-500MG
Procainamide	hydrochloride	Sigma-Aldrich	P9391-25G
Proguanil	hydrochloride	Sigma-Aldrich	G7048-10MG
Propafenone	hydrochloride	Sigma-Aldrich	P4670-5G
Propafenone (R)		TRC	P757495
Propafenone (S)		TRC	P757496
Propanthelin	bromide	Merck	P8891-5G
Propionylcarnitine		Sigma-Aldrich	42602-10MG
Propiverin	hydrochloride	Sigma-Aldrich	SML0602-5MG
Propofol		LKT Laboratories Inc.	P6870
Propranolol	hydrochloride	Sigma-Aldrich	P0884-1G
Propranolol (R)	hydrochloride	Sigma-Aldrich	P0689-100MG
Propranolol (S)	hydrochloride	Sigma-Aldrich	P8688-100MG
Prostaglandin E2		Biomol	Cay14010-10
Prostaglandin F2alpha		Biomol	Cay16010-5
Prucalopride		MedChemExpress	HY-14151
Pseudoephedrin	hydochloride	Sigma-Aldrich	P3850000-50MG
Putrescine	dihydrochloride	Sigma-Aldrich	P5780-5G
Pyrazinamide		Sigma-Aldrich	PHR1576-500MG
Pyridoxal	hydrochloride	Sigma-Aldrich	P9130-500MG
Pyridoxamine	dihydrochloride	Sigma-Aldrich	P9380-1G
Pyridoxin		Sigma-Aldrich	P5669-5G
Pyrilamine	maleate salt	Sigma-Aldrich	P5514-5G
Pyrimethamine		Fluka	46706-250MG
Pyrithiamine	hydrobromide	Sigma-Aldrich	SC-236525A
Quetiapin	fumarate	Sigma-Aldrich	PHR1856-1G
Quinidine		Fluka	22600
Quinine		Sigma-Aldrich	145904-10G
Ractopamine	hydrochloride	Sigma-Aldrich	34198-100MG
Raloxifen	hydochloride	Biomol	Cay10011620-50
Ranitidine	hydrochloride	Sigma-Aldrich	R101-1G
Ranolazin		MedChemExpress	HY-B0280
Rasagiline	mesylate	Sigma-Aldrich	SML0124-10MG
Rasagiline (S)	mesylate	TRC	R126005
Reboxetine	methansulfonate	Upjohn	PNU0155950E
Remoxipride	hydrochloride	ApexBio	B6051
Reproterol		Santa Cruz	sc-477307
Reserpine		Sigma-Aldrich	8350-1G
Revefenacin, CAS		Biozol	SEL-S5258-5MG
Riboflavin		Merck	47861
Rifampicin		Sigma-Aldrich	R3501
Risperidon		Sigma-Aldrich	R3030-10MG
Ritodrine	hydrochloride	Sigma-Aldrich	R0758-250MG
Rivastigmin	tartrate	Sigma-Aldrich	SML0881-50MG
Rizatriptan	benzoate salt	Sigma-Aldrich	SML0247-10MG
Rocuronium	bromide	Sigma-Aldrich	R5155-50MG
Ropinirol	hydrochloride	Sigma-Aldrich	R2530-25MG
Ropivacain	hydrochloride monohydrate	Sigma-Aldrich	PHR2303-500MG
Rucaparib	camsylate	Sigma-Aldrich	PZ0036-5MG
Ruxolitinib		Biomol GmbH	Cay11609-10
Salbutamol		Sigma-Aldrich	S8260-25MG
Salbutamol (R)	hydrochloride	TRC	A514485
Salbutamol (S)	hydrochloride	TRC	A514490

Substance	salt	Company	Purchase number
Salmeterol	xinofate	Sigma-Aldrich	Y0000422
Salsolidine		TRC	S100005
Salsolinol	hydrobromide	Sigma-Aldrich	SML0398-10MG
Sarcosine		Sigma-Aldrich	131776-100G
Scopolamine	hydrochloride	Santa Cruz	sc-253544-1G
Selegilin	hydrochloride	Sigma-Aldrich	PHR3134-500MG
Sematilide	monohydrochloride monohydrate	Sigma-Aldrich	S0323-10MG
Sepantronium	bromide	Biozol	AMO-M2342-10MG
Serotonin	hydrochloride	Sigma-Aldrich	H9523-100MG
Sertraline	hydrochloride	Pfizer	CP05197401
Sitagliptin		MedChemExpress	HY-13749/CS-2914
Solifenacin	succinate	Sigma-Aldrich	SML2141-25MG
Sotalol	hydrochloride	Sigma-Aldrich	sc-203699-10MG
Sparteine (-)		Sigma-Aldrich	S2251-10G
Sparteine (+)		Sigma-Aldrich	92052-1G
Spermidine		Sigma-Aldrich	S2626-1G
Spermine		Sigma-Aldrich	55513-100MG
Stachydrine	chloride	Sigma-Aldrich	89799-25MG
Strychnine		Sigma-Aldrich	S0532-5G
Succinylcarnitine	lithium salt	Sigma-Aldrich	O4609-10MG
Sufentanil		LGC Standards	CAY-15917-1MG
Sulpirid (S)		Sigma-Aldrich	S7771-5G
Sulpiride		Sigma-Aldrich	S8010-25G
Sumatriptan	succinate	Sigma-Aldrich	S1198-50MG
Synephrine		Sigma-Aldrich	S0752-5G
Talinolol		Santa Cruz	sc-212743-5MG
Tamoxifen		Sigma-Aldrich	T5648-1G
Tamsulosin		Sigma-Aldrich	Y0000653
Tapentadol	hydrochloride	Sigma-Aldrich	T-058-1ML
Taurine		Sigma-Aldrich	T0625-10G
Temozolomide		Cayman Chemicals	14163-25mg
Terazosin	hydrochloride	Santa Cruz	sc-204337-50MG
Terbutaline	hemisulfate salt	Sigma-Aldrich	T2528-1G
Terfenadine		Cayman Chemicals	20305
Tetracaine	hydrochloride	Sigma-Aldrich	T7508-5G
Tetracycline	hydrochloride	Cayman Chemicals	14328-10g
Tetraethylammonium	chloride	Sigma-Aldrich	T2265-25G
Thalidomide		Cayman Chemicals	Cay14610-100MG
Thiamine	hydrochloride	Sigma-Aldrich	T1270-25G
Thiamine monophosphate	chloride dihydrate	Sigma-Aldrich	D8637-5G
Thyrotropin-Releasing-Hormon		Sigma-Aldrich	P1319-50MG
Tianeptin	sodium salt	Chemodex	T0456
Tiglycarnitine	hydrochloride	Santa Cruz	sc-475649
Timolol	maleate salt	Sigma-Aldrich	T6394-250MG
Timolol (R)		Sigma-Aldrich	Y0000644
Tiotropium	bromide monohydrate	Sigma-Aldrich	SML2008-10MG
Tofacitinib		Biomol	Cay11598-10
Tolterodine (R)	tartrate	Sigma-Aldrich	PHR1891
Tolterodine (S)	tartrate	TRC	T535796
Topotecan	hydrochloride hydrate	Sigma-Aldrich	T2705-10MG
Tramadol	hydrochloride	LGC Standards	LGCAMP0007.00-01
Tranylcypromine	hydrochloride	Merck	616431-500MG
Triethylentetramine		Sigma-Aldrich	90460-10ML
Trimethoprim		Sigma-Aldrich	PHR1056-1G
Trimipramine	maleate	Sigma-Aldrich	T3146

Substance	salt	Company	Purchase number
Tropicamide		Sigma-Aldrich	PHR3152-500MG
Tropisetron	hydrochloride	Santa Cruz	sc-204930-10MG
Trospium	chloride	Sigma-Aldrich	PHR3378-200MG
Tryptamine	hydrochloride	Sigma-Aldrich	246557-5G
Tryptophan-d5 (L)		Biomol	Cay34829-10
Tubocurarin	hydochloride pentahydrate	Sigma-Aldrich	T2379-100MG
Tulobuterol		Santa Cruz	sc-213131-100MG
Tyramine		Fluka	80345-100MG
Umeclidinium	bromide	TRC	U710000
Upadacitinib		Biomol GmbH	Cay29706-5
Urapidil	hydrochloride	Sigma-Aldrich	U100-100MG
Varenicline	tartrate	Sigma-Aldrich	PZ004-5MG
Vecuronium	bromide	Sigma-Aldrich	76904-10MG
Venlafaxine	hydrochloride	Wyeth	1493-200MG
Venlafaxine (R)		TRC	V120003
Venlafaxine (S)		TRC	V120008
Veralipride		Sigma-Aldrich	T7632-5MG
Verapamil	hydrochloride	Sigma-Aldrich	V4629-1G
Verapamil (R)	hydrochloride	Sigma-Aldrich	V106-5MG
Verapamil (S)	hydrochloride	Sigma-Aldrich	V105-5MG
Vildagliptin		Sigma-Aldrich	SML2302-50MG
Viloxazine	hydrochloride	Sigma-Aldrich	SML3133-10MG
Xamoterol	hemifumarate	TRC	X499808
Ximelagatran		Sigma-Aldrich	SML1768-5MG
Xylometazolin	hydrochloride	Sigma-Aldrich	X6000-5G
Yohimbin	hydrochloride	Sigma-Aldrich	Y3125-1G
Zalcitabine		ChemScene	CS-1110
Zebularine		Absource	SC-S7113
Ziprasidone		Ascent Scientific	ASC-394
Zolmitriptan		Sigma-Aldrich	SML0248-10MG
Zolmitriptan (R)		Sigma-Aldrich	Y0001986
Zolmitriptan (S)		Sigma-Aldrich	SML0248-10MG
Zotepin		Sigma-Aldrich	Z0877-10MG

Table S6: HPLC-MS/MS analysis parameters of the substances included in the study

	Substance	Mass Q1 [Da]	Mass Q3 [Da]	DP [V]	CE [V]	CXP [V]	Internal standard
	**3% organic additive** (96.9% H2O, 0.1% formic acid, 2.6% acetonitrile, 0.4% methanol)						
3	2-Dimethylaminoethanol	90.069	72	41	17	12	Buformin
			(45.1)		(26	(8)	
3	3-Methoxy-p-tyramine	168.1	150.9	41	13	10	Buformin
			(119.0)		(25)	(8)	
3	4-Methoxy-m-tyramine	168.12	151.0	41	13	10	Buformin
			(91.0)		(33)	(16)	
3	4-Methylaminoantipyrine	218.194	159.1	60	21	10	Buformin
			(99.0)		(33)	(18)	
3	5-Aminoindazole	134.077	107.1	36	25	6	Buformin
			(80.0)		(35)	(14)	
3	5-Aminolevulinic acid	131.98	114.1	76	13	6	Buformin
			(86.1)		(19)	(16)	
3	5,7-Dihydroxytryptamine	193.0	176.2	51	13	10	Buformin
			(149.2)		(19)		
3	6-Hydroxydopamine	170.1	153.1	46	13	14	Buformin
			(76.9)		(49)	(14)	
3	Aceclidine	170.1	110	90	26	6	Buformin
			(128.1)		(30)	(12)	
3	Agmatine	131.96	73.0	64	25	12	Buformin
			(114.0)		(16)	(8)	
3	Alpha-methyl norepinephrine (-)	184	166	41	13	10	Buformin
			(103.0)		(36)	(6)	
3	alpha-Methyldopa	212.0	195.0	41	9	13	Buformin
			(177.0)		(10)	(15)	
3	Amifampridine	110	92.9	76	30	17	Buformin
			(82.9)		(27)	(15)	
3	Arcaine	173.164	114.1	65	19	6	Buformin
			(72.2)		(31)	(12)	
3	Betahistin	137.096	94.0	46	19	18	Buformin
			(106.1)		(17)		
3	Betaine	118	58	31	39	10	Buformin
			(59.9)		(23)		
3	Bisnorephedrine	137.89	119.91	29	12	8	Buformin
			(76.86)		(40)	(14)	
3	Buformin	157.91	60.9	36	35	10	Choline-d9
			(47.0)		(66)	(8)	
3	Cadaverine	103.16	86.0	41	14	6	Buformin
			(69.0)		(23)	(12)	
3	Choline-d4	108.156	60.1	81	25	10	
			(49.2)		(29)	(8)	

	Substance	Mass Q1 [Da]	Mass Q3 [Da]	DP [V]	CE [V]	CXP [V]	Internal standard
3	Choline-d9	113.1	69.1	66	27	12	Buformin
			(66.1)		(44)	(12)	
3	Citrulline (L)	177.08	71	31	13	12	Buformin
3	Cotinine (-)	177.035	80.1	56	33	14	Buformin
			(98.0)		(30)	(18)	
3	Creatinine	114.12	44.0	56	28	7	Buformin
			(86.0)		(17)	(11)	
3	Cycloserine (D), (L)	103.064	75	51	11	14	Buformin
			(58)		(15)	(10)	
3	Cytarabine	244.125	112.0	50	17	6	Buformin
			(95.1)		(58)	(18)	
3	Cytisine	191.086	148.1	90	29	10	Buformin
			(44.2)		(47)	(8)	
3	Decitabine	229.116	113.0	40	11	6	Buformin
			(85.9)		(39)	(16)	
3	Deoxyepinephrine	168.109	137.1	56	17	8	Buformin
			(91.1)		(35)	(16)	
3	Dopamine	154.1	137.2	36	15	10	Buformin/Choline-d4
3	Edrophonium	167.2	137	76	37	8	Buformin
			(139.0)		(23)	(9)	
3	Epinephrine	184.1	135	36	15	8	Buformin/Choline-d4
			(166.1)		(13)	(10)	
3	Ergothioneine (L)	230.159	127	50	25	8	Buformin
			(186.2)		(17)	(12)	
3	Ethambutol	205.2	116.1	66	21	6	Buformin
			(55.1)		(45)	(10)	
3	Etilefrine	182.124	164.0	51	17	10	Buformin
			(91.0)		(37)	(6)	
3	Fampridine (4-aminopyridine)	94.967	78.4	86	39	14	Buformin
			(68.1)		(30)	(12)	
3	Gamma Aminobutyric acid	104.2	87	34	15	11	Buformin
			(69.0)		(22)	(12)	
3	Gemcitabine	264.093	112.0	50	24	6	Buformin
			(95.1)		(59)	(18)	
3	Glutamic acid-d5 (D)	152.8	135.1	36	13	8	Buformin
			(88.1)		(23)	(16)	
3	Guanethidine	199.144	140.1	60	25	8	Buformin
			(86.0)		(23)	(16)	
3	Halostachine	152.1	134.0	41	13	8	Buformin
			(119.0)		(27)		
3	Hordenine	166.14	121.0	51	21	8	Buformin
			(77.0)		(47)	(10)	
3	Imeglimin	156.178	113.1	71	23	8	Buformin/Choline-d4
			(71.1)		(39)	(14)	
3	Isoniazid	138.079	121.0	61	19	8	Buformin

	Substance	Mass Q1 [Da]	Mass Q3 [Da]	DP [V]	CE [V]	CXP [V]	Internal standard
			(79.0)		(40)	(6)	
3	Isoprenaline	212.2	194.0	41	15	12	Buformin
			(151.9)		(23)	(10)	
3	Lamivudine	230	111.9	46	17	6	Buformin/Choline-d4
			(95.0)		(53)	(18)	
3	Leucine-d10 (L)	142.1	96.2	50	15	18	Buformin
			(50.2)		(36)	(8)	
3	Levodopa	197.9	152.1	41	19	15	Buformin
			(180.1)		(13)	(12)	
3	Lysin-d3 (L)	150.12	87.20	31	22	16	Buformin
			(133.1)		(8)	(8)	
3	m-Tyramine	138.1	121.0	31	15	8	Buformin
			(77.0)		(37)	(14)	
3	Matrine	249.245	148.2	106	47	8	Buformin
			(150.2)		(45)	(8)	
3	Metanephrine	198.1	180.0	41	13	12	Buformin
			(165.0)		(25)	(10)	
3	Metaproterenol	212.13	152.0	56	23	10	Buformin
			(107.0)		(39)	(8)	
3	Metformin	130	71	40	35	10	Buformin
3	Methacholine	160.107	101	50	17	18	Buformin/Choline-d4
			(99.9)		(15)	(6)	
3	Methylecgonin	200.249	182.1	46	25	12	Buformin
			(82.0)		(35)	(6)	
3	Methylnicotinamide	137	94.1	65	29	5	Buformin
			(78.0)		(35)	(14)	
3	Moroxydine	172.151	60.2	81	27	10	Buformin
			(69.0)		(41)	(12)	
3	N-Methyl-p-Tyramine	152.1	121.2	51	17	8	Buformin
			(103.1)		(31)	(6)	
3	Niacin	124.082	80.2	26	29	14	Buformin
			(78.0)		(32)		
3	Nizatidine	332.2	155	71	27	10	Buformin
			(131.0)		(35)	(10)	
3	NN-Dimethylarginine	203.25	70.0	66	38	12	Buformin
			(88.0)		(23)	(11)	
3	Norepinephrine	170.2	152.0	36	10	10	Buformin/Choline-d4
			(107.0)		(28)	(20)	
3	Normetanephrine	184.1	166.0	36	9	10	Buformin
			(134.0)		(25)	(8)	
3	Norphenylephrine	154.18	136.0	39	11	8	Buformin
			(91.1)		(29)	(16)	
3	Ornithine	133.1	70.0	41	23	12	Buformin
			(116.0)		(13)	(8)	
3	p-Phenylenediamine	108.053	80.0	86	37	4	Buformin

	Substance	Mass Q1 [Da]	Mass Q3 [Da]	DP [V]	CE [V]	CXP [V]	Internal standard
			(53.2)		(51)	(10)	
3	Phenylephrine	168.21	91	41	30	11	Buformin
			(77)		(56)	(4)	
3	Phenylethanol	138.18	103.0	39	28	8	Buformin
3	Pilocarpine	209.115	95.1	96	46	18	Buformin
			(68.0)		(64)	(12)	
3	Piperazine	86.99	42.9	86	26	7	Buformin
			(44.0)			(8)	
3	Procainamide	236.1	163.3	55	22	10	Buformin
			(120.1)		(40)	(7)	
3	Propofol	179.179	101	36	9	6	Buformin
			(79)		(11)		
3	Putrescine	89.12	72.0	41	13	12	Buformin
			(61.0)		(11)		
3	Pyridoxal	168.174	150.0	51	15	10	Buformin
			(94.1)		(32)	(18)	
3	Pyridoxamine	169.152	152.1	55	17	10	Buformin/Choline-d4
			(134.1)		(29)	(8)	
3	Pyridoxin	170.151	152.0	60	19	10	Buformin/Choline-d4
			(134.1)		(28)	(8)	
3	Salsolinol	180.08	163.1	81	19	10	Buformin
			(145.2)		(25)		
3	Sarcosine	90.085	44.0	41	20	8	Buformin
3	Serotonin	177.1	160.1	53	17	10	Buformin
			(132.0)		(30)		
3	Sparteine	235.151	98.2	100	49	8	Buformin
			(233.1)		(36)	(16)	
3	Spermidine	146.21	129	34	9	9	Buformin
			(72.0)		(23)	(12)	
3	Spermine	204.08	186.0	41	9	13	Buformin
			(169.0)		(13)	(8)	
3	Stachydrine	144.006	58.2	70	37	10	Buformin
			(84.0)		(30)	(16)	
3	Synephrine	167.691	149.9	36	35	12	Buformin
			(135.0)			(8)	
3	Thiamine	265.1	122.1	50	25	10	Buformin
			(143.8)		(19)	(10)	
3	Thiamine monophosphate	345.1	122.1	40	35	10	Buformin
3	Thyrotropin-Releasing-Hormon	363.12	249.1	80	25	16	Buformin
			(110.2)		(55)	(6)	
3	Triethylentetramine	147.118	87.2	43	17	16	Buformin
			(44.1)		(36)	(8)	
3	Tyramine	138.1	121.1	43	14	12	Buformin
3	Zebularine	229.123	97.0	41	12	18	Buformin
			(78.9)		(52)	(14)	

	Substance	Mass Q1 [Da]	Mass Q3 [Da]	DP [V]	CE [V]	CXP [V]	Internal standard
	**8% organic additive** (91.9% H2O, 0.1% formic acid, 6.9% acetonitrile, 1.1% methanol)						
8	2-Phenylethylamine	122.13	77.0	44	41	6	Ranitidine-d6
			(79.0)		(31)		
8	4-Hydroxydebrisoquine	192.2	174	60	20	15	Ranitidine-d6
			(132.1)		(29)	(15)	
8	Acyclovir	225.98	151.9	46	17	10	Ranitidine-d6
			(134.93)		(40)	(8)	
8	Amantadine	152.15	77	71	57	5	Ranitidine-d6
8	Amiloride						Ranitidine-d6
8	Benzyltriethylammonium	193.32	92.1	56	28	17	Ranitidine-d6
			(101.0)		(23)	(18)	
8	Bethanechol	162.082	103.2	40	17	18	Ranitidine-d6
			(102.1)				
8	Butyrylcarnitine	232.2	85.0	65	29	6	Ranitidine-d6
			(173.1)		(19)	(11)	
8	Carnitine-d9	171.3	85	81	29	6	Ranitidine-d6
			(129)		(9)	(10)	
8	Carnosine (L)	227.08	110.0	61	31	7	Ranitidine-d6
			(156.0)		(21)	(12)	
8	Cathinone (S)	150.07	117.0	41	29	6	Ranitidine-d6
			(105.0)		(25)	(20)	
8	Cimetidine	253.129	159	61	21	10	Ranitidine-d6
			(95.2)		(34)	(18)	
8	Codeine	303.3	215.1	105	35	15	Ranitidine-d6
			(165.1)		(58)	(15)	
8	Colterol	226.148	152	70	21	12	Ranitidine-d6
			(208.1)		(15)	(14)	
8	Creatine	132.03	90.0	44	17	11	Ranitidine-d6
			(44.0)		(31)	(8)	
8	Debrisoquine	176.2	159.1	80	25	15	Ranitidine-d6
			(134.2)		(26)	(15)	
8	Dimethylphenylpiperazinium	192.29	72.06	96	34	13	Ranitidine-d6
			(58.0)		(54)	(10)	
8	Emtricitabine	247.95	129.97	44	15	8	Ranitidine-d6
8	Entecavir	278.07	152.03	69	25	10	Ranitidine-d6
			(135.04)		(50)	(9)	
8	Ephedrine (+)	166.02	148.1	41	17	9	Ranitidine-d6
			(133)		(27)	(8)	
8	Famotidine	338.5	189	54	27	12	Ranitidine-d6
			(155.0)		(43)	(10)	
8	Frovatriptan	244.3	213	56	19	14	Ranitidine-d6
			(170.1)		(34)	(10)	
8	Heptylamine	116.09	57	51	18	10	Ranitidine-d6
			(70)		(21)	(12)	
8	Histamine	112.09	95.1	31	19	18	Ranitidine-d6

	Substance	Mass Q1 [Da]	Mass Q3 [Da]	DP [V]	CE [V]	CXP [V]	Internal standard
			(67.9)		(31)	(12)	
8	Homoarginin	189.121	84.2	70	31	14	Ranitidine-d6
			(144.2)		(22)	(12)	
8	Hydrocodone	300.472	283.1	56	15	26	Ranitidine-d6
			(133.1)		(21)	(28)	
8	Hydromorphone	286.2	185.2	110	43	5	Ranitidine-d6
			(157.1)		(55)	(10)	
8	Hydroxybenzylamine	124.11	107	29	9	6	Ranitidine-d6
			(77.0)		(35)	(14)	
8	Isobutyrylcarnitine-d6	238.1	85.0	65	25	15	Ranitidine-d6
8	Isoetharine	240.124	222.1	61	19	14	Ranitidine-d6
			(123.1)		(39)	(22)	
8	Kynurenine	209.22	94	46	19	17	Ranitidine-d6
			(146.0)		(23)	(11)	
8	Morphine	286.2	201.1	110	36	15	Ranitidine-d6
			(165.1)		(54)	(15)	
8	N-Acetyl-L-cysteine	164.098	147.2	36	9	10	Ranitidine-d6
			(129.1)		(13)	(8)	
8	N-Methylserotonin	191.101	160.0	60	17	10	Ranitidine-d6
			(148.0)				
8	O-Acetyl-L-Carnitine	204.1	85	60	27	6	Ranitidine-d6
			(134.93)				
8	Octopamine	154.1	136.0	36	11	8	Ranitidine-d6
			(91.0)		(29)	(16)	
8	Olanzapin	313.262	256.1	85	35	16	Ranitidine-d6
			(84.2)		(55)	(12)	
8	Phenelzin	137.06	105.1	26	17	18	Ranitidine-d6
			(79.0)		(35)	(14)	
8	Phenylalanin (L)	166.107	120.0	41	17	8	Ranitidine-d6
8	Pirbuterol	241.3	167.2	65	24	15	Ranitidine-d6
			(149.1)		(30)	(15)	
8	Prenalterol (S)	226.28	149.1	81	23	10	Ranitidine-d6
			(56.0)		(39)	(10)	
8	Propionylcarnitine	218.2	85.0	60	28	6	Ranitidine-d6
			(159.0)		(19)	(10)	
8	Pseudoephedrine	166.17	148.0	50	15	10	Ranitidine-d6
			(133.2)		(29)	(8)	
8	Pyrazinamide	124.055	107.1	56	15	20	Ranitidine-d6
			(80.9)		(23)	(14)	
8	Ranitidine	315.3	176	65	24	11	Ranitidine-d6
			(130.1)		(34)	(8)	
8	Ranitidine-d6	321.2	176	65	25	15	
			(130.1)		(35)	(15)	
8	Rizatriptan	270	158	55	28	11	Ranitidine-d6
			(58.0)		(42)	(11)	

	Substance	Mass Q1 [Da]	Mass Q3 [Da]	DP [V]	CE [V]	CXP [V]	Internal standard
8	Salbutamol	240.2	148.2	60	24	15	Ranitidine-d6
			(222.2)				
8	Salsolidine	208.151	191.2	66	21	12	Ranitidine-d6
			(160.1)		(29)	(12)	
8	Sematilide	314.42	240.9	79	27	16	Ranitidine-d6
			(162.1)		(39)	(10)	
8	Sotalol	273.37	255.1	61	17	16	Ranitidine-d6
			(133.1)		(37)	(8)	
8	Sulpiride	342.2	112.1	70	36	15	Ranitidine-d6
			(214.2)		(42)	(15)	
8	Sumatriptan	296.2	58.2	50	30	12	Ranitidine-d6
			(251.2)		(24)		
8	Taurine	126.0	108.0	65	20	15	Ranitidine-d6
8	Terbutaline	226.2	152.1	60	23	10	Ranitidine-d6
			(107.0)		(40)		
8	Tetraethylammonium	130.247	86.1	61	30	16	Ranitidine-d6
			(58.2)		(45)	(10)	
8	Tiglycarnitine	244.13	85.0	80	20	15	Ranitidine-d6
8	Trimethoprim	291	230.19	94	33	15	Ranitidine-d6
			(123.01)		(33)	(8)	
8	Tryptamine	161.06	144.0	41	13	10	Ranitidine-d6
			(117.0)		(33)	(8)	
8	Veralipride	384.26	124.2	90	39	8	Ranitidine-d6
			(244.1)		(42)	(14)	
8	Vildagliptin	304.286	154.1	90	23	10	Ranitidine-d6
			(97.1)		(43)	(8)	
8	Zalcitabine	212.126	112	36	13	6	Ranitidine-d6
			(95.0)		(47)	(18)	
8	Zolmitriptan	310.3	109.1	70	40	12	Ranitidine-d6
			(141)		(26)		
	**20% organic additive** (79.9% H2O, 0.1% formic acid, 17.2% acetonitrile, 2.8% methanol)						
20	1-(4-Chlorphenyl)biguanidine	212.09	153.02	75	35	10	Fenoterol-d6
			(111.06)		(52)	(10)	
20	1-Methyl-4-phenylpyridinium	170.016	128.1	100	42	8	Fenoterol-d6
			(154)		(43)	(10)	
20	1-Methylxanthine	167.167	110.0	70	27	8	Fenoterol-d6
20	2-Methylamino-1-(3,4-methylendioxyphenyl)butan	208.06	135.03	51	24	8	Fenoterol-d6
			(177.07)		(15)	(11)	
20	3,4-Methylendioxy-N-methylamphetamine	193.9	163	41	17	10	Fenoterol-d6
			(104.9)		(33)	(6)	
20	4-Aminoantipyrine	204.142	159.2	65	19	10	Fenoterol-d6
			(83.0)			(14)	
20	4-Hydroxymexiletine	196.095	58.2	51	23	10	Fenoterol-d6
			(137.1)		(25)	(8)	
20	4-Methoxyphenethylamine	152.1	135	36	11	8	Fenoterol-d6

	Substance	Mass Q1 [Da]	Mass Q3 [Da]	DP [V]	CE [V]	CXP [V]	Internal standard
			(105.1)		(27)	(6)	
20	5-Methoxytryptamine	191.035	174.1	46	13	10	Fenoterol-d6
			(159.1)		(31)		
20	5,6-methylenedioxy-2-aminoindane	177.97	161	43	17	10	Fenoterol-d6
			(131)		(27)	(16)	
20	6β-Naltrexol	344.208	326.2	58	29	22	Fenoterol-d6
			(55.2)		(68)	(10)	
20	Abacavir	287.123	191.3	76	28	14	Fenoterol-d6
			(79.0)		(45)	(14)	
20	Acebutolol	337.243	116.0	91	31	8	Fenoterol-d6
			(98.066)		(29)		
20	Alizapride	316.296	124.2	86	35	8	Fenoterol-d6
			(148.1)		(37)		
20	Allopurinol	136.908	110.2	76	29	20	Fenoterol-d6
			(54.2)		(45)	(8)	
20	Amisulpride	370.2	242.1	90	38	15	Fenoterol-d6
			(195.8)		(52)	(15)	
20	Amoxicillin	366.133	349.3	56	11	10	Fenoterol-d6
			(114.0)		(28)	(6)	
20	Amphetamine	136	91	41	21	16	Fenoterol-d6
			(119)		(13)	(14)	
20	Apomorphine	268.234	237.2	86	23	14	Fenoterol-d6
			(191.4)		(41)		
20	Articaine	285.203	86.2	63	21	16	Fenoterol-d6
			(253.1)		(17)	(24)	
20	Atenolol	267.2	145.2	130	38	10	Fenoterol-d6
20	Atracurium	358.2	206.1	66	28	14	Fenoterol-d6
			(151.1)		(44)		
20	Atropine	290.2	142.2	100	45	12	Fenoterol-d6
			(124.2)		(33)	(12)	
20	Baclofen	214.118	151.0	50	25	10	Fenoterol-d6
			(116.1)		(45)	(22)	
20	Brucine	395.249	324.1	135	42	10	Fenoterol-d6
			(244.0)		(49)	(16)	
20	Bupropion	240.08	183.9	55	17	12	Fenoterol-d6
			(139.0)		(35)	(12)	
20	Butylscopolamine	360.2	121.2	100	40	15	Fenoterol-d6
20	Cabergolin	452.3	381.4	95	25	12	Fenoterol-d6
			(336.5)		(33)	(10)	
20	Caffeine	195.189	138.1	70	27	8	Fenoterol-d6
			(110.0)		(32)		
20	Carteolol	293.219	237.3	63	21	16	Fenoterol-d6
			(202.1)		(30)	(12)	
20	Cathine	152.09	91	39	39	16	Fenoterol-d6
			(117)		(23)	(22)	

	Substance	Mass Q1 [Da]	Mass Q3 [Da]	DP [V]	CE [V]	CXP [V]	Internal standard
20	Cevimeline	200.07	156.1	100	31	10	Fenoterol-d6
			(126.1)		(45)	(8)	
20	Chloroquine	320.22	247.1	60	31	8	Fenoterol-d6
			(142.1)		(51)	(16)	
20	Cilastatin	359.1	97.1	50	34	18	Fenoterol-d6
			(69.1)		(55)	(12)	
20	Cinchonidin (-)	295.15	79.0	110	53	14	Fenoterol-d6
			(81.3)		(45)		
20	Clenbuterol	277.11	203	66	23	14	Fenoterol-d6
			(258.9)		(15)	(18)	
20	Clonidine	230	44	85	46	15	Fenoterol-d6
			(213.0)		(34)	(15)	
20	Clozapine	327.154	270.1	101	31	18	Fenoterol-d6
			(192.2)		(57)	(12)	
20	Crizotinib	450.3	260	100	34	17	Fenoterol-d6
			(177.1)		(52)	(11)	
20	Crotaline	326.2	120.2	120	50	15	Fenoterol-d6
20	Cycloguanil	252.2	195.1	75	25	10	Fenoterol-d6
			(153.0)		(41)	(10)	
20	Denopamine (R)	318.106	165.1	75	27	10	Fenoterol-d6
			(300.2)		(17)	(20)	
20	Deprenyl (R), (S)	188.161	91	50	28	16	Fenoterol-d6
			(119.0)		(17)	(8)	
20	Desvenlafaxine	264.3	107.2	60	50	6	Fenoterol-d6
			(58.1)		(47)	(10)	
20	Diethyltryptamine	217.265	86.0	51	19	16	Fenoterol-d6
			(143.9)		(27)	(10)	
20	Dimethyltryptamine	189.237	58.1	46	25	10	Fenoterol-d6
			(143.9)		(21)	(8)	
20	Dimetindene	293.228	248.2	79	28	14	Fenoterol-d6
			(58.0)		(48)	(10)	
20	Disopyramide	340.242	239.1	60	25	16	Fenoterol-d6
			(195.1)		(41)	(12)	
20	Dobutamine	302.202	137	66	30	10	Fenoterol-d6
			(106.9)		(37)	(6)	
20	Doxylamine (R), (S)	271.2	182.1	51	21	12	Fenoterol-d6
			(167.2)		(44)	(16)	
20	Dropropizine	237.114	120.1	76	37	8	Fenoterol-d6
			(160.1)		(33)	(10)	
20	Efaroxan	217.161	71.1	80	32	12	Fenoterol-d6
			(91.2)		(47)	(16)	
20	Eletriptan	383	144	85	40	10	Fenoterol-d6
			(84.0)		(40)	(16)	
20	Ergometrine	326.274	223.1	80	33	14	Fenoterol-d6
			(208.2)		(37)	(12)	

	Substance	Mass Q1 [Da]	Mass Q3 [Da]	DP [V]	CE [V]	CXP [V]	Internal standard
20	Esmolol	295.868	145.0	81	37	8	Fenoterol-d6
			(219.1)		(27)		
20	Fasudil	292.063	99.2	95	38	8	Fenoterol-d6
			(70.0)		(57)	(12)	
20	Fedratinib	525.325	98.2	120	64	6	Fenoterol-d6
			(70.0)		(112)	(14)	
20	Fenoldopam	306.3	185	80	29	12	Fenoterol-d6
			(106.9)		(37)	(20)	
20	Fenoterol	304.1	107.1	70	44	12	Fenoterol-d6
			(135.2)		(24)	(12)	
20	Fenoterol-d6	310.3	109.1	70	40	12	
			(141.0)		(26)	(12)	
20	Fenpiverinium	338.5	239.1	69	24	16	Fenoterol-d6
			(77.01)		(111)	(14)	
20	Folic acid	442.201	295.2	81	19	8	Fenoterol-d6
			(176.2)		(54)	(12)	
20	Formoterol	345.2	149.1	70	28	15	Fenoterol-d6
			(121.1)		(42)		
20	Gabapentin	172.157	137	36	15	8	Fenoterol-d6
			(153.9)		(19)	(10)	
20	Galantamine	288.221	213.1	86	31	14	Fenoterol-d6
			(231.2)		(23)	(16)	
20	Ganciclovir	256.042	152.0	50	17	10	Fenoterol-d6
			(135.2)		(45)	(8)	
20	Guanfacin	246.2	59.9	36	32	10	Fenoterol-d6
			(229.15)		(9)	(6)	
20	Hexanoylcarnitine	260.2	85.0	76	31	6	Fenoterol-d6
			(201.0)		(21)	(12)	
20	Hexylamine	102.17	74.0	56	21	14	Fenoterol-d6
20	Higenamine	272.058	107.1	76	34	6	Fenoterol-d6
			(255.0)		(21)	(18)	
20	Hydroxybupropion	256.091	238	55	17	16	Fenoterol-d6
			(139.0)		(37)	(8)	
20	Hydroxychloroquine (R), (S)	336.3	247.1	85	29	16	Fenoterol-d6
			(158.2)		(33)	(10)	
20	Hypaphorine	247	188.1	50	19	12	Fenoterol-d6
			(146.2)		(31)	(10)	
20	Ipratropium	332.3	166.2	100	36	12	Fenoterol-d6
			(124.0)		(45)	(12)	
20	Isovalerylcarnitine	246.2	85.0	71	29	6	Fenoterol-d6
20	Labetalol	329.4	311.2	71	19	15	Fenoterol-d6
			(162.0)		(35)	(10)	
20	Lamotrigine	256.0	211.0	100	36	13	Fenoterol-d6
			(187.0)		(37)		
20	Levofloxacin	362.2	318	71	27	22	Fenoterol-d6

	Substance	Mass Q1 [Da]	Mass Q3 [Da]	DP [V]	CE [V]	CXP [V]	Internal standard
			(261)		(37)	(18)	
20	m-Iodobenzylguanidin	276.09	217.0	76	29	14	Fenoterol-d6
			(90.0)		(54)	(16)	
20	Mecamylamine	168.179	137.1	66	15	8	Fenoterol-d6
			(80.9)		(27)	(14)	
20	Medetomidine	201.243	95.1	71	23	18	Fenoterol-d6
			(68.2)		(49)	(12)	
20	Mepenzolate	341.2	130	96	39	8	Fenoterol-d6
			(58.0)		(85)	(10)	
20	Meptazinol	234.469	107	96	35	20	Fenoterol-d6
			(76.9)		(73)	(6)	
20	Meropenem	384.209	67.9	61	70	12	Fenoterol-d6
			(141.1)		(23)	(8)	
20	Mescaline	211.91	165	46	31	10	Fenoterol-d6
			(195)		(15)	(12)	
20	Methoxytryptophan	235.17	176.0	56	25	12	Fenoterol-d6
			(148.0)		(33)	(10)	
20	Methylamphetamine	150.198	119	31	15	8	Fenoterol-d6
			(91.0)		(23)	(16)	
20	Methylbutyrylcarnitine	246.15	85.0	71	29	4	Fenoterol-d6
			(187.0)		(19)	(12)	
20	Methylenedioxyethyl-amphetamine	207.98	163.0	51	19	10	Fenoterol-d6
			(105.0)		(35)	(20)	
20	Methylergometrine	340.177	223.2	90	33	14	Fenoterol-d6
			(208.3)		(35)		
20	Methylnaltrexone	356.093	55.2	98	61	10	Fenoterol-d6
			(284.1)		(33)	(18)	
20	Methylphenidate	234.174	84.1	71	27	6	Fenoterol-d6
			(91.1)		(73)	(8)	
20	Methylscopolamine	319.4	152	109	37	10	Fenoterol-d6
			(45.1)		(49)	(8)	
20	Metoclopramide	300.182	227	65	25	14	Fenoterol-d6
			(183.9)		(42)	(12)	
20	Metoprolol	268.2	116.1	86	27	8	Fenoterol-d6
			(74)		(35)	(14)	
20	Mexiletine	180.161	58	50	21	10	Fenoterol-d6
			(77.0)		(52)	(14)	
20	Mexiletine (R), (S)	180.161	58	50	21	10	Fenoterol-d6
			(77.0)		(52)	(14)	
20	Midodrine	255.1	236.9	36	10	16	Fenoterol-d6
			(180)		(23)	(12)	
20	Milnacipran	247.174	230.2	51	17	14	Fenoterol-d6
			(100.1)		(27)	(8)	
20	Minocyclin	458.104	441.4	81	25	12	Fenoterol-d6
			(283.2)		(61)	(16)	

	Substance	Mass Q1 [Da]	Mass Q3 [Da]	DP [V]	CE [V]	CXP [V]	Internal standard
20	Mirabegron	397.14	379.1	71	20	12	Fenoterol-d6
			(146.1)		(39)	(18)	
20	Molsidomine	243.17	86.1	45	13	16	Fenoterol-d6
			(56.0)		(35)	(10)	
20	Moxonidine	242.057	44.2	103	49	8	Fenoterol-d6
			(206.3)		(25)	(12)	
20	N-Desmethylranitidine	301.168	176	65	21	12	Fenoterol-d6
			(102.1)		(46)	(6)	
20	N-Desmethyltramadol	177.97	161	43	17	10	Fenoterol-d6
			(131)		(27)	(16)	
20	N-Ethyl-lidocaine	264.2	86	81	36	16	Fenoterol-d6
			(58.0)		(64)	(11)	
20	N-Methyl-2-phenylethylamine	136.06	105.1	56	19	20	Fenoterol-d6
			(77.0)		(43)	(14)	
20	N-Methyltryptamine	175.105	144.0	61	17	8	Fenoterol-d6
			(132.0)		(15)		
20	Nadolol	310.06	254.1	66	23	16	Fenoterol-d6
			(201.0)		(31)	(16)	
20	Nafamostat	348.181	162	50	27	10	Fenoterol-d6
			(120.1)		(49)	(8)	
20	Nalbuphine	358.26	340.3	91	32	20	Fenoterol-d6
			(41.2)		(88)	(8)	
20	Nalmefene	340.252	322.2	61	29	10	Fenoterol-d6
			(55.1)		(63)	(10)	
20	Naloxone	328.2	310.3	86	27	20	Fenoterol-d6
			(253.2)		(35)	(16)	
20	Naltrexon	342.3	324.2	90	29	10	Fenoterol-d6
			(270.1)		(36)	(8)	
20	Naratriptan	336	241	85	30	16	Fenoterol-d6
			(70.0)		(50)	(13)	
20	Neostigmine	223.193	72.0	86	46	14	Fenoterol-d6
			(208.1)		(27)		
20	Nicotinamide	123.055	80.0	73	29	14	Fenoterol-d6
			(78.1)		(33)		
20	Nimodipine	419.2	402.3	75	17	12	Fenoterol-d6
			(45.2)		(84)	(8)	
20	Noroxycodone	302.426	284.1	56	25	8	Fenoterol-d6
			(187.0)		(35)	(14)	
20	Nortilidine	260.3	155.1	61	25	12	Fenoterol-d6
			(115.1)		(67)	(10)	
20	Ofloxacin	362.215	318.2	88	27	10	Fenoterol-d6
			(261.1)		(37)	(16)	
20	Ondansetron	294.1	170.1	100	35	15	Fenoterol-d6
20	Oxprenolol	266.155	72	61	33	14	Fenoterol-d6
			(225.1)		(19)	(14)	

	Substance	Mass Q1 [Da]	Mass Q3 [Da]	DP [V]	CE [V]	CXP [V]	Internal standard
20	Oxycodone	316.3	298.2	91	27	10	Fenoterol-d6
			(256.2)		(35)	(8)	
20	Oxytetracyclin	461.211	426.2	73	27	28	Fenoterol-d6
			(443.4)		(17)	(12)	
20	p-Methoxyamphetamin	166.05	149.1	36	13	10	Fenoterol-d6
			(121.1)		(25)	(15)	
20	p-Methoxymetamphetamine	180.05	149.0	46	17	9	Fenoterol-d6
			(121.0)		(28)	(22)	
20	Pancuronium	286.392	100.0	71	41	8	Fenoterol-d6
			(206.9)		(23)	(12)	
20	Pantothenic acid	220.054	90.2	51	19	16	Fenoterol-d6
			(202.2)		(17)	(14)	
20	Paraxanthine	181.25	124.0	70	27	8	Fenoterol-d6
			(67.0)		(43)		
20	Pentamidine	341.184	324.2	96	27	10	Fenoterol-d6
			(120.1)		(55)	(8)	
20	Pethidine	248.394	174.1	51	29	12	Fenoterol-d6
			(70.1)		(47)	(12)	
20	Phenformin	206	105	40	35	10	Fenoterol-d6
20	Phentermine	150.207	133	31	9	8	Fenoterol-d6
			(91.1)		(23)	(16)	
20	Pindolol	249.2	172	46	17	10	Fenoterol-d6
			(116.0)		(23)	(8)	
20	Pinoline	203.14	174	51	17	12	Fenoterol-d6
			(131.1)		(46)	(8)	
20	Pirenzepine	352.106	113.2	86	29	6	Fenoterol-d6
			(70.0)		(63)	(12)	
20	Practolol	267.104	190.1	71	25	12	Fenoterol-d6
			(148.1)		(32)	(10)	
20	Primaquine	260.126	86.1	56	23	16	Fenoterol-d6
			(243.2)		(17)		
20	Proguanil	254.2	170.2	75	24	10	Fenoterol-d6
			(153.1)		(40)	(10)	
20	Prucalopride	368.17	196.0	81	37	12	Fenoterol-d6
			(98.2)		(42)	(18)	
20	Quinine, Quinidine	325.207	172.0	100	46	12	Fenoterol-d6
			(78.9)		(60)	(14)	
20	Ractopamine	302.4	107.0	56	43	6	Fenoterol-d6
			(91.0)		(58)	(16)	
20	Rasagiline	172.109	117	40	11	10	Fenoterol-d6
			(56.1)		(41)	(8)	
20	Reproterol	390.2	221.1	70	35	15	Fenoterol-d6
			(372.2)		(24)		
20	Revefenacin	598.323	302.4	116	45	20	Fenoterol-d6
			(162.3)		(79)	(10)	

	Substance	Mass Q1 [Da]	Mass Q3 [Da]	DP [V]	CE [V]	CXP [V]	Internal standard
20	Ritodrine	288.35	270.1	59	19	18	Fenoterol-d6
			(121.1)		(31)	(8)	
20	Ropinirol	261.2	114.2	75	28	8	Fenoterol-d6
			(86.2)		(40)	(16)	
20	Scopolamine	304.3	138.1	80	30	15	Fenoterol-d6
20	Selegiline	188.2	91	41	27	16	Fenoterol-d6
			(119.3)		(17)	(8)	
20	Sepantronium	363.13	305.3	100	33	20	Fenoterol-d6
			(93.2)		(49)	(18)	
20	Strychnine	335.15	184.1	141	53	10	Fenoterol-d6
			(156.2)		(64)		
20	Succinylcarnitine	262.2	85.0	75	33	6	Fenoterol-d6
20	Tapentadol	222.1	107.1	70	32	15	Fenoterol-d6
20	Temozolomide	194.976	138.1	60	13	8	Fenoterol-d6
			(55.1)		(35)	(10)	
20	Tetracyclin	445.2	410.2	80	27	26	Fenoterol-d6
			(427.2)		(17)	(12)	
20	Tiotropium	392.081	152.1	81	40	10	Fenoterol-d6
			(170.1)		(45)	(10)	
20	Tofacitinib	313.173	149.2	100	38	10	Fenoterol-d6
			(98.2)		(43)	(18)	
20	Topotecan	422.158	377.2	96	27	24	Fenoterol-d6
			(46.2)		(53)	(8)	
20	Tramadol	264.216	58	50	44	10	Fenoterol-d6
			(56.1)		(87)	(4)	
20	Tranylcypromine	134.048	117	41	13	8	Fenoterol-d6
			(115)		(27)		
20	Tropisetron	285.2	124.2	100	30	10	Fenoterol-d6
20	Trospium	392.3	164.1	120	42	12	Fenoterol-d6
			(182.2)		(47)	(12)	
20	Tryptophan-d5 (L)	210.144	192.1	39	14	12	Fenoterol-d6
			(193.1)		(9)		
20	Tubocurarin	609.34	58.1	109	109	10	Fenoterol-d6
			(564.2)		(45)	(36)	
20	Tulobuterol	228.125	153.9	60	23	10	Fenoterol-d6
			(119.1)		(41)	(8)	
20	Urapidil	388.268	233.1	91	33	16	Fenoterol-d6
			(190.0)		(47)	(12)	
20	Varenicline	212.152	169.1	95	29	12	Fenoterol-d6
			(183.1)		(31)	(12)	
20	Vecuronium	557.384	100.1	136	75	6	Fenoterol-d6
			(356.4)		(56)	(22)	
20	Xamoterol	340.219	253.1	81	22	16	Fenoterol-d6
			(157.0)		(33)	(10)	
20	Ximelagatran	474.263	198.2	70	31	12	Fenoterol-d6

	Substance	Mass Q1 [Da]	Mass Q3 [Da]	DP [V]	CE [V]	CXP [V]	Internal standard
			(249.1)		(29)	(18)	
20	Xylometazolin	245.3	189	116	35	12	Fenoterol-d6
			(145.0)		(59)	(10)	
20	Yohimbin	355.203	144.2	110	42	10	Fenoterol-d6
			(212.1)		(33)	(14)	
	**35% organic additive** (64.9% H_2O, 0.1% formic acid, 30.0% acetonitrile, 5.0% methanol)						
35	1-(3-Chlorophenyl)piperazine-d8	205.171	158.1	85	29	10	Bupivacaine
			(123.2)		(37)	(8)	
35	1,3-Diphenylguanidine	212.1	119.1	96	29	22	Bupivacaine
			(77.0)		(55)	(4)	
35	10-OH-Nortriptyline	280.063	262.17	56	17	18	Bupivacaine
			(216.116)		(33)	(14)	
35	2,5-Dimethoxy-4-iodo-amphetamine	321.98	305.0	51	17	20	Bupivacaine
			(277.0)		(27)	(18)	
35	3-Iodothyronamine	356.0	339.1	63	17	22	Bupivacaine
			(212.1)		(26)	(14)	
35	3-Methoxymorphinan	258.446	215.1	106	29	14	Bupivacaine
			(171.2)		(49)	(10)	
35	4-Amino-1,8-naphthalimide	212.99	195.1	91	29	12	Bupivacaine
			(140.1)		(52)	(10)	
35	Abemaciclib	507.402	393.4	101	27	12	Bupivacaine
			(245.1)		(81)		
35	Abrocitinib	324.196	149.3	101	35	10	Bupivacaine
			(70.0)		(44)	(12)	
35	Aclidinium	484.185	262.2	110	45	18	Bupivacaine
			(140.2)		(72)	(10)	
35	Aconitine	646.313	586.3	126	45	18	Bupivacaine
			(105.1)		(89)	(6)	
35	Alfuzosine	390.2	156.1	90	37	10	Bupivacaine
			(235.1)		(37)	(16)	
35	Aliskiren	552.348	436.4	100	27	14	Bupivacaine
			(534.5)		(25)	(18)	
35	Almotriptan	336.219	58.2	71	52	10	Bupivacaine
			(201.2)		(22)	(14)	
35	Alprenolol	250.156	116.2	56	23	6	Bupivacaine
			(56.1)		(44)	(10)	
35	Ambroxol	379.063	264	71	25	16	Bupivacaine
			(116.1)			(6)	
35	Amitriptyline	278.2	117.1	36	30	8	Bupivacaine
			(91.0)		(36)	(16)	
35	Amitriptyline-d6	284.3	91.039	20	30	15	Bupivacaine
35	Amlodipine	409.3	238	55	14	15	Bupivacaine
			(206.0)		(34)	(15)	
35	Amoxapine	314.114	271.3	100	33	16	Bupivacaine
			(193.2)		(60)	(12)	

	Substance	Mass Q1 [Da]	Mass Q3 [Da]	DP [V]	CE [V]	CXP [V]	Internal standard
35	APC366	441.3	271.2	96	31	18	Bupivacaine
			(70.2)		(93)	(14)	
35	Apixaban	460.237	443.5	121	32	12	Bupivacaine
			(77.3)		(90)	(6)	
35	Aspartame	295.1	120	76	36	8	Bupivacaine
			(235.2)		(18)	(16)	
35	Atomoxetine	256.156	44.2	46	35	8	Bupivacaine
			(148.1)		(11)	(10)	
35	Bambuterol	368.197	294.2	71	27	10	Bupivacaine
			(72.1)		(59)	(14)	
35	Baricitinib	372.028	251.3	96	40	16	Bupivacaine
			(186.3)		(43)	(12)	
35	Befunolol	292.2	56.1	80	50	10	Bupivacaine
			(43.0)		(66)	(8)	
35	Berberine	336.199	320.1	90	40	20	Bupivacaine
			(292.2)		(40)	(18)	
35	Betaxolol	308.278	116.2	96	28	8	Bupivacaine
			(55.0)		(54)	(10)	
35	Bicifadine	174.17	133.1	66	23	8	Bupivacaine
			(105.0)		(24)	(8)	
35	Biotin	245.078	227.1	70	19	14	Bupivacaine
			(97.2)		(44)	(18)	
35	Biperiden	312.237	98.2	80	36	18	Bupivacaine
			(70.0)		(72)	(12)	
35	Bisoprolol	326.3	116.1	85	25	8	Bupivacaine
			(74.1)		(41)	(6)	
35	Bitolterol	462.211	445.2	70	19	14	Bupivacaine
			(89.1)		(44)	(16)	
35	Bortezomib	367.176	226.1	96	27	14	Bupivacaine
			(208.2)		(39)		
35	Brofaromine	312.1	217	106	35	14	Bupivacaine
			(82.0)		(43)	(16)	
35	Bromocriptin	654.3	636.3	91	23	20	Bupivacaine
			(301.3)		(51)		
35	Bunitrolol	249.076	193.1	50	20	12	Bupivacaine
			(120.1)		(36)	(22)	
35	Bupivacaine	289.248	140.1	75	30	8	Amitryptiline-d6
			(84.2)		(58)	(16)	
35	Buspirone	386.212	122.2	100	42	10	Bupivacaine
			(95.1)		(71)	(8)	
35	Captopril	218.124	69.9	40	35	14	Bupivacaine
			(46.1)		(23)	(8)	
35	Celiprolol	380.289	251.1	86	32	16	Bupivacaine
			(74.1)		(50)	(14)	
35	Cetirizin	389.19	201.1	55	25	12	Bupivacaine

	Substance	Mass Q1 [Da]	Mass Q3 [Da]	DP [V]	CE [V]	CXP [V]	Internal standard
			(166.1)		(55)		
35	Chlorhexidine	506.344	489.4	70	21	16	Bupivacaine
			(133.0)		(33)	(8)	
35	Chlorpheniramine	275.116	230	50	21	14	Bupivacaine
			(167.0)		(55)	(10)	
35	Chlorpromazine	319.116	58.1	80	59	10	Bupivacaine
			(86.2)		(29)	(16)	
35	Ciprofloxacin	332.19	288.2	85	25	20	Bupivacaine
			(314.2)		(28)		
35	Citalopram	325.155	109.1	100	34	6	Bupivacaine
			(262.2)		(27)	(18)	
35	Clarithromycin	748.539	158.3	91	42	10	Bupivacaine
			(590.4)		(27)	(18)	
35	Clemastin	344.4	215	55	22	14	Bupivacaine
			(130.2)		(16)	(7)	
35	Clidinium	353.179	142	105	43	8	Bupivacaine
			(143.1)		(45)	(10)	
35	Clindamycin	425.236	126.0	101	41	8	Bupivacaine
			(70.1)		(91)	(12)	
35	Cocaine	304.338	182	41	27	12	Bupivacaine
			(77)		(77)	(14)	
35	Coluracetam	342.359	217.1	96	35	14	Bupivacaine
			(98.3)		(49)	(18)	
35	Coptisine	320.101	292.3	100	39	18	Bupivacaine
			(204.2)		(77)	(12)	
35	Cyclopentolate	292.171	274.3	60	18	18	Bupivacaine
			(72.1)		(39)	(14)	
35	Dapson	249.132	156.0	80	21	10	Bupivacaine
			(108.1)		(30)	(8)	
35	Daunorubicin	528.2	321	63	25	10	Bupivacaine
			(363)		(19)	(11)	
35	Dehydrocorydaline	366.132	350.2	101	41	22	Bupivacaine
			(322.3)		(42)	(20)	
35	Denatonium	326.5	91.01	71	51	16	Bupivacaine
			(86.06)		(29)	(16)	
35	Desipramine	267.147	72.1	76	29	12	Bupivacaine
			(44.2)		(61)	(8)	
35	Dextromethorphan	272.2	215.2	105	33	14	Bupivacaine
			(171.1)		(53)	(12)	
35	Dihydroergotamine	584.321	270.2	121	45	16	Bupivacaine
			(253.3)		(47)		
35	Diltiazem	415.166	178	96	33	12	Bupivacaine
			(150.0)		(60)	(10)	
35	Dimenhydrinate	470.311	171.2	66	29	12	Bupivacaine
			(57.1)		(77)	(10)	

	Substance	Mass Q1 [Da]	Mass Q3 [Da]	DP [V]	CE [V]	CXP [V]	Internal standard
35	Diphenhydramine	256.2	167	46	17	10	Bupivacaine
			(152.0)		(49)	(10)	
35	Dipyridamole	505.362	385.3	141	58	12	Bupivacaine
			(429.2)		(56)	(12)	
35	Dofetilide	442.088	198.1	106	39	12	Bupivacaine
			(120.3)		(65)	(10)	
35	Domperidone	426.27	174.9	66	39	12	Bupivacaine
			(147.0)		(57)	(10)	
35	Donepezil	380.278	91.1	56	59	16	Bupivacaine
			(43.1)		(61)	(8)	
35	Doxazosin	452.185	344.2	110	43	10	Bupivacaine
			(247.1)		(55)	(16)	
35	Doxepin	280.145	107.1	61	31	6	Bupivacaine
			(77.0)		(75)	(14)	
35	Doxepine (E), (Z)	280.145	107.1	61	31	6	Bupivacaine
			(77.0)		(75)	(14)	
35	Doxycycline	445.17	428.2	55	25	14	Bupivacaine
35	Duloxetine	298.146	154	46	9	10	Bupivacaine
			(44.1)		(33)	(8)	
35	Epiberberine	336.027	320.2	101	43	20	Bupivacaine
			(292.3)		(45)	(18)	
35	Felbamat	239.1	178.0	61	9	12	Bupivacaine
			(117.1)		(22)	(8)	
35	Fenfluramine (R), (S)	232.122	159.2	66	29	14	Bupivacaine
			(109.1)		(59)	(6)	
35	Fentanyl	337.35	188.2	91	33	12	Bupivacaine
			(76.9)		(107)	(6)	
35	Fesoterodin	412.4	223.2	95	44	14	Bupivacaine
			(370.4)		(33)	(12)	
35	Filgotinib	426.291	291.3	90	37	18	Bupivacaine
			(358.4)		(32)	(10)	
35	Flecainide	415.144	398.2	121	33	12	Bupivacaine
			(301.2)		(49)	(28)	
35	Fluoxetine	310.159	44.1	56	39	8	Bupivacaine
			(148.1)		(13)	(10)	
35	Fluvoxamine	319.167	71.1	61	33	12	Bupivacaine
			(44.9)		(53)	(8)	
35	Gabexate	322.083	96.1	106	34	18	Bupivacaine
			(121.1)		(32)	(8)	
35	Gefitinib	447.175	128.2	106	35	8	Bupivacaine
			(100.1)		(79)	(6)	
35	Glycopyrrolate	318.313	116.1	90	41	8	Bupivacaine
			(58.3)		(77)	(10)	
35	Granisetron	313.2	138.1	106	32	8	Bupivacaine
			(159.1)		(44)	(10)	

	Substance	Mass Q1 [Da]	Mass Q3 [Da]	DP [V]	CE [V]	CXP [V]	Internal standard
35	Harmaline	214.8	174.1	96	31	12	Bupivacaine
			(200.1)		(31)	(12)	
35	Ibogaine	311.269	122.3	121	45	10	Bupivacaine
			(174.1)				
35	Ibutilid	385.3	367.4	96	25	10	Bupivacaine
			(144.1)		(41)	(8)	
35	Imatinib	494.273	394.2	110	35	12	Bupivacaine
			(217.2)			(14)	
35	Imipramine	281.154	86.1	91	23	16	Bupivacaine
			(58.1)		(59)	(10)	
35	Irinotecan	587.24	124.2	150	49	10	Bupivacaine
			(502.2)		(45)	(16)	
35	Ivabradine	469.329	177.2	83	33	12	Bupivacaine
			(262.1)			(16)	
35	Ketamine	239.026	126.1	50	37	8	Bupivacaine
			(125.1)		(34)		
35	Lafutidine	432.226	351.2	66	23	10	Bupivacaine
			(193.2)		(37)	(12)	
35	Landiolol	510.251	157.1	80	48	14	Bupivacaine
			(143.2)		(48)	(14)	
35	Lenalidomide	260.115	149.1	71	21	10	Bupivacaine
			(187.2)		(33)	(12)	
35	Levomepromazin	329.3	100.1	76	27	18	Bupivacaine
			(58.2)		(58)	(10)	
35	Lidocain	235.1	86.2	71	26	16	Bupivacaine
			(158.9)		(11)	(12)	
35	Maprotiline	278.219	250.2	85	27	16	Bupivacaine
			(191.1)		(47)	(12)	
35	Maraviroc	514.4	389.4	94	27	12	Bupivacaine
			(280.2)		(44)	(18)	
35	Memantin	180.19	163.1	46	21	10	Bupivacaine
			(107.0)		(35)	(6)	
35	Mepivacaine	247.2	98	77	28	5	Bupivacaine
35	Methylene blue	284	268	101	47	8	Bupivacaine
			(251.1)		(67)	(16)	
35	Methysergide	354.161	237.1	106	33	16	Bupivacaine
			(221.2)		(36)	(14)	
35	Metipranolol	310.234	191.2	96	31	12	Bupivacaine
			(116.1)		(28)	(6)	
35	Minoxidil	210.15	193.2	70	21	12	Bupivacaine
			(164.1)		(32)	(12)	
35	Moxifloxacin	402.2	384.2	90	30	12	Bupivacaine
			(110.2)		(37)	(6)	
35	N-Desmethylcitalopram	311.189	109	86	33	6	Bupivacaine
			(262.1)		(23)	(18)	

	Substance	Mass Q1 [Da]	Mass Q3 [Da]	DP [V]	CE [V]	CXP [V]	Internal standard
35	N-Desmethylvenlafaxine	264.1	246.1	66	15	16	Bupivacaine
			(215.1)		(21)	(14)	
35	Nebivolol	406.162	151.1	110	42	10	Bupivacaine
			(44.0)		(67)	(8)	
35	Nefazodone	470.299	274.2	100	39	18	Bupivacaine
			(83.0)		(64)	(16)	
35	Norfenfluramine (+)	204.112	159	56	24	10	Bupivacaine
			(187.0)		(15)	(12)	
35	Nortriptyline	264.2	233.2	46	21	16	Bupivacaine
			(91.1)		(30)	(6)	
35	Olodaterol	387.199	163.1	70	27	10	Bupivacaine
			(207.0)		(28)	(14)	
35	Orphenadrine	270.24	181.0	51	15	12	Bupivacaine
			(166.2)		(39)	(10)	
35	Oseltamivir	313.163	225.2	76	13	14	Bupivacaine
			(166.0)		(25)	(10)	
35	Oxymetazoline	261.173	205.2	111	35	12	Bupivacaine
			(135.2)		(45)	(8)	
35	Oxyphenonium	348.237	73	96	47	14	Bupivacaine
			(132.2)		(41)	(8)	
35	Paliperidone	427.294	207.1	90	37	14	Bupivacaine
			(110.0)		(60)	(6)	
35	Palmatin	352.156	336.3	90	39	20	Bupivacaine
			(308.2)				
35	Palonosetron	297.177	110	115	37	6	Bupivacaine
			(81.9)		(52)	(16)	
35	Paracetamol	152.106	109.9	65	21	6	Bupivacaine
			(65.1)		(41)	(12)	
35	Paroxetine	330.183	70	61	51	12	Bupivacaine
			(192.1)		(29)	(12)	
35	Perphenazine	404.267	171.3	75	33	10	Bupivacaine
			(143.2)		(40)	(10)	
35	Pimozide	462.228	445.3	70	19	14	Bupivacaine
			(45.1)		(83)	(8)	
35	Pioglitazone	357.098	134.2	106	39	8	Bupivacaine
			(119.1)		(67)	(10)	
35	Pipamperone	376.3	165.2	56	39	10	Bupivacaine
			(98)		(43)	(8)	
35	Piperacillin	518.28	143.1	100	26	10	Bupivacaine
			(160.2)		(15)		
35	Piritramide	431.274	98.1	95	45	18	Bupivacaine
			(346.2)		(27)	(10)	
35	Pomalidomide	274.138	84.1	71	19	14	Bupivacaine
			(201.2)		(29)		
35	Prazosine	384.197	95	120	72	18	Bupivacaine

	Substance	Mass Q1 [Da]	Mass Q3 [Da]	DP [V]	CE [V]	CXP [V]	Internal standard
			(247.0)		(39)	(16)	
35	Pridinol	296.272	98.1	76	25	18	Bupivacaine
			(70.2)		(65)	(12)	
35	Propafenone	342.211	116.1	80	31	8	Bupivacaine
			(72.0)		(48)	(12)	
35	Propanthelin	368.125	181.1	75	40	12	Bupivacaine
			(100.2)		(32)	(18)	
35	Propiverine	368.3	116.3	110	45	12	Bupivacaine
			(105)		(65)	(18)	
35	Propranolol	260.2	116.2	80	25	8	Bupivacaine
			(183.0)			(12)	
35	Pyrilamine	286.236	121.1	56	33	22	Bupivacaine
			(241.2)		(19)	(22)	
35	Pyrimethamine	250.2	177.9	45	39	12	Bupivacaine
			(129.0)		(23)	(10)	
35	Quetiapine	384.1	253.1	71	31	16	Bupivacaine
			(221.2)		(50)	(14)	
35	Raloxifen	474.208	112.1	131	47	6	Bupivacaine
			(84.1)		(88)	(16)	
35	Ranolazin	428.308	279.3	100	33	18	Bupivacaine
			(98.2)		(58)		
35	Reboxetine	314.214	176	55	19	12	Bupivacaine
			(91.1)		(42)	(16)	
35	Remoxipride	371.124	112	91	35	6	Bupivacaine
			(242.9)		(37)	(16)	
35	Reserpine	609.292	195.1	140	52	12	Bupivacaine
			(174.0)		(59)	(12)	
35	Riboflavin	377.183	243.1	76	33	16	Bupivacaine
			(172.1)		(53)	(12)	
35	Risperidon	411.3	191.2	96	39	18	Bupivacaine
			(69.2)		(83)	(12)	
35	Rivastigmin	251.1	206.1	51	19	14	Bupivacaine
			(86.1)		(33)	(16)	
35	Ropivacaine	275.267	126.2	81	29	8	Bupivacaine
			(84.1)		(59)	(16)	
35	Rucaparib	324.2	293.2	46	13	18	Bupivacaine
			(236.0)		(47)	(14)	
35	Ruxolitinib	307.245	186.2	100	38	12	Bupivacaine
			(105.1)		(79)	(6)	
35	Sitagliptin	408.216	235.1	90	27	16	Bupivacaine
			(174.0)		(37)	(12)	
35	Solifenacin	363.3	110.1	106	37	6	Bupivacaine
			(193.1)		(41)	(12)	
35	Sufentanil	387.152	238.1	81	27	8	Bupivacaine
			(110.9)		(53)	(8)	

	Substance	Mass Q1 [Da]	Mass Q3 [Da]	DP [V]	CE [V]	CXP [V]	Internal standard
35	Talinolol	364.245	308.3	85	27	10	Bupivacaine
			(100.1)		(33)	(8)	
35	Tamsulosin	409.227	228.1	91	33	14	Bupivacaine
			(271.1)		(27)	(18)	
35	Terazosin	388.2	290.2	94	37	8	Bupivacaine
			(247.1)		(43)	(16)	
35	Tetracaine	265.1	176.1	61	20	10	Bupivacaine
			(72.3)		(37)	(14)	
35	Thalidomide	259.089	84.1	75	17	14	Bupivacaine
			(231.2)		(13)	(16)	
35	Tianeptin	437.149	292.1	56	21	18	Bupivacaine
			(228.1)		(51)	(14)	
35	Timolol	317.185	261	80	23	18	Bupivacaine
			(74.1)		(40)	(14)	
35	Tolterodine (R), (S)	326.2	284.3	95	31	8	Bupivacaine
			(147.0)		(38)	(10)	
35	Trimipramine	295.25	100.1	65	24	18	Bupivacaine
			(58.2)		(59)	(10)	
35	Tropicamide	285.188	255.2	91	29	18	Bupivacaine
			(93.2)		(53)	(18)	
35	Upadacitinib	381.266	256.3	119	39	16	Bupivacaine
			(213.2)		(55)	(14)	
35	Venlafaxine	278.2	121	65	39	15	Bupivacaine
			(58.1)		(47)	(10)	
35	Verapamil	455.2	165.1	113	39	10	Bupivacaine
			(150.1)		(54)	(10)	
35	Viloxazine	238.114	56.2	76	46	10	Bupivacaine
			(100.1)		(24)	(8)	
35	Ziprasidone	413.107	194.1	105	39	12	Bupivacaine
			(130.1)		(93)	(8)	
	**50% organic additive** (49.9% ddH2O, 0.1% formic acid, 42.9% acetonitrile, 7.1% methanol)						
50	17α-Methyltestosterone	303.273	109	100	39	6	Propranolol
			(97.0)		(37)	(18)	
50	Agomelatine	244	185.1	71	21	12	Propranolol
			(226.2)		(13)	(14)	
50	Amiodarone	646.025	58.1	110	95	12	Propranolol
			(86.2)		(62)	(16)	
50	Aripiprazole	448.1	285.2	111	37	18	Propranolol
			(176.2)		(45)	(10)	
50	Bumetanide	365	184	60	31	10	Propranolol
			(240.0)		(31)	(10)	
50	Capsaicin	306.262	137.1	61	20	8	Propranolol
			(94.2)		(72)		
50	Carvedilol (R), (S)	407.155	100	86	42	18	Propranolol
			(56.1)		(79)	(10)	

	Substance	Mass Q1 [Da]	Mass Q3 [Da]	DP [V]	CE [V]	CXP [V]	Internal standard
50	Chlomipramine	316.24	86.0	61	28	16	Propranolol
			(58.1)		(67)	(10)	
50	Chloramphenicol	322.97	151.9	-75	-23	-9	Ibuprofen
			(150.8)		(-29)		
50	Chlorprothixene	316.1	271	85	28	18	Propranolol
			(230.9)		(37)	(15)	
50	Corticosterone	347.217	121.1	95	34	8	Propranolol
			(91.0)		(78)	(16)	
50	Cyclophosphamide	261.129	139.9	66	31	8	Propranolol
			(106.0)		(26)	(8)	
50	Decanoyl-L-carnitine-d3	319.311	85.0	66	37	16	Propranolol
			(257.2)		(21)		
50	Delavirdine	457.22	221.2	110	35	14	Propranolol
			(178.2)		(51)	(12)	
50	Diclofenac	296.1	215	60	27	14	Propranolol
			(250.0)		(19)	(16)	
50	Domperidone	424.1	131.1	-120	-74	-7	
			(166.2)		(-56)	(-9)	
50	Endoxifen	316.1	58.1	100	51	10	Propranolol
			(152.1)		(27)	(10)	
50	Estrone-3-sulfate	348.917	269.3	-100	-45	-15	Ibuprofen
			(145.1)		(-74)	(-9)	
50	Etomidate	245.108	141	43	14	10	Propranolol
			(95.0)		(32)	(18)	
50	Fexofenadine	502.322	466.4	100	37	14	Propranolol
			(171.1)		(53)	(10)	
50	Fingolimod	308.362	255.3	66	21	16	Propranolol
			(105.2)		(37)	(6)	
50	Flunarizine	405.303	203.0	60	23	14	Propranolol
			(183.2)		(63)	(12)	
50	Fluphenazine	438.204	171.2	100	35	10	Propranolol
			(143.2)		(42)	(10)	
50	Furosemide	329.1	285.1	-65	-20	-7	Domperidone
			(204.6)		(-30)	(-11)	
50	Gentian violet	372.222	356.3	140	54	10	Propranolol
			(340.4)		(72)	(20)	
50	Haloperidol	376.1	165.2	86	33	10	Propranolol
			(123.2)		(56)	(8)	
50	Hydrochlorothiazide	296.0	269.0	-80	-28	-15	Domperidone
			(205.0)		(-32)		
50	Ibuprofen	204.9	161.1	-50	-10	-9	
			(159.1)				
50	Ifosfamide	261.044	92.1	88	35	16	Propranolol
			(154.1)		(31)	(10)	
50	Indacaterol	393.239	375.4	82	21	10	Propranolol

	Substance	Mass Q1 [Da]	Mass Q3 [Da]	DP [V]	CE [V]	CXP [V]	Internal standard
			(173.0)		(33)		
50	Levetiracetam	171.1	125.9	55	19	10	Propranolol
			(69.0)		(38)		
50	Miconazole	417.024	158.9	100	40	10	Propranolol
			(161.0)		(41)	(12)	
50	Ochratoxin A	404.146	239.2	70	32	16	Propranolol
			(358.0)		(19)	(10)	
50	Oxiconazole	430.029	82.0	90	54	6	Propranolol
			(81.1)		(89)	(16)	
50	Pentorbital	225.1	42.0	-60	-40	-5	Chloramphenicol
			(182.2)		(-18)	(-9)	
50	Propranolol	260.2	116.2	80	25	8	
			(183.0)			(12)	
50	Prostaglandin E2	351.212	315.3	-75	-16	-9	Chloramphenicol
			(333.4)				
50	Prostaglandin F2 alpha	353.217	309.1	-90	-26	-7	Chloramphenicol
			(193.0)		(-35)	(-11)	
50	Pyrithiamine	260.1	138.1	46	17	8	Propranolol
			(107.1)		(60)	(6)	
50	Rifampicin	823.395	791.3	100	23	28	Propranolol
			(95.0)		(92)	(8)	
50	Salmeterol	416.299	398.3	70	21	12	Propranolol
			(380.3)		(27)		
50	Sertraline	306.149	275.0	55	17	18	Propranolol
			(159.0)		(33)	(10)	
50	Tamoxifen	372.232	71.9	90	47	14	Propranolol
			(70.0)		(75)	(6)	
50	Terfenadine	472.295	436.5	114	36	12	Propranolol
			(57.2)		(71)	(10)	
50	Umeclidinium	428.258	96.2	120	68	18	Propranolol
			(91.0)		(77)	(18)	
50	Zotepin	332.2	72.1	60	36	14	Propranolol
			(70.0)		(64)		

Table S7: Substrates of the four transporters predicted from 10,010 low molecular weight substances in drugbank

Transport	Substance	HGB P(0)	HGB P(1)	Prediction HGB	RF P(0)	RF P(1)	Prediction RFC
MATE1	Methylbenactyzium	0.000	1.000	1	0.054	0.946	1
MATE1	Metaraminol	0.001	0.999	1	0.049	0.951	1
MATE1	Oxilofrine	0.001	0.999	1	0.049	0.951	1
MATE1	Poldine	0.001	0.999	1	0.053	0.947	1
MATE1	Penthienate	0.001	0.999	1	0.061	0.939	1
MATE1	Pipenzolate	0.001	0.999	1	0.062	0.938	1
MATE1	Bevonium	0.001	0.999	1	0.065	0.935	1
MATE1	Levosalbutamol	0.002	0.998	1	0.060	0.940	1
MATE1	Norfenefrine	0.002	0.998	1	0.099	0.901	1
MATE1	Rimiterol	0.002	0.998	1	0.108	0.892	1
MATE1	(2s)-2-Amino-4-(Methylsulfanyl)-1-Pyridin-2-Ylbutane-1.1-Diol	0.002	0.998	1	0.140	0.860	1
MATE1	1-(2-Ethanone)-2-Hydroxy-2-(1-Amino-2-Methyl-2-Ethanol)-4-(2-Dimethyl)Ethane-Imidazoline-5-One-Chromophore (Thr-Leu-Gly)	0.002	0.998	1	0.155	0.845	1
MATE1	Tiemonium iodide	0.002	0.998	1	0.155	0.845	1
MATE1	N-(3.5-dimethoxyphenyl)imidodicarbonimidic diamide	0.002	0.998	1	0.164	0.836	1
MATE1	Nalpha-(2-Naphthylsulfonylglycyl)-3-Amidino-D.L-Phenylalanine-Isopropylester	0.002	0.998	1	0.257	0.743	1
MATE1	4-(2.5-DIAMINO-5-HYDROXY-PENTYL)-PHENOL	0.003	0.997	1	0.101	0.899	1
MATE1	L-tyrosinamide	0.003	0.997	1	0.121	0.879	1
MATE1	(2S)-2-AMINO-4-(METHYLSULFANYL)-1-(1.3-THIAZOL-2-YL)BUTANE-1.1-DIOL	0.003	0.997	1	0.145	0.855	1
MATE1	5-Amidino-Benzimidazole	0.003	0.997	1	0.174	0.826	1
MATE1	Melevodopa	0.003	0.997	1	0.179	0.821	1
MATE1	CRA_1144	0.003	0.997	1	0.182	0.818	1
MATE1	Rimeporide	0.003	0.997	1	0.226	0.774	1
MATE1	4-(Hydroxymethyl)Benzamidine	0.003	0.997	1	0.243	0.757	1
MATE1	2-[2-(2-Cyclohexyl-2-guanidino-acetylamino)-acetylamino]-N-(3-mercapto-propyl)-propionamide	0.003	0.997	1	0.248	0.752	1
MATE1	Elamipretide	0.003	0.997	1	0.418	0.582	1
MATE1	Levonordefrin	0.004	0.996	1	0.061	0.939	1
MATE1	Dimetofrine	0.004	0.996	1	0.090	0.910	1
MATE1	4-Sulfonamide-[1-(4-Aminobutane)]Benzamide	0.004	0.996	1	0.147	0.853	1
MATE1	Methantheline	0.004	0.996	1	0.155	0.845	1
MATE1	1-[(2R)-2-aminobutanoyl]-N-(4-carbamimidoylbenzyl)-L-prolinamide	0.004	0.996	1	0.205	0.795	1
MATE1	Bephenium	0.004	0.996	1	0.212	0.788	1

Transport	Substance	HGB P(0)	HGB P(1)	Prediction HGB	RF P(0)	RF P(1)	Prediction RFC
MATE1	M-(N.N.N-Trimethylammonio)-2.2.2-Trifluoro-1.1-Dihydroxyethylbenzene	0.004	0.996	1	0.227	0.773	1
MATE1	Hexocyclium	0.004	0.996	1	0.262	0.738	1
MATE1	Zinc-[amino-[2-[[5-[amino(azaniumylidene)methyl]benzimidazol-1-id-2-yl]methyl]benzimidazol-1-id-5-yl]methylidene]azanium	0.004	0.996	1	0.288	0.712	1
MATE1	Bis(5-Amidino-Benzimidazolyl)Methane	0.004	0.996	1	0.296	0.704	1
MATE1	2-[N'-(4-AMINO-BUTYL)-HYDRAZINOCARBONYL]-PYRROLIDINE-1-CARBOXYLIC ACID BENZYL ESTER	0.005	0.995	1	0.159	0.841	1
MATE1	Propamidine	0.005	0.995	1	0.164	0.836	1
MATE1	Amprolium	0.005	0.995	1	0.246	0.754	1
MATE1	(S)-N-(4-carbamimidoylbenzyl)-1-(2-(cyclopentylamino) ethanoyl)pyrrolidine-2-carboxamide	0.005	0.995	1	0.246	0.754	1
MATE1	1-ETHOXYCARBONYL-D-PHE-PRO-2(4-AMINOBUTYL)HYDRAZINE	0.005	0.995	1	0.287	0.713	1
MATE1	D-phenylalanyl-N-{4-[amino(iminio)methyl]benzyl}-L-prolinamide	0.005	0.995	1	0.301	0.699	1
MATE1	Chelerythrine	0.006	0.994	1	0.131	0.869	1
MATE1	Benzilone	0.006	0.994	1	0.151	0.849	1
MATE1	4-AMINO-2-HEXYLOXY-6-HYDROXYMETHYL-TETRAHYDRO-PYRAN-3.5-DIOL	0.006	0.994	1	0.212	0.788	1
MATE1	Bibenzonium	0.006	0.994	1	0.228	0.772	1
MATE1	D-leucyl-N-(4-carbamimidoylbenzyl)-L-prolinamide	0.006	0.994	1	0.234	0.766	1
MATE1	2.4.6-Triaminoquinazoline	0.006	0.994	1	0.296	0.704	1
MATE1	4-[(6-Amino-4-Pyrimidinyl)Amino]Benzenesulfonamide	0.006	0.994	1	0.300	0.700	1
MATE1	Methylatropine	0.007	0.993	1	0.124	0.876	1
MATE1	2-(2-hydroxy-phenyl)-3H-benzoimidazole-5-carboxamidine	0.007	0.993	1	0.153	0.847	1
MATE1	4-(METHYLSULFONYL)BENZENECARBOXIMIDAMIDE	0.007	0.993	1	0.223	0.777	1
MATE1	Alagebrium	0.007	0.993	1	0.260	0.740	1
MATE1	Tyrosinal	0.007	0.993	1	0.309	0.691	1
MATE1	4-({5-[(4-AMINOCYCLOHEXYL)AMINO][1.2.4]TRIAZOLO[1.5-A]PYRIMIDIN-7-YL}AMINO)BENZENESULFONAMIDE	0.007	0.993	1	0.327	0.673	1
MATE1	Phenylalanylamide	0.007	0.993	1	0.349	0.651	1
MATE1	N-Methylnaloxonium	0.008	0.992	1	0.121	0.879	1
MATE1	Anisotropine methylbromide	0.008	0.992	1	0.195	0.805	1
MATE1	NB-001	0.008	0.992	1	0.211	0.789	1
MATE1	Mafenide	0.008	0.992	1	0.218	0.782	1

Transport	Substance	HGB P(0)	HGB P(1)	Prediction HGB	RF P(0)	RF P(1)	Prediction RFC
MATE1	1-[(4S)-4-amino-5-(1.3-benzothiazol-2-yl)-5-oxopentyl]guanidine	0.008	0.992	1	0.229	0.771	1
MATE1	7-Methoxy-8-[1-(Methylsulfonyl)-1h-Pyrazol-4-Yl]Naphthalene-2-Carboximidamide	0.008	0.992	1	0.250	0.750	1
MATE1	METHYL 4-{[({[(2R.5S)-5-{[(2S)-2-(AMINOMETHYL)PYRROLIDIN-1-YL]CARBONYL}PYRROLIDIN-2-YL]METHYL}AMINO)CARBONYL]AMINO}BENZOATE	0.008	0.992	1	0.278	0.722	1
MATE1	2-(2-Hydroxy-5-Methoxy-Phenyl)-1h-Benzoimidazole-5-Carboxamidine	0.009	0.991	1	0.166	0.834	1
MATE1	Valethamate	0.009	0.991	1	0.233	0.767	1
MATE1	Timepidium	0.009	0.991	1	0.236	0.764	1
MATE1	[[CYCLOHEXANESULFONYL-GLYCYL]-3[PYRIDIN-4-YL-AMINOMETHYL]ALANYL]PIPERIDINE	0.009	0.991	1	0.332	0.668	1
MATE1	2-[4-(DIMETHYLAMINO)PHENYL]-6-HYDROXY-3-METHYL-1.3-BENZOTHIAZOL-3-IUM	0.009	0.991	1	0.355	0.645	1
MATE1	Carbuterol	0.010	0.990	1	0.155	0.845	1
MATE1	Homatropine methylbromide	0.010	0.990	1	0.168	0.832	1
MATE1	Adrenalone	0.010	0.990	1	0.204	0.796	1
MATE1	Melarsomine	0.010	0.990	1	0.281	0.719	1
MATE1	Hexoprenaline	0.010	0.990	1	0.344	0.656	1
MATE1	1-GUANIDINO-4-(N-PHENYLMETHANESULFONYL-L-LEUCYL-L-PROLYLAMINO)BUTANE	0.010	0.990	1	0.348	0.652	1
MATE1	Bis(5-Amidino-2-Benzimidazolyl)Methane Ketone Hydrate	0.010	0.990	1	0.358	0.642	1
MATE1	Atosiban	0.010	0.990	1	0.427	0.573	1
MATE1	Selepressin	0.010	0.990	1	0.444	0.556	1
MATE1	CTCE-9908	0.010	0.990	1	0.510	0.490	0
MATE1	N'-((2S.3R)-3-AMINO-2-HYDROXY-5-(ISOPROPYLSULFANYL)PENTANOYL)-N-3-CHLOROBENZOYL HYDRAZIDE	0.011	0.989	1	0.206	0.794	1
MATE1	S.S'-(1.3-Phenylene-Bis(1.2-Ethanediyl))Bis-Isothiourea	0.011	0.989	1	0.206	0.794	1
MATE1	S.S'-(1.4-Phenylene-Bis(1.2-Ethanediyl))Bis-Isothiourea	0.011	0.989	1	0.206	0.794	1
MATE1	Thiazinam	0.011	0.989	1	0.292	0.708	1
MATE1	Sulfaguanidine	0.012	0.988	1	0.167	0.833	1
MATE1	2-(2-Hydroxy-Phenyl)-1h-Indole-5-Carboxamidine	0.012	0.988	1	0.178	0.822	1
MATE1	N-[(1S)-1-(aminocarbonyl)-4-(ethanimidoylamino)butyl]benzamide	0.012	0.988	1	0.231	0.769	1

Transport	Substance	HGB P(0)	HGB P(1)	Prediction HGB	RF P(0)	RF P(1)	Prediction RFC
MATE1	2-O-(4'-AMIDINOPHENYL)-5-O-(3''-AMIDINOPHENYL)-1.4:3.6-DIANHYDRO-D-SORBITOL	0.012	0.988	1	0.274	0.726	1
MATE1	(1s.2s)-1-Amino-1-(1.3-Thiazol-2-Yl)Propan-2-Ol	0.012	0.988	1	0.319	0.681	1
MATE1	1-GUANIDINO-4-(N-NITRO-BENZOYLAMINO-L-LEUCYL-L-PROLYLAMINO)BUTANE	0.012	0.988	1	0.399	0.601	1
MATE1	Dusquetide	0.012	0.988	1	0.410	0.590	1
MATE1	Bombesin	0.012	0.988	1	0.469	0.531	1
MATE1	Sar9. Met (O2)11-Substance P	0.012	0.988	1	0.473	0.527	1
MATE1	Hydroxyethylpromethazine	0.013	0.987	1	0.189	0.811	1
MATE1	4-({4-[(4-AMINOBUT-2-YNYL)OXY]PHENYL}SULFONYL)-N-HYDROXY-2.2-DIMETHYLTHIOMORPHOLINE-3-CARBOXAMIDE	0.013	0.987	1	0.294	0.706	1
MATE1	Quisinostat	0.013	0.987	1	0.312	0.688	1
MATE1	5'-{[4-(aminooxy)butyl](methyl)amino}-5'-deoxy-8-ethenyladenosine	0.013	0.987	1	0.331	0.669	1
MATE1	N-[1-(4-CARBAMIMIDOYL-BENZYLCARBAMOYL)-3-METHYLSULFANYL-PROPYL]-3-HYDROXY-2-PROPOXYAMINO-BUTYRAMID	0.013	0.987	1	0.406	0.594	1
MATE1	Guanoclor	0.014	0.986	1	0.197	0.803	1
MATE1	8-Aminotheophylline	0.014	0.986	1	0.225	0.775	1
MATE1	CRA_1802	0.014	0.986	1	0.235	0.765	1
MATE1	N-METHYL-1-[4-(9H-PURIN-6-YL)PHENYL]METHANAMINE	0.014	0.986	1	0.297	0.703	1
MATE1	Leupeptin	0.014	0.986	1	0.388	0.612	1
MATE1	Delparantag	0.014	0.986	1	0.450	0.550	1
MATE1	Talabostat	0.015	0.985	1	0.207	0.793	1
MATE1	2-[2-ETHANESULFONYLAMINO-3-(1H-INDOL-3-YL)-PROPIONYLAMINO]-PENTANEDIOIC ACID 5-AMIDE 1-(4-CARBAMIM IDOYL-BENZYLAMIDE)	0.015	0.985	1	0.389	0.611	1
MATE1	(2S)-1-(6H-INDOL-3-YL)-3-{[5-(7H-PYRAZOLO[3.4-C]PYRIDIN-5-YL)PYRIDIN-3-YL]OXY}PROPAN-2-AMINE	0.015	0.985	1	0.398	0.602	1
MATE1	MM3122	0.015	0.985	1	0.414	0.586	1
MATE1	Barusiban	0.015	0.985	1	0.419	0.581	1
MATE1	Cyclotheonamide A	0.015	0.985	1	0.433	0.567	1
MATE1	Desmopressin	0.015	0.985	1	0.495	0.505	1
MATE1	Prexasertib	0.016	0.984	1	0.185	0.815	1
MATE1	Theodrenaline	0.016	0.984	1	0.188	0.812	1
MATE1	Cimetropium	0.017	0.983	1	0.099	0.901	1
MATE1	Bamethan	0.017	0.983	1	0.144	0.856	1
MATE1	Emetonium iodide	0.017	0.983	1	0.239	0.761	1
MATE1	RWJ-56423	0.017	0.983	1	0.297	0.703	1
MATE1	4-Amino-5-Hydroxymethyl-2-Methylpyrimidine	0.017	0.983	1	0.389	0.611	1

Transport	Substance	HGB P(0)	HGB P(1)	Prediction HGB	RF P(0)	RF P(1)	Prediction RFC
MATE1	1.4:3.6-Dianhydro-2-O-(3-carbamimidoylphenyl)-5-O-(4-carbamimidoylphenyl)-D-glucitol	0.018	0.982	1	0.288	0.712	1
MATE1	2.5-O.O-BIS-{4'.4''-AMIDINOPHENYL}-1.4:3.6-DIANHYDRO-D-SORBITOL	0.018	0.982	1	0.298	0.702	1
MATE1	2-[2-ETHANESULFONYLAMINO-3-(5-PROPOXY-1H-INDOL-3-YL)-PROPIONYLAMINO]-PENTANEDIOIC ACID 5-AMIDE 1-(4-CARBAMIMIDOYL-BENZYLAMIDE)	0.018	0.982	1	0.406	0.594	1
MATE1	(2R)-N-[(2R)-2-(DIHYDROXYBORYL)-1-L-PROLYLPYRROLIDIN-2-YL]-N-[(5R)-5-(DIHYDROXYBORYL)-1-L-PROLYLPYRROLIDIN-2-YL]-L-PROLINAMIDE	0.019	0.981	1	0.161	0.839	1
MATE1	AC-(D)PHE-PRO-BOROHOMOORNITHINE-OH	0.019	0.981	1	0.272	0.728	1
MATE1	Bedoradrine	0.019	0.981	1	0.357	0.643	1
MATE1	Levosulpiride	0.020	0.980	1	0.120	0.880	1
MATE1	+/-METHYL 4-(AMINOIMINOMETHYL)-BETA-[3- INH (AMINOIMINO)PHENYL]BENZENE PENTANOATE	0.020	0.980	1	0.166	0.834	1
MATE1	ABT-510	0.020	0.980	1	0.466	0.534	1
MATE2	2-[N'-(4-AMINO-BUTYL)-HYDRAZINOCARBONYL]-PYRROLIDINE-1-CARBOXYLIC ACID BENZYL ESTER	0.009	0.991	1	0.235	0.765	1
MATE2	Anisotropine methylbromide	0.010	0.990	1	0.263	0.737	1
MATE2	Melarsomine	0.010	0.990	1	0.302	0.698	1
MATE2	1-ETHOXYCARBONYL-D-PHE-PRO-2(4-AMINOBUTYL)HYDRAZINE	0.010	0.990	1	0.338	0.662	1
MATE2	Bibenzonium	0.011	0.989	1	0.316	0.684	1
MATE2	N-(3.5-dimethoxyphenyl)imidodicarbonimidic diamide	0.012	0.988	1	0.219	0.781	1
MATE2	2-(2-hydroxy-phenyl)-3H-benzoimidazole-5-carboxamidine	0.012	0.988	1	0.223	0.777	1
MATE2	Pixantrone	0.012	0.988	1	0.295	0.705	1
MATE2	1-[(4S)-4-amino-5-(1.3-benzothiazol-2-yl)-5-oxopentyl]guanidine	0.013	0.987	1	0.218	0.782	1
MATE2	Bephenium	0.015	0.985	1	0.324	0.676	1
MATE2	Levosulpiride	0.016	0.984	1	0.122	0.878	1
MATE2	Valethamate	0.016	0.984	1	0.249	0.751	1
MATE2	CRA_1144	0.016	0.984	1	0.280	0.720	1
MATE2	(3R)-4-[(3R)-3-AMINO-4-(2.4.5-TRIFLUOROPHENYL)BUTANOYL]-3-METHYL-1.4-DIAZEPAN-2-ONE	0.016	0.984	1	0.370	0.630	1
MATE2	Tiapride	0.017	0.983	1	0.376	0.624	1
OCT1	Methylbenactyzium	0.000	1.000	1	0.044	0.956	1

Transport	Substance	HGB P(0)	HGB P(1)	Prediction HGB	RF P(0)	RF P(1)	Prediction RFC
OCT1	Levosalbutamol	0.001	0.999	1	0.028	0.972	1
OCT1	Penthienate	0.001	0.999	1	0.053	0.947	1
OCT1	Poldine	0.001	0.999	1	0.055	0.945	1
OCT1	Pipenzolate	0.001	0.999	1	0.060	0.940	1
OCT1	2.6-Diamino-8-(2-Dimethylaminoethylsulfanylmethyl)-3h-Quinazolin-4-One	0.001	0.999	1	0.063	0.937	1
OCT1	Bevonium	0.001	0.999	1	0.063	0.937	1
OCT1	N'-((2S.3R)-3-AMINO-2-HYDROXY-5-(ISOPROPYLSULFANYL)PENTANOYL)-N-3-CHLOROBENZOYL HYDRAZIDE	0.001	0.999	1	0.111	0.889	1
OCT1	4-Sulfonamide-[4-(Thiomethylaminobutane)]Benzamide	0.001	0.999	1	0.121	0.879	1
OCT1	Anisotropine methylbromide	0.001	0.999	1	0.128	0.872	1
OCT1	Bibenzonium	0.001	0.999	1	0.157	0.843	1
OCT1	Bephenium	0.001	0.999	1	0.165	0.835	1
OCT1	Methylatropine	0.002	0.998	1	0.097	0.903	1
OCT1	2-[N'-(4-AMINO-BUTYL)-HYDRAZINOCARBONYL]-PYRROLIDINE-1-CARBOXYLIC ACID BENZYL ESTER	0.002	0.998	1	0.148	0.852	1
OCT1	Valethamate	0.002	0.998	1	0.161	0.839	1
OCT1	Emetonium iodide	0.002	0.998	1	0.173	0.827	1
OCT1	N-(2-Aminoethyl)-5-Chloroisoquinoline-8-Sulfonamide	0.003	0.997	1	0.103	0.897	1
OCT1	Methantheline	0.003	0.997	1	0.112	0.888	1
OCT1	Chelerythrine	0.003	0.997	1	0.114	0.886	1
OCT1	(1S)-2-[(2S.5R)-2-(AMINOMETHYL)-5-ETHYNYLPYRROLIDIN-1-YL]-1-CYCLOPENTYL-2-OXOETHANAMINE	0.003	0.997	1	0.158	0.842	1
OCT1	3.5.6.8-Tetramethyl-N-Methyl Phenanthrolinium	0.003	0.997	1	0.301	0.699	1
OCT1	Levosulpiride	0.004	0.996	1	0.041	0.959	1
OCT1	Tiemonium iodide	0.004	0.996	1	0.106	0.894	1
OCT1	NB-001	0.004	0.996	1	0.153	0.847	1
OCT1	Ambazone	0.004	0.996	1	0.176	0.824	1
OCT1	2-(2-Hydroxy-Phenyl)-1h-Indole-5-Carboxamidine	0.004	0.996	1	0.178	0.822	1
OCT1	Guanoclor	0.004	0.996	1	0.185	0.815	1
OCT1	Hexocyclium	0.004	0.996	1	0.242	0.758	1
OCT1	N-[2-(METHYLAMINO)ETHYL]-5-ISOQUINOLINESULFONAMIDE	0.005	0.995	1	0.092	0.908	1
OCT1	(2S)-4-(4-fluorobenzyl)-N-(2-sulfanylethyl)piperazine-2-carboxamide	0.005	0.995	1	0.138	0.862	1
OCT1	4-(2.5-DIAMINO-5-HYDROXY-PENTYL)-PHENOL	0.005	0.995	1	0.143	0.857	1
OCT1	Cariporide	0.005	0.995	1	0.144	0.856	1
OCT1	4-(METHYLSULFONYL)BENZENE CARBOXIMIDAMIDE	0.005	0.995	1	0.153	0.847	1

Transport	Substance	HGB P(0)	HGB P(1)	Prediction HGB	RF P(0)	RF P(1)	Prediction RFC
OCT1	1-[(4S)-4-amino-5-(1.3-benzothiazol-2-yl)-5-oxopentyl]guanidine	0.005	0.995	1	0.189	0.811	1
OCT1	1-(4-Amidinophenyl)-3-(4-Chlorophenyl)Urea	0.005	0.995	1	0.193	0.807	1
OCT1	(1R.2R)-N-(2-Aminoethyl)-2-{[(4-methoxyphenyl)sulfonyl]methyl}cyclohexanecarboxamide	0.005	0.995	1	0.207	0.793	1
OCT1	Benzilone	0.006	0.994	1	0.111	0.889	1
OCT1	Cimetropium	0.006	0.994	1	0.113	0.887	1
OCT1	Lunasine	0.006	0.994	1	0.156	0.844	1
OCT1	1-butanoyl-N-(4-carbamimidoylbenzyl)-L-prolinamide	0.006	0.994	1	0.187	0.813	1
OCT1	PXS-5505	0.006	0.994	1	0.192	0.808	1
OCT1	Lapyrium	0.006	0.994	1	0.192	0.808	1
OCT1	2-(2-hydroxy-phenyl)-3H-benzoimidazole-5-carboxamidine	0.006	0.994	1	0.197	0.803	1
OCT1	Thiazinam	0.006	0.994	1	0.205	0.795	1
OCT1	Ciclonium bromide	0.006	0.994	1	0.234	0.766	1
OCT1	2-[2-(2-Cyclohexyl-2-guanidino-acetylamino)-acetylamino]-N-(3-mercapto-propyl)-propionamide	0.006	0.994	1	0.346	0.654	1
OCT1	Epanolol	0.007	0.993	1	0.157	0.843	1
OCT1	Timepidium	0.007	0.993	1	0.160	0.840	1
OCT1	Nimustine	0.007	0.993	1	0.176	0.824	1
OCT1	Guanazodine	0.007	0.993	1	0.187	0.813	1
OCT1	Alagebrium	0.007	0.993	1	0.205	0.795	1
OCT1	Tiapride	0.007	0.993	1	0.213	0.787	1
OCT1	SC45647	0.007	0.993	1	0.244	0.756	1
OCT1	N-[(1S)-5-amino-1-(chloroacetyl)pentyl]-4-methylbenzenesulfonamide	0.007	0.993	1	0.266	0.734	1
OCT1	6-(2-HYDROXY-CYCLOPENTYL)-7-OXO-HEPTANAMIDINE	0.008	0.992	1	0.127	0.873	1
OCT1	N-(3.5-dimethoxyphenyl)imidodicarbonimidic diamide	0.008	0.992	1	0.148	0.852	1
OCT1	4-{[(2S)-3-(tert-butylamino)-2-hydroxypropyl]oxy}-3H-indole-2-carbonitrile	0.008	0.992	1	0.181	0.819	1
OCT1	Tridihexethyl	0.008	0.992	1	0.231	0.769	1
OCT1	Diethylnorspermine	0.008	0.992	1	0.243	0.757	1
OCT1	4-(2-aminoethyl)-2-ethylphenol	0.008	0.992	1	0.248	0.752	1
OCT1	Oxilofrine	0.009	0.991	1	0.094	0.906	1
OCT1	Exametazime	0.009	0.991	1	0.125	0.875	1
OCT1	Veliparib	0.009	0.991	1	0.145	0.855	1
OCT1	1-[4-(hydroxymethyl)phenyl]guanidine	0.009	0.991	1	0.167	0.833	1
OCT1	(3R)-4-[(3R)-3-AMINO-4-(2.4.5-TRIFLUOROPHENYL)BUTANOYL]-3-METHYL-1.4-DIAZEPAN-2-ONE	0.009	0.991	1	0.179	0.821	1
OCT1	Guanoxan	0.009	0.991	1	0.183	0.817	1
OCT1	Rimeporide	0.009	0.991	1	0.190	0.810	1

Transport	Substance	HGB P(0)	HGB P(1)	Prediction HGB	RF P(0)	RF P(1)	Prediction RFC
OCT1	5-Amidino-Benzimidazole	0.009	0.991	1	0.241	0.759	1
OCT1	Gallamine triethiodide	0.009	0.991	1	0.383	0.617	1
OCT1	Rimiterol	0.010	0.990	1	0.097	0.903	1
OCT1	Oxitropium	0.010	0.990	1	0.106	0.894	1
OCT1	N-Methylnaloxonium	0.010	0.990	1	0.110	0.890	1
OCT1	Zilpaterol	0.010	0.990	1	0.141	0.859	1
OCT1	L-alanyl-N-[(1S.2R)-1-benzyl-2-hydroxypropyl]-L-alaninamide	0.010	0.990	1	0.143	0.857	1
OCT1	GLYCYLALANYL-N-2-NAPHTHYL-L-PROLINEAMIDE	0.010	0.990	1	0.189	0.811	1
OCT1	CRA_1144	0.010	0.990	1	0.242	0.758	1
OCT1	N-{(4S)-4-Amino-5-[(2-aminoethyl)amino]pentyl}-N'-nitroguanidine	0.010	0.990	1	0.314	0.686	1
OCT1	Hydroxy(oxo)(2-{(1S)-2.2.2-trifluoro-1-[2-(trimethylarsonio)ethoxy]ethyl}phenyl)ammonium	0.011	0.989	1	0.194	0.806	1
OCT1	Amprolium	0.011	0.989	1	0.223	0.777	1
OCT1	2.4-Diamino-6-Phenyl-5.6.7.8.-Tetrahydropteridine	0.011	0.989	1	0.225	0.775	1
OCT1	Phenglutarimide	0.011	0.989	1	0.275	0.725	1
OCT1	Ticalopride	0.012	0.988	1	0.161	0.839	1
OCT1	Guanadrel	0.012	0.988	1	0.177	0.823	1
OCT1	4-[(6-Amino-4-Pyrimidinyl)Amino]Benzenesulfonamide	0.012	0.988	1	0.199	0.801	1
OCT1	Rimazolium	0.012	0.988	1	0.248	0.752	1
OCT1	4-{[(2.6-difluorophenyl)carbonyl]amino}-N-[(3S)-piperidin-3-yl]-1H-pyrazole-3-carboxamide	0.012	0.988	1	0.249	0.751	1
OCT1	(1R.2R.3R.4S.5R)-4-(Benzylamino)-5-(methylthio)cyclopentane-1.2.3-triol	0.013	0.987	1	0.143	0.857	1
OCT1	Meladrazine	0.013	0.987	1	0.168	0.832	1
OCT1	8-(Pyrimidin-2-Ylamino)Naphthalene-2-Carboximidamide	0.013	0.987	1	0.211	0.789	1
OCT1	4-(2-AMINOETHYL)BENZENESULFONAMIDE	0.014	0.986	1	0.187	0.813	1
OCT1	Y-27632	0.014	0.986	1	0.192	0.808	1
OCT1	PF-06305591	0.014	0.986	1	0.238	0.762	1
OCT1	(2R.3R.4R.5S)-2-(HYDROXYMETHYL)-1-NONYLPIPERIDINE-3.4.5-TRIOL	0.015	0.985	1	0.225	0.775	1
OCT1	(3-EXO)-3-(10.11-DIHYDRO-5H-DIBENZO[A.D][7]ANNULEN-5-YLOXY)-8.8-DIMETHYL-8-AZONIABICYCLO[3.2.1]OCTANE	0.015	0.985	1	0.248	0.752	1
OCT1	Hydroxyethylpromethazine	0.016	0.984	1	0.157	0.843	1
OCT1	Gefapixant	0.016	0.984	1	0.186	0.814	1
OCT1	4-AMINO-2-OCTYLOXY-6-HYDROXYMETHYL-TETRAHYDRO-PYRAN-3.5-DIOL	0.017	0.983	1	0.151	0.849	1
OCT1	Resminostat	0.017	0.983	1	0.205	0.795	1

Transport	Substance	HGB P(0)	HGB P(1)	Prediction HGB	RF P(0)	RF P(1)	Prediction RFC
OCT1	Arbutamine	0.017	0.983	1	0.253	0.747	1
OCT1	Prexasertib	0.017	0.983	1	0.278	0.722	1
OCT1	L-Histidine Beta Naphthylamide	0.018	0.982	1	0.208	0.792	1
OCT1	Cu-Cyclam	0.019	0.981	1	0.312	0.688	1
OCT1	N-METHYL-1-[4-(9H-PURIN-6-YL)PHENYL]METHANAMINE	0.020	0.980	1	0.137	0.863	1
OCT1	N-[3-(aminomethyl)benzyl] acetamidine	0.020	0.980	1	0.174	0.826	1
OCT1	Metocurine iodide	0.020	0.980	1	0.376	0.624	1
OCT1	Pipecuronium	0.020	0.980	1	0.539	0.461	0
OCT2	Poldine	0.003	0.997	1	0.126	0.874	1
OCT2	Bevonium	0.003	0.997	1	0.146	0.854	1
OCT2	Pipenzolate	0.003	0.997	1	0.144	0.856	1
OCT2	Timepidium	0.004	0.996	1	0.185	0.815	1
OCT2	Bibenzonium	0.004	0.996	1	0.254	0.746	1
OCT2	Methylatropine	0.005	0.995	1	0.143	0.857	1
OCT2	Methylbenactyzium	0.005	0.995	1	0.170	0.830	1
OCT2	Anisotropine methylbromide	0.005	0.995	1	0.249	0.751	1
OCT2	Methantheline	0.006	0.994	1	0.138	0.862	1
OCT2	Hydroxyethylpromethazine	0.006	0.994	1	0.150	0.850	1
OCT2	Penthienate	0.007	0.993	1	0.187	0.813	1
OCT2	Oxitropium	0.007	0.993	1	0.149	0.851	1
OCT2	Chelerythrine	0.008	0.992	1	0.178	0.822	1
OCT2	Thiazinam	0.008	0.992	1	0.232	0.768	1
OCT2	Cimetropium	0.009	0.991	1	0.130	0.870	1
OCT2	Bephenium	0.010	0.990	1	0.239	0.761	1
OCT2	N-Methylnaloxonium	0.011	0.989	1	0.204	0.796	1
OCT2	Hexocyclium	0.012	0.988	1	0.324	0.676	1
OCT2	Tiemonium iodide	0.012	0.988	1	0.269	0.731	1
OCT2	(1S)-2-[(2S.5R)-2-(AMINOMETHYL)-5-ETHYNYLPYRROLIDIN-1-YL]-1-CYCLOPENTYL-2-OXOETHANAMINE	0.013	0.987	1	0.299	0.701	1
OCT2	Hydroxy(oxo)(2-{(1S)-2.2.2-trifluoro-1-[2-(trimethylarsonio) ethoxy]ethyl}phenyl)ammonium	0.013	0.987	1	0.370	0.630	1
OCT2	Amprolium	0.013	0.987	1	0.316	0.684	1
OCT2	Homatropine methylbromide	0.014	0.986	1	0.235	0.765	1
OCT2	Lunasine	0.016	0.984	1	0.253	0.747	1
OCT2	Levosalbutamol	0.017	0.983	1	0.212	0.788	1
OCT2	1-[4-(hydroxymethyl) phenyl]guanidine	0.017	0.983	1	0.251	0.749	1
OCT2	Guanadrel	0.020	0.980	1	0.268	0.732	1

Each line refers to the transporter and the substance listed in the respective columns. **HGB**, Histogram Gradient Boosting Classifier; **RF**, Random forest classifier. **P(0)** is the probability that a substance is NOT a substrate of the respective transporter, and **P(1)** is the probability that the substance is a substrate of the transporter. Predictions columns by HGB and RF are based on the default cutoff of both classifiers, which is much lower than 0.98. Only those predictions are shown here, which have a probability of at least 0.98 according to the HGB classifier. As shown, predictions by the RF classifier are not identical but often in the same direction.
